# Supplementary material for: Investigating room temperature ferroelectric nematogens and their structure-property relationships
Source: Nat Commun. 2026 Feb 20;17:2965. doi: 10.1038/s41467-026-69484-z (PMC13035879; doi:10.1038/s41467-026-69484-z)
Supplement: Supplementary file 1 — Supplementary Information [file 41467_2026_69484_MOESM1_ESM.pdf]

Supplementary Information

Naila Tufaha[1], Gytis Stepanafas[1], Ewan Cruickshank[1,‡,*], Damian Pociecha[2], Ewa Gorecka[2], John M.D. Storey[1], Rebecca Walker[1] & Corrie T. Imrie[1,†]

[1]Department of Chemistry, University of Aberdeen, Old Aberdeen, AB24 3UE, U.K.

[2]Faculty of Chemistry, University of Warsaw, Zwirki i Wigury 101, 02-089 Warsaw, Poland

‡Present Address: School of Pharmacy, Applied Sciences and Public Health, Robert Gordon University, Aberdeen, AB10 7GJ, U.K.

[†]Deceased 14th January 2025

*Author for correspondence: e.cruickshank2@rgu.ac.uk

Supplementary Methods

Synthesis

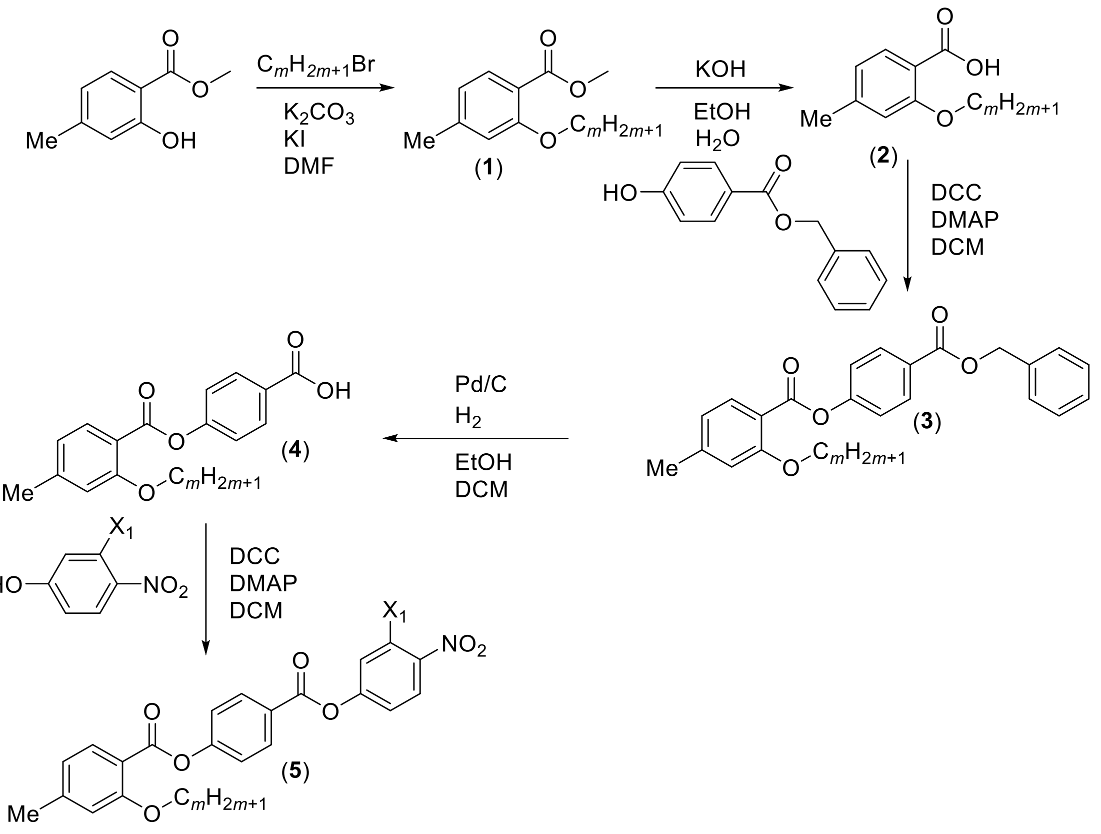

A.*m* => X_1 = H
B.*m* => X_1 = F

Figure SI1. Synthetic scheme for the A.*m* and B.*m* series. The compounds were synthesised using a five-step reaction with the final step being a Steglich esterification.

Methyl 2-alkyloxy-4-methylbenzoates (1)

To a pre-dried flask flushed with argon and fitted with a condenser, methyl 4-methoxysilicylate (1 eq, 4.00 g, 0.0241 mol), potassium iodide (1 eq, 4.00 g, 0.0241 mol), and potassium carbonate (3 eq, 9.98 g, 0.0723 mol) were combined in DMF (30 mL). To the mixture, the appropriate 1-bromoalkane (1.2 eq) was added and stirred at 90°C overnight. The quantities of the 1-bromoalkanes used in each reaction are listed in **Table SI1**. The extent of the reaction was monitored by TLC using a suitable solvent system (RF values quoted in the product data). The reaction mixture was cooled to room temperature and poured into water (100 mL). The resulting suspension was extracted with ethyl acetate (3 × 100 mL). The organic fractions were combined, washed with water (3 × 100 mL) and dried over anhydrous magnesium sulfate. The magnesium sulfate was removed using vacuum filtration and the solvent evaporated under vacuum to leave a yellow or colourless oil. The product was carried forwards without any further purification.

Table SI1. Quantities of 1-bromoalkanes used in the syntheses of the methyl 2-alkoxy-4-methoxybenzoates

m	1-Bromoalkane
2	2.16 mL, 3.15 g, 0.0289 mol
3	2.62 mL, 3.55 g, 0.0289 mol
4	3.11 mL, 3.96 g, 0.0289 mol
5	3.58 mL, 4.36 g, 0.0289 mol
6	4.06 mL, 4.77 g, 0.0289 mol
7	4.54 mL, 5.17 g, 0.0289 mol
8	4.99 mL, 5.58 g, 0.0289 mol

1.1 Methyl 2-ethoxy-4-methylbenzoate

Colourless oil. Yield: 3.67 g, 79 %. RF: 0.45 (10 % ethyl acetate:90 % 40:60 petroleum ether).

ν_{max}/cm^{-1}: 2981 (C-H), 1727 (C=O), 1573 (Ar C=C), 1503 (Ar C=C)

δ_H/ppm (400 MHz, $CDCl_3$): 7.55 (d, *J* = 7.8 Hz, 1H, Ar-H), 6.94 (s, 1H, Ar-H), 6.80 (d, *J* = 7.6 Hz, 1H, Ar-H), 4.06 (q, *J* = 6.9 Hz, 2H, O$\underline{CH_2}$$CH_3$), 3.75 (s, 3H, OCH_3), 2.32 (s, 3H, Ar-CH_3), 1.32 (t, *J* = 6.9 Hz, 3H, $OCH_2\underline{CH_3}$)

δ_C/ppm (100 MHz, $CDCl_3$): 166.84, 158.89, 144.49, 131.86, 121.06, 117.51, 114.29, 64.68, 51.84, 22.00, 14.89

1.2 Methyl 4-methyl-2-propoxybenzoate

Colourless oil. Yield: 3.32 g, 66 %. RF: 0.51 (10 % ethyl acetate:90 % 40:60 petroleum ether).

ν_{max}/cm^{-1}: 2948 (C-H), 1727 (C=O), 1574 (Ar C=C), 1502 (Ar C=C)

δ_H/ppm (400 MHz, $CDCl_3$): 7.56 (d, *J* = 7.8 Hz, 1H, Ar-H), 6.93 (s, 1H, Ar-H), 6.79 (d, *J* = 7.8 Hz, 1H, Ar-H), 3.95 (t, *J* = 6.3 Hz, 2H, O$\underline{CH_2}$$CH_2CH_3$), 3.75 (s, 3H, OCH_3), 2.32 (s, 3H, Ar-CH_3), 1.71 (sext, 2H, *J* = 7.3 Hz, $OCH_2\underline{CH_2}CH_3$), 0.99 (t, *J* = 7.4 Hz, 3H, $OCH_2CH_2\underline{CH_3}$)

δ_C/ppm (100 MHz, $CDCl_3$): 166.92, 158.99, 144.42, 131.84, 120.91, 117.42, 114.09, 70.43, 51.75, 22.69, 21.93, 10.60

1.3 Methyl 2-butoxy-4-methylbenzoate

Yellow oil. Yield: 3.34 g, 63 %. RF: 0.51 (10 % ethyl acetate:90 % 40:60 petroleum ether).

ν_{max}/cm^{-1}: 2955 (C-H), 1727 (C=O), 1574 (Ar C=C), 1503 (Ar C=C)

δ_H/ppm (400 MHz, $CDCl_3$): 7.55 (d, *J* = 7.8 Hz, 1H, Ar-H), 6.95 (s, 1H, Ar-H), 6.80 (d, *J* = 7.8 Hz, 1H, Ar-H), 4.00 (t, *J* = 6.3 Hz, 2H, O$\underline{CH_2}$$(CH_2)_2CH_3$), 3.75 (s, 3H, OCH_3), 2.33 (s, 3H, Ar-CH_3), 1.73 – 1.64 (m, 2H, $OCH_2\underline{CH_2}CH_2CH_3$), 1.46 (sext, *J* = 7.3 Hz, 2H, $O(CH_2)_2\underline{CH_2}CH_3$), 0.93 (t, *J* = 7.4 Hz, 3H, $O(CH_2)_3\underline{CH_3}$)

δ_C/ppm (100 MHz, $CDCl_3$): 166.88, 158.99, 144.40, 131.82, 120.89, 117.42, 114.06, 68.63, 51.73, 31.35, 21.92, 19.26, 13.89

1.4 Methyl 4-methyl-2-pentoxybenzoate

Yellow oil. Yield: 4.27 g, 75 %. RF: 0.60 (10 % ethyl acetate:90 % 40:60 petroleum ether).

ν_{max}/cm^{-1}: 2951 (C-H), 1728 (C=O), 1574 (Ar C=C), 1503 (Ar C=C)

δ_H/ppm (400 MHz, $CDCl_3$): 7.55 (d, *J* = 7.7 Hz, 1H, Ar-H), 6.94 (s, 1H, Ar-H), 6.80 (d, *J* = 7.9 Hz, 1H, Ar-H), 3.99 (t, *J* = 6.3 Hz, 2H, $O\underline{CH_2}(CH_2)_3CH_3$), 3.75 (s, 3H, OCH_3), 2.32 (s, 3H, Ar-CH_3), 1.69 (quin, *J* = 6.7 Hz, 2H, $OCH_2\underline{CH_2}(CH_2)_2CH_3$), 1.47 – 1.29 (m, 4H, $OCH_2CH_2(\underline{CH_2})_2CH_3$), 0.89 (t, *J* = 7.1 Hz, 3H, $O(CH_2)_4\underline{CH_3}$)

δ_C/ppm (100 MHz, $CDCl_3$): 166.98, 159.00, 144.43, 131.87, 120.93, 117.46, 114.11, 69.00, 51.78, 29.00, 28.23, 22.50, 21.96, 14.13

1.5 Methyl 2-hexyloxy-4-methylbenzoate

Yellow oil. Yield: 5.11 g, 85 %. RF: 0.60 (10 % ethyl acetate:90 % 40:60 petroleum ether).

ν_{max}/cm^{-1}: 2929 (C-H), 1729 (C=O), 1574 (Ar C=C), 1503 (Ar C=C)

δ_H/ppm (400 MHz, $CDCl_3$): 7.55 (d, *J* = 7.8 Hz, 1H, Ar-H), 6.94 (s, 1H, Ar-H), 6.80 (d, *J* = 7.9 Hz, 1H, Ar-H), 3.99 (t, *J* = 6.3 Hz, 2H, $O\underline{CH_2}(CH_2)_4CH_3$), 3.74 (s, 3H, OCH_3), 2.32 (s, 3H, Ar-CH_3), 1.69 (quin, *J* = 6.7 Hz, 2H, $OCH_2\underline{CH_2}(CH_2)_3CH_3$), 1.49 – 1.23 (m, 6H, $OCH_2CH_2(\underline{CH_2})_3CH_3$), 0.88 (t, *J* = 7.0 Hz, 3H, $O(CH_2)_5\underline{CH_3}$)

δ_C/ppm (100 MHz, $CDCl_3$): 166.97, 158.98, 144.41, 131.86, 120.91, 117.45, 114.08, 68.98, 51.76, 31.62, 29.27, 25.73, 22.69, 21.94, 14.10

1.6 Methyl 2-heptyloxy-4-methylbenzoate

Yellow oil. Yield: 5.51 g, 87 %. RF: 0.68 (70 % dichloromethane:30 % 40:60 petroleum ether).

ν_{max}/cm^{-1}: 2926 (C-H), 1729 (C=O), 1575 (Ar C=C), 1503 (Ar C=C)

δ_H/ppm (400 MHz, $CDCl_3$): 7.69 (d, *J* = 8.1 Hz, 1H, Ar-H), 6.77 – 6.71 (m, 2H, Ar-H), 3.99 (t, *J* = 6.5 Hz, 2H, $O\underline{CH_2}(CH_2)_5CH_3$), 3.84 (s, 3H, OCH_3), 2.34 (s, 3H, Ar-CH_3), 1.81 (quin, *J* = 6.5 Hz, 2H, $OCH_2\underline{CH_2}(CH_2)_4CH_3$), 1.55 – 1.20 (m, 8H, $OCH_2CH_2(\underline{CH_2})_4CH_3$), 0.88 (t, *J* = 7.0 Hz, 3H, $O(CH_2)_6\underline{CH_3}$)

δ_C/ppm (100 MHz, $CDCl_3$): 166.95, 158.96, 144.39, 131.83, 120.88, 117.41, 114.05, 68.95, 51.73, 31.86, 29.28, 29.08, 25.98, 22.67, 21.91, 14.13

1.7 Methyl 4-methyl-2-octyloxybenzoate

Yellow oil. Yield: 4.34 g, 65 %. RF: 0.68 (70 % dichloromethane:30 % 40:60 petroleum ether).

v_{max}/cm^{-1}: 2924 (C-H), 1730 (C=O), 1575 (Ar C=C), 1503 (Ar C=C)

δ_H/ppm (400 MHz, $CDCl_3$): 7.68 (d, *J* = 8.1 Hz, 1H, Ar-H), 6.80 – 6.70 (m, 2H, Ar-H), 3.99 (t, *J* = 6.5 Hz, 2H, O$\underline{CH_2}(CH_2)_6CH_3$), 3.84 (s, 3H, OCH_3), 2.33 (s, 3H, Ar-CH_3), 1.80 (quin, *J* = 6.4 Hz, 2H, $OCH_2\underline{CH_2}(CH_2)_5CH_3$), 1.62 – 1.15 (m, 10H, $OCH_2CH_2(\underline{CH_2})_5CH_3$), 0.86 (t, *J* = 7.2 Hz, 3H, $O(CH_2)_7\underline{CH_3}$

δ_C/ppm (100 MHz, $CDCl_3$): 166.96, 158.97, 144.39, 131.83, 120.89, 117.44, 114.08, 68.98, 51.73, 31.89, 29.37, 29.32, 29.28, 26.02, 22.72, 21.91, 14.14

2-Alkyloxy-4-methylbenzoic acids (2)

To a pre-dried flask flushed with argon and fitted with a condenser, potassium hydroxide (3 eq) was added to water. **Compound 1** (1 eq) was solubilised in EtOH, added to the flask and the resultant mixture stirred at reflux overnight. The quantities of the reagents used in each reaction are listed in **Table SI2**. The extent of the reaction was monitored by TLC using an appropriate solvent system (RF values quoted in the product data). The reaction mixture was cooled to room temperature and the pH of the mixture was adjusted to 1 using 32% hydrochloric acid (25 mL). The reaction mixture was extracted with ethyl acetate (2 x 200 mL). The organic layers were combined, washed with water (2 x 100 mL) and dried over anhydrous magnesium sulfate. The magnesium sulfate was removed using vacuum filtration and the solvent evaporated under vacuum to leave a white solid or colourless oil.

Table SI2. Quantities of reagents used in the syntheses of the 2-alkyloxy-4-methylbenzoic acids

m	(**1**)	Ethanol	Potassium Hydroxide	Water
2	3.45 g, 0.0178 mol	40 mL	2.99 g, 0.0534 mol	40 mL
3	4.19 g, 0.0201 mol	50 mL	3.38 g, 0.0603 mol	50 mL
4	4.50 g, 0.0203 mol	50 mL	3.41 g, 0.0609 mol	50 mL
5	4.35 g, 0.0184 mol	50 mL	3.09 g, 0.0552 mol	50 mL
6	5.05 g, 0.0202 mol	50 mL	3.39 g, 0.0606 mol	50 mL
7	5.67 g, 0.0215 mol	50 mL	3.61 g, 0.0644 mol	50 mL
8	4.03 g, 0.0145 mol	40 mL	2.44 g, 0.0435 mol	40 mL

2.1 2-Ethoxy-4-methylbenzoic acid

White solid. Yield: 2.61 g, 82 %. RF: 0.13 (20 % ethyl acetate:80 % 40:60 petroleum ether). M.P = 75 °C

v_{max}/cm^{-1}: 2984 (C-H), 2886 (broad OH), 1654 (C=O), 1569 (Ar C=C), 1501 (Ar C=C)

δ_H/ppm (400 MHz, DMSO-d_6): 12.29 (s, 1H, COOH), 7.54 (d, *J* = 7.9 Hz, 1H, Ar-H), 6.93 (s, 1H, Ar-H), 6.79 (d, *J* = 7.8 Hz, 1H, Ar-H), 4.07 (q, *J* = 6.9 Hz, 2H, $O\underline{CH_2}CH_3$), 2.32 (s, 3H, Ar-CH_3), 1.32 (t, *J* = 6.9 Hz, 3H, $OCH_2\underline{CH_3}$)

δ_C/ppm (100 MHz, DMSO-d_6): 167.20, 157.68, 143.55, 130.97, 120.75, 118.44, 114.16, 63.98, 21.34, 14.65

2.2 4-Methyl-2-propoxybenzoic acid

White solid. Yield: 3.80 g, 97 %. RF: 0.20 (20 % ethyl acetate:80 % 40:60 petroleum ether). M.P = 65 °C

ν_{max}/cm^{-1}: 2964 (C-H), 2874 (broad OH), 1657 (C=O), 1569 (Ar C=C), 1501 (Ar C=C)

δ_H/ppm (400 MHz, DMSO-d_6): 12.27 (s, 1H, COOH), 7.55 (d, *J* = 7.8 Hz, 1H, Ar-H), 6.91 (s, 1H, Ar-H), 6.78 (d, *J* = 7.6 Hz, 1H, Ar-H), 3.96 (t, *J* = 6.4 Hz, 2H, $O\underline{CH_2}CH_2CH_3$), 2.32 (s, 3H, Ar-$CH_3$), 1.72 (sext, 2H, *J* = 7.2 Hz, $OCH_2\underline{CH_2}CH_3$), 0.99 (t, *J* = 7.4 Hz, 3H, $OCH_2CH_2\underline{CH_3}$)

δ_C/ppm (100 MHz, DMSO-d_6): 167.24, 157.88, 143.55, 130.99, 120.66, 118.36, 114.04, 69.66, 22.11, 21.33, 10.44

2.3 2-Butoxy-4-methylbenzoic acid

White solid. Yield: 4.15 g, 98 %. RF: 0.28 (20 % ethyl acetate:80 % 40:60 petroleum ether). M.P = 63 °C

ν_{max}/cm^{-1}: 2947 (C-H), 2859 (broad OH), 1660 (C=O), 1567 (Ar C=C), 1500 (Ar C=C)

δ_H/ppm (400 MHz, DMSO-d_6): 12.29 (s, 1H, COOH), 7.54 (d, *J* = 7.8 Hz, 1H, Ar-H), 6.93 (s, 1H, Ar-H), 6.78 (d, *J* = 7.8 Hz, 1H, Ar-H), 4.01 (t, *J* = 6.4 Hz, 2H, $O\underline{CH_2}(CH_2)_2CH_3$), 2.32 (s, 3H, Ar-$CH_3$), 1.69 (quin, *J* = 6.5 Hz, 2H, $OCH_2\underline{CH_2}CH_2CH_3$), 1.45 (sext, *J* = 7.3 Hz, 2H, $O(CH_2)_2\underline{CH_2}CH_3$), 0.92 (t, *J* = 7.4 Hz, 3H, $O(CH_2)_3\underline{CH_3}$))

δ_C/ppm (100 MHz, DMSO-d_6): 167.23, 157.87, 143.52, 130.96, 120.66, 118.40, 114.03, 67.89, 30.80, 21.32, 18.68, 13.67

2.4 4-Methyl-2-pentoxybenzoic acid

White solid. Yield: 3.51 g, 86 %. RF: 0.38 (20 % ethyl acetate:80 % 40:60 petroleum ether). M.P = 75 °C

ν_{max}/cm^{-1}: 2953 (C-H), 2870 (broad OH), 1657 (C=O), 1567 (Ar C=C), 1502 (Ar C=C)

δ_H/ppm (400 MHz, DMSO-d_6): 12.29 (s, 1H, COOH), 7.54 (d, *J* = 7.8 Hz, 1H, Ar-H), 6.93 (s, 1H, Ar-H), 6.78 (d, *J* = 7.7 Hz, 1H, Ar-H), 4.00 (t, *J* = 6.4 Hz, 2H, $O\underline{CH_2}(CH_2)_3CH_3$), 2.32 (s, 3H, Ar-$CH_3$), 1.71 (quin, *J* = 6.6 Hz, 2H, $OCH_2\underline{CH_2}(CH_2)_2CH_3$), 1.46 – 1.29 (m, 4H, $OCH_2CH_2\underline{(CH_2)_2}CH_3$), 0.89 (t, *J* = 7.1 Hz, 3H, $O(CH_2)_4\underline{CH_3}$)

δ_C/ppm (100 MHz, DMSO-d_6): 167.23, 157.89, 143.53, 130.97, 120.66, 118.40, 114.05, 68.20, 28.40, 27.64, 21.86, 21.33, 13.94

2.5 2-Hexyloxy-4-methylbenzoic acid

Colourless oil. Yield: 4.32 g, 91 %. RF: 0.33 (100% dichloromethane).

ν_{max}/cm^{-1}: 2926 (C-H), 2856 (broad OH), 1611 (C=O), 1574 (Ar C=C), 1504 (Ar C=C)

δ_H/ppm (400 MHz, DMSO-d$_6$): 12.23 (s, 1H, COOH), 7.54 (d, *J* = 7.8 Hz, 1H, Ar-H), 6.92 (s, 1H, Ar-H), 6.78 (d, *J* = 7.8 Hz, 1H, Ar-H), 3.99 (t, *J* = 6.4 Hz, 2H, O$\underline{CH_2}$(CH$_2$)$_4$CH$_3$), 2.32 (s, 3H, Ar-CH$_3$), 1.69 (quin, *J* = 6.7 Hz, 2H, OCH$_2\underline{CH_2}$(CH$_2$)$_3$CH$_3$), 1.48 – 1.22 (m, 6H, OCH$_2$CH$_2$($\underline{CH_2}$)$_3$CH$_3$), 0.87 (t, *J* = 6.9 Hz, 3H, O(CH$_2$)$_5\underline{CH_3}$)

δ_C/ppm (100 MHz, DMSO-d$_6$): 167.21, 157.90, 143.53, 130.98, 120.65, 118.37, 114.03, 68.19, 30.96, 28.67, 25.09, 22.11, 21.32, 13.89

2.6 2-Heptyloxy-4-methylbenzoic acid

Colourless oil. Yield: 4.56 g, 85 %. RF: 0.35 (100% dichloromethane).

ν_{max}/cm^{-1}: 2938 (C-H), 2851 (broad OH), 1662 (C=O), 1570 (Ar C=C), 1501 (Ar C=C)

δ_H/ppm (400 MHz, DMSO-d$_6$): 12.40 (s, 1H, COOH), 7.55 (d, *J* = 7.8 Hz, 1H, Ar-H), 6.91 (s, 1H, Ar-H), 6.77 (d, *J* = 7.7 Hz, 1H, Ar-H), 3.98 (t, *J* = 6.4 Hz, 2H, O$\underline{CH_2}$(CH$_2$)$_5$CH$_3$), 2.31 (s, 3H, Ar-CH$_3$), 1.69 (quin, *J* = 6.5 Hz, 2H, OCH$_2\underline{CH_2}$(CH$_2$)$_4$CH$_3$), 1.47 – 1.21 (m, 8H, OCH$_2$CH$_2$($\underline{CH_2}$)$_4$CH$_3$), 0.86 (t, *J* = 6.9 Hz, 3H, O(CH$_2$)$_6\underline{CH_3}$)

δ_C/ppm (100 MHz, DMSO-d$_6$): 167.18, 157.88, 143.51, 130.97, 120.63, 118.35, 114.03, 68.17, 31.30, 28.70, 28.41, 25.38, 22.07, 21.31, 13.96

2.7 4-Methyl-2-octyloxybenzoic acid

Colourless oil. Yield: 2.85 g, 75 %. RF: 0.43 (100% dichloromethane).

ν_{max}/cm^{-1}: 2924 (C-H), 2854 (broad OH), 1611 (C=O), 1574 (Ar C=C), 1503 (Ar C=C)

δ_H/ppm (400 MHz, DMSO-d$_6$): 12.33 (s, 1H, COOH), 7.55 (d, *J* = 7.8 Hz, 1H, Ar-H), 6.90 (s, 1H, Ar-H), 6.77 (d, *J* = 7.8 Hz, 1H, Ar-H), 3.98 (t, *J* = 6.4 Hz, 2H, O$\underline{CH_2}$(CH$_2$)$_6$CH$_3$), 2.31 (s, 3H, Ar-CH$_3$), 1.69 (quin, *J* = 6.5 Hz, 2H, OCH$_2\underline{CH_2}$(CH$_2$)$_5$CH$_3$), 1.46 – 1.17 (m, 10H, OCH$_2$CH$_2$($\underline{CH_2}$)$_5$CH$_3$), 0.85 (t, *J* = 7.1 Hz, 3H, O(CH$_2$)$_7\underline{CH_3}$)

δ_C/ppm (100 MHz, DMSO-d$_6$): 167.15, 157.90, 143.49, 130.99, 120.62, 118.35, 114.02, 68.18, 31.28, 28.75, 28.74, 28.71, 25.44, 22.14, 21.30, 13.93

4-[(Benzyloxy)carbonyl]phenyl 2-alkyloxy-4-methylbenzoates (3)

To a pre-dried flask flushed with argon, **Compound 2** (1 eq), benzyl 4-hydroxybenzoate (0.84 eq) and *N*,*N′*-dicyclohexylcarbodiimide (1.2 eq) were added. The solids were solubilised with dichloromethane (100 mL) and stirred for 2 min before 4-dimethylaminopyridine (0.084 eq) was added to the flask and the reaction was allowed to proceed overnight. The quantities of the reagents used in each reaction are listed in **Table SI3**. The extent of the reaction was monitored by TLC using an appropriate solvent system (RF values quoted in the product data). The white precipitate which formed was removed by vacuum filtration and the filtrate collected. The collected solvent was evaporated under vacuum to leave a white solid which was recrystallised from hot ethanol (40 mL).

Table SI3. Quantities of reagents used in the syntheses of the 4-[(benzyloxy)carbonyl]phenyl 2-alkyloxy-4-methylbenzoates

m	(**2**)	Benzyl 4-Hydroxybenzoate	4-Dimethylaminopyridine	*N,N'*-Dicyclohexylcarbodiimide
2	2.54 g, 0.0141 mol	2.69 g, 0.0118 mol	0.140 g, 1.18×10^{-3} mol	3.49 g, 0.0169 mol
3	2.67 g, 0.0138 mol	2.85 g, 0.0125 mol	0.150 g, 1.25×10^{-3} mol	3.36 g, 0.0166 mol
4	2.47 g, 0.0119 mol	2.36 g, 0.0108 mol	0.130 g, 1.08×10^{-3} mol	2.88 g, 0.0140 mol
5	2.68 g, 0.0121 mol	2.30 g, 0.0101 mol	0.120 g, 1.01×10^{-3} mol	2.70 g, 0.0131 mol
6	2.96 g, 0.0125 mol	2.60 g, 0.0114 mol	0.0140 g, 1.14×10^{-3} mol	3.05 g, 0.0148 mol
7	4.59 g, 0.0184 mol	3.81 g, 0.0167 mol	0.200 g, 1.67×10^{-3} mol	4.47 g, 0.0217 mol
8	2.63 g, 9.96×10^{-3} mol	2.06 g, 9.05×10^{-3} mol	0.110 g, 0.91×10^{-3} mol	2.43 g, 0.0118 mol

3.1 4-[(Benzyloxy)carbonyl]phenyl 2-ethoxy-4-methylbenzoate

Yield: 1.91 g, 42 %. RF: 0.60 (20 % ethyl acetate:80 % 40:60 petroleum ether). M.P = 68 °C

ν_{max}/cm^{-1}: 2985 (C-H), 1750 (C=O), 1600 (Ar C=C), 1571 (Ar C=C)

δ_H/ppm (400 MHz, DMSO-d$_6$): 8.09 (d, *J* = 8.7 Hz, 2H, Ar-H), 7.82 (d, *J* = 7.9 Hz, 1H, Ar-H), 7.51 – 7.33 (m, 7H, Ar-H), 7.05 (s, 1H, Ar-H), 6.91 (d, *J* = 7.8 Hz, 1H, Ar-H), 5.37 (s, 2H, OCH_2-Ar-H), 4.13 (q, *J* = 6.9 Hz, 2H, $O\underline{CH_2}CH_3$), 2.38 (s, 3H, Ar-CH_3), 1.34 (t, *J* = 6.9 Hz, 3H, $OCH_2\underline{CH_3}$)

δ_C/ppm (100 MHz, DMSO-d$_6$): 165.87, 163.86, 159.78, 155.11, 145.92, 136.12, 132.46, 131.33 (2C), 128.70 (2C), 128.35, 128.23 (2C), 127.45, 122.05 (2C), 121.17, 115.85, 114.19, 66.82, 64.66, 22.14, 14.87

3.2 4-[(Benzyloxy)carbonyl]phenyl 4-methyl-2-propoxybenzoate

Yield: 2.40 g, 48 %. RF: 0.80 (20 % ethyl acetate:80 % 40:60 petroleum ether). M.P = 73 °C

ν_{max}/cm^{-1}: 2964 (C-H), 1746 (C=O), 1599 (Ar C=C), 1570 (Ar C=C)

δ_H/ppm (400 MHz, $CDCl_3$): 8.09 (d, J = 8.5 Hz, 2H, Ar-H), 7.82 (d, J = 7.9 Hz, 1H, Ar-H), 7.53 – 7.32 (m, 7H, Ar-H), 7.05 (s, 1H, Ar-H), 6.90 (d, J = 7.9 Hz, 1H, Ar-H), 5.37 (s, 2H, OCH_2-Ar-H), 4.04 (t, J = 6.3 Hz, 2H, $O\underline{CH_2}CH_2CH_3$), 2.38 (s, 3H, Ar-$CH_3$), 1.74 (sext, 2H, J = 6.8 Hz, $OCH_2\underline{CH_2}CH_3$), 0.98 (t, J = 7.4 Hz, 3H, $OCH_2CH_2\underline{CH_3}$)

δ_C/ppm (100 MHz, $CDCl_3$): 165.88, 164.02, 159.88, 155.14, 145.91, 136.13, 132.55, 131.36 (2C), 128.71 (2C), 128.35, 128.23 (2C), 127.45, 122.05 (2C), 121.07, 115.80, 114.02, 70.45, 66.83, 22.71, 22.14, 10.71

3.3 4-[(Benzyloxy)carbonyl]phenyl 2-butoxy-4-methylbenzoate

Yield: 1.60 g, 36 %. RF: 0.85 (20 % ethyl acetate:80 % 40:60 petroleum ether). M.P = 50 °C

ν_{max}/cm^{-1}: 2945 (C-H), 1744 (C=O), 1602 (Ar C=C), 1570 (Ar C=C)

δ_H/ppm (400 MHz, $CDCl_3$): 8.09 (d, J = 8.5 Hz, 2H, Ar-H), 7.81 (d, J = 7.9 Hz, 1H, Ar-H), 7.53 – 7.33 (m, 7H, Ar-H), 7.06 (s, 1H, Ar-H), 6.90 (d, J = 8.0 Hz, 1H, Ar-H), 5.37 (s, 2H, OCH_2-Ar-H), 4.07 (t, J = 6.2 Hz, 2H, $O\underline{CH_2}(CH_2)_2CH_3$), 2.38 (s, 3H, Ar-$CH_3$), 1.70 (quin, J = 6.7 Hz, 2H, $OCH_2\underline{CH_2}CH_2CH_3$), 1.45 (sext, J = 7.4 Hz, 2H, $O(CH_2)_2\underline{CH_2}CH_3$), 0.88 (t, J = 7.4 Hz, 3H)

δ_C/ppm (100 MHz, $CDCl_3$): 165.88, 163.99, 159.90, 155.15, 145.90, 136.13, 132.54, 131.36 (2C), 128.71 (2C), 128.36, 128.24 (2C), 127.45, 122.04 (2C), 121.05, 115.80, 114.00, 68.62, 66.83, 31.35, 22.15, 19.30, 13.90

3.4 4-[(Benzyloxy)carbonyl]phenyl 4-methyl-2-pentoxybenzoate

Yield: 2.42 g, 56 %. RF: 0.73 (20 % ethyl acetate:80 % 40:60 petroleum ether). M.P = 67 °C

ν_{max}/cm^{-1}: 2944 (C-H), 1743 (C=O), 1603 (Ar C=C), 1570 (Ar C=C)

δ_H/ppm (400 MHz, $CDCl_3$): 8.09 (d, J = 8.7 Hz, 2H, Ar-H), 7.81 (d, J = 7.9 Hz, 1H, Ar-H), 7.51 – 7.35 (m, 7H, Ar-H), 7.05 (s, 1H, Ar-H), 6.90 (d, J = 7.9 Hz, 1H, Ar-H), 5.37 (s, 2H, OCH_2-Ar-H), 4.07 (t, J = 6.3 Hz, 2H, $O\underline{CH_2}(CH_2)_3CH_3$), 2.38 (s, 3H, Ar-$CH_3$), 1.72 (quin, J = 6.7 Hz, 2H, $OCH_2\underline{CH_2}(CH_2)_2CH_3$), 1.45 – 1.24 (m, 4H, $OCH_2CH_2(\underline{CH_2})_2CH_3$), 0.81 (t, J = 7.3 Hz, 3H, $O(CH_2)_4\underline{CH_3}$)

δ_C/ppm (100 MHz, $CDCl_3$): 165.89, 164.07, 159.86, 155.16, 145.89, 136.14, 132.55, 131.36 (2C), 128.71 (2C), 128.36, 128.25 (2C), 127.45, 122.04 (2C), 121.07, 115.86, 114.03, 68.98, 66.84, 29.01, 28.24, 22.48, 22.15, 14.09

3.5 4-[(Benzyloxy)carbonyl]phenyl 2-hexyloxy-4-methylbenzoate

Yield: 1.67 g, 33 %. RF: 0.71 (20 % ethyl acetate:80 % 40:60 petroleum ether). M.P = 74 °C

ν_{max}/cm^{-1}: 2944 (C-H), 1743 (C=O), 1603 (Ar C=C), 1570 (Ar C=C)

δ_H/ppm (400 MHz, $CDCl_3$): 8.15 (d, J = 8.6 Hz, 2H, Ar-H), 7.91 (d, J = 7.8 Hz, 1H, Ar-H), 7.49 – 7.32 (m, 5H, Ar-H), 7.29 (d, J = 8.6 Hz, 2H, Ar-H) 6.86 – 6.81 (m, 2H, Ar-H), 5.38 (s, 2H, OCH_2-Ar-H), 4.06 (t, J = 6.5 Hz, 2H, $O\underline{CH_2}(CH_2)_4CH_3$), 2.41 (s, 3H, Ar-$CH_3$), 1.84 (quin, J = 6.6 Hz, 2H, $OCH_2\underline{CH_2}(CH_2)_3CH_3$), 1.55 – 1.22 (m, 6H, $OCH_2CH_2(\underline{CH_2})_3CH_3$), 0.86 (t, J = 6.9 Hz, 3H, $O(CH_2)_5\underline{CH_3}$)

δ_C/ppm (100 MHz, $CDCl_3$): 165.91, 164.10, 159.87, 155.17, 145.90, 136.15, 132.56, 131.37 (2C), 128.73 (2C), 128.37, 128.26 (2C), 127.46, 122.05 (2C), 121.08, 115.87, 114.04, 69.00, 66.85, 31.62, 29.31, 25.79, 22.68, 22.17, 14.12

3.6 4-[(Benzyloxy)carbonyl]phenyl 2-heptyloxy-4-methylbenzoate

Yield: 3.49 g, 46 %. RF: 0.71 (20 % ethyl acetate:80 % 40:60 petroleum ether). M.P = 67 °C

ν_{max}/cm^{-1}: 2946 (C-H), 1745 (C=O), 1603 (Ar C=C), 1571 (Ar C=C)

δ_H/ppm (400 MHz, $CDCl_3$): 8.16 (d, *J* = 8.4 Hz, 2H, Ar-H), 7.91 (d, *J* = 7.8 Hz, 1H, Ar-H), 7.49 – 7.33 (m, 5H, Ar-H), 7.30 (d, *J* = 8.4 Hz, 2H, Ar-H), 6.88 – 6.81 (m, 2H, Ar-H), 5.39 (s, 2H, OCH_2-Ar-H), 4.06 (t, *J* = 6.4 Hz, 2H, $O\underline{CH_2}(CH_2)_5CH_3$), 2.42 (s, 3H, Ar-$CH_3$), 1.84 (quin, *J* = 6.6 Hz, 2H, $OCH_2\underline{CH_2}(CH_2)_4CH_3$), 1.53 – 1.20 (m, 8H, $OCH_2CH_2\underline{(CH_2)}_4CH_3$), 0.86 (t, *J* = 6.9 Hz, 3H, $O(CH_2)_6\underline{CH_3}$)

δ_C/ppm (100 MHz, $CDCl_3$): 165.89, 164.11, 159.86, 155.16, 145.89, 136.14, 132.55, 131.35 (2C), 128.71 (2C), 128.36, 128.24 (2C), 127.45, 122.04 (2C), 121.07, 115.86, 114.03, 68.98, 66.84, 31.84, 29.34, 29.11, 26.06, 22.68, 22.15, 14.17

3.7 4-[(Benzyloxy)carbonyl]phenyl 4-methyl-2-octyloxybenzoate

Yield: 0.98 g, 23 %. RF: 0.71 (20 % ethyl acetate:80 % 40:60 petroleum ether). M.P = 71 °C

ν_{max}/cm^{-1}: 2920 (C-H), 1744 (C=O), 1600 (Ar C=C), 1572 (Ar C=C)

δ_H/ppm (400 MHz, $CDCl_3$): 8.15 (d, *J* = 8.6 Hz, 2H, Ar-H), 7.91 (d, *J* = 7.8 Hz, 1H, Ar-H), 7.49 – 7.35 (m, 5H, Ar-H), 7.30 (d, *J* = 8.6 Hz, 2H, Ar-H), 6.88 – 6.81 (m, 2H, Ar-H), 5.38 (s, 2H, OCH_2-Ar-H), 4.06 (t, *J* = 6.4 Hz, 2H, $O\underline{CH_2}(CH_2)_6CH_3$), 2.41 (s, 3H, Ar-$CH_3$), 1.84 (quin, *J* = 6.7 Hz, 2H, $OCH_2\underline{CH_2}(CH_2)_5CH_3$), 1.53 – 1.17 (m, 10H, $OCH_2CH_2\underline{(CH_2)}_5CH_3$), 0.86 (t, *J* = 7.1 Hz, 3H, $O(CH_2)_7\underline{CH_3}$)

δ_C/ppm (100 MHz, $CDCl_3$): 165.90, 164.12, 159.87, 155.18, 145.89, 136.16, 132.57, 131.37 (2C), 128.73 (2C), 128.37, 128.26 (2C), 127.46, 122.05 (2C), 121.08, 115.89, 114.04, 69.01, 66.85, 31.90, 29.42, 29.36, 29.33, 26.13, 22.76, 22.17, 14.20

4-(2-Alkyloxy-4-methylbenzoyloxy)benzoic acids (4)

To a pre-dried flask flushed with argon, **Compound 3** (1 eq) was dissolved in a mixture of dichloromethane and ethanol and stirred. The mixture was sparged with argon and 5 % Pd/C catalyst (0.1 eq) was added. The argon atmosphere was evacuated under vacuum and replaced by hydrogen gas. The quantities of the reagents used in each reaction are listed in **Table SI4**. The reaction was allowed to proceed for 4 h at room temperature, with the extent of the reaction monitored by TLC using an appropriate solvent system (RF values quoted in the product data). The hydrogen gas, after the reaction was completed, was evacuated under vacuum and the flask was purged using argon. The mixture was filtered through Celite, and the collected solvent was evaporated under vacuum to leave a white solid which was carried forwards without any further purification.

Table SI4. Quantities of reagents used in the syntheses of the 4-(2-alkyloxy-4-methylbenzoyloxy)benzoic acids

m	(**3**)	5 % Palladium on Carbon	Dichloromethane	Ethanol
2	1.85 g, 4.74×10^{-3} mol	0.050 g, 4.74×10^{-4} mol	100 mL	100 mL
3	2.32 g, 5.74×10^{-3} mol	0.061 g, 5.74×10^{-4} mol	100 mL	100 mL
4	1.50 g, 3.59×10^{-3} mol	0.038 g, 3.59×10^{-4} mol	80 mL	80 mL
5	2.30 g, 5.32×10^{-3} mol	0.061 g, 5.32×10^{-4} mol	100 mL	100 mL
6	1.63 g, 3.65×10^{-3} mol	0.039 g, 3.65×10^{-4} mol	70 mL	70 mL
7	3.39 g, 7.34×10^{-3} mol	0.078 g, 7.34×10^{-4} mol	100 mL	100 mL
8	0.94 g, 1.98×10^{-3} mol	0.021 g, 1.98×10^{-4} mol	50 mL	50 mL

4.1 4-(2-Ethoxy-4-methylbenzoyloxy)benzoic acid

Yield: 1.40 g, 98 %. RF: 0.08 (20 % ethyl acetate:80 % 40:60 petroleum ether). M.P = 166 °C

ν_{max}/cm^{-1}: 2938 (C-H), 2809 (broad OH), 1746 (C=O), 1601 (Ar C=C), 1570 (Ar C=C)

δ_H/ppm (400 MHz, DMSO-d$_6$): 13.03 (s, 1H, COOH), 8.03 (d, *J* = 8.7 Hz, 2H, Ar-H), 7.81 (d, *J* = 7.9 Hz, 1H, Ar-H), 7.35 (d, *J* = 8.7 Hz, 2H, Ar-H), 7.05 (s, 1H, Ar-H), 6.90 (d, *J* = 7.8 Hz, 1H, Ar-H), 4.14 (q, *J* = 6.9 Hz, 2H, O$\underline{CH_2}$CH$_3$), 2.38 (s, 3H, Ar-CH$_3$), 1.34 (t, *J* = 6.9 Hz, 3H, OCH$_2\underline{CH_3}$)

δ_C/ppm (100 MHz, DMSO-d$_6$): 166.69, 163.44, 158.71, 154.22, 145.58, 131.64, 130.91 (2C), 128.31, 122.12 (2C), 120.96, 115.51, 114.35, 64.11, 21.50, 14.57

4.2 4-(4-Methyl-2-propoxybenzoyloxy)benzoic acid

Yield: 1.77 g, 98 %. RF: 0.08 (20 % ethyl acetate:80 % 40:60 petroleum ether). M.P = 172 °C

ν_{max}/cm^{-1}: 2966 (C-H), 2878 (broad OH), 1740 (C=O), 1604 (Ar C=C), 1571 (Ar C=C)

δ_H/ppm (400 MHz, DMSO-d$_6$): 13.01 (s, 1H, COOH), 8.02 (d, *J* = 8.4 Hz, 2H, Ar-H), 7.81 (d, *J* = 7.9 Hz, 1H, Ar-H), 7.34 (d, *J* = 8.4 Hz, 2H, Ar-H), 7.05 (s, 1H, Ar-H), 6.90 (d, *J* = 7.9 Hz, 1H, Ar-H), 4.04 (t, *J* = 6.3 Hz, 2H, O$\underline{CH_2}$CH$_2$CH$_3$), 2.38 (s, 3H, Ar-CH$_3$), 1.73 (sext, *J* = 6.8 Hz, 2H, OCH$_2\underline{CH_2}$CH$_3$), 0.98 (t, *J* = 7.3 Hz, 3H, OCH$_2$CH$_2\underline{CH_3}$)

δ_C/ppm (100 MHz, DMSO-d$_6$): 166.70, 163.54, 158.90, 154.24, 145.67, 131.75, 130.93 (2C), 128.32, 122.10 (2C), 120.89, 115.32, 114.18, 69.73, 22.08, 21.50, 10.42

4.3 4-(2-Butoxy-4-methylbenzoyloxy)benzoic acid

Yield: 1.11 g, 94 %. RF: 0.08 (20 % ethyl acetate:80 % 40:60 petroleum ether). M.P = 153 °C

ν_{max}/cm^{-1}: 2951 (C-H), 2867 (broad OH), 1720 (C=O), 1602 (Ar C=C), 1571 (Ar C=C)

δ_H/ppm (400 MHz, DMSO-d_6): 13.01 (s, 1H, COOH), 8.03 (d, *J* = 8.4 Hz, 2H, Ar-H), 7.81 (d, *J* = 7.9 Hz, 1H, Ar-H), 7.33 (d, *J* = 8.5 Hz, 2H, Ar-H), 7.06 (s, 1H, Ar-H), 6.90 (d, *J* = 7.9 Hz, 1H, Ar-H), 4.08 (t, *J* = 6.1 Hz, 2H, $O\underline{CH_2}(CH_2)_2CH_3$), 2.38 (s, 3H, Ar-$CH_3$), 1.70 (quin, *J* = 6.8 Hz, 2H, $OCH_2\underline{CH_2}CH_2CH_3$), 1.45 (sext, *J* = 7.3 Hz, 2H, $O(CH_2)_2\underline{CH_2}CH_3$), 0.89 (t, *J* = 7.4 Hz, 3H, $O(CH_2)_3\underline{CH_3}$)

δ_C/ppm (100 MHz, DMSO-d_6): 166.69, 163.50, 158.89, 154.22, 145.63, 131.69, 130.89 (2C), 128.33, 122.04 (2C), 120.86, 115.35, 114.17, 67.91, 30.71, 21.47, 18.63, 13.56

4.4 4-(4-Methyl-2-pentoxybenzoyloxy)benzoic acid

Yield: 1.79 g, 98 %. RF: 0.08 (20 % ethyl acetate:80 % 40:60 petroleum ether). M.P = 143 °C

ν_{max}/cm^{-1}: 2958 (C-H), 2856 (broad OH), 1719 (C=O), 1602 (Ar C=C), 1572 (Ar C=C)

δ_H/ppm (400 MHz, DMSO-d_6): 13.01 (s, 1H, COOH), 8.02 (d, *J* = 8.6 Hz, 2H, Ar-H), 7.80 (d, *J* = 7.9 Hz, 1H, Ar-H), 7.33 (d, *J* = 8.6 Hz, 2H, Ar-H), 7.05 (s, 1H, Ar-H), 6.90 (d, *J* = 8.1 Hz, 1H, Ar-H), 4.07 (t, *J* = 6.2 Hz, 2H, $O\underline{CH_2}(CH_2)_3CH_3$), 2.38 (s, 3H, Ar-$CH_3$), 1.71 (quin, *J* = 6.6 Hz, 2H, $OCH_2\underline{CH_2}(CH_2)_2CH_3$), 1.45 – 1.24 (m, 4H, $OCH_2CH_2\underline{(CH_2)}_2CH_3$), 0.82 (t, *J* = 7.2 Hz, 3H, $O(CH_2)_4\underline{CH_3}$)

δ_C/ppm (100 MHz, DMSO-d_6): 166.70, 163.63, 158.87, 154.26, 145.64, 131.73, 130.90 (2C), 128.32, 122.06 (2C), 120.88, 115.37, 114.16, 68.21, 28.37, 27.65, 21.81, 21.50, 13.84

4.5 4-(2-Hexyloxy-4-methylbenzoyloxy)benzoic acid

Yield: 1.28 g, 98 %. RF: 0.08 (20 % ethyl acetate:80 % 40:60 petroleum ether). M.P = 149 °C

ν_{max}/cm^{-1}: 2953 (C-H), 2854 (broad OH), 1728 (C=O), 1603 (Ar C=C), 1572 (Ar C=C)

δ_H/ppm (400 MHz, DMSO-d_6): 13.03 (s, 1H, COOH), 8.02 (d, *J* = 8.8 Hz, 2H, Ar-H), 7.80 (d, *J* = 7.9 Hz, 1H, Ar-H), 7.33 (d, *J* = 8.7 Hz, 2H, Ar-H), 7.05 (s, 1H, Ar-H), 6.90 (d, *J* = 7.9 Hz, 1H, Ar-H), 4.07 (t, *J* = 6.2 Hz, 2H, $O\underline{CH_2}(CH_2)_4CH_3$), 2.38 (s, 3H, Ar-$CH_3$), 1.71 (quin, *J* = 6.7 Hz, 2H, $OCH_2\underline{CH_2}(CH_2)_3CH_3$), 1.43 (quin, *J* = 7.2 Hz, 2H, $O(CH_2)_2\underline{CH_2}(CH_2)_2CH_3$), 1.30 – 1.16 (m, 4H, $O(CH_2)_3\underline{(CH_2)}_2CH_3$), 0.80 (t, *J* = 7.2 Hz, 3H, $O(CH_2)_5\underline{CH_3}$)

δ_C/ppm (100 MHz, DMSO-d_6): 166.71, 163.66, 158.85, 154.24, 145.62, 131.71, 130.89 (2C), 128.34, 122.04 (2C), 120.88, 115.39, 114.15, 68.22, 30.91, 28.65, 25.14, 22.04, 21.49, 13.80

4.6 4-(2-Heptyloxy-4-methylbenzoyloxy)benzoic acid

Yield: 2.70 g, 99 %. RF: 0.08 (20 % ethyl acetate:80 % 40:60 petroleum ether). M.P = 145 °C

ν_{max}/cm^{-1}: 2920 (C-H), 2853 (broad OH), 1713 (C=O), 1601 (Ar C=C), 1571 (Ar C=C)

δ_H/ppm (400 MHz, DMSO-d_6): 13.02 (s, 1H, COOH), 8.02 (d, *J* = 8.7 Hz, 2H, Ar-H), 7.79 (d, *J* = 7.9 Hz, 1H, Ar-H), 7.33 (d, *J* = 8.7 Hz, 2H, Ar-H), 7.05 (s, 1H, Ar-H), 6.90 (d, *J* = 8.0 Hz, 1H, Ar-H), 4.07 (t, *J* = 6.2 Hz, 2H, $O\underline{CH_2}(CH_2)_5CH_3$), 2.38 (s, 3H, Ar-$CH_3$), 1.71 (quin, *J* = 6.6 Hz, 2H, $OCH_2\underline{CH_2}(CH_2)_4CH_3$), 1.42 (quin, *J* = 7.2 Hz, 2H, $O(CH_2)_2\underline{CH_2}(CH_2)_3CH_3$), 1.31 – 1.11 (m, 6H, $O(CH_2)_3\underline{(CH_2)}_3CH_3$), 0.80 (t, *J* = 7.1 Hz, 3H, $O(CH_2)_6\underline{CH_3}$)

δ_C/ppm (100 MHz, DMSO-d$_6$): 166.70, 163.72, 158.84, 154.25, 145.62, 131.72, 130.88 (2C), 128.32, 122.02 (2C), 120.88, 115.40, 114.15, 68.21, 31.23, 28.72, 28.41, 25.45, 21.99, 21.49, 13.89

4.7 4-(4-Methyl-2-octyloxybenzoyloxy)benzoic acid

White solid. Yield: 0.94 g, 99 %. RF: 0.08 (20 % ethyl acetate:80 % 40:60 petroleum ether). M.P = 115 °C

ν_{max}/cm^{-1}: 2925 (C-H), 2853 (broad OH), 1725 (C=O), 1601 (Ar C=C), 1571 (Ar C=C)

δ_H/ppm (400 MHz, DMSO-d$_6$): 13.06 (s, 1H, COOH), 8.02 (d, *J* = 8.8 Hz, 2H, Ar-H), 7.79 (d, *J* = 7.9 Hz, 1H, Ar-H), 7.32 (d, *J* = 8.7 Hz, 2H, Ar-H), 7.05 (s, 1H, Ar-H), 6.90 (d, *J* = 7.8 Hz, 1H, Ar-H), 4.07 (t, *J* = 6.2 Hz, 2H, O$\underline{CH_2}$(CH$_2$)$_6$CH$_3$), 2.38 (s, 3H, Ar-CH$_3$), 1.71 (quin, *J* = 6.4 Hz, 2H, OCH$_2$$\underline{CH_2}$(CH$_2$)$_5CH_3$), 1.42 (quin, *J* = 7.2 Hz, 2H, O(CH$_2$)$_2$$\underline{CH_2}$(CH$_2$)$_4CH_3$), 1.30 – 1.12 (m, 8H, O(CH$_2$)$_3$$\underline{(CH_2)}$$_4CH_3$), 0.80 (t, *J* = 7.1 Hz, 3H, O(CH$_2$)$_7$$\underline{CH_3}$)

δ_C/ppm (100 MHz, DMSO-d$_6$): 166.80, 163.76, 158.82, 154.14, 145.59, 131.71, 130.86 (2C), 128.68, 121.94 (2C), 120.87, 115.44, 114.14, 68.21, 31.18, 28.79, 28.72, 28.69, 25.52, 22.08, 21.49, 13.88

5.1 4-[(4-Nitrophenoxy)carbonyl]phenyl 2-alkyloxy-4-methylbenzoates (A.m)

To a pre-dried flask flushed with argon and kept in an ice bath in order to maintain the temperature at 0°C, **Compound 4** (1 eq), 4-nitrophenol (0.91 eq) and *N,N'*-dicyclohexylcarbodiimide (1.18 eq) were added. The solids were solubilised with dichloromethane (30 mL) and stirred for 2 min before 4-dimethylaminopyridine (0.091 eq) was added to the flask. The quantities of the reagents used in each reaction are listed in **Table SI5**. The temperature of the reaction mixture was increased to room temperature and the reaction was allowed to proceed overnight. A white precipitate which formed was removed by vacuum filtration and the filtrate collected. The solvent was removed under vacuum and the crude product was purified using a silica gel column with an appropriate solvent system (RF values quoted in product data). The eluent fractions of interest were evaporated under vacuum to leave a white solid which was recrystallised from hot ethanol (50 mL).

Table SI5. Quantities of reagents used in the syntheses of the 4-[(4-nitrophenoxy)carbonyl]phenyl 2-alkyloxy-4-methylbenzoates

m	(**4**)	4-Nitrophenol	4-Dimethylaminopyridine	*N,N'*-Dicyclohexylcarbodiimide
2	0.30 g, 1.0×10^{-3} mol	0.13 g, 9.1×10^{-4} mol	0.011 g, 9.1×10^{-5} mol	0.24 g, 1.18×10^{-3} mol
3	0.30 g, 9.6×10^{-4} mol	0.12 g, 8.7×10^{-4} mol	0.011 g, 8.7×10^{-5} mol	0.23 g, 1.13×10^{-3} mol
4	0.30 g, 9.1×10^{-4} mol	0.12 g, 8.3×10^{-4} mol	0.010 g, 8.3×10^{-5} mol	0.22 g, 1.08×10^{-3} mol

5	0.30 g, 8.8×10^{-4} mol	0.11 g, 8.0×10^{-4} mol	0.010 g, 8.0×10^{-5} mol	0.21 g, 1.04×10^{-3} mol
6	0.30 g, 8.4×10^{-4} mol	0.11 g, 7.6×10^{-4} mol	0.009 g, 7.6×10^{-5} mol	0.20 g, 9.9×10^{-4} mol
7	0.30 g, 8.1×10^{-4} mol	0.10 g, 7.4×10^{-4} mol	0.009 g, 7.4×10^{-5} mol	0.20 g, 9.6×10^{-4} mol
8	0.30 g, 7.8×10^{-4} mol	0.99 g, 7.1×10^{-4} mol	0.009 g, 7.1×10^{-5} mol	0.19 g, 9.2×10^{-4} mol

5.1.1 4-[(4-Nitrophenoxy)carbonyl]phenyl 2-ethoxy-4-methylbenzoate (A.2)

Yield: 0.10 g, 25 %. RF: 0.44 (30 % 40:60 petroleum ether: 70 % dichloromethane).

T_{CrI} 133 °C T_{N_FN} (102 °C) T_{NI} (105 °C)

ν_{max}/cm^{-1}: 2984 (C-H), 1734 (C=O), 1592 (Ar C=C), 1573 (Ar C=C), 1521 (NO), 1343 (NO)

δ_H/ppm (400 MHz, $CDCl_3$): 8.37 (d, *J* = 8.8 Hz, 2H, Ar-H), 8.26 (d, *J* = 8.3 Hz, 2H, Ar-H), 7.85 (d, *J* = 7.7 Hz, 1H, Ar-H), 7.66 (d, *J* = 8.8 Hz, 2H, Ar-H), 7.50 (d, *J* = 8.3 Hz, 2H, Ar-H), 7.07 (s, 1H, Ar-H), 6.93 (d, *J* = 7.8 Hz, 1H, Ar-H), 4.15 (q, *J* = 6.9 Hz, 2H, $O\underline{CH_2}CH_3$), 2.40 (s, 3H, Ar-CH_3), 1.36 (t, *J* = 6.9 Hz, 3H, $OCH_2\underline{CH_3}$)

δ_C/ppm (100 MHz, $CDCl_3$): 163.76, 163.74, 159.96, 156.14, 155.84, 146.26, 145.59, 132.58, 132.09 (2C), 125.79, 125.45 (2C), 122.80 (2C), 122.59 (2C), 121.27, 115.61, 114.25, 64.74, 22.25, 14.93

MS = $[M+H]^+$: Calculated for $C_{23}H_{20}NO_7$: 422.1247. Found: 422.1240; Difference: 1.7 ppm

5.1.2 4-[(4-Nitrophenoxy)carbonyl]phenyl 4-methyl-2-propoxybenzoate (A.3)

Yield: 0.20 g, 53 %. RF: 0.43 (30 % 40:60 petroleum ether: 70 % dichloromethane).

T_{CrI} 132 °C T_{N_FI} (79 °C)

ν_{max}/cm^{-1}: 2966 (C-H), 1735 (C=O), 1592 (Ar C=C), 1569 (Ar C=C), 1510 (NO), 1350 (NO)

δ_H/ppm (400 MHz, $CDCl_3$): 8.34 (d, *J* = 9.2 Hz, 2H, Ar-H), 8.26 (d, *J* = 8.8 Hz, 2H, Ar-H), 7.94 (d, *J* = 7.8 Hz, 1H, Ar-H), 7.43 (d, *J* = 9.2 Hz, 2H, Ar-H), 7.40 (d, *J* = 8.8 Hz, 2H, Ar-H), 6.89 – 6.82 (m, 2H, Ar-H), 4.05 (t, *J* = 6.4 Hz, 2H, $O\underline{CH_2}CH_2CH_3$), 2.43 (s, 3H, Ar-$CH_3$), 1.88 (sext, 2H, *J* = 7.3 Hz, $OCH_2\underline{CH_2}CH_3$), 1.07 (t, *J* = 7.4 Hz, 3H, $OCH_2CH_2\underline{CH_3}$)

δ_C/ppm (100 MHz, $CDCl_3$): 163.89, 163.76, 160.06, 156.18, 155.85, 146.25, 145.60, 132.67, 132.12 (2C), 125.79, 125.45 (2C), 122.81 (2C), 122.59 (2C), 121.18, 115.56, 114.09, 70.53, 22.77, 22.25, 10.77

MS = $[M+H]^+$: Calculated for $C_{24}H_{22}NO_7$: 436.1388. Found: 436.1396; Difference: -1.8 ppm

5.1.3 4-[(4-Nitrophenoxy)carbonyl]phenyl 2-butoxy-4-methylbenzoate (A.4)

Yield: 0.24 g, 62 %. RF: 0.28 (30 % 40:60 petroleum ether: 70 % dichloromethane).

T_{CrI} 136 °C T_{N_FI} (58 °C)

ν_{max}/cm^{-1}: 2960 (C-H), 1735 (C=O), 1592 (Ar C=C), 1567 (Ar C=C), 1511 (NO), 1549 (NO)

δ_H/ppm (400 MHz, $CDCl_3$): 8.34 (d, *J* = 9.2 Hz, 2H, Ar-H), 8.27 (d, *J* = 8.8 Hz, 2H, Ar-H), 7.93 (d, *J* = 8.1 Hz, 1H, Ar-H), 7.43 (d, *J* = 9.2 Hz, 2H, Ar-H), 7.39 (d, *J* = 8.8 Hz, 2H, Ar-H), 6.89 – 6.81 (m, 2H, Ar-H), 4.09 (t, *J* = 6.4 Hz, 2H, $O\underline{CH_2}(CH_2)_2CH_3$), 2.43 (s, 3H, Ar-$CH_3$), 1.88 – 1.78 (m, 2H, $OCH_2\underline{CH_2}CH_2CH_3$), 1.54 (sext, *J* = 7.4 Hz, 2H, $O(CH_2)_2\underline{CH_2}CH_3$), 0.96 (t, *J* = 7.4 Hz, 3H, $O(CH_2)_3\underline{CH_3}$)

δ_C/ppm (100 MHz, $CDCl_3$): 163.85, 163.75, 160.06, 156.17, 155.84, 146.23, 145.59, 132.65, 132.11 (2C), 125.77, 125.44 (2C), 122.80 (2C), 122.57 (2C), 121.15, 115.54, 114.06, 68.69, 31.40, 22.24, 19.35, 13.95

MS = $[M+Na]^+$: Calculated for $C_{25}H_{24}NO_7$: 450.1566. Found: 450.1553; Difference: 2.9 ppm

5.1.4 4-[(4-Nitrophenoxy)carbonyl]phenyl 4-methyl-2-pentoxybenzoate (A.5)

Yield: 0.24 g, 66 %. RF: 0.30 (30 % 40:60 petroleum ether: 70 % dichloromethane).

T_{CrI} 120 °C T_{N_FI} (42 °C)

ν_{max}/cm^{-1}: 2949 (C-H), 1727 (C=O), 1601 (Ar C=C), 1567 (Ar C=C), 1521 (NO), 1350 (NO)

δ_H/ppm (400 MHz, $CDCl_3$): 8.34 (d, *J* = 9.1 Hz, 2H, Ar-H), 8.27 (d, *J* = 8.7 Hz, 2H, Ar-H), 7.93 (d, *J* = 7.8 Hz, 1H, Ar-H), 7.43 (d, *J* = 9.1 Hz, 2H, Ar-H), 7.40 (d, *J* = 8.7 Hz, 2H, Ar-H), 6.89 – 6.82 (m, 2H, Ar-H), 4.08 (t, *J* = 6.5 Hz, 2H, $O\underline{CH_2}(CH_2)_3CH_3$), 2.43 (s, 3H, Ar-$CH_3$), 1.85 (quin, *J* = 6.5 Hz, 2H, $OCH_2\underline{CH_2}(CH_2)_2CH_3$), 1.53 – 1.31 (m, 4H, $OCH_2CH_2(\underline{CH_2})_2CH_3$), 0.89 (t, *J* = 7.2 Hz, 3H, $O(CH_2)_4\underline{CH_3}$)

δ_C/ppm (100 MHz, $CDCl_3$): 163.93, 163.75, 160.02, 156.17, 155.84, 146.22, 145.59, 132.66, 132.10 (2C), 125.78, 125.44 (2C), 122.80 (2C), 122.57 (2C), 121.16, 115.57, 114.07, 69.03, 29.06, 28.29, 22.53, 22.24, 14.14

MS = $[M+H]^+$: Calculated for $C_{26}H_{26}NO_7$: 464.1707. Found: 464.1709; Difference: -0.4 ppm

5.1.5 4-[(4-Nitrophenoxy)carbonyl]phenyl 2-hexyloxy-4-methylbenzoate (A.6)

Yield: 0.19 g, 50 %. RF: 0.33 (30 % 40:60 petroleum ether: 70 % dichloromethane).

T_{CrI} 97 °C T_{N_FI} (29 °C)

ν_{max}/cm^{-1}: 2921 (C-H), 1727 (C=O), 1600 (Ar C=C), 1567 (Ar C=C), 1525 (NO), 1349 (NO)

δ_H/ppm (400 MHz, $CDCl_3$): 8.34 (d, *J* = 9.1 Hz, 2H, Ar-H), 8.26 (d, *J* = 8.7 Hz, 2H, Ar-H), 7.93 (d, *J* = 7.8 Hz, 1H, Ar-H), 7.43 (d, *J* = 9.1 Hz, 2H, Ar-H), 7.40 (d, *J* = 8.7 Hz, 2H, Ar-H), 6.88 – 6.82 (m, 2H, Ar-H), 4.08 (t, *J* = 6.5 Hz, 2H, $O\underline{CH_2}(CH_2)_6CH_3$), 2.43 (s, 3H, Ar-$CH_3$), 1.84 (quin, *J* = 6.5 Hz, 2H, $OCH_2\underline{CH_2}(CH_2)_5CH_3$), 1.49 (quin, *J* = 7.1 Hz, 2H, $O(CH_2)_2\underline{CH_2}(CH_2)_4CH_3$), 1.37 – 1.19 (m, 8H, $O(CH_2)_3(\underline{CH_2})_4CH_3$), 0.86 (t, *J* = 7.2 Hz, 3H, $O(CH_2)_7\underline{CH_3}$)

δ_C/ppm (100 MHz, $CDCl_3$): 163.93, 163.75, 160.02, 156.18, 155.84, 146.21, 145.59, 132.65, 132.09 (2C), 125.77, 125.44 (2C), 122.80 (2C), 122.57 (2C), 121.15, 115.59, 114.08, 69.04, 31.65, 29.33, 25.82, 22.71, 22.24, 14.15

MS = $[M+H]^+$: Calculated for $C_{27}H_{28}NO_7$: 478.1866. Found: 478.1866; Difference: 0.0 ppm

5.1.6 4-[(4-Nitrophenoxy)carbonyl]phenyl 2-heptyloxy-4-methylbenzoate (A.7)

Yield: 0.18 g, 51 %. RF: 0.28 (30 % 40:60 petroleum ether: 70 % dichloromethane).

T_{CrI} 60 °C T_{N_FI} (26 °C)

ν_{max}/cm^{-1}: 2925 (C-H), 1728 (C=O), 1600 (Ar C=C), 1568 (Ar C=C), 1526 (NO), 1351 (NO)

δ_H/ppm (400 MHz, $CDCl_3$): 8.33 (d, *J* = 9.2 Hz, 2H, Ar-H), 8.26 (d, *J* = 8.8 Hz, 2H, Ar-H), 7.93 (d, *J* = 7.8 Hz, 1H, Ar-H), 7.43 (d, *J* = 9.2 Hz, 2H, Ar-H), 7.40 (d, *J* = 8.8 Hz, 2H, Ar-H), 6.89 – 6.82 (m, 2H, Ar-H), 4.08 (t, *J* = 6.5 Hz, 2H, O$\underline{CH_2}$(CH_2)$_5$$CH_3$), 2.43 (s, 3H, Ar-$CH_3$), 1.85 (quin, *J* = 6.5 Hz, 2H, $OCH_2\underline{CH_2}(CH_2)_4CH_3$), 1.49 (quin, *J* = 7.2 Hz, 2H, $O(CH_2)_2\underline{CH_2}(CH_2)_3CH_3$), 1.38 – 1.21 (m, 6H, $O(CH_2)_3(\underline{CH_2})_3CH_3$), 0.86 (t, *J* = 7.2 Hz, 3H, $O(CH_2)_6\underline{CH_3}$)

δ_C/ppm (100 MHz, $CDCl_3$): 163.94, 163.74, 160.01, 156.17, 155.83, 146.20, 145.58, 132.64, 132.08 (2C), 125.76, 125.43 (2C), 122.79 (2C), 122.55 (2C), 121.15, 115.58, 114.07, 69.03, 31.89, 29.37, 29.14, 26.10, 22.72, 22.23, 14.22

MS = $[M+H]^+$: Calculated for $C_{28}H_{30}NO_7$: 492.2022. Found: 492.2022; Difference: 0.0 ppm

5.1.7 4-[(4-Nitrophenoxy)carbonyl]phenyl 4-methoxy-2-octyloxybenzoate (A.8)

Yield: 0.18 g, 50 %. RF: 0.30 (30 % 40:60 petroleum ether: 70 % dichloromethane).

T_{CrI} 81 °C T_{N_FI} (26 °C)

ν_{max}/cm^{-1}: 2951 (C-H), 1729 (C=O), 1601 (Ar C=C), 1567 (Ar C=C), 1526 (NO), 1349 (NO)

δ_H/ppm (400 MHz, $CDCl_3$): 8.34 (d, *J* = 9.1 Hz, 2H, Ar-H), 8.26 (d, *J* = 8.7 Hz, 2H, Ar-H), 7.93 (d, *J* = 7.8 Hz, 1H, Ar-H), 7.43 (d, *J* = 9.1 Hz, 2H, Ar-H), 7.40 (d, *J* = 8.7 Hz, 2H, Ar-H), 6.88 – 6.82 (m, 2H, Ar-H), 4.08 (t, *J* = 6.5 Hz, 2H, $O\underline{CH_2}(CH_2)_6CH_3$), 2.43 (s, 3H, Ar-$CH_3$), 1.84 (quin, *J* = 6.5 Hz, 2H, $OCH_2\underline{CH_2}(CH_2)_5CH_3$), 1.49 (quin, *J* = 7.1 Hz, 2H, $O(CH_2)_2\underline{CH_2}(CH_2)_4CH_3$), 1.37 – 1.19 (m, 8H, $O(CH_2)_3(\underline{CH_2})_4CH_3$), 0.86 (t, *J* = 7.2 Hz, 3H, $O(CH_2)_7\underline{CH_3}$)

δ_C/ppm (100 MHz, $CDCl_3$): 163.94, 163.73, 160.00, 156.17, 155.82, 146.20, 145.57, 132.64, 132.08 (2C), 125.75, 125.43 (2C), 122.78 (2C), 122.55 (2C), 121.15, 115.58, 114.07, 69.03, 31.93, 29.45, 29.38, 29.36, 26.16, 22.79, 22.24, 14.24

MS = $[M+H]^+$: Calculated for $C_{29}H_{32}NO_7$: 506.2184. Found: 506.2179; Difference: 1.0 ppm

5.2 4-[(3-Fluoro-4-nitrophenoxy)carbonyl]phenyl 2-alkyloxy-4-methylbenzoates (B.m)

To a pre-dried flask flushed with argon **Compound 4** (1 eq), 3-fluoro-4-nitrophenol (0.91 eq) and *N,N'*-dicyclohexylcarbodiimide (1.18 eq) were added. The solids were solubilised with dichloromethane (30 mL) and stirred for 2 min before 4-dimethylaminopyridine (0.091 eq) was added to the flask. The quantities of the reagents used in each reaction are listed in **Table SI6**. The temperature of the reaction mixture was increased to room temperature and the reaction was allowed to proceed overnight. A white precipitate which formed was removed by vacuum filtration and the filtrate collected. The solvent was removed under vacuum and the crude product was purified using a silica gel column with an appropriate solvent system (RF values quoted in product data). The eluent fractions of interest were evaporated under vacuum to leave a white solid which was recrystallised from hot ethanol (50 mL).

Table SI6. Quantities of reagents used in the syntheses of the 4-[(3-fluoro-4-nitrophenoxy)carbonyl]phenyl 2-alkyloxy-4-methylbenzoates

m	(**4**)	3-Fluoro-4-nitrophenol	4-Dimethylaminopyridine	*N,N'*-Dicyclohexylcarbodiimide
2	0.30 g, 1.0×10^{-3} mol	0.14 g, 9.1×10^{-4} mol	0.011 g, 9.1×10^{-5} mol	0.24 g, 1.18×10^{-3} mol
3	0.30 g, 9.6×10^{-4} mol	0.14 g, 8.7×10^{-4} mol	0.011 g, 8.7×10^{-5} mol	0.23 g, 1.13×10^{-3} mol
4	0.30 g, 9.1×10^{-4} mol	0.13 g, 8.3×10^{-4} mol	0.010 g, 8.3×10^{-5} mol	0.22 g, 1.08×10^{-3} mol
5	0.30 g, 8.8×10^{-4} mol	0.13 g, 8.0×10^{-4} mol	0.010 g, 8.0×10^{-5} mol	0.21 g, 1.04×10^{-3} mol
6	0.30 g, 8.4×10^{-4} mol	0.12 g, 7.6×10^{-4} mol	0.009 g, 7.6×10^{-5} mol	0.20 g, 9.9×10^{-4} mol
7	0.30 g, 8.1×10^{-4} mol	0.12 g, 7.4×10^{-4} mol	0.009 g, 7.4×10^{-5} mol	0.20 g, 9.6×10^{-4} mol
8	0.30 g, 7.8×10^{-4} mol	0.11 g, 7.1×10^{-4} mol	0.009 g, 7.1×10^{-5} mol	0.19 g, 9.2×10^{-4} mol

5.2.1 4-[(3-Fluoro-4-nitrophenoxy)carbonyl]phenyl 2-ethoxy-4-methylbenzoate (A.2)

Yield: 0.10 g, 25 %. RF: 0.44 (30 % 40:60 petroleum ether: 70 % dichloromethane).

T_{CrI} 143 °C T_{N_FI} (108 °C)

ν_{max}/cm^{-1}: 2985 (C-H), 1737 (C=O), 1600 (Ar C=C), 1572 (Ar C=C), 1528 (NO), 1334 (NO)

δ_H/ppm (400 MHz, $CDCl_3$): 8.32 (app t, *J* = 8.8 Hz, 1H, Ar-H), 8.25 (d, *J* = 8.7 Hz, 2H, Ar-H), 7.85 (d, *J* = 7.9 Hz, 1H, Ar-H), 7.79 (dd, *J* = 12.0, 2.4 Hz, 1H, Ar-H), 7.54 – 7.46 (m, 3H, Ar-H), 7.07 (s, 1H, Ar-H), 6.92 (d, *J* = 7.9 Hz, 1H, Ar-H), 4.15 (q, *J* = 6.9 Hz, 2H, O$\underline{CH_2}$CH$_3$), 2.39 (s, 3H, Ar-CH), 1.35 (t, *J* = 6.9 Hz, 3H, OCH$_2\underline{CH_3}$)

δ_C/ppm (100 MHz, $CDCl_3$): 163.69, 163.28, 159.98, 157.71, 156.34, 155.97, 155.86, 155.05, 146.32, 135.05, 134.98, 132.59, 132.16 (2C), 127.40, 127.38, 125.35, 122.68 (2C), 121.28, 118.27, 118.23, 115.54, 114.25, 112.68, 112.44, 64.74, 22.26, 14.93

MS = $[M+H]^+$: Calculated for $C_{23}H_{19}NO_7F$: 440.1153. Found: 440.1146; Difference: 1.6 ppm

5.2.2 4-[(3-Fluoro-4-nitrophenoxy)carbonyl]phenyl 4-methyl-2-propoxybenzoate (A.3)

Yield: 0.20 g, 53 %. RF: 0.43 (30 % 40:60 petroleum ether: 70 % dichloromethane).

T_{CrI} 110 °C T_{N_FI} (87 °C)

ν_{max}/cm^{-1}: 2973 (C-H), 1745 (C=O), 1599 (Ar C=C), 1570 (Ar C=C), 1526 (NO), 1334 (NO)

δ_H/ppm (400 MHz, $CDCl_3$): 8.25 (d, *J* = 8.8 Hz, 2H, Ar-H), 8.20 (app t, *J* = 8.7 Hz, 1H, Ar-H), 7.93 (d, *J* = 7.8 Hz, 1H, Ar-H), 7.40 (d, *J* = 8.8 Hz, 2H, Ar-H), 7.30 (dd, *J* = 11.2, 2.4 Hz, 1H, Ar-H), 7.23 (ddd, *J* = 9.1, 2.4, 1.3 Hz, 1H, Ar-H), 6.91 – 6.81 (m, 2H, Ar-H), 4.05 (t, *J* = 6.4 Hz, 2H, $O\underline{CH_2}CH_2CH_3$), 2.43 (s, 3H, Ar-$CH_3$), 1.88 (sext, 2H, *J* = 7.3 Hz, $OCH_2\underline{CH_2}CH_3$), 1.07 (t, *J* = 7.4 Hz, 3H, $OCH_2CH_2\underline{CH_3}$)

δ_C/ppm (100 MHz, $CDCl_3$): 163.69, 163.14, 159.94, 157.57, 156.23, 155.83, 155.73, 154.92, 146.16, 134.91, 134.84, 132.53, 132.05 (2C), 127.26, 127.24, 125.20, 122.53 (2C), 121.04, 118.13, 118.09, 115.35, 113.95, 112.54, 112.30, 70.39, 22.63, 22.11, 10.63

MS = $[M+H]^+$: Calculated for $C_{24}H_{21}NO_7F$: 454.1305. Found: 454.1302; Difference: 0.7 ppm

5.2.3 4-[(3-Fluoro-4-nitrophenoxy)carbonyl]phenyl 2-butoxy-4-methylbenzoate (A.4)

Yield: 0.19 g, 49 %. RF: 0.33 (30 % 40:60 petroleum ether: 70 % dichloromethane).

T_{CrI} 101 °C T_{N_FI} (69 °C)

ν_{max}/cm^{-1}: 2960 (C-H), 1744 (C=O), 1601 (Ar C=C), 1567 (Ar C=C), 1531 (NO), 1346 (NO)

δ_H/ppm (400 MHz, $CDCl_3$): 8.25 (d, *J* = 8.8 Hz, 2H, Ar-H), 8.20 (app t, *J* = 8.7 Hz, 1H, Ar-H), 7.93 (d, *J* = 7.9 Hz, 1H, Ar-H), 7.40 (d, *J* = 8.8 Hz, 2H, Ar-H), 7.30 (dd, *J* = 11.2, 2.4 Hz, 1H, Ar-H), 7.23 (ddd, *J* = 9.0, 2.4, 1.3 Hz, 1H, Ar-H), 6.89 – 6.82 (m, 2H, Ar-H), 4.09 (t, *J* = 6.4 Hz, 2H, $O\underline{CH_2}(CH_2)_2CH_3$), 2.43 (s, 3H, Ar-$CH_3$), 1.83 (quin, *J* = 6.7 Hz, 2H, $CH_2\underline{CH_2}CH_2CH_3$), 1.53 (sext, *J* = 7.3 Hz, 2H, $O(CH_2)_2\underline{CH_2}CH_3$), 0.96 (t, *J* = 7.4 Hz, 3H, $O(CH_2)_3\underline{CH_3}$)

δ_C/ppm (100 MHz, $CDCl_3$): 163.80, 163.28, 160.08, 157.70, 156.36, 155.96, 155.86, 155.05, 146.29, 135.04, 134.97, 132.66, 132.17 (2C), 127.40, 127.37, 125.33, 122.66 (2C), 121.15, 118.26, 118.22, 115.47, 114.06, 112.67, 112.43, 68.69, 31.40, 22.24, 19.35, 13.95

MS = $[M+Na]^+$: Calculated for $C_{25}H_{23}NO_7F$: 468.1473. Found: 468.1459; Difference: 3.0 ppm

5.2.4 4-[(3-Fluoro-4-nitrophenoxy)carbonyl]phenyl 4-methyl-2-pentoxybenzoate (A.5)

Yield: 0.23 g, 58 %. RF: 0.38 (30 % 40:60 petroleum ether: 70 % dichloromethane).

T_{CrI} 117 °C T_{N_FI} (52 °C)

ν_{max}/cm^{-1}: 2951 (C-H), 1730 (C=O), 1599 (Ar C=C), 1567 (Ar C=C), 1528 (NO), 1347 (NO)

δ_H/ppm (400 MHz, $CDCl_3$): 8.25 (d, *J* = 8.8 Hz, 2H, Ar-H), 8.20 (app t, *J* = 8.7 Hz, 1H, Ar-H), 7.93 (d, *J* = 7.8 Hz, 1H, Ar-H), 7.40 (d, *J* = 8.8 Hz, 2H, Ar-H), 7.30 (dd, *J* = 11.3, 2.4 Hz, 1H, Ar-H), 7.23 (ddd, *J* = 9.0, 2.4, 1.3 Hz, 1H, Ar-H), 6.88 – 6.82 (m, 2H, Ar-H), 4.08 (t, *J* = 6.5 Hz, 2H, $O\underline{CH_2}(CH_2)_3CH_3$), 2.43 (s, 3H, Ar-$CH_3$), 1.85 (quin, *J* = 6.7 Hz, 2H, $OCH_2\underline{CH_2}(CH_2)_2CH_3$), 1.52 – 1.31 (m, 4H, $OCH_2CH_2(\underline{CH_2})_2CH_3$), 0.89 (t, *J* = 7.2 Hz, 3H, $O(CH_2)_4\underline{CH_3}$)

δ_C/ppm (100 MHz, $CDCl_3$): 163.88, 163.29, 160.05, 157.71, 156.38, 155.97, 155.87, 155.06, 146.28, 135.04, 134.97, 132.68, 132.18 (2C), 127.41, 127.39, 125.34, 122.67 (2C), 121.18, 118.28, 118.24, 115.51, 114.08, 112.68, 112.45, 69.03, 29.07, 28.30, 22.54, 22.25, 14.16

MS = $[M+H]^+$: Calculated for $C_{26}H_{25}NO_7F$: 482.1628. Found: 482.1615; Difference: 2.7 ppm

5.2.5 4-[(3-Fluoro-4-nitrophenoxy)carbonyl]phenyl 2-hexyloxy-4-methylbenzoate (A.6)

Yield: 0.17 g, 45 %. RF: 0.43 (30 % 40:60 petroleum ether: 70 % dichloromethane).

T_{CrI} 78 °C T_{N_FI} (43 °C)

ν_{max}/cm^{-1}: 2921 (C-H), 1730 (C=O), 1597 (Ar C=C), 1567 (Ar C=C), 1529 (NO), 1346 (NO)

δ_H/ppm (400 MHz, $CDCl_3$): 8.24 (d, *J* = 8.7 Hz, 2H, Ar-H), 8.20 (app t, *J* = 8.7 Hz, 1H), 7.93 (d, *J* = 7.8 Hz, 1H, Ar-H), 7.40 (d, *J* = 8.7 Hz, 2H, Ar-H), 7.30 (dd, *J* = 11.2, 2.4 Hz, 1H, Ar-H), 7.23 (ddd, *J* = 9.1, 2.4, 1.3 Hz, 1H, Ar-H), 6.88 – 6.82 (m, 2H, Ar-H), 4.08 (t, *J* = 6.5 Hz, 2H, $O\underline{CH_2}(CH_2)_4CH_3$), 2.43 (s, 3H, Ar-$CH_3$), 1.84 (quin, *J* = 6.7 Hz, 2H, $OCH_2\underline{CH_2}(CH_2)_3CH_3$), 1.50 (quin, *J* = 7.2 Hz, 2H, $O(CH_2)_2\underline{CH_2}(CH_2)_2CH_3$), 1.37 – 1.24 (m, 4H, $O(CH_2)_3(\underline{CH_2})_2CH_3$), 0.87 (t, *J* = 7.2 Hz, 3H, $O(CH_2)_5\underline{CH_3}$)

δ_C/ppm (100 MHz, $CDCl_3$): 163.88, 163.27, 160.03, 157.69, 156.37, 155.96, 155.86, 155.04, 146.26, 135.03, 134.97, 132.66, 132.15 (2C), 127.39, 127.37, 125.32, 122.65 (2C), 121.16, 118.26, 118.22, 115.51, 114.07, 112.67, 112.43, 69.03, 31.65, 29.33, 25.82, 22.71, 22.24, 14.15

MS = $[M+H]^+$: Calculated for $C_{27}H_{27}NO_7F$: 496.1785. Found: 496.1772; Difference: 2.6 ppm

5.2.6 4-[(3-Fluoro-4-nitrophenoxy)carbonyl]phenyl 2-heptyloxy-4-methylbenzoate (A.7)

Yield: 0.08 g, 21 %. RF: 0.35 (30 % 40:60 petroleum ether: 70 % dichloromethane).

T_{CrI} 63 °C T_{N_FI} (40 °C)

ν_{max}/cm^{-1}: 2924 (C-H), 1729 (C=O), 1602 (Ar C=C), 1570 (Ar C=C), 1529 (NO), 1347 (NO)

δ_H/ppm (400 MHz, $CDCl_3$): 8.24 (d, *J* = 8.8 Hz, 2H, Ar-H), 8.20 (app t, *J* = 8.6 Hz, 1H, Ar-H), 7.92 (d, *J* = 7.8 Hz, 1H, Ar-H), 7.40 (d, *J* = 8.8 Hz, 2H, Ar-H), 7.30 (dd, *J* = 11.2, 2.4 Hz, 1H, Ar-H), 7.23 (ddd, *J* = 9.1, 2.4, 1.3 Hz, 1H, Ar-H), 6.89 – 6.82 (m, 2H, Ar-H), 4.07 (t, *J* = 6.5 Hz, 2H, $O\underline{CH_2}(CH_2)_5CH_3$), 2.43 (s, 3H, Ar-$CH_3$), 1.84 (quin, *J* = 6.5 Hz, 2H, $OCH_2\underline{CH_2}(CH_2)_4CH_3$), 1.49 (quin, *J* = 7.1 Hz, 2H, $O(CH_2)_2\underline{CH_2}(CH_2)_3CH_3$), 1.37 – 1.21 (m, 6H, $O(CH_2)_3(\underline{CH_2})_3CH_3$), 0.86 (t, *J* = 7.2 Hz, 3H, $O(CH_2)_6\underline{CH_3}$)

δ_C/ppm (100 MHz, $CDCl_3$): 163.89, 163.27, 160.03, 157.69, 156.37, 155.96, 155.86, 155.04, 146.26, 135.03, 134.96, 132.65, 132.15 (2C), 127.39, 127.37, 125.31, 122.64 (2C), 121.16, 118.26, 118.22, 115.52, 114.08, 112.66, 112.43, 69.03, 31.89, 29.37, 29.14, 26.10, 22.73, 22.24, 14.22

MS = $[M+H]^+$: Calculated for $C_{28}H_{29}NO_7F$: 510.1940. Found: 510.1928; Difference: 2.4 ppm

5.2.7 4-[(3-Fluoro-4-nitrophenoxy)carbonyl]phenyl 4-methoxy-2-octyloxybenzoate (A.8)

Yield: 0.06 g, 16 %. RF: 0.38 (30 % 40:60 petroleum ether: 70 % dichloromethane).

T_{CrI} 68 °C T_{N_FI} (37 °C)

ν_{max}/cm^{-1}: 2923 (C-H), 1732 (C=O), 1601 (Ar C=C), 1570 (Ar C=C), 1527 (NO), 1346 (NO)

δ_H/ppm (400 MHz, $CDCl_3$): 8.24 (d, *J* = 8.7 Hz, 2H, Ar-H), 8.20 (app t, *J* = 8.5 Hz, 1H, Ar-H), 7.92 (d, *J* = 7.8 Hz, 1H, Ar-H), 7.40 (d, *J* = 8.7 Hz, 2H, Ar-H), 7.30 (dd, *J* = 11.3, 2.4 Hz, 1H, Ar-H), 7.23 (ddd, *J* = 9.1, 2.4, 1.3 Hz, 1H, Ar-H), 6.88 – 6.82 (m, 2H, Ar-H), 4.07 (t, *J* = 6.5 Hz, 2H, $O\underline{CH_2}(CH_2)_6CH_3$), 2.43 (s, 3H, Ar-$CH_3$), 1.84 (quin, *J* = 6.5 Hz, 2H, $OCH_2\underline{CH_2}(CH_2)_5CH_3$), 1.49 (quin, *J* = 7.2 Hz, 2H, $O(CH_2)_2\underline{CH_2}(CH_2)_4CH_3$), 1.37 – 1.19 (m, 8H, $O(CH_2)_3(\underline{CH_2})_4CH_3$), 0.86 (t, *J* = 7.2 Hz, 3H, $O(CH_2)_7\underline{CH_3}$)

δ_C/ppm (100 MHz, $CDCl_3$): 63.90, 163.26, 160.03, 157.70, 156.37, 155.96, 155.86, 155.04, 146.26, 135.04, 134.97, 132.66, 132.16 (2C), 127.39, 127.37, 125.31, 122.65 (2C), 121.16, 118.25, 118.22, 115.52, 114.08, 112.66, 112.42, 69.04, 31.93, 29.45, 29.38, 29.36, 26.15, 22.79, 22.24, 14.23

MS = $[M+H]^+$: Calculated for $C_{29}H_{31}NO_7F$: 524.2090. Found: 524.2085; Difference: 1.0 ppm

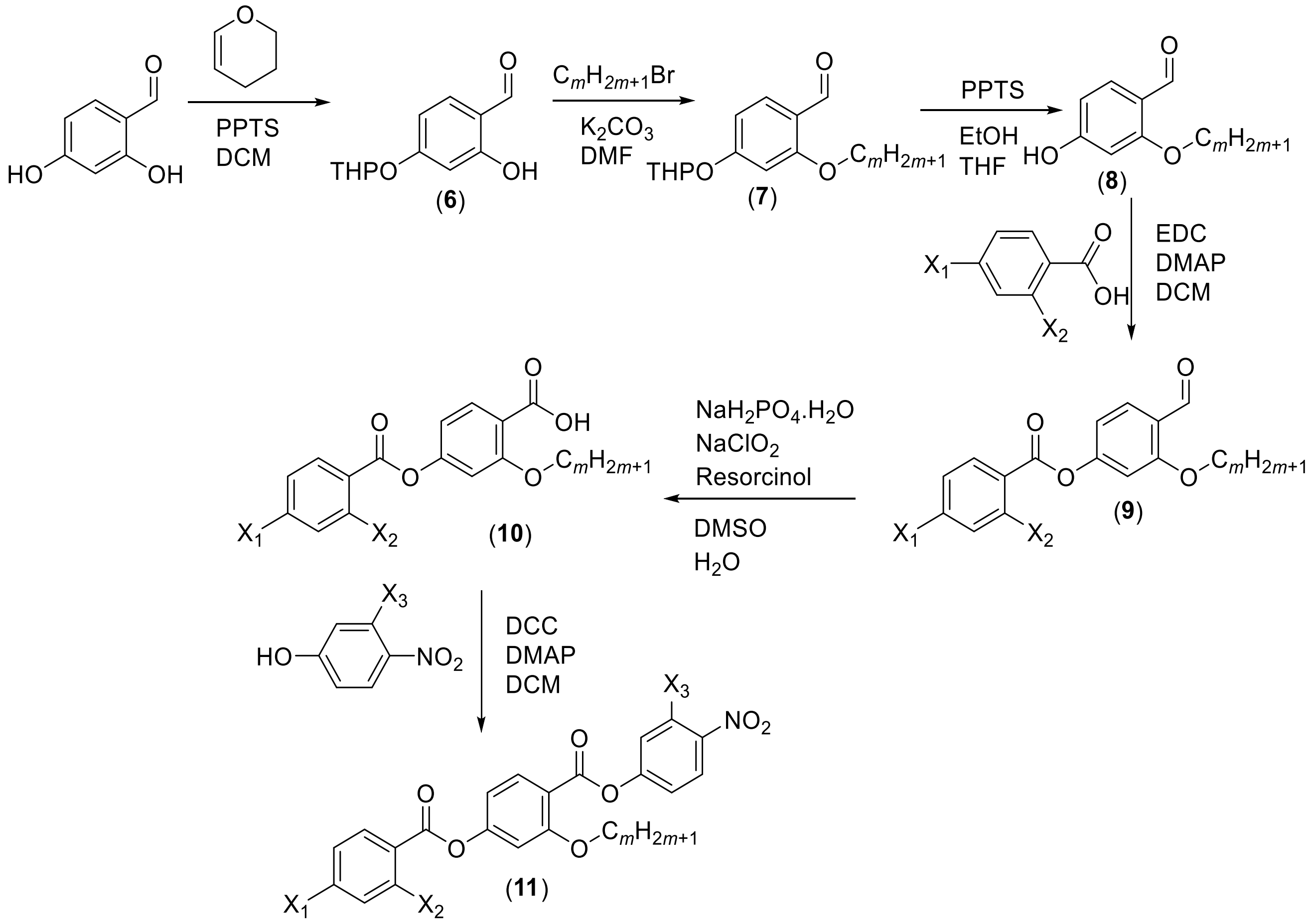

C.*m* => X_1 = Me, X_2 = H, X_3 = H
D.*m* => X_1 = Me, X_2 = H, X_3 = F
G.*m* => X_1 = Me, X_2 = OMe, X_3 = H
H.*m* => X_1 = Me, X_2 = OMe, X_3 = F
K.*m* => X_1 = OMe, X_2 = OMe, X_3 = H
L.*m* => X_1 = OMe, X_2 = OMe, X_3 = F

Figure SI2. Synthetic scheme for the C.*m*, D.*m*, G.*m*, H.*m*, K.*m* and L.*m* series. The compounds were synthesised using a six-step reaction with the final step being a Steglich esterification.

(6) 2-Hydroxy-4-(oxan-2-yloxy)benzaldehyde

Under inert conditions, to a solution of 2,4-dihydroxybenzaldehyde (1 eq, 10.000 g, 0.0724 mol) and 3,4-dihydropyran (1.2 eq, 7.31 g, 7.90 mL, 0.0868 mol) in 30 ml dichloromethane, pyridinium p-toluenesulfonate (0.1 eq, 1.802 g, 7.16×10^{-2} mol) was added dissolved in dichloromethane (5 mL). The reaction mixture was allowed to react for 2 h and then quenched by the addition of saturated $NaHCO_3$ (aq). The mixture was extracted with dichloromethane (3 × 50 mL) and then dried with $MgSO_4$. The drying agent was removed by filtration, and the solvent was removed on the rotary evaporator. The product was purified by flash column chromatography using a mixture of ethyl acetate and hexane (5:95) to obtain the pure compound as a colourless oil (10.459 g) with a yield of 65%.
Colourless oil. Yield: 10.459 g, 65% RF: 0.38 (10% ethyl acetate: 90% hexane).

ν_{max}/cm^{-1}: 3228 (OH), 2945 (-CH_2), 2854 (-CH), 1627 (RC(=O)H), 1506 (Ar C=C).

δ_H/ppm (400 MHz, DMSO-d_6): 11.35 (s, 1H, -HCO), 9.71 (s, 1H, OH), 7.42 (d, *J* = 8.6 Hz, 1H, Ar-H), 6.64 (dd, *J* = 8.6, 2.2 Hz, 1H, Ar-H), 6.61 (d, *J* = 2.2 Hz, 1H, Ar-H), 5.49 (t, *J* = 3.2 Hz,

1H, THP), 3.81 (ddd, J = 11.4, 9.9, 3.2 Hz, 1H, THP), 3.62 (dtd, J = 11.4, 4.1, 1.4 Hz, 1H, THP), 1.98 – 1.41 (m, 6H, THP).

δ_C/ppm (100 MHz, DMSO-d$_6$): 194.69, 164.46, 164.29, 135.41, 115.87, 109.52, 103.79, 96.35, 62.31, 30.07, 25.08, 18.55.

Data consistent with reported literature.[1]

(7) 2-Alkyloxy-4-(oxan-2-yloxy)benzaldehydes

A two-neck round bottom flask was charged with **Compound 6** (1 eq) and potassium carbonate (1.2 eq) under inert conditions. The quantities of reagents used in the reaction are listed in **Table SI7**. The solids were suspended in dimethylformamide and the required 1-bromoalkane chain was injected. The reaction was allowed to proceed overnight at 60˚C. After cooling, the reaction mixture was diluted with water (100 mL), extracted with ethyl acetate (3 × 50 mL) and dried with $MgSO_4$. The drying agent was removed by filtration and the solvent was removed on the rotary evaporator to obtain the pure compound without further purification.

Table SI7. Quantities of reagents used to synthesise 2-alkyloxy-4-(oxan-2-yloxy)benzaldehydes.

m	(**6**)	1-Bromoalkane	Potassium Carbonate
2	4.000 g, 0.0179 mol	2.281 g, 1.56 ml, 0.0216 mol	2.971 g, 0.0216 mol
3	3.000 g, 0.0134 mol	1.982 g, 1.46 ml, 0.0161 mol	2.230 g, 0.0161 mol
4	8.841 g, 0.0397 mol	6.540 g, 5.15ml, 0.0477 mol	6.601 g, 0.0477 mol
5	11.000 g, 0.0490 mol	8.881 g, 7.29 ml, 0.058 mol	8.171 g, 0.0590 mol
6	10.000 g, 0.0447 mol	6.602 g, 5.62 ml, 0.0537 mol	8.874 g, 0.0537 mol
7	6.000 g, 0.0269 mol	5.774 g, 5.06 ml, 0.0323 mol	5.323 g, 0.0323 mol

7.1 2-Ethoxy-4-(oxan-2-yloxy)benzaldehyde

Colourless oil. Yield: 4.271 g, 95%. RF: 0.45 (20% ethyl acetate:80% hexane)

v_{max}/cm^{-1}: 2941 (-CH_2), 2852 (-CH), 1674 (RC(=O)H), 1596 (Ar C=C), 1034 (C-O-C).

δ_H/ppm (400 MHz, DMSO-d_6): 10.21 (s, 1H, -HCO), 7.65 (d, *J* = 8.6 Hz, 1H, Ar-H), 6.75 (d, *J* = 2.2 Hz, 1H, Ar-H), 6.72 (dd, *J* = 8.6, 2.2, 1H, Ar-H), 5.65 (t, *J* = 3.0 Hz, 1H, THP), 4.16 (q, *J* = 7.0 Hz, 2H, $O\underline{CH_2}CH_3$), 3.80 – 3.67 (m, 1H, THP), 3.64 – 3.54 (m, 1H, THP), 1.94 – 1.47 (m, 6H, THP), 1.38 (t, *J* = 7.0 Hz, 3H, $OCH_2\underline{CH_3}$).

δ_C/ppm (100 MHz, DMSO-d_6): 187.89, 163.72, 163.09, 129.89, 119.19, 108.95, 101.48, 96.05, 64.62, 62.10, 29.95, 25.00, 18.75, 14.82.

Data consistent with reported literature.[1]

7.2 2-Propoxy-4-(oxan-2-yloxy)benzaldehyde (7.3)

Colourless oil. Yield: 3.762 g, 95%. RF: 0.45 (20% ethyl acetate:80% hexane)

v_{max}/cm^{-1}: 2978 (-CH_3), 2942 (-CH_2), 2852 (-CH), 1676 (RC(=O)H), 1597 (Ar C=C), 1035 (C-O-C).

δ_H/ppm (400 MHz, DMSO-d_6): 10.23 (s, 1H, -HCO), 7.65 (d, *J* = 8.6 Hz, 1H, Ar-H), 6.76 (d, *J* = 2.1 Hz, 1H, Ar-H), 6.71 (dd, *J* = 8.6, 2.1 Hz, 1H, Ar-H), 5.66 (t, *J* = 3.0 Hz, 1H, THP), 4.12 – 4.00 (m, 4H, $O\underline{CH_2CH_2}CH_3$) 3.80 – 3.67 (m, 1H, THP), 3.64 – 3.54 (m, 1H, THP), 1.94 - 1.70 (m, 4H, THP), 1.68 – 1.44 (m, 2H, THP), 1.00 (t, *J* = 7.4 Hz, 3H, $OCH_2CH_2\underline{CH_3}$).

δ_C/ppm (100 MHz, DMSO-d_6): 188.36, 163.70, 163.27, 129.85, 119.44, 108.41, 100.32, 96.10, 69.94, 61.97, 30.03, 25.02, 22.40, 18.36, 10.54.

Data consistent with reported literature.[1]

7.3 2-Butoxy-4-(oxan-2-yloxy)benzaldehyde (7.4)

Yellow oil. Yield: 11.001 g, 100%. RF: 0.52 (20% ethyl acetate:80% hexane)

v_{max}/cm^{-1}: 2939 (-CH_2), 2873 (-CH), 1738, 1676 (RC(=O)H), 1597 (Ar C=C), 1036 (C-O-C).

δ_H/ppm (400 MHz, DMSO-d_6): 10.22 (s, 1H, -HCO), 7.65 (d, *J* = 8.6 Hz, 1H, Ar-H), 6.79 – 6.68 (m, 2H, Ar-H), 5.66 (t, *J* = 3.1 Hz, 1H, THP), 4.15 – 4.05 (m, 2H, $O\underline{CH_2}CH_2CH_2CH_3$), 3.73 (m, 1H, THP), 3.70 – 3.80 (m, 1H, THP), 1.92 – 1.69 (m, 4H, THP), 1.691.49 (m, 2H, THP), 1.51 – 1.41 (m, 4H, $OCH_2\underline{CH_2CH_2}CH_3$), 0.95 (t, *J* = 7.4 Hz, 3H, OCH_2 $CH_2CH_2\underline{CH_3}$).

δ_C/ppm (100 MHz, DMSO-d_6): 187.32, 163.27, 162.79, 129.41, 118.78, 108.49, 100.98, 95.55, 68.05, 61.60, 59.75, 30.50, 29.48, 24.54, 18.73, 13.66.

7.4 2-Pentoxy-4-(oxan-2-yloxy)benzaldehyde

Orange oil. Yield: 14.440 g, 100%. RF: 0.52 (20% ethyl acetate:80% hexane)

ν_{max}/cm^{-1}: 2936 (-CH_2), 2871 (-CH), 1677 (RC(=O)H), 1596 (Ar C=C), 1036 (C-O-C).

δ_H/ppm (400 MHz, DMSO-d_6): 10.21 (s, 1H, -HCO), 7.64 (d, *J* = 8.6 Hz, 1H, Ar-H), 6.75 (d, *J* = 2.1 Hz, 1H, Ar-H), 6.71 (dd, *J* = 8.6, 2.1 Hz, 1H, Ar-H), 5.65 (t, *J* = 3.1 Hz, 1H, THP), 4.09 (t, *J* = 6.4 Hz, 2H, $O\underline{CH_2}CH_2CH_2CH_2CH_3$), 3.71-3.79 (m, 1H, THP), 3.63 – 3.47 (m, 1H, THP), 1.95-1.68 (m, 6H, THP, $OCH_2\underline{CH_2}CH_2CH_2CH_3$), 1.68 – 1.47 (m, 2H, THP), 1.48 – 1.27 (m, 4H, $OCH_2CH_2\underline{CH_2CH_2}CH_3$), 0.89 (t, *J* = 7.1 Hz, 3H, $OCH_2CH_2CH_2CH_2\underline{CH_3}$).

δ_C/ppm (100 MHz, DMSO-d_6): 187.38, 163.29, 162.82, 129.45, 118.79, 108.53, 101.01, 95.58, 68.37, 61.64, 29.50, 28.12, 27.69, 24.56, 21.86, 18.28, 13.92.

7.5 2-Hexyloxy-4-(oxan-2-yloxy)benzaldehyde

Brown oil. Yield: 12.420 g, 90%. RF: 0.56 (20% ethyl acetate:80% hexane)

ν_{max}/cm^{-1}: 2932 (-CH_2), 2855 (-CH), 1676 (RC(=O)H), 1598 (Ar C=C), 1036 (C-O-C).

δ_H/ppm (400 MHz, DMSO-d_6): 10.21 (s, 1H, -HCO), 7.64 (d, *J* = 8.5 Hz, 1H, Ar-H), 6.75 (d, *J* = 2.1 Hz, 1H, Ar-H), 6.74 – 6.68 (d, *J* = 8.5, 2.1 Hz, 1H, Ar-H), 5.65 (q, *J* = 3.0 Hz, 1H, THP), 4.15 – 4.15 (m, 2H, $O\underline{CH_2}CH_2CH_2CH_2CH_2CH_3$), 3.72 (m, 1H, THP), 3.64 – 3.54 (m, 1H, THP), 1.92 – 1.68 (m, 6H, THP, $OCH_2\underline{CH_2}CH_2CH_2CH_2CH_3$), 1.70 – 1.49 (m, 2H, THP), 1.43 (p, *J* = 7.5 Hz, 2H, $OCH_2CH_2\underline{CH_2}CH_2CH_2CH_3$), 1.38 – 1.26 (m, 4H, $OCH_2CH_2CH_2\underline{CH_2CH_2}CH_3$), 0.87 (t, *J* = 6.6 Hz, 3H, $OCH_2CH_2CH_2CH_2CH_2\underline{CH_3}$).

δ_C/ppm (100 MHz, DMSO-d_6): 187.31, 163.26, 162.78, 129.40, 118.76, 108.49, 100.99, 95.54, 68.34, 61.60, 30.91, 29.47, 28.35, 25.11, 24.51, 22.02, 18.25, 13.86.

7.6 2-Heptyloxy-4-(oxan-2-yloxy)benzaldehyde

Brown oil. Yield: 8.614 g, 100%. RF: 0.59 (20% ethyl acetate:80% hexane)

ν_{max}/cm^{-1}: 2928 (-CH_2), 2854 (-CH), 1738, 1676 (RC(=O)H), 1598 (Ar C=C), 1035 (C-O-C).

δ_H/ppm (400 MHz, DMSO-d_6): 10.21 (s, 1H, -HCO), 7.64 (d, *J* = 8.6 Hz, 1H, Ar-H), 6.76 (d, *J* = 2.2 Hz, 1H, Ar-H), 6.71 (dd, *J* = 8.6, 2.2 Hz, 1H, Ar-H), 5.66 (t, *J* = 3.1 Hz, 1H, THP), 4.20 – 4.06 (m, 2H, $O\underline{CH_2}CH_2CH_2CH_2CH_2CH_2CH_3$), 3.77 – 3.67 (m, 1H, THP), 3.64 – 3.55 (m, 1H, THP), 1.96 – 1.70 (m, 4H, THP), 1.66 – 1.49 (m, 2H, THP), 1.48 – 1.23 (m, 10H, $OCH_2\underline{CH_2CH_2CH_2CH_2CH_2}CH_3$), 0.86 (t, *J* = 6.9 Hz, 3H, $OCH_2CH_2CH_2CH_2CH_2CH_2\underline{CH_3}$).

δ_C/ppm (100 MHz, DMSO-d_6): 187.27, 163.26, 162.78, 129.37, 118.76, 108.47, 100.96, 95.53, 68.33, 61.58, 31.20, 29.47, 28.39, 28.37, 25.43, 24.53, 22.01, 18.24, 13.89.

(8) 2-Alkyloxy-4-hydroxybenzaldehydes

To a solution of **Compound 7** (1 eq) in tetrahydrofuran:ethanol (1:1), solid pyridinium *p*-toluenesulfonate (1.5 eq) was added. The quantities of reagents used in the reaction are listed in **Table SI8**. The reaction mixture was allowed to react at reflux overnight and then quenched by evaporation of the solvent to dryness. The reaction crude was dissolved in dichloromethane (200 mL) and washed with water (3 × 200 mL) and brine (200 mL). The organic layer was dried with $MgSO_4$, the drying agent was removed by filtration, and the solvent was removed on the rotary evaporator. The product was then purified by flash column chromatography using a mixture of ethyl acetate and hexane (50:50).

Table SI8. Quantities of reagents used to synthesise 2-alkyloxy-4-hydroxybenzaldehydes.

m	(7)	Pyridinium *p*-toluenesulfonate
2	4.001 g, 1.60×10^{-2} mol	6.051 g, 2.40×10^{-2} mol
3	3.762 g, 1.49×10^{-2} mol	2.672 g, 2.24×10^{-2} mol
4	10.000 g, 3.61×10^{-2} mol	13.601 g, 5.41×10^{-2} mol
5	14.000 g, 4.77×10^{-2} mol	18.000 g, 7.15×10^{-2} mol
6	11.700 g, 3.81×10^{-2} mol	14.410 g, 5.72×10^{-2} mol
7	8.610 g, 2.69×10^{-2} mol	10.120 g, 4.03×10^{-2} mol

8.1 2-Ethoxy-4-hydroxybenzaldehyde

Yellow crystals. Yield: 2.122 g, 80%. RF: 0.11 (20% ethyl acetate:80% hexane). M.P. = 167 ˚C

ν_{max}/cm^{-1}: 3015 (OH), 2980 (-CH_3), 2876 (-CH_2), 1639 (RC(=O)H), 1568 (Ar C=C), 1033 (C-O-C).

δ_H/ppm (400 MHz, DMSO-d_6): 10.62 (s, 1H, ArOH), 10.14 (s, 1H, -HCO), 7.56 (d, *J* = 8.4 Hz, 1H, Ar-H), 6.50 – 6.41 (m, 2H, Ar-H), 4.10 (q, *J* = 6.9 Hz, 2H, O$\underline{CH_2}CH_3$), 1.37 (t, *J* = 6.9 Hz, 3H, $OCH_2\underline{CH_3}$).

δ_C/ppm (100 MHz, DMSO-d_6): 186.89, 165.13, 163.15, 129.77, 116.99, 108.47, 99.52, 63.82, 14.39.

Data consistent with reported literature.[1]

8.2 2-Propoxy-4-hydroxybenzaldehyde

Pink crystals. Yield: 1.300 g, 48%. RF: 0.13 (20% ethyl acetate:80% hexane). M.P. = 167 ˚C

ν_{max}/cm^{-1}: 3126 (OH), 2968 (-CH_3), 2877 (-CH_2), 1639 (RC(=O)H), 1575 (Ar C=C), 1040 (C-O-C).

δ_H/ppm (400 MHz, DMSO-d_6): 10.61 (s, 1H, ArOH), 10.16 (s, 1H, -HCO), 7.56 (d, *J* = 8.4 Hz, 1H, Ar-H), 6.51 – 6.37 (m, 2H, Ar-H), 3.99 (t, *J* = 6.4 Hz, 2H, O$\underline{CH_2}CH_2CH_3$), 1.77 (h, *J* = 6.4 Hz, 2H, $OCH_2\underline{CH_2}CH_3$), 0.99 (t, *J* = 7.4 Hz, 3H, $OCH_2CH_2\underline{CH_3}$).

δ_C/ppm (100 MHz, DMSO-d_6): 186.85, 165.16, 163.34, 129.81, 117.10, 108.49, 99.54, 69.48, 21.89, 10.42.

Data consistent with reported literature.[1]

8.3 2-Butoxy-4-hydroxybenzaldehyde

Brown crystals. Yield: 4.991 g, 71%. RF: 0.13 (20% ethyl acetate:80% hexane). M.P. = 88 ˚C

ν_{max}/cm^{-1}: 3068 (OH), 2954 ($-CH_3$), 2933 ($-CH_2$), 2868 ($-CH_2$), 1637 (RC(=O)H), 1598, (Ar C=C), 1056 (C-O-C).

δ_H/ppm (400 MHz, DMSO-d_6): 10.49 (s, 1H, ArOH), 10.14 (s, 1H, -HCO), 7.56 (d, *J* = 8.5, 1H, Ar-H), 6.50 – 6.41 (m, 2H, Ar-H), 4.04 (t, *J* = 6.3 Hz, 2H, $O\underline{CH_2}CH_2CH_2CH_3$), 1.74 (p, *J* = 6.4 Hz, 2H, $OCH_2\underline{CH_2}CH_2CH_3$), 1.53 – 1.37 (h, *J* = 7.24 Hz, 2H, $OCH_2CH_2\underline{CH_2}CH_3$), 0.98 – 0.90 (t, *J* = 7.2 Hz, 3H, $OCH_2CH_2CH_2\underline{CH_3}$).

δ_C/ppm (100 MHz, DMSO-d_6): 186.84, 165.20, 163.34, 129.79, 117.08, 108.50, 99.54, 67.76, 30.52, 18.78, 13.70.

8.4 2-Pentoxy-4-hydroxybenzaldehyde

Brown crystals. Yield: 11.020 g, 100%. RF: 0.17 (20% ethyl acetate:80% hexane). M.P. = 109 ˚C

ν_{max}/cm^{-1}: 3070 (OH), 2953 ($-CH_3$), 2926 ($-CH_2$), 2870 ($-CH_2$), 1639 (RC(=O)H), 1596, (Ar C=C).

δ_H/ppm (400 MHz, DMSO-d_6): 10.61 (s, 1H, ArOH), 10.14 (s, 1H, -HCO), 7.56 (d, *J* = 8.4 Hz, 1H, Ar-H), 6.49 – 6.41 (m, 2H, Ar-H), 4.03 (t, *J* = 6.8 Hz, 2H, $O\underline{CH_2}CH_2CH_2\,CH_2CH_3$), 1.81 – 1.67 (p, *J* = 6.9 Hz, 2H, $OCH_2\underline{CH_2}CH_2CH_2CH_3$), 1.52 – 1.27 (m, 4H, $OCH_2CH_2\underline{CH_2}\underline{CH_2}CH_3$), 0.89 (t, *J* = 7.1 Hz, 3H, $OCH_2CH_2CH_2CH_2\underline{CH_3}$).

δ_C/ppm (100 MHz, DMSO-d_6): 186.79, 165.16, 163.32, 129.74, 117.06, 108.47, 99.51, 68.03, 28.12, 27.70, 21.85, 13.90.

8.5 2-Hexoxy-4-hydroxybenzaldehyde

Brown crystals. Yield: 9.614 g, 100%. RF: 0.20 (20% ethyl acetate:80% hexane). M.P. = 106 ˚C

ν_{max}/cm^{-1}: 3182 (OH), 2932 ($-CH_2$), 2870 ($-CH_2$), 1660 (RC(=O)H), 1579 (Ar C=C), 1035 (C-O-C).

δ_H/ppm (400 MHz, DMSO-d_6): 10.62 (s, 1H, ArOH), 10.15 (s, 1H, -HCO), 7.56 (d, *J* = 8.4 Hz, 1H, Ar-H), 6.48 – 6.42 (m, 2H, Ar-H), 4.01 (t, *J* = 6.3 Hz, 2H, $O\underline{CH_2}CH_2CH_2CH_2\,CH_3$), 1.82 – 1.64 (m, 2H, $OCH_2\underline{CH_2}CH_2CH_2CH_2CH_3$), 1.49 – 1.34 (m, 4H, $OCH_2CH_2\underline{CH_2}\,\underline{CH_2}CH_2CH_3$), 1.40 – 1.32 (m, 2H, $OCH_2CH_2CH_2CH_2\underline{CH_2}CH_3$), 0.92 – 0.78 (t, *J* = 7.1 Hz, 3H, $OCH_2CH_2CH_2CH_2CH_2\underline{CH_3}$).

δ_C/ppm (100 MHz, DMSO-d_6): 186.75, 165.22, 163.37, 129.70, 117.14, 108.48, 99.48, 68.05, 31.01, 28.45, 25.23, 22.10, 13.85.

8.6 2-Heptoxy-4-hydroxybenzaldehyde

Brown crystals. Yield: 5.690 g, 52%. RF: 0.20 (20% ethyl acetate:80% hexane). M.P. = 86 ˚C

v_{max}/cm^{-1}: 3034 (OH), 2923 (-CH_2), 2854 (-CH_2), 2724, 1636 (RC(=O)H), 1568 (Ar C=C), 1112, 1019 (C-O-C).

δ_H/ppm (400 MHz, DMSO-d_6): 10.60 (s, 1H, ArOH), 10.15 (s, 1H, -HCO), 7.56 (d, *J* = 8.4 Hz, 1H, Ar-H), 6.51 – 6.41 (m, 2H, Ar-H), 4.02 (t, *J* = 6.3 Hz, 2H, O$\underline{CH_2}$$CH_2CH_2CH_2CH_2CH_2CH_3$), 1.74 (p, *J* = 6.3 Hz, 2H, $OCH_2$$\underline{CH_2}$$CH_2CH_2CH_2CH_2CH_3$), 1.48 – 1.20 (m, 8H, $OCH_2CH_2$$\underline{CH_2CH_2CH_2CH_2}$$CH_3$), 0.92 – 0.79 (t, *J* = 6.9 Hz, 3H, $OCH_2CH_2CH_2CH_2CH_2CH_2$$\underline{CH_3}$).

δ_C/ppm (100 MHz, DMSO-d_6): 186.79, 165.13, 163.32, 129.75, 117.08, 108.46, 99.52, 68.04, 31.21, 28.39, 25.46, 22.04, 18.56, 13.93.

Synthesis of Compound 9

Under inert conditions, a mixture of the required **Compound 8** (1 eq) and the required benzoic acid (1.5 eq) in dichloromethane (60 ml) was cooled with an ice bath. To this, dissolved 1-ethyl-3-(3-dimethylaminopropyl)carbodiimide hydrochloride (EDC) (1.5 eq) in dichloromethane (100 mL) was added and allowed to react at 0 ˚C for 30-40 min. The quantities of reagents used in the reaction are listed in **Tables SI9-11**. A catalytic amount of solid 4-dimethylaminopyridine (DMAP) was added to the solution and left to react overnight slowly warming up to room temperature. The reaction mixture was then quenched by the addition of water (50 mL) and washed with water (3 × 100 mL) and brine (50 mL). The organic layer was dried with $MgSO_4$, the drying agent was removed by filtration and the solvent was removed on the rotary evaporator. The product was then purified by hot filtration and recrystallisation from ethanol.

(9.1) (3-Alkyloxy-4-formylphenyl) 4-methylbenzoates

Table SI9. Quantities of reagents used to synthesise (3-alkyloxy-4-formylphenyl) 4-methylbenzoates.

m	(**8**)	4-Methylbenzoic acid	EDC
2	1.300 g, 7.82×10^{-3} mol	1.592 g, 1.17×10^{-2} mol	1.910 g, 1.17×10^{-2} mol
3	1.300 g, 7.82×10^{-3} mol	1.274 g, 9.36×10^{-3} mol	1.794 g, 9.36×10^{-3} mol
4	1.400 g, 7.21×10^{-3} mol	1.473 g, 1.08×10^{-2} mol	2.075 g, 1.08×10^{-2} mol
5	1.300 g, 6.24×10^{-3} mol	1.274 g, 9.36×10^{-3} mol	1.792 g, 9.36×10^{-3} mol
6	1.300 g, 5.84×10^{-3} mol	1.192 g, 8.77×10^{-3} mol	1.674 g, 8.77×10^{-3} mol
7	3.170 g, 1.34×10^{-2} mol	2.741 g, 2.01×10^{-2} mol	3.86 g, 2.01×10^{-3} mol

9.1.1 (3-Ethoxy-4-formylphenyl) 4-methylbenzoate

White powder. Yield: 1.1 g, 29%. RF: 0.51 (20% ethyl acetate:80% hexane). M.P. = 88 ˚C

v_{max}/cm^{-1}: 2985 (-CH_3), 2861 (-CH_2), 1732 (COOR), 1678 (RC(=O)H), 1606 (Ar C=C), 1257 (C-O-C).

δ_H/ppm (400 MHz, $CDCl_3$): 10.34 (s, 1H, -HCO), 8.04 (d, *J* = 8.3 Hz, 2H, Ar-H), 7.78 (d, *J* = 8.5 Hz, 1H, Ar-H), 7.43 (d, *J* = 7.9 Hz, 2H, Ar-H), 7.23 (d, *J* = 2.1 Hz, 1H, Ar-H), 7.00 (dd, *J* = 8.3, 1.4 Hz, 1H, Ar-H), 4.20 (q, *J* = 6.9 Hz, 2H, O$\underline{CH_2}$$CH_3$), 2.43 (s, 3H, -$\underline{CH_3}$), 1.39 (t, *J* = 6.9 Hz, 3H, $OCH_2$$\underline{CH_3}$).

δ_C/ppm (100 MHz, DMSO-d_6): 188.27, 163.98, 161.97, 156.91, 144.88, 130.00, 129.60, 129.00, 125.80, 122.14, 114.56, 107.77, 64.68, 21.30, 14.33.

9.1.2 (3-Propoxy-4-formylphenyl) 4-methylbenzoate

White powder. Yield: 1.08 g, 60%. RF: 0.56 (20% ethyl acetate:80% hexane). M.P. = 71 ˚C

ν_{max}/cm^{-1}: 2959 ($-CH_3$), 2877 ($-CH_2$), 1733 (COOR), 1674 (RC(=O)H), 1604 (Ar C=C), 1389, 1239 (C-O-C).

δ_H/ppm (400 MHz, $CDCl_3$): 10.47 (s, 1H, -HCO), 8.08 (d, J = 7.7 Hz, 2H, Ar-H), 7.91 (d, J = 8.4 Hz, 1H, Ar-H), 7.32 (d, J = 7.8 Hz, 2H, Ar-H), 6.91 – 6.84 (m, 2H, Ar-H), 4.04 (t, J = 7.0 Hz, 2H, $O\underline{CH_2}CH_2CH_3$), 2.46 (s, 3H, $-\underline{CH_3}$), 1.89 (h, J = 6.6 Hz, 2H, $OCH_2\underline{CH_2}CH_3$), 1.08 (t, J = 8.9 Hz, 3H, $OCH_2CH_2\underline{CH_3}$).

δ_C/ppm (100 MHz, $CDCl_3$): 188.90, 164.66, 162.68, 157.21, 145.07, 130.46, 129.72, 129.58, 126.37, 122.85, 114.29, 106.67, 70.50, 22.51, 21.97, 10.66.

9.1.3 (3-Butoxy-4-formylphenyl) 4-methylbenzoate

White powder. Yield: 0.612 g, 27%. RF: 0.59 (20% ethyl acetate:80% hexane). M.P. = 67 ˚C

ν_{max}/cm^{-1}: 2990 ($-CH_3$), 2854 ($-CH_2$), 1726 (COOR), 1686 (RC(=O)H), 1600(Ar C=C), 1392, 1249 (C-O-C).

δ_H/ppm (400 MHz, DMSO-d_6): 10.33 (s, 1H, -HCO), 8.03 (d, J = 8.3 Hz, 2H, Ar-H), 7.78 (d, J = 8.4 Hz, 1H, Ar-H), 7.43 (d, J = 8.0 Hz, 2H, Ar-H), 7.25 (d, J = 2.0 Hz, 1H, Ar-H), 7.00 (dd, J = 8.5, 2.0 Hz, 1H, Ar-H), 4.14 (t, J = 6.4 Hz, 2H, $O\underline{CH_2}CH_2CH_2CH_3$), 2.43 (s, 3H, $-\underline{CH_3}$), 1.77 (p, J = 6.6 Hz, 2H, $OCH_2\underline{CH_2}CH_2CH_3$), 1.47 (h, J = 8.3 Hz, 2H, $OCH_2CH_2\underline{CH_2}CH_3$), 0.94 (t, J = 7.4 Hz, 3H, $OCH_2CH_2CH_2\underline{CH_3}$).

δ_C/ppm (100 MHz, DMSO-d_6): 188.20, 163.99, 162.14, 156.93, 144.88, 130.00, 129.61, 129.02, 125.80, 122.19, 114.56, 107.79, 68.59, 30.43, 21.31, 18.69, 13.67.

9.1.4 (3-Pentoxy-4-formylphenyl) 4-methylbenzoate

White powder. Yield: 1.008 g, 49%. RF: 0.59 (20% ethyl acetate:80% hexane). M.P. = 76 ˚C

ν_{max}/cm^{-1}2946 ($-CH_3$), 2866 ($-CH_2$), 1727 (COOR), 1686 (RC(=O)H), 1601(Ar C=C), 1391, (C-O-C).

δ_H/ppm (400 MHz, $CDCl_3$): 10.46 (s, 1H, -HCO), 8.08 (d, J = 7.8 Hz, 2H, Ar-H), 7.91 (d, J = 9.2 Hz, 1H, Ar-H), 7.32 (d, J = 7.9 Hz, 2H, Ar-H), 6.89 – 6.85 (m, 2H, Ar-H), 4.07 (t, J = 6.4 Hz, 2H, $O\underline{CH_2}CH_2CH_2CH_2CH_3$), 2.46 (s, 3H, $-\underline{CH_3}$), 1.86 (p, J = 6.4 Hz, 2H, $OCH_2\underline{CH_2}CH_2CH_2CH_3$), 1.51 – 1.30 (m, 4H, $OCH_2CH_2\underline{CH_2CH_2}CH_3$), 0.94 (t, J = 7.0 Hz, 3H, $OCH_2CH_2CH_2CH_2\underline{CH_3}$).

δ_C/ppm (100 MHz, $CDCl_3$): 188.94, 164.67, 162.71, 157.23, 145.10, 130.45, 129.71, 129.58, 126.35, 122.83, 114.26, 106.63, 69.05, 28.79, 28.31, 22.52, 21.95, 14.13.

9.1.5 (3-Hexyloxy-4-formylphenyl) 4-methylbenzoate

Creamy powder. Yield: 1.154 g, 58%. RF: 0.61 (20% ethyl acetate:80% hexane). M.P. = 46 ˚C

ν_{max}/cm^{-1}: 2991 ($-CH_3$), 2853 ($-CH_2$), 1727 (COOR), 1686 (RC(=O)H), 1601(Ar C=C), 1389, 1245 (C-O-C).

δ_H/ppm (400 MHz, DMSO-d_6): 10.34 (s, 1H, -HCO), 8.03 (d, J = 7.7 Hz, 2H, Ar-H), 7.77 (d, J = 8.5 Hz, 1H, Ar-H), 7.43 (d, J = 8.0 Hz, 2H, Ar-H), 7.24 (d, J = 2.0 Hz, 1H, Ar-H), 7.00 (dd, J = 8.4, 2.0 Hz, 1H, Ar-H), 4.13 (t, J = 6.4 Hz, 2H, $O\underline{CH_2}CH_2CH_2CH_2CH_2CH_3$), 2.43 (s, 3H, -$\underline{CH_3}$), 1.78 (p, J = 7.7 Hz, 2H, $OCH_2\underline{CH_2}CH_2CH_2CH_2CH_3$), 1.44 (p, J = 7.5 Hz, 2H, $OCH_2CH_2\underline{CH_2}CH_2CH_2CH_3$), 1.38 – 1.24 (m, 4H, $OCH_2CH_2CH_2\underline{CH_2CH_2}CH_3$), 0.87 (t, J = 6.8 Hz, 3H, $OCH_2CH_2CH_2CH_2CH_2\underline{CH_3}$).

δ_C/ppm (100 MHz, DMSO-d_6): 188.17, 163.98, 162.13, 156.93, 144.87, 129.99, 129.60, 129.00, 125.80, 122.18, 114.55, 107.79, 68.87, 30.89, 28.29, 25.07, 22.04, 21.30, 13.89.

9.1.6 (3-Heptyloxy-4-formylphenyl) 4-methylbenzoate

Creamy powder. Yield: 2.675 g, 56%. RF: 0.63 (20% ethyl acetate:80% hexane). M.P. = 50 ˚C

ν_{max}/cm^{-1}: 2922 (-CH_3), 2852 (-CH_2), 1728 (COOR), 1682 (RC(=O)H), 1601(Ar C=C), 1247 (C-O-C).

δ_H/ppm (400 MHz, DMSO-d_6): 10.34 (s, 1H, -HCO), 8.05 (d, J = 8.3 Hz, 2H, Ar-H), 7.77 (d, J = 8.5 Hz, 1H, Ar-H), 7.43 (d, J = 7.9 Hz, 2H, Ar-H), 7.24 (d, J = 2.0 Hz, 1H, Ar-H), 7.00 (dd, J = 8.5, 2.1 Hz, 1H, Ar-H), 4.13 (t, J = 6.4 Hz, 2H, $O\underline{CH_2}CH_2CH_2CH_2CH_2CH_2CH_3$), 2.43 (s, 3H, -$\underline{CH_3}$), 1.78 (p, J = 6.5 Hz, 2H, $OCH_2\underline{CH_2}CH_2CH_2CH_2CH_2CH_3$), 1.44 (p, J = 6.6 Hz, 2H, $OCH_2CH_2\underline{CH_2}CH_2CH_2CH_2CH_3$), 1.36 – 1.24 (m, 6H, $OCH_2CH_2CH_2$ $\underline{CH_2CH_2CH_2}CH_3$), 0.86 (t, J = 6.5 Hz, 3H, $OCH_2CH_2CH_2CH_2CH_2$ $CH_2\underline{CH_3}$).

δ_C/ppm (100 MHz, DMSO-d_6): 188.18, 163.99, 162.14, 156.94, 144.89, 130.00, 129.61, 129.02, 125.81, 122.19, 114.55, 107.79, 68.88, 54.92, 31.22, 28.36, 25.39, 22.05, 21.31, 13.96.

(9.2) (3-Alkyloxy-4-formylphenyl) 2-methoxy-4-methylbenzoates

Table SI10. Quantities of reagents used to synthesise (3-alkyloxy-4-formylphenyl) 2-methoxy-4-methylbenzoates

m	(**8**)	2-Methoxy-4-methylbenzoic acid	EDC
2	0.7 g, 4.21×10^{-3} mol	0.528 g, 3.18×10^{-3} mol	0.61 g, 3.18×10^{-3} mol
3	1.2 g, 5.76×10^{-3} mol	1.44 g, 8.64×10^{-3} mol	1.66 g, 8.64×10^{-3} mol
4	1 g, 5.14×10^{-3} mol	1.28 g, 7.72×10^{-3} mol	1.48 g, 7.72×10^{-3} mol
5	1.5 g, 7.2×10^{-3} mol	1.79 g, 1.08×10^{-2} mol	2.07g, 1.08×10^{-2} mol
6	1.5 g, 6.07×10^{-3} mol	1.51 g, 9.11×10^{-3} mol	1.74 g, 9.11×10^{-3} mol
7	1.5 g, 6.34×10^{-3} mol	1.58 g, 9.52×10^{-3} mol	1.83 g, 9.52×10^{-3} mol

9.2.1 (3-Ethoxy-4-formylphenyl) 2-methoxy-4-methylbenzoate

White powder. Yield: 0.519 g, 51%. RF: 0.20 (20% ethyl acetate:80% hexane). M.P. = 93 ˚C

ν_{max}/cm^{-1}: 2982 (-CH_3), 2882 (-CH_2), 1741 (COOR), 1683 (RC(=O)H), 1594 (Ar C=C), 1258 (C-O-C).

δ_H/ppm (400 MHz, DMSO-d_6): 10.33 (s, 1H, -HCO), 7.86 (d, J = 7.9 Hz, 1H, Ar-H), 7.76 (d, J = 8.5 Hz, 1H, Ar-H), 7.15 (d, J = 2.0 Hz, 1H, Ar-H), 7.08 (d, J = 2.0 Hz, 1H, Ar-H), 7.00 – 6.89 (m, 2H, Ar-H), 4.20 (q, J = 7.0 Hz, 2H, $O\underline{CH_2}CH_3$), 3.86 (s, 3H, $O\underline{CH_3}$), 2.40 (s, 3H, $-\underline{CH_3}$), 1.39 (t, J = 6.9 Hz, 3H, $OCH_2\underline{CH_3}$).

δ_C/ppm (100 MHz, DMSO-d_6): 188.24, 162.76, 161.99, 159.56, 156.98, 145.98, 131.94, 128.93, 121.97, 120.94, 114.83, 114.65, 113.39, 107.71, 64.64, 55.87, 21.56, 14.33.

9.2.2 (3-Propoxy-4-formylphenyl) 2-methoxy-4-methylbenzoate

White powder. Yield: 1.244 g, 60%. RF: 0.22 (20% ethyl acetate:80% hexane). M.P. = 88 ˚C

v_{max}/cm^{-1}: 2964 ($-CH_3$), 2912, 2855 ($-CH_2$), 2764, 1747 (COOR), 1684 (RC(=O)H), 1604 (Ar C=C), 1265 (C-O-C).

δ_H/ppm (400 MHz, DMSO-d_6): 10.35 (s, 1H, -HCO), 7.87 (d, J = 7.9 Hz, 1H, Ar-H), 7.77 (d, J = 8.5 Hz, 1H, Ar-H), 7.16 (d, J = 2.0 Hz, 1H, Ar-H), 7.08 (d, J = 2.0 Hz, 1H, Ar-H), 6.98 – 6.89 (m, 2H, Ar-H), 4.10 (t, J = 6.4 Hz, 2H, $O\underline{CH_2}CH_2CH_3$), 3.86 (s, 3H, $O\underline{CH_3}$), 2.40 (s, 3H, $-\underline{CH_3}$), 1.80 (h, J = 7.0 Hz, 2H, $OCH_2\underline{CH_2}CH_3$), 1.01 (t, J = 7.4 Hz, 3H, $OCH_2CH_2\underline{CH_3}$).

δ_C/ppm (100 MHz, DMSO-d_6): 188.19, 162.77, 162.15, 159.57, 157.00, 146.00, 131.96, 128.98, 122.05, 120.95, 114.83, 114.65, 113.40, 107.73, 70.26, 55.88, 21.83, 21.57, 10.35.

9.2.3 (3-Butoxy-4-formylphenyl) 2-methoxy-4-methylbenzoate

Yellow powder. Yield: 1.141 g, 64%. RF: 0.26 (20% ethyl acetate:80% hexane). M.P. = 63 ˚C

v_{max}/cm^{-1}: 2947 ($-CH_3$), 2885 ($-CH_2$), 1736 (COOR), 1682 (RC(=O)H), 1607 (Ar C=C), 1258 (C-O-C).

δ_H/ppm (400 MHz, DMSO-d_6): 10.33 (s, 1H, -HCO), 7.86 (d, J = 7.9 Hz, 1H, Ar-H), 7.76 (d, J = 8.5 Hz, 1H, Ar-H), 7.16 (d, J = 2.0 Hz, 1H, Ar-H), 7.07 (d, J = 1.5 Hz, 1H, J = 2.0 Hz, Ar-H), 6.98 – 6.89 (m, 2H, Ar-H), 4.13 (t, J = 6.4 Hz, 2H, $O\underline{CH_2}CH_2CH_2CH_3$), 3.86 (s, 3H, OCH_3), 2.40 (s, 3H, $-CH_3$), 1.76 (p, J = 6.5 Hz, 2H, $OCH_2\underline{CH_2}CH_2CH_3$), 1.47 (h, J = 6.7 Hz, 2H, $OCH_2CH_2\underline{CH_2}CH_3$), 0.94 (t, J = 7.4 Hz, 3H, $OCH_2CH_2CH_2\underline{CH_3}$).

δ_C/ppm (100 MHz, DMSO-d_6): 188.25, 162.83, 162.21, 159.62, 157.05, 146.07, 132.01, 129.03, 122.07, 121.00, 114.85, 114.69, 113.43, 107.75, 68.60, 55.92, 30.48, 21.62, 18.74, 13.71.

9.2.4 (3-Pentoxy-4-formylphenyl) 2-methoxy-4-methylbenzoate

Pink powder. Yield: 1.421 g, 55%. RF: 0.28 (20% ethyl acetate:80% hexane). M.P. = 54 ˚C

v_{max}/cm^{-1}: 2950 ($-CH_3$), 2859 ($-CH_2$), 1737 (COOR), 1684 (RC(=O)H), 1604 (Ar C=C), 1257 (C-O-C).

δ_H/ppm (400 MHz, DMSO-d_6): 10.34 (s, 1H, -HCO), 7.87 (d, J = 7.9 Hz, 1H, Ar-H), 7.76 (d, J = 8.5 Hz, 1H, Ar-H), 7.16 (d, J = 2.0 Hz, 1H, Ar-H), 7.08 (d, J = 2.0 Hz, 1H, Ar-H), 6.97 – 6.89 (m, 2H, Ar-H), 4.13 (t, J = 6.4 Hz, 2H, $O\underline{CH_2}CH_2CH_2CH_2CH_3$), 3.86 (s, 3H, $O\underline{CH_3}$), 2.40 (s, 3H, $-\underline{CH_3}$), 1.78 (p, J = 6.4 Hz, 2H, $OCH_2\underline{CH_2}CH_2CH_2CH_3$), 1.49 – 1.29 (m, 4H, $OCH_2CH_2\underline{CH_2CH_2}CH_3$), 0.90 (t, J = 7.1 Hz, 3H, $OCH_2CH_2CH_2CH_2\underline{CH_3}$).

δ_C/ppm (100 MHz, DMSO-d_6): 188.16, 162.76, 162.15, 159.57, 157.01, 146.00, 131.95, 128.94, 122.01, 120.95, 114.82, 114.64, 113.39, 107.72, 68.83, 55.88, 28.05, 27.61,

21.82, 21.57, 13.90.

9.2.5 (3-Hexoxy-4-formylphenyl) 2-methoxy-4-methylbenzoate

Pink powder. Yield: 0.852 g, 37%. RF: 0.31 (20% ethyl acetate:80% hexane). M.P. = 50 ˚C

ν_{max}/cm^{-1}: 2935 (-CH_3), 2854 (-CH_2), 1747 (COOR), 1671 (RC(=O)H), 1600 (Ar C=C), 1258 (C-O-C).

δ_H/ppm (400 MHz, DMSO-d_6): 10.33 (s, 1H, -HCO), 7.86 (d, J = 7.9 Hz, 1H, Ar-H), 7.76 (d, J = 8.5 Hz, 1H, Ar-H), 7.16 (d, J = 2.0 Hz, 1H, Ar-H), 7.07 (d, J = 2.0 Hz, 1H, Ar-H), 6.97 – 6.89 (m, 2H, Ar-H), 4.13 (t, J = 6.4 Hz, 2H, $O\underline{CH_2}CH_2CH_2CH_2CH_2CH_3$), 3.86 (s, 3H, $O\underline{CH_3}$), 2.40 (s, 3H, -$\underline{CH_3}$), 1.82 – 1.74 (m, 2H, $OCH_2\underline{CH_2}CH_2CH_2CH_2CH_3$), 1.50 – 1.39 (m, 2H, $OCH_2CH_2\underline{CH_2}CH_2CH_2CH_3$), 1.38 – 1.24 (m, 4H, $OCH_2CH_2CH_2\underline{CH_2CH_2}CH_3$), 0.87 (t, J = 6.7 Hz, 3H, $OCH_2CH_2CH_2CH_2CH_2\underline{CH_3}$).

δ_C/ppm (100 MHz, DMSO-d_6): 188.13, 162.75, 162.15, 159.57, 157.01, 145.99, 131.95, 128.94, 122.01, 120.94, 114.82, 114.64, 113.39, 107.72, 68.83, 55.87, 30.89, 28.31, 25.08, 22.03, 21.57, 13.88.

9.2.6 (3-Heptyloxy-4-formylphenyl) 2-methoxy-4-methylbenzoate

Yellow powder. Yield: 1.151 g, 47%. RF: 0.33 (20% ethyl acetate:80% hexane). M.P. = 50 ˚C

ν_{max}/cm^{-1}: 2915 (-CH_3), 2855 (-CH_2), 1744 (COOR), 1673 (RC(=O)H), 1603 (Ar C=C), 1258 (C-O-C).

δ_H/ppm (400 MHz, DMSO-d_6): 10.31 (s, 1H, -HCO), 7.86 (d, J = 7.9 Hz, 1H, Ar-H), 7.76 (d, J = 8.4 Hz, 1H, Ar-H), 7.16 (d, J = 2.0 Hz, 1H, Ar-H), 7.07 (d, J = 1.4 Hz, 1H, Ar-H), 6.97 – 6.89 (m, 2H, Ar-H), 4.13 (t, J = 6.4 Hz, 2H, $O\underline{CH_2}CH_2CH_2CH_2CH_2CH_2CH_3$), 3.86 (s, 3H, $O\underline{CH_3}$), 2.40 (s, 3H, -$\underline{CH_3}$), 1.78 (p, J = 6.7 Hz, 2H, $OCH_2\underline{CH_2}CH_2CH_2CH_2CH_3$), 1.50 – 1.39 (m, 2H, $OCH_2CH_2\underline{CH_2}CH_2CH_2CH_2CH_3$), 1.38 – 1.22 (m, 6H, $OCH_2CH_2CH_2\underline{CH_2}\ \underline{CH_2CH_2}CH_3$), 0.90 – 0.82 (m, 3H, $OCH_2CH_2CH_2CH_2CH_2CH_2\underline{CH_3}$).

δ_C/ppm (100 MHz, DMSO-d_6): 188.16, 162.77, 162.16, 159.58, 157.02, 146.01, 131.96, 128.95, 122.02, 120.96, 114.83, 114.65, 113.40, 107.73, 68.84, 55.88, 31.21, 28.36, 28.36, 25.39, 22.05, 21.58, 13.95.

(9.3) (3-Alkyloxy-4-formylphenyl) 2,4-dimethoxybenzoate

Table SI11. Quantities of reagents used to synthesise (3-alkoxy-4-formylphenyl) 2,4-methoxybenzoates.

m	(**8**)	2,4-Dimethoxybenzoic acid	EDC
2	0.850 g, 5.11×10^{-3} mol	1.111 g, 6.13×10^{-3} mol	1.470 g, 7.65×10^{-3} mol
3	0.640 g, 3.51×10^{-3} mol	0.770 g, 4.22×10^{-3} mol	1.000 g, 5.26×10^{-3} mol
4	0.300 g, 1.54×10^{-3} mol	0.337 g, 1.85 ×10^{-3} mol	0.442 g, 2.31×10^{-3} mol
5	2.000 g, 9.6×10^{-3} mol	2.622 g, 1.44×10^{-2} mol	2.764 g, 1.44×10^{-2} mol
6	1.500 g, 6.75×10^{-3} mol	1.661 g, 9.11×10^{-3} mol	1.752 g, 9.11×10^{-3} mol

7	1.500 g, 6.34×10^{-3} mol	1.734 g, 9.52×10^{-3} mol	1.833 g, 9.52×10^{-3} mol

9.3.1 (3-Ethoxy-4-formylphenyl) 2,4-dimethoxybenzoate

White powder. Yield: 0.737 g, 43%. RF: 0.09 (20% ethyl acetate:80% hexane). M.P. = 130 ˚C

ν_{max}/cm^{-1}: 2991 ($-CH_3$), 2852 ($-CH_2$), 1739 (COOR), 1684 (RC(=O)H), 1604 (Ar C=C), 1207 (C-O-C).

δ_H/ppm (400 MHz, DMSO-d_6): 10.33 (s, 1H, -HCO), 7.98 (d, J = 8.8 Hz, 1H, Ar-H), 7.75 (d, J = 8.5 Hz, 1H, Ar-H), 7.13 (d, J = 2.0 Hz, 1H, Ar-H), 6.92 (dd, J = 8.5, 2.0 Hz, 1H, Ar-H), 6.72 (d, J = 2.3 Hz, 1H, Ar-H), 6.68 (dd, J = 8.8, 2.3 Hz, 1H, Ar-H), 4.20 (q, J = 7.0 Hz, 2H, $O\underline{CH_2}CH_3$), 3.92 – 3.84 (m, 6H, 2× $O\underline{CH_3}$), 1.39 (t, J = 7.0 Hz, 3H, $OCH_2\underline{CH_3}$).

δ_C/ppm (100 MHz, DMSO-d_6): 188.24, 165.03, 162.16, 161.98, 161.85, 157.14, 134.14, 128.89, 121.87, 114.71, 109.70, 107.75, 105.74, 99.00, 64.62, 56.02, 55.76, 14.34.

9.3.2 (3-Propoxy-4-formylphenyl) 2,4-dimethoxybenzoate

White powder. Yield: 0.779 g, 61%. RF: 0.14 (20% ethyl acetate:80% hexane). M.P. = 130 ˚C

IR cm^{-1}: 2964 ($-CH_3$), 2851 ($-CH_2$), 1742 (COOR), 1684 (RC(=O)H), 1606 (Ar C=C), 1206 (C-O-C).

δ_H/ppm (400 MHz, DMSO-d_6): 10.35 (s, 1H, -HCO), 7.98 (d, J = 8.7 Hz, 1H, Ar-H), 7.76 (d, J = 8.5 Hz, 1H, Ar-H), 7.14 (d, J = 2.0 Hz, 1H, Ar-H), 6.92 (dd, J = 8.5, 2.0 Hz, 1H, Ar-H), 6.72 (d, J = 2.3 Hz, 1H, Ar-H), 6.68 (dd, J = 8.8, 2.3 Hz, 1H, Ar-H), 4.10 (t, J = 6.4 Hz, 2H, $O\underline{CH_2}CH_2CH_3$), 3.92 – 3.84 (m, 2× $O\underline{CH_3}$), 1.80 (h, J = 7.0 Hz, 2H, $OCH_2\underline{CH_2}CH_3$), 1.01 (t, J = 7.4 Hz, 3H, $OCH_2CH_2\underline{CH_3}$).

δ_C/ppm (100 MHz, DMSO-d_6): 188.16, 165.02, 162.15, 162.13, 161.85, 157.15, 134.14, 128.92, 121.93, 114.70, 109.70, 107.76, 105.74, 99.00, 70.23, 56.02, 55.76, 21.83, 10.35.

9.3.3 (3-Butoxy-4-formylphenyl) 2,4-dimethoxybenzoate

White powder. Yield: 0.336 g, 60%. RF: 0.16 (20% ethyl acetate:80% hexane). M.P. = 123 ˚C

IR cm^{-1}: 2957 ($-CH_3$), 2871 ($-CH_2$), 1742 (COOR), 1681 (RC(=O)H), 1610 (Ar C=C), 1234 (C-O-C).

δ_H/ppm (400 MHz, DMSO-d_6): 10.33 (s, 1H, -HCO), 7.98 (d, J = 8.7 Hz, 1H, Ar-H), 7.75 (d, J = 8.5 Hz, 1H, Ar-H), 7.14 (d, J = 2.0 Hz, 1H, Ar-H), 6.92 (dd, J = 8.5, 2.0 Hz, 1H, Ar-H), 6.72 (d, J = 2.3 Hz, 1H, Ar-H), 6.68 (dd, J = 8.8, 2.4 Hz, 1H, Ar-H), 4.12 (t, J = 6.4 Hz, 2H, $O\underline{CH_2}CH_2CH_2CH_3$), 3.92 – 3.84 (m, 6H, 2× $O\underline{CH_3}$), 1.78 (p, J = 6.6 Hz, 2H, $OCH_2\underline{CH_2}CH_2CH_3$), 1.48 – 1.42 (m, 2H, $OCH_2CH_2\underline{CH_2}CH_3$), 0.86 (t, J = 5.8 Hz, 3H, $OCH_2CH_2CH_2\underline{CH_3}$).

δ_C/ppm (100 MHz, DMSO-d_6): 188.63, 165.50, 162.63, 162.61, 162.32, 157.63, 135.02, 129.37, 122.38, 115.18, 110.16, 108.23, 106.21, 99.47, 68.99, 56.49, 56.23, 30.92, 19.16, 14.13.

9.3.4 (3-Pentoxy-4-formylphenyl) 2,4-dimethoxybenzoate

Pink powder. Yield: 2.181 g, 60%. RF: 0.16 (20% ethyl acetate:80% hexane). M.P. = 85 ˚C

ν_{max}/cm^{-1}: 2939 ($-CH_3$), 2855 ($-CH_2$), 1746 (COOR), 1674 (RC(=O)H), 1600 (Ar C=C), 1232 (C-O-C).

δ_H/ppm (400 MHz, DMSO-d_6): 10.33 (s, 1H, -HCO), 7.98 (d, J = 8.7 Hz, 1H, Ar-H), 7.75 (d, J = 8.5 Hz, 1H, Ar-H), 7.14 (d, J = 2.0 Hz, 1H, Ar-H), 6.92 (dd, J = 8.5, 2.0 Hz, 1H, Ar-H), 6.72 (d, J = 2.3 Hz, 1H, Ar-H), 6.68 (dd, J = 8.8, 2.2 Hz, 1H, Ar-H), 4.12 (t, J = 6.4 Hz, 2H, $O\underline{CH_2}CH_2CH_2CH_2CH_3$), 3.92 – 3.84 (m, 6H, 2× $O\underline{CH_3}$), 1.78 (p, J = 6.6 Hz, 2H, $OCH_2\underline{CH_2}CH_2CH_2CH_3$), 1.49 – 1.29 (m, 4H, $OCH_2CH_2\underline{CH_2}\underline{CH_2}CH_3$), 0.90 (t, J = 7.1 Hz, 3H, $OCH_2CH_2CH_2CH_2\underline{CH_3}$).

δ_C/ppm (100 MHz, DMSO-d_6): 188.61, 165.49, 162.61, 162.32 (2× CH), 157.63, 134.61, 129.35, 122.38, 115.17, 110.17, 108.23, 106.21, 99.47, 69.27, 56.49, 56.22, 28.53, 28.09, 22.29, 14.37.

9.3.5 (3-Hexyloxy-4-formylphenyl) 2,4-dimethoxybenzoate

White powder. Yield: 1.08 g, 33%. RF: 0.19 (20% ethyl acetate:80% hexane). M.P. = 86 ˚C

v_{max}/cm^{-1}: 2941 (-CH_3), 2852 (-CH_2), 1746 (COOR), 1676 (RC(=O)H), 1601 (Ar C=C), 1232 (C-O-C).

δ_H/ppm (400 MHz, DMSO-d_6): 10.33 (s, 1H, -HCO), 7.98 (d, J = 8.7 Hz, 1H, Ar-H), 7.75(d, J = 8.4 Hz, 1H, Ar-H), 7.13 (d, J = 2.0 Hz, 1H, Ar-H), 6.92 (dd, J = 8.4, 1.9 Hz, 1H, Ar-H), 6.75 – 6.64 (m, 2H, Ar-H), 4.36 (t, J = 5.1 Hz, 2H, $O\underline{CH_2}CH_2CH_2CH_2CH_2CH_3$), 3.93 – 3.85 (m, 6H, 2× $O\underline{CH_3}$), 1.77 (p, J = 6.6 Hz, 2H, $OCH_2\underline{CH_2}CH_2CH_2CH_2CH_3$), 1.44 (p, J = 7.5 Hz, 2H, $OCH_2CH_2\underline{CH_2}CH_2CH_2CH_3$), 1.38 – 1.26 (m, 4H, $OCH_2CH_2CH_2\underline{CH_2}\underline{CH_2}CH_3$), 0.88 (t, J = 6.1 Hz, 3H, $OCH_2CH_2CH_2CH_2CH_2\underline{CH_3}$).

δ_C/ppm (100 MHz, DMSO-d_6): 188.13, 165.02, 162.14, 162.13, 161.85, 157.16, 134.13, 128.88, 121.90, 114.69, 109.69, 107.76, 105.73, 98.99, 68.80, 56.01, 55.75, 31.20, 28.34, 25.38, 22.03, 13.94.

9.3.6 (3-Heptyloxy-4-formylphenyl) 2,4-dimethoxybenzoate

White powder. Yield: 1.41 g, 55%. RF: 0.19 (20% ethyl acetate:80% hexane). M.P. = 65 ˚C

v_{max}/cm^{-1}: 2941 (-CH_3), 2852 (-CH_2), 1746 (COOR), 1673 (RC(=O)H), 1601 (Ar C=C), 1232 (C-O-C).

δ_H/ppm (400 MHz, DMSO-d_6): 10.75 (s, 1H, -HCO), 8.39 (d, J = 8.7 Hz, 1H, Ar-H), 8.17 (d, J = 8.4 Hz, 1H, Ar-H), 7.55 (d, J = 2.0 Hz, 1H, Ar-H), 7.33 (dd, J = 8.5, 2.0, 1H, Ar-H), 7.13 (d, J = 2.3 Hz, 1H, Ar-H), 7.10 (dd, J = 8.8, 2.3 Hz, 1H, Ar-H), 4.54 (t, J = 6.4 Hz, 2H, $O\underline{CH_2}CH_2CH_2CH_2CH_2CH_2CH_3$), 4.32 – 4.26 (m, 6H, 2× $O\underline{CH_3}$), 1.91 – 1.80 (p, J = 7.06 Hz, 2H, $OCH_2\underline{CH_2}CH_2CH_2CH_2CH_2CH_3$), 1.80 – 1.62 (m, 8H, $OCH_2CH_2\underline{CH_2}\underline{CH_2}\underline{CH_2}\underline{CH_2}CH_3$), 1.32 – 1.24 (m, 3H, $OCH_2CH_2CH_2CH_2CH_2CH_2\underline{CH_3}$).

δ_C/ppm (100 MHz, DMSO-d_6): 188.16, 165.05, 162.18, 162.16, 161.88, 157.19, 134.16, 128.91, 121.93, 114.72, 109.71, 107.77, 105.76, 99.00, 68.83, 56.03, 55.77, 31.23, 30.70, 28.37, 25.40, 22.06, 13.95.

Synthesis of Compound 10

To a mixture of **Compound 9** (1 eq) and resorcinol (1.5 eq) in dimethyl sulfoxide (DMSO) (30 mL), a solution of $H_2NaO_4P.H_2O$ (4 eq) and $ClNaO_2$ (3.5 eq) in water (10 mL) was slowly added dropwise. The quantities of reagents used in the reaction are listed in **Tables SI12-14**. The reaction mixture was allowed to react overnight and then diluted with water (150 mL). The pH was adjusted to 8 with an aqueous solution of saturated $NaHCO_3$ and left for 1 h then acidified with 1 M HCl solution to pH 4 until precipitation was observed. The precipitated solid was filtered and washed with water. The product was purified by hot recrystallisation and filtration with ethanol.

10.1 2-Alkyloxy-4-(4-methylbenzoyl)oxybenzoic acids

Tables SI12. Quantities of reagents used to synthesise 2-alkoxy-4-(4-methylbenzoyl)oxybenzoic acids.

m	(**9.1**)	$H_2NaO_4P.H_2O$	$ClNaO_2$	Resorcinol
2	1.000 g, 3.52×10^{-3} mol	2.501 g, 1.41×10^{-2} mol	1.11 g, 1.23×10^{-2} mol	0.581 g, 5.28×10^{-3} mol
3	1.000 g, 3.35×10^{-3} mol	2.382 g, 1.34×10^{-2} mol	1.061 g, 1.17×10^{-2} mol	0.553 g, 5.03×10^{-3} mol
4	0.600 g, 1.95×10^{-3} mol	1.394 g, 7.80×10^{-3} mol	0.617 g, 6.82×10^{-3} mol	0.323 g, 2.93×10^{-3} mol
5	0.972 g, 2.97×10^{-3} mol	2.1114 g, 1.19×10^{-2} mol	0.940 g, 1.04×10^{-2} mol	0.492 g, 4.46×10^{-3} mol
6	1.150 g, 3.38×10^{-3} mol	2.413 g, 1.35×10^{-2} mol	1.074 g, 1.18×10^{-2} mol	0.558 g, 5.07×10^{-3} mol
7	2.600 g, 7.33×10^{-3} mol	5.224 g, 2.93×10^{-2} mol	2.321 g, 2.56×10^{-2} mol	1.211 g, 1.10×10^{-2} mol

10.1.1 2-Ethoxy-4-(4-methylbenzoyl)oxybenzoic acid

White powder. Yield: 1.051 g, 86%. RF: 0 (20% ethyl acetate:80% hexane). M.P. = 182 ˚C

ν_{max}/cm^{-1}: 2986 ($-CH_3$), 2880 ($-CH_2$), 1732 (COOR), 1672 (COOH dimer), 1606 (Ar C=C), 1248 (C-O-C).

δ_H/ppm (400 MHz, DMSO-d_6): 8.03 (d, *J* = 8.4 Hz, 2H, Ar-H), 7.71 (d, *J* = 8.4 Hz, 1H, Ar-H), 7.42 (d, *J* = 8.0 Hz, 2H, Ar-H), 7.08 (d, *J* = 2.1 Hz, 1H, Ar-H), 6.90 (dd, *J* = 8.4, 2.1 Hz, 1H, Ar-H), 4.09 (q, *J* = 6.9 Hz, 2H, $O\underline{CH_2}CH_3$), 2.43 (s, 3H, $-\underline{CH_3}$), 1.32 (t, *J* = 6.9 Hz, 3H, $OCH_2\underline{CH_3}$).

δ_C/ppm (100 MHz, DMSO-d_6): 166.83, 164.25, 158.54, 154.13, 144.77, 131.73, 129.98, 129.61, 125.99, 119.32, 113.58, 107.73, 64.44, 21.33, 14.50.

10.1.2 2-Propoxy-4-(4-methylbenzoyl)oxybenzoic acid

White powder. Yield: 0.786 g, 74%. RF: 0 (20% ethyl acetate:80% hexane). M.P. = 151 ˚C

ν_{max}/cm^{-1}: 2967 ($-CH_3$), 2875 ($-CH_2$), 1735 (COOR), 1675 (COOH dimer), 1606 (Ar C=C), 1247 (C-O-C).

δ_H/ppm (400 MHz, $CDCl_3$): 10.81 (s, 1H, -COOH), 8.27 (d, *J* = 8.4 Hz, 1H, Ar-H), 8.08 (d, *J* = 7.9 Hz, 2H, Ar-H), 7.33 (d, *J* = 7.9 Hz, 2H, Ar-H), 7.07 – 6.93 (m, 2H, Ar-H), 4.23 (t, *J* = 6.5 Hz, 2H, $O\underline{CH_2}CH_2CH_3$), 2.47 (s, 3H, $-\underline{CH_3}$), 1.97 (h, *J* = 7.2 Hz, 2H, $OCH_2\underline{CH_2}CH_3$), 1.11 (t, *J* = 7.4 Hz, 3H, $OCH_2CH_2\underline{CH_3}$).

δ_C/ppm (100 MHz, DMSO-d_6): 164.74, 164.59, 158.43, 156.22, 145.29, 135.12, 130.47, 129.63, 126.12, 115.76, 115.33, 106.94, 72.28, 22.37, 21.97, 10.52.

10.1.3 2-Butoxy-4-(4-methylbenzoyl)oxybenzoic acid

White powder. Yield: 0.305 g, 47%. RF: 0.1 (20% ethyl acetate:80% hexane). M.P. = 130 ˚C

ν_{max}/cm^{-1}: 2960 (-CH_3), 2875 (-CH_2), 1731 (COOR), 1672 (COOH), 1603 (Ar C=C), 1248 (C-O-C).

δ_H/ppm (400 MHz, DMSO-d_6): 12.59 (s, 1H, -COOH), 8.03 (d, *J* = 8.5 Hz, 2H, Ar-H), 7.71 (d, *J* = 8.4 Hz, 1H, Ar-H), 7.42 (d, *J* = 8.0 Hz, 2H, Ar-H), 7.10 (d, *J* = 2.1 Hz, 1H, Ar-H), 6.90 (dd, *J* = 8.4, 2.1 Hz, 1H, Ar-H), 4.02 (t, *J* = 6.4 Hz, 2H, $O\underline{CH_2}CH_2CH_2CH_3$), 2.43 (s, 3H, -$\underline{CH_3}$), 1.70 (p, *J* = 6.4 Hz, 2H, $OCH_2\underline{CH_2}CH_2CH_3$), 1.46 (p, *J* = 8.4 Hz, 2H, $OCH_2CH_2\,\underline{CH_2}CH_3$), 0.92 (t, *J* = 7.4 Hz, 3H, $OCH_2CH_2CH_2\underline{CH_3}$).

δ_C/ppm (100 MHz, DMSO-d_6): 166.79, 164.20, 158.70, 154.11, 144.71, 131.66, 129.93, 129.56, 125.97, 119.18, 113.47, 107.61, 68.28, 30.58, 21.28, 18.60, 13.64.

10.1.4 2-Pentoxy-4-(4-methylbenzoyl)oxybenzoic acid

White powder. Yield: 0.746 g, 73%. RF: 0.1 (20% ethyl acetate:80% hexane). M.P. = 112 ˚C

ν_{max}/cm^{-1}: 2956 (-CH_3), 2875 (-CH_2), 1733 (COOR), 1671 (COOH dimer), 1604 (Ar C=C), 1245 (C-O-C).

δ_H/ppm (400 MHz, $CDCl_3$): 10.80 (s, 1H, -COOH), 8.27 (d, *J* = 8.4 Hz, 1H, Ar-H), 8.08 (d, *J* = 7.9 Hz, 2H, Ar-H), 7.33 (d, *J* = 7.9 Hz, 2H, Ar-H), 7.04 – 6.97 (m, 2H, Ar-H), 4.25 (t, *J* = 6.4 Hz, 2H, $O\underline{CH_2}CH_2CH_2CH_2CH_3$), 2.47 (s, 3H, -$\underline{CH_3}$), 1.94 (p, *J* = 6.8 Hz, 2H, $OCH_2\underline{CH_2}CH_2CH_2CH_3$), 1.54 – 1.35 (m, 4H, $OCH_2CH_2\underline{CH_2CH_2}CH_3$), 0.95 (t, *J* = 7.1 Hz, 3H, $OCH_2CH_2CH_2CH_2\underline{CH_3}$).

δ_C/ppm (100 MHz, $CDCl_3$): 164.72, 164.59, 158.45, 156.22, 145.29, 135.13, 130.48, 129.63, 126.13, 115.76, 115.33, 106.92, 70.85, 28.65, 28.08, 22.41, 21.97, 14.01.

10.1.5 2-Hexyloxy-4-(4-methylbenzoyl)oxybenzoic acid

White powder. Yield: 0.652 g, 56%. RF: 0.2 (20% ethyl acetate:80% hexane). M.P. = 104 ˚C

ν_{max}/cm^{-1}: 2949 (-CH_3), 2872 (-CH_2), 1734 (COOR), 1675 (COOH dimer), 1605 (Ar C=C), 1245 (C-O-C).

δ_H/ppm (400 MHz, DMSO-d_6): 12.58 (s, 1H, -COOH), 8.02 (d, *J* = 7.9 Hz, 2H, Ar-H), 7.71 (d, *J* = 8.4 Hz, 1H, Ar-H), 7.42 (dd, *J* = 596.7, 7.7 Hz, 2H, Ar-H), 7.09 (d, *J* = 2.1 Hz, 1H, Ar-H), 6.90 (dd, *J* = 8.4, 2.1 Hz, 1H, Ar-H), 4.01 (t, *J* = 6.4 Hz, 2H, $O\underline{CH_2}CH_2CH_2CH_2CH_2CH_3$), 2.43 (s, 3H, -$\underline{CH_3}$), 1.70 (p, *J* = 6.4 Hz, 2H, $OCH_2\underline{CH_2}CH_2CH_2CH_2CH_3$), 1.43 (p, *J* = 6.6 Hz, 2H, $OCH_2CH_2\underline{CH_2}CH_2CH_2CH_3$), 1.40 – 1.30 (m, 4H, $OCH_2CH_2\,CH_2\underline{CH_2CH_2}CH_3$), 0.87 (d, *J* = 7.3 Hz, 3H, $OCH_2CH_2CH_2CH_2CH_2\underline{CH_3}$).

δ_C/ppm (100 MHz, DMSO-d_6): 166.77, 164.20, 158.72, 154.14, 144.72, 131.69, 129.94, 129.57, 125.97, 119.11, 113.48, 107.63, 68.58, 30.85, 28.43, 24.98, 22.07, 21.29, 13.89.

10.1.6 2-Heptyloxy-4-(4-methylbenzoyl)oxybenzoic acid

White powder. Yield: 1.834 g, 67%. RF: 0.3 (20% ethyl acetate:80% hexane. M.P. = 107 ˚C

v_{max}/cm^{-1}: 2954 (-CH_3), 2855 (-CH_2), 1735 (COOR), 1676 (COOH dimer), 1606 (Ar C=C), 1246 (C-O-C).

δ_H/ppm (400 MHz, DMSO-d_6): 12.61 (s, 1H, -COOH), 8.03 (d, *J* = 8.0 Hz, 2H, Ar-H), 7.71 (d, *J* = 8.4 Hz, 1H, Ar-H), 7.42 (d, *J* = 8.1 Hz, 2H, Ar-H), 7.09 (d, *J* = 2.1 Hz, 1H, Ar-H), 6.90 (dd, *J* = 8.4, 2.1 Hz, 1H, Ar-H), 4.01 (t, *J* = 6.4 Hz, 2H, O$\underline{CH_2}$$CH_2CH_2CH_2CH_2CH_2CH_3$), 2.43 (s, 3H, -$\underline{CH_3}$), 1.70 (p, *J* = 8.2 Hz, 2H, $OCH_2\underline{CH_2}CH_2CH_2CH_2CH_2CH_3$), 1.42 (p, *J* = 7.0 Hz, 2H, $OCH_2CH_2\underline{CH_2}CH_2CH_2CH_2CH_3$), 1.36 – 1.20 (m, 6H, $OCH_2CH_2CH_2$ $\underline{CH_2CH_2CH_2}CH_3$), 0.87 (t, *J* = 6.4 Hz, 3H, $OCH_2CH_2CH_2CH_2CH_2CH_2\underline{CH_3}$).

δ_C/ppm (100 MHz, DMSO-d_6): 166.79, 164.21, 158.71, 154.12, 144.73, 131.68, 129.94, 129.57, 125.98, 119.19, 113.47, 107.63, 68.57, 31.26, 28.49, 28.33, 25.30, 22.04, 21.30, 13.98.

10.2 2-Alkyloxy-4-(2-methoxy-4-methylbenzoyl)oxybenzoic acids.

Tables SI13. Quantities of reagents used to synthesise 2-alkyloxy-4-(2-methoxy-4-methylbenzoyl) oxybenzoic acids.

m	(**9.2**)	$H_2NaO_4P.H_2O$	$ClNaO_2$	Resorcinol
2	0.514 g, 1.64×10^{-3} mol	1.174 g, 6.56×10^{-3} mol	0.519 g, 5.74×10^{-3} mol	0.270 g, 2.45×10^{-3} mol
3	1.120 g, 3.08×10^{-3} mol	2.192 g, 1.23×10^{-2} mol	0.975 g, 1.07×10^{-2} mol	0.509 g, 4.63×10^{-3} mol
4	1.000 g, 2.91×10^{-3} mol	2.074 g, 1.16×10^{-2} mol	0.921 g, 1.02×10^{-2} mol	0.481 g, 4.37×10^{-3} mol
5	1.301 g, 3.49×10^{-3} mol	2.492 g, 1.40×10^{-2} mol	1.101 g, 1.22×10^{-2} mol	0.578 g, 5.25×10^{-3} mol
6	0.800 g, 2.07×10^{-3} mol	1.541 g, 8.64×10^{-3} mol	0.683 g, 7.52×10^{-3} mol	0.356 g, 3.23×10^{-3} mol
7	1.060 g, 2.60×10^{-3} mol	1.854 g, 1.04×10^{-2} mol	0.823 g, 9.10×10^{-3} mol	0.430 g, 3.90×10^{-3} mol

10.2.1 2-Ethoxy-4-(2-methoxy-4-methylbenzoyl)oxybenzoic acid

White powder. Yield: 0.440 g, 81%. RF: 0 (20% ethyl acetate:80% hexane). M.P. = 143 ˚C

v_{max}/cm^{-1}: 2923 (-CH_3), 2869 (-CH_2), 1741 (COOR), 1674 (COOH dimer), 1605 (Ar C=C), 1216 (C-O-C).

δ_H/ppm (400 MHz, DMSO-d_6): 12.61 (s, 1H, -COOH), 7.85 (d, *J* = 7.9 Hz, 1H, Ar-H), 7.69 (d, *J* = 8.4 Hz, 1H, Ar-H), 7.07 (d, *J* = 2.1 Hz, 1H, Ar-H), 7.00 (d, *J* = 2.1 Hz, 1H), 6.92 (d, *J* = 8.0 Hz, 1H, Ar-H), 6.84 (dd, *J* = 8.4, 2.1 Hz, 1H, Ar-H), 4.09 (q, *J* = 6.9 Hz, 2H, O$\underline{CH_2}$$CH_3$), 3.86 (s, 3H, O$\underline{CH_3}$), 2.40 (s, 3H, -$\underline{CH_3}$), 1.32 (t, *J* = 7.0 Hz, 3H, $OCH_2\underline{CH_3}$).

δ_C/ppm (100 MHz, DMSO-d_6): 166.85, 163.07, 159.43, 158.45, 154.05, 145.74, 131.84, 131.59, 120.91, 118.87, 115.12, 113.60, 113.35, 107.66, 64.35, 55.85, 21.55, 14.48.

10.2.2 2-Propoxy-4-(2-methoxy-4-methylbenzoyl)oxybenzoic acid

White powder. Yield: 1.031 g, 90%. RF: 0 (20% ethyl acetate:80% hexane). M.P. = 132 ˚C

ν_{max}/cm^{-1}: 2967 (-CH_3), 2874 (-CH_2), 1742 (COOR), 1675 (COOH dimer), 1606 (Ar C=C), 1215 (C-O-C).

δ_H/ppm (400 MHz, DMSO-d_6): 12.57 (s, 1H, -COOH dimer), 7.85 (d, *J* = 7.9 Hz, 1H, Ar-H), 7.71 (d, *J* = 8.4 Hz, 1H, Ar-H), 7.07 (d, *J* = 1.1 Hz, 1H, Ar-H), 7.01 (d, *J* = 2.1 Hz, 1H, Ar-H), 6.92 (dd, *J* = 7.9, 1.5 Hz, 1H, Ar-H), 6.84 (dd, *J* = 8.4, 2.1 Hz, 1H, Ar-H), 3.99 (t, *J* = 6.4 Hz, 2H, O$\underline{CH_2}CH_2CH_3$), 3.86 (s, 3H, O$\underline{CH_3}$), 2.40 (s, 3H, -$\underline{CH_3}$), 1.73 (h, *J* = 13.7 Hz, 2H, $OCH_2\underline{CH_2}CH_3$), 0.99 (t, *J* = 7.4 Hz, 3H, $OCH_2CH_2\underline{CH_3}$).

δ_C/ppm (100 MHz, DMSO-d_6): 166.77, 163.06, 159.45, 158.74, 154.21, 145.75, 131.85, 131.71, 120.91, 119.31, 115.10, 113.56, 113.36, 107.60, 70.00, 55.85, 21.93, 21.55, 10.37.

10.2.3 2-Butoxy-4-(2-methoxy-4-methylbenzoyl)oxybenzoic acid

White powder. Yield: 0.614 g, 58%. RF: 0 (20% ethyl acetate:80% hexane). M.P. = 101 ˚C

ν_{max}/cm^{-1}: 2941 (-CH_3), 2874 (-CH_2), 1742 (COOR), 1674 (COOH dimer), 1604 (Ar C=C), 1499, 1404, 1264, 1217 (C-O-C), 1163, 1023, 769, 691, 578, 483.

δ_H/ppm (400 MHz, DMSO-d_6): 12.55 (s, 1H, -COOH), 7.85 (d, *J* = 7.9 Hz, 1H, Ar-H), 7.70 (d, *J* = 8.4 Hz, 1H, Ar-H), 7.07 (d, *J* = 1.4 Hz, 1H, Ar-H), 7.02 (d, *J* = 2.1 Hz, 1H, Ar-H), 6.91 (dd, *J* = 8.0, 1.5 Hz, 1H, Ar-H), 6.84 (dd, *J* = 8.4, 2.1 Hz, 1H, Ar-H), 4.02 (t, *J* = 6.3 Hz, 2H, O$\underline{CH_2}CH_2CH_2CH_3$), 3.86 (s, 3H, O$\underline{CH_3}$), 2.40 (s, 3H, -$\underline{CH_3}$), 1.69 (p, *J* = 6.4 Hz, 2H, $OCH_2\underline{CH_2}CH_2CH_3$), 1.46 (h, *J* = 7.8 Hz, 2H, $OCH_2CH_2\underline{CH_2}CH_3$), 0.92 (t, *J* = 7.4 Hz, 3H, $OCH_2CH_2CH_2\underline{CH_3}$).

δ_C/ppm (100 MHz, DMSO-d_6): 166.79, 163.07, 159.45, 158.73, 154.20, 145.77, 131.87, 131.67, 120.92, 118.84, 115.11, 113.57, 113.36, 107.60, 68.27, 55.86, 30.60, 21.56, 18.61, 13.65.

10.2.4 2-Pentoxy-4-(2-methoxy-4-methylbenzoyl)oxybenzoic acid

White powder. Yield: 0.917 g, 67%. RF: 0.04 (20% ethyl acetate:80% hexane). M.P. = 85 ˚C

ν_{max}/cm^{-1}: 2954 (-CH_3), 2854 (-CH_2), 1741 (COOR), 1669 (COOH dimer), 1604 (Ar C=C), 1216 (C-O-C).

δ_H/ppm (400 MHz, DMSO-d_6): 12.37 (s, 1H, -COOH), 7.85 (d, *J* = 7.9 Hz, 1H, Ar-H), 7.68 (d, *J* = 8.4 Hz, 1H, Ar-H), 7.06 (d, *J* = 2.0 Hz, 1H, Ar-H), 7.00 (d, *J* = 2.1 Hz, 1H, Ar-H), 6.92 (dd, *J* = 8.1, 1.4 Hz, 1H, Ar-H), 6.83 (dd, *J* = 8.4, 2.1 Hz, 1H, Ar-H), 4.01 (t, *J* = 6.4 Hz, 2H, O$\underline{CH_2}CH_2CH_2CH_2CH_3$), 3.86 (s, 3H, O$\underline{CH_3}$), 2.40 (s, 3H, -$\underline{CH_3}$), 1.77 – 1.65 (m, 2H, $OCH_2\underline{CH_2}CH_2CH_2CH_3$), 1.47 – 1.26 (m, 4H, $OCH_2CH_2\underline{CH_2CH_2}CH_3$), 0.88 (t, *J* = 7.1 Hz, 3H, $OCH_2CH_2CH_2CH_2\underline{CH_3}$).

δ_C/ppm (100 MHz, DMSO-d_6): 166.89, 163.08, 159.45, 158.68, 154.11, 145.76, 131.86, 131.61, 120.92, 119.18, 115.12, 113.55, 113.36, 107.61, 68.56, 55.86, 28.19, 27.55, 21.78, 21.56, 13.93.

10.2.5 2-Hexyloxy-4-(2-methoxy-4-methylbenzoyl)oxybenzoic acid)

White powder. Yield: 0.550 g, 65%. RF: 0.04 (20% ethyl acetate:80% hexane). M.P. = 60 ˚C

ν_{max}/cm^{-1}: 2934 (-CH_3), 2857 (-CH_2), 1738 (COOR), 1673 (COOH dimer), 1606 (Ar C=C), 1221 (C-O-C).

δ_H/ppm (400 MHz, DMSO-d_6): 12.59 (s, 1H, -COOH), 7.86 (d, *J* = 7.9 Hz, 1H, Ar-H), 7.70 (d, *J* = 8.4 Hz, 1H, Ar-H), 7.07 (d, *J* = 2.0 Hz, 1H, Ar-H), 7.01 (d, *J* = 2.1 Hz, 1H, Ar-H), 6.92 (dd, *J* = 8.3, 2.0 Hz, 1H, Ar-H), 6.84 (dd, *J* = 8.4, 2.0 Hz, 1H, Ar-H), 4.01 (t, *J* = 6.4 Hz, 2H, O$\underline{CH_2}CH_2CH_2CH_2CH_2CH_3$), 3.86 (s, 3H,

$O\underline{CH_3}$), 2.40 (s, 3H, -$\underline{CH_3}$), 1.70 (p, *J* = 6.5 Hz, 2H, $OCH_2\underline{CH_2}CH_2CH_2CH_2CH_3$), 1.43 (p, *J* = 7.2 Hz, 2H, $OCH_2CH_2\underline{CH_2}CH_2CH_2CH_3$), 1.30 – 1.20 (m, 4H, $OCH_2CH_2CH_2\underline{CH_2CH_2}CH_3$), 0.87 (t, *J* = 7.0 Hz, 3H, $OCH_2CH_2CH_2CH_2CH_2\underline{CH_3}$).

δ_C/ppm (100 MHz, DMSO-d_6): 166.81, 163.08, 159.48, 158.77, 154.23, 145.81, 131.91, 131.72, 120.93, 118.90, 115.08, 113.59, 113.36, 107.62, 68.55, 55.86, 30.89, 28.48, 25.02, 22.10, 21.58, 13.92.

10.2.6 2-Heptyloxy-4-(2-methoxy-4-methylbenzoyl)oxybenzoic acid

White powder. Yield: 0.783 g, 75%. RF: 0.04 (20% ethyl acetate:80% hexane). M.P. = 53 ˚C

v_{max}/cm^{-1}: 2932 (-CH_3), 2850 (-CH_2), 1738 (COOR), 1670 (COOH dimer), 1604 (Ar C=C), 1219 (C-O-C).

δ_H/ppm (400 MHz, DMSO-d_6): 12.53 (s, 1H, -COOH), 7.85 (d, *J* = 7.9 Hz, 1H, Ar-H), 7.70 (d, *J* = 8.4 Hz, 1H, Ar-H), 7.07 (d, *J* = 2.0 Hz, 1H, Ar-H), 7.01 (d, *J* = 2.1 Hz, 1H, Ar-H), 6.92 (dd, *J* = 8.0, 1.5 Hz, 1H, Ar-H), 6.84 (dd, *J* = 8.4, 2.1 Hz, 1H, Ar-H), 4.01 (t, *J* = 6.4 Hz, 2H, $O\underline{CH_2}CH_2CH_2CH_2CH_2CH_2CH_3$), 3.86 (s, 3H, $O\underline{CH_3}$), 2.40 (s, 3H, -$\underline{CH_3}$), 1.70 (p, *J* = 6.5 Hz, 2H, $OCH_2\underline{CH_2}CH_2CH_2CH_2CH_2CH_3$), 1.48 – 1.37 (m, 2H, $OCH_2CH_2\underline{CH_2}CH_2\,CH_2CH_2CH_3$), 1.36 – 1.19 (m, 6H, $OCH_2CH_2CH_2\underline{CH_2CH_2CH_2}CH_3$), 0.86 (t, *J* = 6.6 Hz, 3H, $OCH_2CH_2CH_2CH_2CH_2CH_2\underline{CH_3}$).

δ_C/ppm (100 MHz, DMSO-d_6): 167.24, 163.54, 159.92, 159.21, 154.66, 146.23, 132.33, 132.15, 121.38, 119.40, 115.58, 114.03, 113.83, 108.08, 69.02, 56.33, 31.73, 28.97, 28.80, 25.76, 22.50, 22.02, 14.44.

10.3 2-Alkyloxy-4-(2,4-dimethoxybenzoyl)oxybenzoic acids.

Tables SI14. Quantities of reagents used to synthesise 2-alkyloxy-4-(2,4-dimethoxybenzoyl)oxybenzoic acids.

m	(**9.3**)	$H_2NaO_4P.H_2O$	$ClNaO_2$	Resorcinol
2	0.258 g, 7.81 ×10^{-4} mol	0.431 g, 3.12×10^{-3} mol	0.247 g, 2.73×10^{-3} mol	-
3	0.400 g, 1.07×10^{-3} mol	0.590 g, 4.28×10^{-3} mol	0.338 g, 3.74×10^{-3} mol	-
4	0.800 g, 2.23×10^{-3} mol	1.232 g, 8.93×10^{-3} mol	0.705 g, 7.81×10^{-3} mol	-
5	1.900 g, 5.10×10^{-3} mol	3.631 g, 2.04×10^{-2} mol	1.612 g, 1.79×10^{-2} mol	0.843 g, 7.66×10^{-3} mol
6	0.976 g, 2.53×10^{-3} mol	1.801 g, 10.1×10^{-2} mol	0.801 g, 8.85×10^{-3} mol	0.412 g, 3.79×10^{-3} mol
7	1.300 g, 3.25×10^{-3} mol	2.310 g, 1.30×10^{-2} mol	1.031 g, 1.14×10^{-2} mol	0.536 g, 4.87×10^{-3} mol

10.3.1 2-Ethoxy-4-(2,4-dimethoxybenzoyl)oxybenzoic acid

White powder. Yield: 0.081 g, 30%. RF: 0 (20% ethyl acetate:80% hexane). M.P. = 160 ˚C

ν_{max}/cm^{-1}: 2974 (-CH_3), 2850 (-CH_2), 1738 (COOR), 1668 (COOH dimer), 1607 (Ar C=C), 1233 (C-O-C).

δ_H/ppm (400 MHz, DMSO-d_6): 12.53 (s, 1H, COOH), 7.97 (d, *J* = 9.0 Hz, 1H, Ar-H), 7.69 (d, *J* = 8.4 Hz, 1H, Ar-H), 6.99 (d, *J* = 2.1 Hz, 1H, Ar-H), 6.83 (dd, *J* = 8.4, 2.1 Hz, 1H, Ar-H), 6.71 (d, *J* = 2.5 Hz, 1H, Ar-H), 6.68 (dd, *J* = 8.8, 2.8 Hz, 1H, Ar-H), 4.09 (q, *J* = 7.0 Hz, 2H, $O\underline{CH_2}CH_3$), 3.93 – 3.85 (m, 6H, 2× $O\underline{CH_3}$), 1.32 (t, *J* = 7.0 Hz, 3H, $OCH_2\underline{CH_3}$).

δ_C/ppm (100 MHz, DMSO-d_6): 166.79, 164.90, 162.47, 161.74, 158.53, 157.15, 154.31, 134.06, 131.66, 113.72, 109.98, 107.76, 105.69, 99.00, 64.37, 56.01, 55.74, 14.49.

10.3.2 2-Propoxy-4-(2,4-dimethoxybenzoyl)oxybenzoic acid

White powder. Yield: 0.188 g, 46%. RF: 0 (20% ethyl acetate:80% hexane). M.P. = 156 ˚C

ν_{max}/cm^{-1}: 2958 (-CH_3), 2849 (-CH_2), 1740 (COOR), 1671 (COOH dimer), 1608 (Ar C=C), 1570, 1449, 1304, 1233 (C-O-C), 1206, 1174, 1133, 1014, 866, 825, 758, 664, 531, 473.

δ_H/ppm (400 MHz, DMSO-d_6): 12.50 (s, 1H, COOH), 7.97 (d, *J* = 8.7 Hz, 1H, Ar-H), 7.70 (d, *J* = 8.4 Hz, 1H, Ar-H), 6.99 (d, *J* = 2.1 Hz, 1H, Ar-H), 6.82 (dd, *J* = 8.4, 2.1 Hz, 1H, Ar-H), 6.71 (d, *J* = 2.4 Hz, 1H, Ar-H), 6.67 (dd, *J* = 8.8, 2.4 Hz, 1H, Ar-H), 3.98 (t, *J* = 6.3 Hz, 2H, $O\underline{CH_2}CH_2CH_3$), 3.93 – 3.85 (m, 6H, 2× $O\underline{CH_3}$), 1.72 (h, *J* = 7.1 Hz, 2H, $OCH_2\underline{CH_2}CH_3$), 0.99 (t, *J* = 7.4 Hz, 3H, $OCH_2CH_2\underline{CH_3}$).

δ_C/ppm (100 MHz, DMSO-d_6): 166.83, 164.89, 162.47, 161.73, 158.71, 157.15, 154.31, 134.05, 131.67, 113.64, 109.98, 107.67, 105.68, 99.00, 70.00, 56.01, 55.74, 21.95, 10.39.

10.3.3 2-Butoxy-4-(2,4-dimethoxybenzoyl)oxybenzoic acid

White powder. Yield: 0.556 g, 66%. RF: 0 (20% ethyl acetate:80% hexane). M.P. = 114 ˚C

ν_{max}/cm^{-1}: 2944 (-CH_3), 2875 (-CH_2), 1741 (COOR), 1665 (COOH dimer), 1608 (Ar C=C), 1570, 1505, 1451, 1234 (C-O-C), 1205, 1156, 1009, 877, 830, 760, 662, 531, 461.

δ_H/ppm (400 MHz, DMSO-d_6): 12.42 (s, 1H, -COOH), 7.97 (d, *J* = 8.7 Hz, 1H, Ar-H), 7.68 (d, *J* = 8.4 Hz, 1H, Ar-H), 6.99 (d, *J* = 2.1 Hz, 1H, Ar-H), 6.82 (dd, *J* = 8.4, 2.1 Hz, 1H, Ar-H), 6.71 (d, *J* = 2.3 Hz, 1H, Ar-H), 6.67 (dd, *J* = 8.8, 2.3 Hz, 1H, Ar-H), 4.02 (t, *J* = 6.4 Hz, 2H, $O\underline{CH_2}CH_2CH_2CH_3$), 3.94 – 3.86 (m, 6H, 2× $O\underline{CH_3}$), 1.69 (p, *J* = 8.4 Hz, 2H, $OCH_2\underline{CH_2}CH_2CH_3$), 1.46 (h, *J* = 7.9 Hz, 2H, $OCH_2CH_2\underline{CH_2}CH_3$), 0.92 (t, *J* = 7.4 Hz, 3H, $OCH_2CH_2CH_2\underline{CH_3}$).

δ_C/ppm (100 MHz, DMSO-d_6): 166.86, 164.87, 162.46, 161.72, 158.68, 154.26, 134.04, 131.59, 113.61, 109.97, 107.65, 105.66, 98.99, 68.23, 55.99, 55.72, 30.60, 18.60, 13.64.

10.3.4 2-Pentoxy-4-(2,4-dimethoxybenzoyl)oxybenzoic acid

White powder. Yield: 1.78 g, 97%. RF: 0.01 (20% ethyl acetate:80% hexane). M.P. = 104 ˚C

ν_{max}/cm^{-1}: 2952 (-CH_3), 2868 (-CH_2), 1749 (COOR), 1662 (COOH dimer), 1601 (Ar C=C), 1235 (C-O-C).

δ_H/ppm (400 MHz, DMSO-d_6): 12.48 (s, 1H, COOH), 7.96 (d, *J* = 8.7 Hz, 1H, Ar-H), 7.69 (d, *J* = 8.4 Hz, 1H, Ar-H), 6.99 (d, *J* = 2.1 Hz, 1H, Ar-H), 6.82 (dd, *J* = 8.4, 2.1 Hz, 1H, Ar-H), 6.71 (d, *J* = 2.4 Hz, 1H, Ar-H), 6.67 (dd, *J* = 8.8, 2.3 Hz, 1H, Ar-H), 4.01 (t, *J* = 6.4 Hz, 2H, $O\underline{CH_2}CH_2CH_2CH_2CH_3$), 3.95 – 3.85 (m, 6H,

2× $O\underline{CH_3}$), 1.71 (p, *J* = 6.7 Hz, 2H, $OCH_2\underline{CH_2}CH_2CH_2CH_3$), 1.48 – 1.26 (m, 4H, $OCH_2CH_2\underline{CH_2}\underline{CH_2}CH_3$), 0.89 (t, *J* = 7.2 Hz, 3H, $OCH_2CH_2CH_2CH_2\underline{CH_3}$).

δ_C/ppm (100 MHz, DMSO-d_6): 166.84, 164.88, 162.47, 161.72, 158.68, 154.27, 134.04, 131.59, 113.63, 109.98, 107.68, 105.67, 98.99, 68.54, 56.00, 55.73, 28.19, 27.55, 21.77, 13.93.

10.3.5 2-Hexyloxy-4-(2,4-dimethoxybenzoyl)oxybenzoic acid

White powder. Yield: 0.721 g, 71%. RF: 0.02 (20% ethyl acetate:80% hexane). M.P. = 100 ˚C

ν_{max}/cm^{-1}: 2949 ($-CH_3$), 2854 ($-CH_2$), 1741 (COOR), 1665 (COOH dimer), 1602 (Ar C=C), 1235 (C-O-C).

δ_H/ppm (400 MHz, DMSO-d_6): 12.52 (s, 1H, COOH), 7.96 (d, *J* = 8.7 Hz, 1H, Ar-H), 7.69 (d, *J* = 8.4 Hz, 1H, Ar-H), 6.99 (d, *J* = 2.1 Hz, 1H, Ar-H), 6.82 (dd, *J* = 8.4, 2.1 Hz, 1H, Ar-H), 6.71 (d, *J* = 2.3 Hz, 1H, Ar-H), 6.69 (dd, *J* = 8.7, 2.1 Hz, 1H, Ar-H), 4.01 (t, *J* = 6.4 Hz, 2H, $O\underline{CH_2}CH_2CH_2CH_2CH_2CH_3$), 3.92 – 3.84 (m, 6H, 2× $O\underline{CH_3}$), 1.70 (p, *J* = 6.5 Hz, 2H, $OCH_2\underline{CH_2}CH_2CH_2CH_2CH_3$), 1.50 – 1.38 (m, 2H, $OCH_2CH_2\underline{CH_2}CH_2CH_2CH_3$), 1.36 – 1.23 (m, 4H, $OCH_2CH_2CH_2\underline{CH_2}\underline{CH_2}CH_3$), 0.87 (t, *J* = 5.5 Hz, 3H, $OCH_2CH_2CH_2CH_2CH_2\underline{CH_3}$).

δ_C/ppm (100 MHz, DMSO-d_6): 166.78, 164.87, 162.45, 161.72, 158.72, 154.33, 134.04, 131.64, 113.62, 109.96, 107.67, 105.66, 98.99, 68.53, 55.99, 55.72, 30.85, 28.45, 24.98, 22.06, 13.88.

10.3.6 2-Heptyloxy-4-(2,4-dimethoxybenzoyl)oxybenzoic acid

White powder. Yield: 1.09 g, 80%. RF: 0.1 (20% ethyl acetate:80% hexane). M.P. = 116 ˚C

ν_{max}/cm^{-1}: 2922 ($-CH_3$), 2850 ($-CH_2$), 1742 (COOR), 1665 (COOH dimer), 1607 (Ar C=C), 1232 (C-O-C).

δ_H/ppm (400 MHz, DMSO-d_6): 12.48 (s, 1H, COOH), 7.96 (d, *J* = 8.8 Hz, 1H, Ar-H), 7.69 (d, *J* = 8.4 Hz, 1H, Ar-H), 6.99 (d, *J* = 2.1 Hz, 1H, Ar-H), 6.82 (dd, *J* = 8.4, 2.1 Hz, 1H, Ar-H), 6.71 (d, *J* = 2.3 Hz, 1H, Ar-H), 6.67 (dd, *J* = 8.8, 2.3 Hz, 1H, Ar-H), 4.01 (t, *J* = 6.4 Hz, 2H, $O\underline{CH_2}CH_2CH_2CH_2CH_2CH_2CH_3$), 3.92 – 3.85 (m, 6H, 2× $O\underline{CH_3}$), 1.70 (p, *J* = 6.4 Hz, 2H, $OCH_2\underline{CH_2}CH_2CH_2CH_2CH_2CH_3$), 1.49 – 1.37 (m, 2H, $OCH_2CH_2\underline{CH_2}CH_2CH_2CH_2CH_3$), 1.36 – 1.20 (m, 6H, $OCH_2CH_2CH_2\underline{CH_2}\underline{CH_2}\underline{CH_2}CH_3$), 0.90 – 0.82 (m, 3H, $OCH_2CH_2CH_2CH_2CH_2CH_2\underline{CH_3}$).

δ_C/ppm (100 MHz, DMSO-d_6): 166.82, 164.88, 162.47, 161.72, 158.69, 154.26, 134.04, 131.60, 113.61, 109.98, 107.67, 105.67, 98.99, 68.53, 56.00, 55.73, 31.26, 28.51, 28.33, 25.30, 22.03, 13.97.

Synthesis of Compound 11

Method 1: Under inert conditions, a mixture of **Compound 10** (2 eq) and DCC (1.5 eq) were dissolved in dichloromethane (10 ml) on ice and allowed to react for 30 min. The required 4-nitrophenol or 3-fluoro-4-nitrophenol (1.5 eq) was dissolved in dry dichloromethane (2 mL), added to the solution and allowed to react overnight slowly warming up to room temperature. The quantities of reagents used in the reaction are listed in **Tables S15-18**. The reaction mixture is then quenched by filtration of the precipitated DCU and then purified by flash column chromatography with dichloromethane. The product was then purified by hot recrystallization in ethanol.

11.1 (4-Nitrophenyl)-2-alkyloxy-4-(4-methylbenzoyl)oxybenzoates

Tables SI15. Quantities of reagents used to synthesise (4-nitrophenyl)-2-alkoxy-4-(4-methyl benzoyl)oxybenzoates.

m	(**10.1**)	4-Nitrophenol	DCC
2	0.250 g, $7.57{\times}10^{-4}$ mol	0.079 g, $5.67{\times}10^{-4}$ mol	0.117 g, $5.67{\times}10^{-4}$ mol
3	0.300 g, $9.55{\times}10^{-4}$ mol	0.099 g, $7.16{\times}10^{-4}$ mol	0.147 g, $7.16{\times}10^{-4}$ mol
4	0.140 g, $3.88{\times}10^{-4}$ mol	0.054 g, $3.88{\times}10^{-4}$ mol	0.100 g, $4.85{\times}10^{-4}$ mol
5	0.250 g, $7.30{\times}10^{-4}$ mol	0.076 g, $5.48{\times}10^{-4}$ mol	0.113 g, $5.48{\times}10^{-4}$ mol
6	0.200 g, $5.61{\times}10^{-4}$ mol	0.071 g, $5.10{\times}10^{-4}$ mol	0.132 g, $6.38{\times}10^{-4}$ mol
7	0.200 g, $5.39{\times}10^{-4}$ mol	0.068 g, $4.91{\times}10^{-4}$ mol	0.127 g, $6.14{\times}10^{-4}$ mol

11.1.1 (4-Nitrophenyl)-2-ethoxy-4-(4-methylbenzoyl)oxybenzoate (C.2)

White powder. Yield: 0.050 g, 15%. RF: 0.36 (100% dichloromethane)

T_{CrI} 146 ˚C T_{N_FI} (129 ˚C)

ν_{max}/cm^{-1}: 3093 (Aryl-H), 2989 (-CH_3), 2935 (-CH_2), 1750, 1734 (COOR), 1610 (Ar C=C), 1517 (NO_2), 1261 (C-O-C), 1206 (C-F).

δ_H/ppm (400 MHz, DMSO-d$_6$): 8.36 (d, *J* = 8.9 Hz, 2H, Ar-H), 8.10 – 8.02 (m, 3H, Ar-H), 7.59 (d, *J* = 8.9 Hz, 2H, Ar-H), 7.44 (d, *J* = 7.9 Hz, 2H, Ar-H), 7.25 (d, *J* = 2.1 Hz, 1H, Ar-H), 7.06 (dd, *J* = 8.6, 1.3 Hz, 1H, Ar-H), 4.17 (q, *J* = 6.9 Hz, 2H, O$\underline{CH_2}$$CH_3$), 2.44 (s, 3H, -$\underline{CH_3}$), 1.36 (t, *J* = 6.9 Hz, 3H, $OCH_2$$\underline{CH_3}$).

δ_C/ppm (100 MHz, DMSO-d$_6$): 164.05, 162.47, 159.96, 155.81, 155.57, 145.07, 144.86, 133.06, 130.01, 129.61, 125.83, 125.36, 123.32, 115.44, 114.02, 108.09, 64.76, 21.31, 14.42.

MS = $[M+H]^+$ Calculated mass for $C_{23}H_{20}NO_7$: 422.1240. Found: 422.1245. Diff: 1.2 ppm.

11.1.2 (4-Nitrophenyl)-2-propoxy-4-(4-methylbenzoyl)oxybenzoate (C.3)

White powder. Yield: 0.168 g, 55%. RF: 0.40 (100% dichloromethane)

T_{CrI} 161 ˚C $T_{N_F I}$ (106 ˚C)

v_{max}/cm^{-1}: 3115 (Aryl-H), 2962 (-CH_3), 2945 (-CH_2), 2830 (-CH_2), 1738 (COOR), 1601 (Ar C=C), 1519 (NO_2), 1259 (C-O-C), 1170 (C-F).

δ_H/ppm (400 MHz, DMSO-d_6): 8.32 (d, *J* = 8.9 Hz, 2H, Ar-H), 8.14 – 8.06 (m, 3H, Ar-H), 7.41 (d, *J* = 8.6 Hz, 2H, Ar-H), 7.34 (d, *J* = 8.0 Hz, 2H, Ar-H), 6.96 – 6.90 (m, 2H, Ar-H), 4.06 (t, *J* = 6.4 Hz, 2H, $O\underline{CH_2}CH_2CH_3$), 2.47 (s, 3H, -$\underline{CH_3}$), 1.88 (h, *J* = 7.0 Hz, 2H, $OCH_2\underline{CH_2}CH_3$), 1.06 (t, *J* = 7.4 Hz, 3H, $OCH_2CH_2\underline{CH_3}$).

δ_C/ppm (100 MHz, DMSO-d_6): 164.69, 162.93, 161.25, 156.52, 156.06, 145.40, 145.16, 133.90, 130.47, 129.60, 126.31, 125.39, 122.84, 115.41, 113.75, 107.31, 70.89, 22.60, 21.97, 10.71.

MS = $[M+H]^+$ Calculated mass for $C_{24}H_{22}NO_7$: 436.1396. Found: 436.1376. Diff: 4.6 ppm.

11.1.3 (4-Nitrophenyl)-2-butoxy-4-(4-methylbenzoyl)oxybenzoate (C.4)

White powder. Yield: 0.043 g, 24%. RF: 0.60 (100% dichloromethane)

T_{CrI} 134 ˚C $T_{N_F I}$ (88 ˚C)

v_{max}/cm^{-1}: 3093 (Aryl-H), 2970 (-CH_3), 2950 (-CH_2), 2880 (-CH_2), 1740 (COOR), 1610 (Ar C=C), 1522 (NO_2), 1256 (C-O-C), 1196 (C-F).

δ_H/ppm (400 MHz, DMSO-d_6): 8.36 (d, *J* = 8.9 Hz, 2H, Ar-H), 8.08 – 8.02 (m, 3H, Ar-H), 7.56 (d, *J* = 8.9 Hz, 2H, Ar-H), 7.44 (d, *J* = 8.0 Hz, 2H, Ar-H), 7.25 (d, *J* = 2.1 Hz, 1H, Ar-H), 7.05 (dd, *J* = 8.6, 2.1 Hz, 1H, Ar-H), 4.10 (t, *J* = 6.3 Hz, 2H, $O\underline{CH_2}CH_2CH_2CH_3$), 2.43 (s, 3H, -$\underline{CH_3}$), 1.71 (p, *J* = 6.7 Hz, 2H, $OCH_2\underline{CH_2}CH_2CH_3$), 1.45 (h, *J* = 7.5 Hz, 2H, $OCH_2CH_2\underline{CH_2}CH_3$), 0.89 (t, *J* = 7.4 Hz, 3H, $OCH_2CH_2CH_2\underline{CH_3}$).

δ_C/ppm (100 MHz, DMSO-d_6): 164.17, 162.63, 160.24, 155.95, 155.66, 145.15, 144.97, 133.23, 130.08, 129.69, 125.89, 125.46, 123.36, 115.35, 114.06, 108.04, 68.65, 30.57, 21.38, 18.67, 13.64.

MS = $[M+H]^+$ Calculated mass for $C_{25}H_{24}NO_7$: 450.1553. Found: 450.1555. Diff: 0.4 ppm.

11.1.4 (4-Nitrophenyl)-2-pentoxy-4-(4-methylbenzoyl)oxybenzoate (C.5)

White powder. Yield: 0.027 g, 15%. RF: 0.63 (100% dichloromethane)

T_{CrI} 110 ˚C $T_{N_F I}$ (73 ˚C)

v_{max}/cm^{-1}: 3117 (Aryl-H), 2952 (-CH_3), 2872 (-CH_2), 1737 (COOR), 1604 (Ar C=C), 1518 (NO_2), 1347, 1258 (C-O-C), 1199 (C-F).

δ_H/ppm (400 MHz, DMSO-d_6): 8.36 (d, *J* = 9.2 Hz, 2H, Ar-H), 8.09 – 8.02 (m, 3H, Ar-H), 7.57 (d, *J* = 9.1 Hz, 2H, Ar-H), 7.44 (d, *J* = 8.0 Hz, 2H, Ar-H), 7.26 (d, *J* = 2.1 Hz, 1H, Ar-H), 7.05 (dd, *J* = 8.5, 2.1 Hz, 1H, Ar-H), 4.10 (t, *J* = 6.3 Hz, 2H, $O\underline{CH_2}CH_2CH_2CH_2CH_3$), 2.44 (s, 3H, -$\underline{CH_3}$), 1.74 (p, *J* = 6.5 Hz, 2H, $OCH_2\underline{CH_2}CH_2CH_2CH_3$), 1.41 (p, *J* = 6.6 Hz, 2H, $OCH_2CH_2\underline{CH_2}CH_2CH_3$), 1.30 (h, *J* = 6.9 Hz, 2H, $OCH_2CH_2CH_2\underline{CH_2}CH_3$), 0.82 (t, *J* = 7.3 Hz, 3H, $OCH_2CH_2CH_2CH_2\underline{CH_3}$).

δ_C/ppm (100 MHz, DMSO-d_6): 164.05, 162.60, 160.12, 155.86, 155.60, 145.06, 144.85, 133.14, 130.00, 129.60, 125.83, 125.37, 123.26, 115.31, 113.98, 107.99, 68.85, 28.13, 27.56, 21.72, 21.30, 13.87.

MS = $[M+H]^+$ Calculated mass for $C_{26}H_{26}NO_7$: 464.1709. Found: 464.1716. Diff: 1.5 ppm.

11.1.5 (4-Nitrophenyl)-2-hexyloxy-4-(4-methylbenzoyl)oxybenzoate (C.6)

White powder. Yield: 0.105 g, 50%. RF: 0.73 (100% dichloromethane)

T_{CrI} 90 ˚C T_{N_FI} (65 ˚C)

ν_{max}/cm^{-1}: 3117 (Aryl-H), 29652 (-CH_3), 2870 (-CH_2), 1741 (COOR), 1604 (Ar C=C), 1526 (NO_2), 1348, 1257 (C-O-C), 1201 (C-F).

δ_H/ppm (400 MHz, DMSO-d_6): 8.36 (d, *J* = 9.2 Hz, 2H, Ar-H), 8.09 – 8.02 (m, 3H, Ar-H), 7.57 (d, *J* = 9.0 Hz, 2H, Ar-H), 7.44 (d, *J* = 8.0 Hz, 2H, Ar-H), 7.26 (d, *J* = 2.1 Hz, 1H, Ar-H), 7.05 (dd, *J* = 8.5, 2.1 Hz, 1H, Ar-H), 4.10 (t, *J* = 6.3 Hz, 2H, $O\underline{CH_2}CH_2CH_2CH_2CH_2CH_3$), 2.44 (s, 3H, -$\underline{CH_3}$), 1.73 (p, *J* = 6.5 Hz, 2H, $OCH_2\underline{CH_2}CH_2CH_2CH_2CH_3$), 1.42 (p, *J* = 7.3 Hz, 2H, $OCH_2CH_2\underline{CH_2}CH_2CH_2CH_3$), 1.32 – 1.15 (m, 4H, $OCH_2CH_2CH_2\underline{CH_2CH_2}CH_3$), 0.80 (t, *J* = 7.0 Hz, 3H, $OCH_2CH_2CH_2CH_2CH_2\underline{CH_3}$).

δ_C/ppm (100 MHz, DMSO-d_6): 164.08, 162.67, 160.12, 155.88, 155.62, 145.07, 144.88, 133.15, 130.02, 129.62, 125.85, 125.38, 123.27, 115.36, 114.00, 108.01, 68.89, 30.84, 28.43, 25.06, 22.05, 21.32, 13.83.

EA: Calculated: C 67.91%, H 5.70%, N 2.93%. Found: C 68.09%, H 5.27 %, N 2.84%.

11.1.6 (4-Nitrophenyl)-2-heptyloxy-4-(4-methylbenzoyl)oxybenzoate (C.7)

White powder. Yield: 0.039 g, 16%. RF: 0.50 (100% dichloromethane)

T_{CrI} 82 ˚C T_{N_FI} (60 ˚C)

ν_{max}/cm^{-1}: 3110 (Aryl-H), 2927 (-CH_3), 2858 (-CH_2), 1742 (COOR), 1605 (Ar C=C), 1524 (NO_2), 1258 (C-O-C), 1201 (C-F).

δ_H/ppm (400 MHz, DMSO-d_6): 8.36 (d, *J* = 8.7 Hz, 2H, Ar-H), 8.08 – 8.01 (m, 3H, Ar-H), 7.57 (d, *J* = 9.0 Hz, 2H, Ar-H), 7.44 (d, *J* = 8.2 Hz, 2H, Ar-H), 7.25 (d, *J* = 2.1 Hz, 1H, Ar-H), 7.05 (dd, *J* = 8.5, 2.1 Hz, 1H, Ar-H), 4.10 (t, *J* = 6.2 Hz, 2H, $O\underline{CH_2}CH_2CH_2CH_2CH_2CH_2CH_3$), 2.44 (s, 3H, -$\underline{CH_3}$), 1.72 (p, *J* = 6.4 Hz, 2H, $OCH_2\underline{CH_2}CH_2CH_2CH_2CH_2CH_3$), 1.41 (p, *J* = 7.2 Hz, 2H, $OCH_2CH_2\underline{CH_2}CH_2CH_2CH_2CH_3$), 1.32 – 1.12 (m, 6H, $OCH_2CH_2CH_2\underline{CH_2CH_2\ CH_2}CH_3$), 0.79 (t, *J* = 6.8 Hz, 2H, $OCH_2CH_2CH_2CH_2CH_2CH_2\underline{CH_3}$).

δ_C/ppm (100 MHz, DMSO-d_6): 164.09, 162.73, 160.12, 155.88, 155.64, 145.06, 144.89, 133.16, 130.02, 129.63, 125.85, 125.39, 123.26, 115.38, 114.02, 108.01, 68.89, 31.23, 28.50, 28.33, 25.38, 22.00, 21.32, 13.92.

MS = $[M+H]^+$ Calculated mass for $C_{28}H_{30}NO_7$: 492.2022. Found: 492.2037. Diff: 3.0 ppm.

11.2 (3-Fluoro-4-nitrophenyl)-2-alkyloxy-4-(4-methylbenzoyl)oxybenzoates

Tables SI16. Quantities of reagents used to synthesise (3-fluoro-4-nitrophenyl)-2-alkoxy-4-(4-methylbenzoyl)oxybenzoates.

m	(**10.1**)	3-Fluoro-4-nitrophenol	DCC
2	0.250 g, 7.58×10^{-4} mol	0.089 g, 5.67×10^{-4} mol	0.117 g, 5.67×10^{-4} mol
3	0.300 g, 9.55×10^{-4} mol	0.113 g, 7.16×10^{-4} mol	0.147 g, 7.16×10^{-4} mol
4	0.150 g, 4.56×10^{-4} mol	0.054 g, 3.42×10^{-4} mol	0.071 g, 3.42×10^{-4} mol
5	0.250 g, 7.30×10^{-4} mol	0.086 g, 5.48×10^{-4} mol	0.113 g, 5.48×10^{-4} mol
6	0.200 g, 5.61×10^{-4} mol	0.066 g, 4.21×10^{-4} mol	0.087 g, 4.21×10^{-4} mol
7	0.250 g, 6.75×10^{-4} mol	0.079 g, 5.06×10^{-4} mol	0.104 g, 5.06×10^{-3} mol

11.2.1 (3-Fluoro-4-nitrophenyl)-2-ethoxy-4-(4-methylbenzoyl)oxybenzoate (D.2)

White powder. Yield: 0.150 g, 45%. RF: 0.36 (100% dichloromethane)

T_{CrI} 145 ˚C T_{N_FI} (128 ˚C)

ν_{max}/cm^{-1}: 2988 (Aryl-H), 2970 ($-CH_3$), 2932 ($-CH_2$), 2840 ($-CH_2$), 1740 (COOR), 1607 (Ar C=C), 1527 (NO_2), 1260 (C-O-C), 1212 (C-F).

δ_H/ppm (400 MHz, DMSO-d_6): 8.31 (t, *J* = 8.9 Hz, 1H, Ar-H), 8.11 – 8.00 (m, 3H, Ar-H), 7.71 (dd, *J* = 12.0, 2.4 Hz, 1H, Ar-H), 7.44 (d, *J* = 8.1 Hz, 3H, Ar-H), 7.41 (dd, *J* = 2.4, 1.2 Hz, 1H, Ar-H), 7.26 (d, *J* = 2.1 Hz, 1H, Ar-H), 7.06 (dd, *J* = 8.6, 2.1 Hz, 1H, Ar-H), 4.17 (q, *J* = 6.9 Hz, 2H, $O\underline{CH_2}CH_3$), 2.44 (s, 3H, , -$\underline{CH_3}$), 1.35 (t, *J* = 6.9 Hz, 3H, $OCH_2\underline{CH_3}$).

δ_C/ppm (100 MHz, DMSO-d_6): 164.03, 161.86, 160.19, 156.60 (d, *J* = 262.5 Hz), 156.01, 155.62 (d, *J* = 11.5 Hz), 144.88, 134.54 (d, *J* = 7.0 Hz), 133.28, 130.01, 129.61, 127.56 (d, *J* = 2.5 Hz), 125.81, 119.16 (d, *J* = 3.9 Hz), 114.91, 114.03, 112.74 (d, *J* = 23.7 Hz), 108.11, 64.78, 21.31, 14.39.

MS = $[M+H]^+$ Calculated mass for $C_{23}H_{19}NO_7F$: 440.1146. Found: 440.1124. Diff: 5 ppm.

MS = $[2M+Na]^+$ Calculated mass for $C_{46}H_{36}N_2O_{14}F_2Na$: 901.2032. Found: 901.2048. Diff: 1.8 ppm.

11.2.2 (3-Fluoro-4-nitrophenyl)-2-propoxy-4-(4-methylbenzoyl)oxybenzoate (D.3)

White powder. Yield: 0.181 g, 56%. RF: 0.58 (100% dichloromethane)

T_{CrI} 136 ˚C T_{N_FI} (109 ˚C)

ν_{max}/cm^{-1}: 3087 (Aryl-H), 2960 ($-CH_3$), 2944 ($-CH_2$), 2830 ($-CH_2$), 1732 (COOR), 1601 (Ar C=C), 1526 (NO_2), 1250 (C-O-C), 1216 (C-F).

δ_H/ppm (400 MHz, DMSO-d_6): 8.18 (t, *J* = 8.7 Hz, 1H, Ar-H), 8.13 – 8.05 (m, 3H, Ar-H), 7.34 (d, *J* = 8.0 Hz, 2H, Ar-H), 7.29 (dd, *J* = 9.5, 2.4 Hz, 1H, Ar-H), 7.21 (ddd, *J* = 9.0, 2.5, 1.4 Hz, 1H, Ar-H), 6.93 (d, *J* = 7.7 Hz, 2H, Ar-H), 4.06 (t, *J* = 6.4 Hz, 2H, $O\underline{CH_2}CH_2CH_3$), 2.47 (s, 3H, $-CH_3$), 1.88 (h, *J* = 7.0 Hz, 2H, $OCH_2\underline{CH_2}CH_3$), 1.07 (t, *J* = 7.4 Hz, 3H, $OCH_2CH_2\underline{CH_3}$).

δ_C/ppm (100 MHz, DMSO-d$_6$): 164.65, 162.37, 161.38, 157.71 (d, *J* = 266.9 Hz), 156.75, 156.19 (d, *J* = 10.4 Hz), 145.21, 134.74 (d, *J* = 7.3 Hz), 133.96, 130.47, 129.61, 127.30 (d, *J* = 2.1 Hz), 126.25, 118.29 (d, *J* = 4.0 Hz), 114.88, 113.81, 112.53 (d, *J* = 23.8 Hz), 107.32, 70.90, 22.58, 21.97, 10.69.

MS = $[M+H]^+$ Calculated mass for $C_{24}H_{21}NO_7F$: 454.1302. Found: 454.1318. Diff: 3.5 ppm.

11.2.3 (3-Fluoro-4-nitrophenyl)-2-butoxy-4-(4-methylbenzoyl)oxybenzoate (D.4)

White powder. Yield: 0.080 g, 37%. RF: 0.58 (100% dichloromethane)

T_{CrI} 133 ˚C T_{N_FI} (93 ˚C)

ν_{max}/cm^{-1}: 2959 (-CH_3), 2932 (-CH_2), 2870 (-CH_2), 1732 (COOR), 1601 (Ar C=C), 1524 (NO_2), 1251 (C-O-C), 1216 (C-F).

δ_H/ppm (400 MHz, DMSO-d$_6$): 8.31 (t, *J* = 8.9 Hz, 1H, Ar-H), 8.10 – 8.02 (m, 3H, Ar-H), 7.70 (dt, *J* = 11.9, 1.6 Hz, 1H, Ar-H), 7.47 – 7.37 (m, 3H, Ar-H), 7.29 – 7.24 (m, 1H, Ar-H), 7.06 (dd, *J* = 8.7, 2.0 Hz, 1H, Ar-H), 4.11 (t, *J* = 6.3 Hz, 2H, $O\underline{CH_2}CH_2CH_2CH_3$), 2.44 (s, 3H, -$CH_3$), 1.72 (p, *J* = 6.5 Hz, 2H, $OCH_2\underline{CH_2}CH_2CH_2CH_3$), 1.46 (h, *J* = 7.5 Hz, 2H, $OCH_2\,CH_2\underline{CH_2}CH_2CH_3$), 0.90 (t, *J* = 7.4 Hz, 3H, $OCH_2CH_2CH_2CH_2\underline{CH_3}$).

δ_C/ppm (100 MHz, DMSO-d$_6$): 164.03, 161.91, 160.40, 156.61 (d, *J* = 263.4 Hz), 156.07, 155.71 (d, *J* = 11.4 Hz), 144.87, 134.58 (d, *J* = 7.1 Hz), 133.35, 130.00, 129.60, 127.58 (d, *J* = 1.9 Hz), 125.81, 119.15 (d, *J* = 3.8 Hz), 114.76, 113.98, 112.71 (d, *J* = 23.8 Hz), 108.00, 68.59, 30.49, 21.30, 18.59, 13.57.

MS = $[M+H]^+$ Calculated mass for $C_{25}H_{23}NO_7F$: 468.1459. Found: 468.1479. Diff: 4.3 ppm.

11.2.4 (3-Fluoro-4-nitrophenyl)-2-pentoxy-4-(4-methylbenzoyl)oxybenzoate (D.5)

White powder. Yield: 0.138 g, 78%. RF: 0.58 (100% dichloromethane)

T_{CrI} 134 ˚C T_{N_FI} (81 ˚C)

ν_{max}/cm^{-1}: 2958 (-CH_3), 2856 (-CH_2), 1740 (COOR), 1602 (Ar C=C), 1526 (NO_2), 1225 (C-O-C), 1212 (C-F).

δ_H/ppm (400 MHz, DMSO-d$_6$): 8.31 (t, *J* = 8.9 Hz, 1H, Ar-H), 8.04 – 8.03 (m, 3H, Ar-H), 7.69 (dd, *J* = 12.0, 2.4 Hz, 1H, Ar-H), 7.47 – 7.37 (m, 3H, Ar-H), 7.26 (d, *J* = 2.2 Hz, 1H, Ar-H), 7.05 (dd, *J* = 8.6, 2.1 Hz, 1H, Ar-H), 4.09 (t, *J* = 6.3 Hz, 2H, $O\underline{CH_2}CH_2CH_2CH_2CH_3$), 2.44 (s, 3H, -$CH_3$), 1.73 (p, *J* = 6.5 Hz, 2H, $OCH_2\underline{CH_2}CH_2CH_2CH_3$), 1.41 (p, *J* = 6.7 Hz, 2H, $OCH_2CH_2\underline{CH_2}CH_2CH_3$), 1.31 (h, *J* = 7.2 Hz, 2H, $OCH_2CH_2CH_2\underline{CH_2}CH_3$), 0.83 (t, *J* = 7.2 Hz, 3H, $OCH_2CH_2CH_2CH_2\underline{CH_3}$).

δ_C/ppm (100 MHz, DMSO-d$_6$): 164.10, 162.12, 160.38, 156.65 (d, *J* = 264.5 Hz), 156.11, 155.70 (d, *J* = 11.5 Hz), 144.94, 134.65 (d, *J* = 7.6 Hz), 133.39, 130.06, 129.66, 127.62 (d, *J* = 2.3 Hz), 125.85, 119.15 (d, *J* = 3.5 Hz), 114.88, 114.05, 112.71 (d, *J* = 23.8 Hz), 108.05, 68.96, 28.45, 25.10, 22.09, 21.35, 13.86.

MS = $[M+H]^+$ Calculated mass for $C_{26}H_{25}NO_7F$: 482.1615. Found: 482.1611. Diff: 0.8 ppm.

11.2.5 (3-Fluoro-4-nitrophenyl)-2-hexoxy-4-(4-methylbenzoyl)oxybenzoate (D.6)

White powder. Yield: 0.070 g, 28%. RF: 0.63 (100% dichloromethane)

T_{CrI} 89 ˚C T_{N_FI} (73 ˚C)

IR cm^{-1}: 2933 ($-CH_2$), 2862 ($-CH_2$), 1749 (COOR), 1603 (Ar C=C), 1528 (NO_2), 1259 (C-O-C), 1215 (C-F).

δ_H/ppm (400 MHz, DMSO-d_6): 8.31 (t, *J* = 8.9 Hz, 1H, Ar-H), 8.06 (d, *J* = 3.8 Hz, 1H, Ar-H), 8.05 – 8.03 (m, 2H, Ar-H), 7.68 (dd, *J* = 12.0, 2.4 Hz, 1H, Ar-H), 7.44 (d, *J* = 8.2 Hz, 2H, Ar-H), 7.40 (ddd, *J* = 9.0, 2.5, 1.2 Hz, 1H, Ar-H), 7.25 (d, *J* = 2.1 Hz, 1H, Ar-H), 7.05 (dd, *J* = 8.6, 2.1 Hz, 1H, Ar-H), 4.09 (t, *J* = 6.3 Hz, 2H, $O\underline{CH_2}CH_2CH_2CH_2CH_3$), 2.44 (s, 3H, $-\underline{CH_3}$), 1.72 (p, *J* = 6.6 Hz, 2H, $OCH_2\underline{CH_2}CH_2CH_2CH_2CH_3$), 1.42 (p, *J* = 7.1 Hz, 2H, $OCH_2CH_2\underline{CH_2}CH_2CH_2CH_3$), 1.32 – 1.18 (m, 4H, $OCH_2CH_2CH_2\underline{CH_2}\underline{CH_2}CH_3$), 0.85 – 0.77 (m, 3H, $OCH_2CH_2CH_2CH_2CH_2\underline{CH_3}$).

δ_C/ppm (100 MHz, DMSO-d_6): 164.10, 162.12, 160.38, 156.65 (d, *J* = 264.5 Hz), 156.11, 155.70 (d, *J* = 11.5 Hz), 144.94, 134.65 (d, *J* = 7.6 Hz), 133.39, 130.06, 129.66, 127.62 (d, *J* = 2.3 Hz), 125.85, 119.15 (d, *J* = 3.5 Hz), 114.88, 114.05, 112.71 (d, *J* = 23.8 Hz), 108.05, 68.96, 30.89, 28.45, 25.10, 22.09, 21.35, 13.86.

MS = $[M+H]^+$ Calculated mass for $C_{27}H_{27}NO_7F$: 496.1772. Found: 496.1785. Diff: 2.6 ppm.

11.2.6 (3-Fluoro-4-nitrophenyl)-2-heptoxy-4-(4-methylbenzoyl)oxybenzoate (D.7)

White powder. Yield: 0.050 g, 18%. RF: 0.70 (100% dichloromethane)

T_{CrI} 66 ˚C T_{N_FI} 70 °C

IR cm^{-1}: 2915 ($-CH_2$), 2859 ($-CH_2$), 1742 (COOR), 1601 (Ar C=C), 1527 (NO_2), 1287 (C-O-C), 1228 (C-F).

δ_H/ppm (400 MHz, DMSO-d_6): 8.31 (t, *J* = 8.9 Hz, 1H, Ar-H), 8.08 – 8.02 (m, 3H, Ar-H), 7.69 (dd, *J* = 12.0, 2.4 Hz, 1H, Ar-H), 7.47 – 7.37 (m, 3H, Ar-H), 7.26 (d, *J* = 2.1 Hz, 1H, Ar-H), 7.06 (dd, *J* = 8.6, 2.1 Hz, 1H, Ar-H), 4.10 (t, *J* = 6.2 Hz, 2H, $O\underline{CH_2}CH_2CH_2CH_2CH_2CH_3$), 2.44 (s, 3H, $-\underline{CH_3}$), 1.73 (p, *J* = 6.5 Hz, 2H, $OCH_2\underline{CH_2}CH_2CH_2CH_2CH_3$), 1.42 (p, *J* = 7.1 Hz, 2H, $OCH_2CH_2\underline{CH_2}CH_2CH_2CH_3$), 1.32 – 1.13 (m, 4H, $OCH_2CH_2CH_2\underline{CH_2}\underline{CH_2}CH_3$), 0.81 (t, *J* = 6.8 Hz, 3H, $OCH_2CH_2CH_2CH_2CH_2\underline{CH_3}$).

δ_C/ppm (100 MHz, DMSO-d_6): 164.03, 162.11, 160.32, 156.60 (d, *J* = 262.3 Hz), 156.06, 155.78 (d, *J* = 15.7 Hz), 144.87, 134.00 (d, *J* = 6.3 Hz), 133.33, 130.00, 129.61, 127.57 (d, *J* = 1.5 Hz), 125.81, 119.10 (d, *J* = 3.6 Hz), 114.85, 114.01, 112.77 (d, *J* = 22.0 Hz), 108.02, 68.90, 31.23, 28.47, 28.33, 25.37, 21.99, 21.30, 13.90.

MS = $[M+H]^+$ Calculated mass for $C_{28}H_{29}NO_7F$: 510.1928. Found: 510.1946. Diff: 3.5 ppm.

11.3 (4-Nitrophenyl)-2-alkoxy-4-(2-methoxy-4-methylbenzoyl)oxybenzoates

Tables SI17. Quantities of reagents used to synthesise (4-nitrophenyl)-2-alkoxy-4-(2-methoxy-4-methylbenzoyl)oxybenzoates.

m	(**10.2**)	4-Nitrophenol	DCC

2	0.200 g, 6.05×10^{-4} mol	0.076 g, 5.50×10^{-4} mol	0.142 g, 6.85×10^{-4} mol
3	0.300 g, 8.71×10^{-4} mol	0.110 g, 7.92×10^{-4} mol	0.204 g, 9.90×10^{-4} mol
4	0.250 g, 6.98×10^{-4} mol	0.088 g, 6.35×10^{-4} mol	0.163 g, 7.94×10^{-4} mol
5	0.300 g, 8.06×10^{-4} mol	0.115 g, 7.32×10^{-4} mol	0.175 g, 9.15×10^{-4} mol
6	0.250 g, 4.92×10^{-4} mol	0.044 g, 3.20×10^{-4} mol	0.066 g, 3.20×10^{-4} mol
7	0.250 g, 6.24×10^{-4} mol	0.074 g, 4.68×10^{-4} mol	0.097 g, 4.68 ×10^{-4} mol

11.3.1 (4-Nitrophenyl)-2-ethoxy-4-(2-methoxy-4-methylbenzoyl)oxybenzoate (G.2)

White powder. Yield: 0.033 g, 12%. RF: 0.16 (100% dichloromethane)

T_{CrI} 122 ˚C $T_{N_F I}$ (77 ˚C)

ν_{max}/cm^{-1}: 2922 (-CH_2), 1739 (COOR), 1610 (Ar C=C), 1592, 1524 (NO_2), 1203 (C-O-C).

δ_H/ppm (400 MHz, DMSO-d_6): 8.35 (d, *J* = 8.9 Hz, 2H, Ar-H), 8.04 (d, *J* = 8.6 Hz, 1H, Ar-H), 7.89 (d, *J* = 7.9 Hz, 1H, Ar-H), 7.58 (d, *J* = 9.1 Hz, 2H, Ar-H), 7.16 (d, *J* = 2.1 Hz, 1H, Ar-H), 7.08 (s, 1H, Ar-H), 6.99 (dd, *J* = 8.6, 2.1 Hz, 1H, Ar-H), 6.93 (d, *J* = 8.0 Hz, 1H, Ar-H), 4.16 (q, *J* = 6.9 Hz, 2H, O$\underline{CH_2}$$CH_3$), 3.87 (s, 3H, O$\underline{CH_3}$), 2.40 (s, 3H, -$\underline{CH_3}$), 1.35 (t, *J* = 6.9 Hz, 3H, $OCH_2$$\underline{CH_3}$).

δ_C/ppm (100 MHz, DMSO-d_6): 162.91, 162.55, 160.07, 159.64, 155.96, 155.64, 146.09, 145.12, 133.11, 132.04, 125.42, 123.38, 121.03, 115.26, 114.89, 114.19, 113.45, 108.10, 64.82, 55.95, 21.64, 14.48.

MS = $[M+H]^+$ Calculated mass for $C_{24}H_{22}NO_8$: 452.135. Found: 452.136. Diff: 2.2 ppm.

11.3.2 (4-Nitrophenyl)-2-propoxy-4-(2-methoxy-4-methylbenzoyl)oxybenzoate (G.3)

White powder. Yield: 0.045 g, 12%. RF: 0.20 (100% dichloromethane)

T_{CrI} 110 ˚C $T_{N_F I}$ (62 ˚C)

ν_{max}/cm^{-1}: 2968 (-CH_3), 2937 (-CH_2), 1737 (COOR), 1610 (Ar C=C), 1518 (NO_2), 1229 (C-O-C).

δ_H/ppm (400 MHz, DMSO-d_6): 8.36 (d, *J* = 9.2 Hz, 2H, Ar-H), 8.05 (d, *J* = 8.5 Hz, 1H, Ar-H), 7.89 (d, *J* = 7.9 Hz, 1H, Ar-H), 7.58 (d, *J* = 9.1 Hz, 2H, Ar-H), 7.17 (d, *J* = 2.1 Hz, 1H, Ar-H), 7.09 (d, *J* = 1.4 Hz, 1H, Ar-H), 6.99 (dd, *J* = 8.6, 2.1 Hz, 1H, Ar-H), 6.96 – 6.90 (m, 1H, Ar-H), 4.07 (t, *J* = 6.3 Hz, 2H, O$\underline{CH_2}$$CH_2CH_3$), 3.88 (s, 3H, O$\underline{CH_3}$), 2.41 (s, 3H, -$\underline{CH_3}$), 1.75 (h, *J* = 7.0 Hz, 2H, $OCH_2$$\underline{CH_2}$$CH_3$), 0.99 (t, *J* = 7.4 Hz, 3H, $OCH_2CH_2$$\underline{CH_3}$).

δ_C/ppm (100 MHz, DMSO-d_6): 162.81, 162.56, 160.18, 159.58, 155.95, 155.60, 145.99, 145.06, 133.14, 131.98, 125.38, 123.30, 120.95, 115.06, 114.85, 114.08, 113.40, 107.95, 70.32, 55.89, 21.93, 21.58, 10.39.

EA: Calculated: C 64.51%, H 4.98%, N 3.01%. Found: C 64.44%, H 5.10%, N 2.97%.

11.3.3 (4-Nitrophenyl)-2-butoxy-4-(2-methoxy-4-methylbenzoyl)oxybenzoate (G.4)

White powder. Yield: 0.093 g, 30%. RF: 0.25 (100% dichloromethane)

T_{CrI} 124 ˚C T_{N_FI} (42 ˚C)

v_{max}/cm^{-1}: 3066 (Aryl-H), 2955 (-CH_3), 2928 (-CH_2), 2864 (-CH_2), 1731 (COOR), 1605 (Ar C=C), 1520 (NO_2), 1281 (C-O-C).

δ_H/ppm (400 MHz, DMSO-d_6): 8.36 (d, *J* = 8.8 Hz, 2H, Ar-H), 8.04 (d, *J* = 8.6 Hz, 1H, Ar-H), 7.89 (d, *J* = 7.9 Hz, 1H, Ar-H), 7.57 (d, *J* = 8.5 Hz, 2H, Ar-H), 7.18 (d, *J* = 2.1 Hz, 1H, Ar-H), 7.08 (d, *J* = 2.1 Hz, 1H, Ar-H), 6.99 (dd, *J* = 8.8, 1.7 Hz, 1H, Ar-H), 6.93 (dd, *J* = 8.0, 2.1 Hz, 1H, Ar-H), 4.11 (t, *J* = 6.3 Hz, 2H, O$\underline{CH_2}CH_2CH_2CH_3$), 3.87 (s, 3H, O$\underline{CH_3}$), 2.41 (s, 3H, -$\underline{CH_3}$), 1.72 (p, *J* = 6.5 Hz, 2H, $OCH_2\underline{CH_2}CH_2CH_3$), 1.46 (h, *J* = 7.4 Hz, 2H, $OCH_2CH_2\underline{CH_2}CH_3$), 0.89 (t, *J* = 7.5 Hz, 3H, $OCH_2CH_2CH_2\underline{CH_3}$).

δ_C/ppm (100 MHz, DMSO-d_6): 162.80, 162.52, 160.20, 159.59, 155.96, 155.61, 145.99, 145.04, 133.12, 131.98, 125.36, 123.28, 120.94, 115.04, 114.84, 114.06, 113.39, 107.94, 68.53, 55.88, 30.53, 21.57, 18.60, 13.57.

EA: Calculated: C 65.13%, H 5.26%, N 2.92%. Found: C 65.10%, H 4.98%, N 2.92%.

11.3.4 (4-Nitrophenyl)-2-pentoxy-4-(2-methoxy-4-methylbenzoyl)oxybenzoate (G.5)

White powder. Yield: 0.094 g, 26%. RF: 0.52 (100% dichloromethane)

T_{CrI} 124 ˚C T_{N_FI} (28 ˚C)

v_{max}/cm^{-1}: 2950 (-CH_3), 2868 (-CH_2), 1731 (COOR), 1607 (Ar C=C), 1517 (NO_2), 1268 (C-O-C).

δ_H/ppm (400 MHz, DMSO-d_6): 8.36 (d, *J* = 9.3 Hz, 2H, Ar-H), 8.04 (d, *J* = 8.6 Hz, 1H, Ar-H), 7.89 (d, *J* = 7.9 Hz, 1H, Ar-H), 7.57 (d, *J* = 9.2 Hz, 2H, Ar-H), 7.17 (d, *J* = 2.1 Hz, 1H, Ar-H), 7.09 (d, *J* = 1.3 Hz, 1H, Ar-H), 6.99 (dd, *J* = 8.5, 2.1 Hz, 1H, Ar-H), 6.93 (dd, *J* = 8.1, 1.1 Hz, 1H, Ar-H), 4.10 (t, *J* = 6.3 Hz, 2H, O$\underline{CH_2}CH_2CH_2CH_2CH_3$), 3.87 (s, 3H, O$\underline{CH_3}$), 2.41 (s, 3H, -$\underline{CH_3}$), 1.73 (p, *J* = 6.5 Hz, 2H, $OCH_2\underline{CH_2}CH_2CH_2CH_3$), 1.47 – 1.36 (m, 2H, $OCH_2CH_2\underline{CH_2}CH_2CH_3$), 1.35 – 1.23 (m, 2H, $OCH_2CH_2CH_2\underline{CH_2}CH_3$), 0.82 (t, *J* = 7.3 Hz, 3H, $OCH_2CH_2CH_2CH_2\underline{CH_3}$).

δ_C/ppm (100 MHz, DMSO-d_6): 162.81, 162.61, 160.16, 159.58, 155.95, 155.62, 145.99, 145.04, 133.12, 131.98, 125.36, 123.27, 120.95, 115.08, 114.84, 114.08, 113.39, 107.95, 68.83, 55.88, 28.15, 27.57, 21.73, 21.58, 13.87.

EA: Calculated: C 65.71%, H 5.51%, N 2.84%. Found: C 65.69%, H 5.32%, N 2.84%.

11.3.5 (4-Nitrophenyl)-2-hexyloxy-4-(2-methoxy-4-methylbenzoyl)oxybenzoate (G.5)

White powder. Yield: 0.064 g, 39%. RF: 0.42 (Dichloromethane)

T_{CrI} 123 ˚C T_{N_FI} (26 ˚C)

v_{max}/cm^{-1}: 2933 (-CH_2), 2857 (-CH_2), 1736 (COOR), 1605 (Ar C=C), 1520 (NO_2), 1229 (C-O-C).

δ_H/ppm (400 MHz, DMSO-d_6): 8.35 (d, *J* = 9.2 Hz, 2H, Ar-H), 8.03 (d, *J* = 8.5 Hz, 1H, Ar-H), 7.89 (d, *J* = 7.9 Hz, 1H, Ar-H), 7.56 (d, *J* = 9.1 Hz, 2H, Ar-H), 7.17 (d, *J* = 2.1 Hz, 1H, Ar-H), 7.18 – 7.02 (m, 1H, Ar-H), 6.99 (dd, *J* = 8.6, 2.1 Hz, 1H, Ar-H), 6.93 (dd, *J* = 8.3, 1.2 Hz, 1H, Ar-H), 4.10 (t, *J* = 6.2 Hz, 2H, O$\underline{CH_2}CH_2CH_2CH_2CH_2CH_3$), 3.87 (s, 3H, O$\underline{CH_3}$), 2.41 (s, 3H, -$\underline{CH_3}$), 1.72 (p, *J* = 6.4 Hz, 2H,

$OCH_2\underline{CH_2}CH_2CH_2CH_2CH_3$), 1.43 (p, *J* = 7.4 Hz, 2H, $OCH_2CH_2\underline{CH_2}CH_2CH_2CH_3$), 1.32 – 1.15 (m, 4H, $OCH_2CH_2CH_2\underline{CH_2CH_2}CH_3$), 0.80 (t, *J* = 7.0 Hz, 3H, $OCH_2CH_2CH_2CH_2CH_2\underline{CH_3}$).

δ_C/ppm (100 MHz, DMSO-d_6): 162.83, 162.67, 160.16, 159.60, 155.96, 155.64, 146.01, 145.05, 133.13, 131.99, 125.37, 123.27, 120.97, 115.11, 114.85, 114.10, 113.41, 107.96, 68.86, 55.90, 30.85, 28.45, 25.07, 22.05, 21.59, 13.83.

EA: Calculated: C 66.26%, H 5.76%, N 2.76%. Found: C 66.02%, H 5.79%, N 2.73%.

11.3.6 (4-Nitrophenyl)-2-heptoxy-4-(2-methoxy-4-methylbenzoyl)oxybenzoate (G.5)

White powder. Yield: 0.100 g, 40%. RF: 0.44 (100% dichloromethane)

T_{CrI} 101 ˚C T_{N_FI} (20 ˚C)

v_{max}/cm^{-1}: 3083 (Aryl-H), 2921 (-CH_2), 2855 (-CH_2), 1736 (COOR), 1605 (Ar C=C), 1520 (NO_2), 1228 (C-O-C).

δ_H/ppm (400 MHz, DMSO-d_6): 8.36 (d, *J* = 8.8 Hz, 2H, Ar-H), 8.03 (d, *J* = 8.5 Hz, 1H, Ar-H), 7.89 (d, *J* = 7.9 Hz, 1H, Ar-H), 7.56 (d, *J* = 9.2 Hz, 2H, Ar-H), 7.17 (d, *J* = 2.0 Hz, 1H, Ar-H), 7.10 – 7.07 (m, 1H, Ar-H), 6.99 (dd, *J* = 8.7, 1.5 Hz, 1H, Ar-H), 6.93 (dd, *J* = 8.0, 1.0 Hz, 1H, Ar-H), 4.09 (t, *J* = 6.2 Hz, 2H, $O\underline{CH_2}CH_2CH_2CH_2CH_2CH_2CH_3$), 3.87 (s, 3H, $O\underline{CH_3}$), 2.41 (s, 3H, -$\underline{CH_3}$), 1.72 (p, *J* = 6.4 Hz, 2H, $OCH_2\underline{CH_2}CH_2CH_2CH_2CH_2CH_3$), 1.41 (p, *J* = 7.5 Hz, 2H, $OCH_2CH_2\underline{CH_2}CH_2CH_2CH_2CH_3$), 1.35 – 1.10 (m, 6H, $OCH_2CH_2CH_2\underline{CH_2CH_2\,CH_2}CH_3$), 0.79 (t, *J* = 6.5 Hz, 3H, $OCH_2CH_2CH_2CH_2CH_2CH_2\underline{CH_3}$).

δ_C/ppm (100 MHz, DMSO-d_6): 162.81, 162.71, 160.15, 159.59, 155.96, 155.64, 146.00, 145.03, 133.12, 131.98, 125.35, 123.24, 120.95, 115.11, 114.85, 114.09, 113.39, 107.94, 68.84, 55.88, 31.22, 28.51, 28.33, 25.38, 21.98, 21.58, 13.90.

EA: Calculated: C 66.78%, H 5.99%, N 2.69%. Found: C 67.09%, H 5.57%, N 2.67%.

11.4 (3-Fluoro-4-nitrophenyl)-2-alkyloxy-4-(2-methoxy-4-methylbenzoyl)oxybenzoates

Tables SI18. Quantities of reagents used to synthesise (3-fluoro-4-nitrophenyl)-2-alkyloxy-4-(2-methoxy-4-methylbenzoyl)oxybenzoates.

m	(**10.2**)	3-Fluoro-4-nitrophenol	DCC
2	0.200 g, 9.08×10^{-4} mol	0.107 g, 6.81×10^{-4} mol	0.140 g, 6.81×10^{-4} mol
3	0.300 g, 8.71×10^{-4} mol	0.124 g, 7.92×10^{-4} mol	0.204 g, 9.90×10^{-4} mol
4	0.250 g, 6.98×10^{-4} mol	0.100 g, 6.35×10^{-4} mol	0.163 g, 9.05×10^{-4} mol
5	0.300 g, 8.06×10^{-4} mol	0.115 g, 7.32×10^{-4} mol	0.175 g, 9.15×10^{-4} mol

6	0.250 g, 6.47×10^{-4} mol	0.503 g, 3.20×10^{-4} mol	0.066 g, 3.20×10^{-4} mol
7	0.250 g, 6.24×10^{-4} mol	0.074 g, 4.68×10^{-4} mol	0.097 g, 4.68×10^{-4} mol

11.4.1 (3-Fluoro-4-nitrophenyl)-2-ethoxy-4-(2-methoxy-4-methylbenzoyl)oxybenzoate (H.2)

White powder. Yield: 0.032 g, 11%. RF: 0.29 (100% dichloromethane)

T_{CrI} 144 ˚C T_{N_FI} (88 ˚C)

v_{max}/cm^{-1}: 3056 (Aryl-H), 2999 (-CH_3), 2922 (-CH_2), 1745 (COOR), 1603 (Ar C=C), 1521 (NO_2), 1279 (C-O-C), 1220 (C-F).

δ_H/ppm (400 MHz, DMSO-d_6): 8.30 (t, *J* = 8.8 Hz, 1H, Ar-H), 8.06 (d, *J* = 8.5 Hz, 1H, Ar-H), 7.89 (d, *J* = 7.9 Hz, 1H, Ar-H), 7.71 (dd, *J* = 12.0, 2.3 Hz, 1H, Ar-H), 7.42 (dt, *J* = 9.1, 2.6 Hz, 1H, Ar-H), 7.17 (d, *J* = 1.7 Hz, 1H, Ar-H), 7.08 (s, 1H, Ar-H), 7.00 (dd, *J* = 8.5, 1.6 Hz, 1H, Ar-H), 6.93 (d, *J* = 8.0 Hz, 1H, Ar-H), 4.18 (q, *J* = 7.0 Hz, 2H, O$\underline{CH_2}$$CH_3$), 3.87 (s, 3H, O$\underline{CH_3}$), 2.41 (s, 3H, -$\underline{CH_3}$), 1.36 (t, *J* = 6.9 Hz, 3H, O$CH_2$$\underline{CH_3}$).

δ_C/ppm (100 MHz, DMSO-d_6): 162.75, 161.85, 160.22, 159.58, 156.09, 155.69 (d, *J* = 11.4 Hz), 153.98 (d, *J* = 263.6 Hz), 145.99, 134.55 (d, *J* = 7.2 Hz), 133.23, 131.97, 127.53 (d, *J* = 2.0 Hz), 120.94, 119.17 (d, *J* = 3.7 Hz), 114.81, 114.66, 114.12, 113.38, 112.73 (d, *J* = 23.8 Hz), 108.06, 64.75, 55.87, 21.57, 14.39.

MS = $[M+H]^+$ Calculated mass for $C_{24}H_{21}NO_8F$: 470.1251. Found: 470.1267. Diff: 3.4 ppm.

11.4.2 (3-Fluoro-4-nitrophenyl)-2-propoxy-4-(2-methoxy-4-methylbenzoyl)oxybenzoate (H.3)

White powder. Yield: 0.036 g, 8%. RF: 0.34 (100% dichloromethane)

T_{CrI} 134 ˚C T_{N_FI} (73 ˚C)

v_{max}/cm^{-1}: 3085 (Aryl-H), 2967 (-CH_3), 2935 (-CH_2), 2876 (-CH_2), 1735 (COOR), 1603 (Ar C=C), 1520 (NO_2), 1266 (C-O-C), 1213 (C-F).

δ_H/ppm (400 MHz, DMSO-d_6): 8.31 (t, *J* = 8.9 Hz, 1H, Ar-H), 8.06 (d, *J* = 8.6 Hz, 1H, Ar-H), 7.89 (d, *J* = 7.9 Hz, 1H, Ar-H), 7.71 (dd, *J* = 12.0, 2.4 Hz, 1H, Ar-H), 7.41 (ddd, *J* = 9.0, 2.4, 1.2 Hz, 1H, Ar-H), 7.17 (d, *J* = 2.1 Hz, 1H, Ar-H), 7.09 (d, *J* = 1.5 Hz, 1H, Ar-H), 7.00 (dd, *J* = 8.6, 2.1 Hz, 1H, Ar-H), 6.94 (ddd, *J* = 7.9, 1.5, 0.7 Hz, 1H, Ar-H), 4.07 (t, *J* = 6.3 Hz, 2H, O$\underline{CH_2}$$CH_2CH_3$), 3.87 (s, 3H, O$\underline{CH_3}$), 2.41 (s, 3H, -$\underline{CH_3}$), 1.82 – 1.69 (m, 2H, O$CH_2$$\underline{CH_2}$$CH_3$), 0.99 (t, *J* = 7.4 Hz, 3H, O$CH_2CH_2$$\underline{CH_3}$).

δ_C/ppm (100 MHz, DMSO-d_6): 162.76, 161.94, 160.41, 159.59, 156.15, 155.71 (d, *J* = 11.1 Hz), 153.99 (d, *J* = 262.4 Hz), 146.00, 134.61 (d, *J* = 7.4 Hz), 133.33, 131.98, 127.57 (d, *J* = 2.0 Hz), 120.94, 119.16 (d, *J* = 3.7 Hz), 114.81, 114.53, 114.08, 113.39, 112.73 (d, *J* = 23.6 Hz), 107.97, 70.34, 55.88, 21.90, 21.57, 10.39.

MS = $[M+Na]^+$ Calculated mass for $C_{25}H_{22}NO_8FNa$: 506.123. Found: 506.122. Diff: 0.6 ppm.

11.4.3 (3-Fluoro-4-nitrophenyl)-2-butoxy-4-(2-methoxy-4-methylbenzoyl)oxybenzoate (H.4)

White powder. Yield: 0.027 g, 8%. RF: 0.51 (100% dichloromethane)

T_{CrI} 100 ˚C T_{N_FI} (56 ˚C)

v_{max}/cm^{-1}: 2956 (-CH_3), 2873 (-CH_2), 1715 (COOR), 1600 (Ar C=C), 1520 (NO_2), 1285 (C-O-C), 1217 (C-F).

δ_H/ppm (400 MHz, DMSO-d_6): 8.32 (t, *J* = 8.9 Hz, 1H, Ar-H), 8.06 (d, *J* = 8.6 Hz, 1H, Ar-H), 7.90 (d, *J* = 7.9 Hz, 1H, Ar-H), 7.70 (dd, *J* = 11.9, 2.4 Hz, 1H, Ar-H), 7.41 (dt, *J* = 9.0, 1.1 Hz, 1H, Ar-H), 7.19 (d, *J* = 2.2 Hz, 1H, Ar-H), 7.09 (s, 1H, Ar-H), 7.00 (dd, *J* = 8.6, 2.1 Hz, 1H, Ar-H), 6.95 (dd, *J* = 7.9, 0.8 Hz, 1H, Ar-H), 4.12 (t, *J* = 6.3 Hz, 2H, $O\underline{CH_2}CH_2CH_2CH_3$), 3.88 (s, 3H, -$\underline{CH_3}$), 2.42 (s, 3H, $O\underline{CH_3}$), 1.73 (p, *J* = 6.9 Hz, 2H, $OCH_2\underline{CH_2}CH_2CH_3$), 1.47 (h, *J* = 7.4 Hz, 2H, $OCH_2CH_2\underline{CH_2}CH_3$), 0.91 (t, *J* = 7.4 Hz, 3H, $OCH_2CH_2CH_2\underline{CH_3}$).

δ_C/ppm (100 MHz, DMSO-d_6): 162.76, 161.91, 160.43, 159.59, 156.16, 155.78 (d, *J* = 11.9 Hz), 153.99 (d, *J* = 262.9 Hz), 146.01, 134.56 (d, *J* = 7.2 Hz), 133.32, 131.98, 127.56 (d, *J* = 2.0 Hz), 120.94, 119.15 (d, *J* = 4.0 Hz), 114.81, 114.52, 114.08, 113.39, 112.71 (d, *J* = 23.7 Hz), 107.96, 68.55, 55.88, 30.51, 21.58, 18.59, 13.58.

MS = $[M+Na]^+$ Calculated mass for $C_{26}H_{24}NO_8FNa$: 520.138. Found: 520.137. Diff: 3.5 ppm.

11.4.4 (3-Fluoro-4-nitrophenyl)-2-pentoxy-4-(2-methoxy-4-methylbenzoyl)oxybenzoate (H.5)

White powder. Yield: 0.030 g, 8%. RF: 0.45 (100% dichloromethane)

T_{CrI} 101 ˚C T_{N_FI} (43 ˚C)

v_{max}/cm^{-1}: 2955 (-CH_3), 2868 (-CH_2), 1723 (COOR), 1600 (Ar C=C), 1520 (NO_2), 1285 (C-O-C), 1212 (C-F).

δ_H/ppm (400 MHz, DMSO-d_6): 8.31 (t, *J* = 8.9 Hz, 1H, Ar-H), 8.05 (d, *J* = 8.6 Hz, 1H, Ar-H), 7.89 (d, *J* = 7.9 Hz, 1H, Ar-H), 7.69 (dd, *J* = 12.0, 2.4 Hz, 1H, Ar-H), 7.40 (dt, *J* = 9.0, 1.1 Hz, 1H, Ar-H), 7.18 (d, *J* = 2.1 Hz, 1H, Ar-H), 7.12 – 7.04 (m, 1H, Ar-H), 6.99 (dd, *J* = 8.6, 2.1 Hz, 1H, Ar-H), 6.94 (d, *J* = 7.9 Hz, 1H, Ar-H), 4.10 (t, *J* = 6.3 Hz, 2H, $O\underline{CH_2}CH_2CH_2CH_2CH_3$), 3.87 (s, 3H, $O\underline{CH_3}$), 2.41 (s, 3H, -$\underline{CH_3}$), 1.73 (p, *J* = 6.5 Hz, 2H, $OCH_2\underline{CH_2}CH_2CH_2CH_3$), 1.41 (p, *J* = 8.4 Hz, 2H, $OCH_2CH_2\underline{CH_2}CH_2CH_3$), 1.31 (h, *J* = 7.1 Hz, 2H, $OCH_2CH_2CH_2\ \underline{CH_2}CH_3$), 0.83 (t, *J* = 7.2 Hz, 3H, $OCH_2CH_2CH_2CH_2\underline{CH_3}$).

δ_C/ppm (100 MHz, DMSO-d_6): 162.77, 162.01, 160.40, 159.60, 156.16, 155.74 (d, *J* = 11.2 Hz), 154.00 (d, *J* = 262.6 Hz), 146.01, 134.55 (d, *J* = 7.2 Hz), 133.32, 131.99, 127.56 (d, *J* = 1.9 Hz), 120.95, 119.13 (d, *J* = 3.6 Hz), 114.81, 114.56, 114.09, 113.39, 112.68 (d, *J* = 23.7 Hz), 107.96, 68.86, 55.88, 28.15, 27.58, 21.75, 21.58, 13.87.

MS = $[M+H]^+$ Calculated mass for $C_{27}H_{27}NO_8F$: 512.172. Found: 512.174. Diff: 3.3 ppm.

11.4.5 (3-Fluoro-4-nitrophenyl)-2-hexoxy-4-(2-methoxy-4-methylbenzoyl)oxybenzoate (H.6)

White powder. Yield: 0.053 g, 31%. RF: 0.34 (100% dichloromethane)

T_{CrI} 86 ˚C T_{N_FI} (40 ˚C)

IR cm^{-1}: 2939 (-CH_2), 1724 (COOR), 1601 (Ar C=C), 1527 (NO_2), 1282 (C-O-C), 1216 (C-F).

δ_H/ppm (400 MHz, DMSO-d_6): 8.31 (t, *J* = 8.9 Hz, 1H, Ar-H), 8.04 (d, *J* = 8.6 Hz, 1H, Ar-H), 7.89 (d, *J* = 7.9 Hz, 1H, Ar-H), 7.69 (dd, *J* = 12.0, 2.4 Hz, 1H, Ar-H), 7.40 (dt, *J* = 9.1, 1.1 Hz, 1H, Ar-H), 7.17 (d, *J* = 2.1 Hz, 1H, Ar-H), 7.08 (s, 1H, Ar-H), 6.99 (dd, *J* = 8.6, 2.1 Hz, 1H, Ar-H), 6.93 (d, *J* = 7.7 Hz, 1H, Ar-H), 4.09 (t, *J* = 6.2 Hz, 2H, $O\underline{CH_2}CH_2CH_2CH_2CH_2CH_3$), 3.87 (s, 3H, $O\underline{CH_3}$), 2.41 (s, 3H, -$\underline{CH_3}$), 1.72 (p, *J* = 7.3 Hz, 2H, $OCH_2\underline{CH_2}CH_2CH_2CH_2CH_3$), 1.43 (p, *J* = 7.4 Hz, 2H, $OCH_2CH_2\underline{CH_2}CH_2CH_2CH_3$), 1.32 – 1.16 (m, 4H, $OCH_2CH_2CH_2\,\underline{CH_2CH_2}CH_3$), 0.81 (t, *J* = 6.9 Hz, 3H, $OCH_2CH_2CH_2CH_2CH_2\underline{CH_3}$).

δ_C/ppm (100 MHz, DMSO-d_6): 162.81, 162.10, 160.40, 159.63, 156.17, 155.76 (d, *J* = 10.8 Hz), 154.02 (d, *J* = 263.0 Hz), 146.06, 134.62 (d, *J* = 7.9 Hz), 133.34, 132.02, 127.60, 120.99, 119.14 (d, *J* = 3.9 Hz), 114.83, 114.63, 114.13, 113.42, 112.70 (d, *J* = 23.4 Hz), 107.99, 68.91, 55.91, 30.88, 28.46, 25.09, 22.07, 21.61, 13.85.

MS = $[M+H]^+$ Calculated mass for $C_{28}H_{29}NO_8F$: 536.188. Found: 526.186. Diff: 4.4 ppm.

11.4.6 (3-Fluoro-4-nitrophenyl)-2-heptoxy-4-(2-methoxy-4-methylbenzoyl)oxybenzoate (H.7)

White powder. Yield: 0.122 g, 48%. RF: 0.54 (100% dichloromethane)

T_{CrI} 93 ˚C T_{N_FI} (37 ˚C)

ν_{max}/cm^{-1}: 3057 (Aryl-H), 2929 (-CH_2), 2856 (-CH_2), 1720 (COOR), 1601 (Ar C=C), 1520 (NO_2), 1282 (C-O-C), 1216 (C-F).

δ_H/ppm (400 MHz, DMSO-d_6): 8.31 (t, *J* = 8.8 Hz, 1H, Ar-H), 8.04 (d, *J* = 8.6 Hz, 1H, Ar-H), 7.88 (d, *J* = 7.8 Hz, 1H, Ar-H), 7.68 (dd, *J* = 12.0, 2.3 Hz, 1H, Ar-H), 7.40 (dt, *J* = 9.2, 1.1 Hz, 1H, Ar-H), 7.17 (d, *J* = 2.0 Hz, 1H, Ar-H), 7.18 – 7.02 (m, 1H, Ar-H), 6.99 (dd, *J* = 8.6, 2.0 Hz, 1H, Ar-H), 6.93 (d, *J* = 8.0 Hz, 1H, Ar-H), 4.09 (t, *J* = 6.2 Hz, 2H, $O\underline{CH_2}CH_2CH_2CH_2CH_2\,CH_2CH_3$), 3.87 (s, 3H, $O\underline{CH_3}$), 2.41 (s, 3H, -$\underline{CH_3}$), 1.72 (p, *J* = 6.6 Hz, 2H, $OCH_2\underline{CH_2}CH_2\,CH_2CH_2CH_2CH_3$), 1.41 (p, *J* = 7.0 Hz, 2H, $OCH_2CH_2\underline{CH_2}CH_2\,CH_2CH_2CH_3$), 1.35 – 1.11 (m, 6H, $OCH_2CH_2CH_2\underline{CH_2CH_2CH_2}CH_3$), 0.80 (t, *J* = 7.0 Hz, 3H, $OCH_2CH_2CH_2CH_2\,CH_2CH_2\underline{CH_3}$).

δ_C/ppm (100 MHz, DMSO-d_6): 162.75, 162.10, 160.35, 159.58, 156.14, 155.73 (d, *J* = 11.2 Hz), 153.98 (d, *J* = 263.8 Hz), 145.99, 134.57 (d, *J* = 6.2 Hz), 133.30, 131.97, 127.54, 120.93, 119.11 (d, *J* = 3.9 Hz), 114.80, 114.59, 114.09, 113.39, 112.64 (d, *J* = 23.7 Hz), 107.96, 68.86, 55.87, 31.23, 28.48, 28.33, 25.37, 21.98, 21.57, 13.88.

MS = $[2M+Na]^+$ Calculated mass for $C_{59}H_{60}N_2O_{16}F_2Na$: 1101.381. Found: 1101.378. Diff: 2.4 ppm.

Method 2: Under inert conditions, a mixture of the required **Compound 10** *(*1.1 eq) and either 3-fluoro-4-nitrophenol (1 eq) or 4-nitrophenol (1 eq) as required, were dissolved in dry dichloromethane (10 ml). To this, DCC (1.25 eq) in dichloromethane (2 mL) was added and allowed to react for 1 h. A catalytic amount of DMAP was added to the solution and allowed to react overnight. The quantities of reagents used in the reaction are listed in Tables SI19-20. The reaction mixture was then quenched by filtration of the precipitated DCU and then purified by flash column chromatography with a mixture of dichloromethane:EtOAc (97:3). The product was then purified by hot recrystallisation and filtration in ethanol.

11.5 (4-Nitrophenyl)-2-alkyloxy-4-(2,4-dimethoxybenzoyl)oxybenzoates

Tables SI19. Quantities of reagents used to synthesise (4-nitrophenyl)-2-alkoxy-4-(2,4-dimethoxy benzoyl)oxybenzoates.

m	**(10.3)**	4-Nitrophenol	DCC
2	0.200 g, 5.74×10^{-4} mol	0.051 g, 3.67×10^{-4} mol	0.095 g, 4.58×10^{-4} mol
3	0.188 g, 5.20×10^{-4} mol	0.066 g, 4.73×10^{-4} mol	0.121 g, 5.91×10^{-4} mol
4	0.250 g, 7.17×10^{-4} mol	0.091 g, 6.52×10^{-4} mol	0.168 g, 8.15×10^{-4} mol
5	0.250 g, 6.43×10^{-4} mol	0.081 g, 5.85×10^{-4} mol	0.151 g, 7.30×10^{-4} mol
6	0.300 g, 7.45×10^{-4} mol	0.094 g, 6.77×10^{-4} mol	0.174 g, 8.46×10^{-4} mol
7	0.250 g, 6.00×10^{-4} mol	0.076 g, 5.46×10^{-4} mol	0.140 g, 6.82×10^{-4} mol

11.5.1 (4-Nitrophenyl)-2-ethoxy-4-(2,4-dimethoxybenzoyl)oxybenzoate (K.2)

White powder. Yield: 0.036 g, 19%. RF: 0.10 (100% dichloromethane)

T_{CrI} 154 ˚C T_{N_FI} (72 ˚C)

ν_{max}/cm^{-1}: 3086 (Aryl-H), 2971 (-CH_3), 2930 (-CH_2), 2840 (-CH_2), 1743 (COOR), 1593 (Ar C=C), 1516 (NO_2), 1233 (C-O-C).

δ_H/ppm (400 MHz, DMSO-d_6): 8.36 (d, *J* = 9.0 Hz, 2H, Ar-H), 8.04 (d, *J* = 8.5 Hz, 1H, Ar-H), 8.00 (d, *J* = 8.7 Hz, 1H, Ar-H), 7.58 (d, *J* = 9.1 Hz, 2H, Ar-H), 7.15 (d, *J* = 2.2 Hz, 1H, Ar-H), 6.98 (dd, *J* = 8.5, 2.1 Hz, 1H, Ar-H), 6.73 (d, *J* = 2.3 Hz, 1H, Ar-H), 6.69 (dd, *J* = 8.8, 2.3 Hz, 1H, Ar-H), 4.17 (q, *J* = 6.9 Hz, 2H, $O\underline{CH_2}CH_3$), 3.95 – 3.82 (m, 6H, 2× $O\underline{CH_3}$), 1.35 (t, *J* = 6.9 Hz, 3H, $OCH_2\underline{CH_3}$).

δ_C/ppm (100 MHz, DMSO-d_6): 165.05, 162.52, 162.25, 161.89, 160.02, 156.07, 155.63, 145.08, 134.19, 133.03, 125.38, 123.35, 115.08, 114.22, 109.75, 108.14, 105.78, 99.02, 64.75, 56.06, 55.79, 14.46.

MS = $[M+H]^+$ Calculated mass for $C_{24}H_{22}NO_9$:468.130. Found: 468.131. Diff: 2.3 ppm.

11.5.2 (4-Nitrophenyl)-2-propoxy-4-(2,4-dimethoxybenzoyl)oxybenzoate (K.3)

White powder. Yield: 0.109 g, 44%. RF: 0.14 (100% dichloromethane)

T_{CrI} 133 ˚C T_{N_FI} (51 ˚C)

ν_{max}/cm^{-1}: 2967 (-CH_3), 2942 (-CH_2), 2878 (-CH_2), 1747 (COOR), 1520 (NO_2), 1234 (C-O-C).

δ_H/ppm (400 MHz, DMSO-d_6): 8.36 (d, *J* = 9.0 Hz, 2H, Ar-H), 8.04 (d, *J* = 8.6 Hz, 1H, Ar-H), 8.00 (d, *J* = 8.7 Hz, 1H, Ar-H), 7.57 (d, *J* = 9.1 Hz, 2H, Ar-H), 7.15 (d, *J* = 2.1 Hz, 1H, Ar-H), 6.97 (dd, *J* = 8.5, 2.1 Hz, 1H, Ar-H), 6.73 (d, *J* = 2.3 Hz, 1H, Ar-H), 6.69 (dd, *J* = 8.8, 2.3 Hz, 1H, Ar-H), 4.07 (t, *J* = 6.2 Hz, 2H, $O\underline{CH_2}CH_2CH_3$), 3.94 – 3.86 (m, 6H, 2× $O\underline{CH_3}$), 1.75 (h, *J* = 6.9 Hz, 2H, $OCH_2\underline{CH_2}CH_3$), 0.98 (t, *J* = 7.4 Hz, 3H, $OCH_2CH_2\underline{CH_3}$).

δ_C/ppm (100 MHz, DMSO-d_6): 165.04, 162.60, 162.23, 161.89, 160.20, 156.12, 155.63, 145.07, 134.19, 133.12, 125.40, 123.32, 114.92, 114.16, 109.74, 108.02, 105.77, 99.02, 70.33, 56.05, 55.78, 21.95, 10.41.

MS = $[M+H]^+$ Calculated mass for $C_{25}H_{24}NO_9$: 482.1451. Found: 482.1444. Diff: 1.5 ppm.

11.5.3 (4-Nitrophenyl)-2-butoxy-4-(2,4-dimethoxybenzoyl)oxybenzoate (K.4)

White powder. Yield: 0.104 g, 32%. RF: 0.15 (100% dichloromethane)

T_{CrI} 139 ˚C T_{N_FI} (28 ˚C)

ν_{max}/cm^{-1}: 2940 (-CH_2), 2874 (-CH_2), 1740 (COOR), 1610 (Ar C=C), 1521 (NO_2), 1238 (C-O-C).

δ_H/ppm (400 MHz, DMSO-d_6): 8.36 (d, *J* = 9.3 Hz, 2H, Ar-H), 8.04 (dd, *J* = 8.6, 1.1 Hz, 1H, Ar-H), 8.00 (dd, *J* = 8.8, 1.1 Hz, 1H, Ar-H), 7.56 (d, *J* = 9.0 Hz, 2H, Ar-H), 7.16 (d, *J* = 1.7 Hz, 1H, Ar-H), 6.97 (dd, *J* = 8.5, 2.0 Hz, 1H, Ar-H), 6.73 (d, *J* = 2.2 Hz, 1H, Ar-H), 6.69 (dd, *J* = 8.8, 1.6 Hz, 1H, Ar-H), 4.10 (t, *J* = 6.2 Hz, 2H, $O\underline{CH_2}CH_2CH_2CH_3$), 3.94 – 3.86 (m, 6H, 2× $O\underline{CH_3}$), 1.72 (p, *J* = 6.6 Hz, 2H, $OCH_2\underline{CH_2}CH_2CH_3$), 1.46 (h, *J* = 7.4 Hz, 2H, $OCH_2CH_2\underline{CH_2}CH_3$), 0.89 (t, *J* = 7.9 Hz, 3H, $OCH_2CH_2CH_2\underline{CH_3}$).

δ_C/ppm (100 MHz, DMSO-d_6): 165.03, 162.16, 161.92, 161.87, 160.43, 156.31, 134.17, 133.28, 127.56, 119.13, 114.37, 114.14, 112.83, 112.60, 109.69, 108.01, 105.74, 99.00, 68.53, 56.03, 55.76, 30.51, 18.59, 13.58.

EA: Calculated: C 63.03%, H 5.09%, N 2.83%. Found: C 63.23%, H 4.76%, N 2.78%.

11.5.4 (4-Nitrophenyl)-2-pentoxy-4-(2,4-dimethoxybenzoyl)oxybenzoate (K.5)

White powder. Yield: 0.147 g, 49%. RF: 0.17 (100% dichloromethane)

T_{CrI} 141 ˚C T_{N_FI} (16 ˚C)

ν_{max}/cm^{-1}: 2949 (-CH_2), 2870 (-CH_2), 1737 (COOR), 1609 (Ar C=C), 1520 (NO_2), 1236 (C-O-C).

δ_H/ppm (400 MHz, DMSO-d_6): 8.36 (d, *J* = 8.0 Hz, 2H, Ar-H), 8.03 (d, *J* = 8.5 Hz, 1H, Ar-H), 8.00 (d, *J* = 8.7 Hz, 1H, Ar-H), 7.57 (d, *J* = 9.4 Hz, 2H, Ar-H), 7.15 (d, *J* = 2.1 Hz, 1H, Ar-H), 6.97 (dd, *J* = 8.5, 2.1 Hz, 1H, Ar-H), 6.73 (d, *J* = 2.2 Hz, 1H, Ar-H), 6.69 (dd, *J* = 8.8, 1.6 Hz, 1H, Ar-H), 4.09 (t, *J* = 6.3 Hz, 2H, $O\underline{CH_2}CH_2CH_2CH_2CH_3$), 3.93 – 3.86 (m, 6H, 2× $O\underline{CH_3}$), 1.73 (p, *J* = 6.5 Hz, 2H, $OCH_2\underline{CH_2}CH_2CH_2CH_3$), 1.41 (p, *J* = 6.2 Hz, 2H, $OCH_2CH_2\underline{CH_2}CH_2CH_3$), 1.30 (h, *J* = 7.2 Hz, 2H, $OCH_2CH_2CH_2\underline{CH_2}CH_3$), 0.82 (t, *J* = 7.2 Hz, 3H, $OCH_2CH_2\,CH_2CH_2\underline{CH_3}$).

δ_C/ppm (100 MHz, DMSO-d_6): 165.02, 162.62, 162.21, 161.87, 160.17, 156.11, 155.63, 145.04, 134.16, 133.09, 125.35, 123.27, 114.92, 114.14, 109.72, 108.00, 105.73, 98.99, 68.81, 56.03, 55.76, 28.16, 27.58, 21.74, 13.87.

EA: Calculated: C 63.65%, H 5.34%, N 2.75%. Found: C 63.55%, H 4.92%, N 2.79%.

11.5.5 (4-Nitrophenyl)-2-hexoxy-4-(2,4-dimethoxybenzoyl)oxybenzoate (K.6)

White powder. Yield: 0.131 g, 38%. RF: 0.17 (100% dichloromethane)

T_{CrI} 114 ˚C T_{NFI} (10 ˚C)

v_{max}/cm^{-1}: 2950 (-CH_2), 2870 (-CH_2), 1736 (COOR), 1608 (Ar C=C), 1519 (NO_2), 1232 (C-O-C).

δ_H/ppm (400 MHz, DMSO-d_6): 8.36 (d, *J* = 8.5 Hz, 2H, Ar-H), 8.01 (t, *J* = 9.7 Hz, 1H, Ar-H), 7.57 (d, *J* = 8.4 Hz, 2H, Ar-H), 7.15 (s, 1H, Ar-H), 6.97 (d, *J* = 8.6 Hz, 1H, Ar-H), 6.73 (s, 1H, Ar-H), 6.69 (d, *J* = 9.2 Hz, 1H, Ar-H), 6.73 (d, *J* = 2.3 Hz, 1H, Ar-H), 6.69 (dd, *J* = 8.8, 2.4 Hz, 1H, Ar-H), 4.10 (t, *J* = 6.3 Hz, 2H, O$\underline{CH_2}CH_2CH_2CH_2CH_2CH_3$), 3.92 – 3.86 (m, 6H, 2× O$\underline{CH_3}$), 1.72 (p, *J* = 7.3 Hz, 2H, $OCH_2\underline{CH_2}CH_2CH_2CH_2CH_3$), 1.42 (p, *J* = 7.8 Hz, 2H, $OCH_2CH_2\underline{CH_2}CH_2CH_2CH_3$), 1.29 – 1.08 (m, 4H, $OCH_2CH_2CH_2\underline{CH_2CH_2}CH_3$), 0.80 (t, *J* = 6.8 Hz, 3H, $OCH_2CH_2CH_2CH_2CH_2\underline{CH_3}$).

δ_C/ppm (100 MHz, DMSO-d_6): 165.01, 162.64, 162.19, 161.86, 160.14, 156.09, 155.63, 145.02, 134.15, 133.06, 125.34, 123.25, 114.93, 114.13, 109.72, 107.99, 105.72, 98.99, 68.81, 56.02, 55.75, 30.83, 28.44, 25.05, 22.03, 13.81.

EA: Calculated: C 64.24%, H 5.58%, N 2.68%. Found: C 64.06%, H 5.69%, N 2.61%.

11.5.6 (4-Nitrophenyl)-2-heptoxy-4-(2,4-dimethoxybenzoyl)oxybenzoate (K.7)

White powder. Yield: 0.143 g, 48%. RF: 0.23 (100% dichloromethane)

T_{CrI} 80 ˚C T_{NFI} (14 ˚C)

v_{max}/cm^{-1}: 2940 (-CH_2), 2869 (-CH_2), 1742 (COOR), 1612 (Ar C=C), 1524 (NO_2), 1236 (C-O-C).

δ_H/ppm (400 MHz, DMSO-d_6): 8.36 (d, *J* = 9.5 Hz, 2H, Ar-H), 8.12 – 7.92 (m, 2H, Ar-H), 7.57 (d, *J* = 9.4 Hz, 2H, Ar-H), 7.16 (d, *J* = 2.2 Hz, 1H, Ar-H), 6.98 (dd, *J* = 8.6, 2.1 Hz, 1H, Ar-H), 6.73 (d, *J* = 2.4 Hz, 1H, Ar-H), 6.69 (dd, *J* = 8.8, 2.4 Hz, 1H, Ar-H), 4.10 (t, *J* = 5.8 Hz, 2H, O$\underline{CH_2}CH_2CH_2CH_2CH_2CH_3$), 3.93 – 3.86 (m, 6H, 2× O$\underline{CH_3}$), 1.72 (p, *J* = 6.8 Hz, 2H, $OCH_2\underline{CH_2}CH_2CH_2CH_2CH_2CH_3$), 1.41 (p, *J* = 7.7 Hz, 2H, $OCH_2CH_2\underline{CH_2}CH_2CH_2CH_2CH_3$), 1.34 – 1.10 (m, 6H, $OCH_2CH_2CH_2\underline{CH_2CH_2CH_2}CH_3$), 0.80 (t, *J* = 7.1 Hz, 3H, $OCH_2CH_2CH_2CH_2CH_2CH_2\underline{CH_3}$).

δ_C/ppm (100 MHz, DMSO-d_6): 165.02, 162.71, 162.20, 161.87, 160.13, 156.10, 155.64, 145.01, 134.15, 133.07, 125.34, 123.24, 114.95, 114.14, 109.72, 107.99, 105.73, 98.99, 68.81, 56.02, 55.75, 31.22, 28.51, 28.32, 25.37, 21.98, 13.89.

EA: Calculated: C 64.80%, H 5.81%, N 2.61%. Found: C 65.26%, H 5.43%, N 2.51%.

11.6 (3-Fluoro-4-nitrophenyl)-2-alkyloxy-4-(2,4-dimethoxybenzoyl)oxybenzoates

Tables SI19. Quantities of reagents used to synthesise (3-fluoro-4-nitrophenyl)-2-alkoxy-4-(2,4-methoxybenzoyl)oxybenzoates.

m	(**10.3**)	3-Fluoro-4-nitrophenol	DCC
2	0.200 g, 4.59×10^{-4} mol	0.058 g, 4.17×10^{-4} mol	0.107 g, 5.21×10^{-4} mol

3	0.240 g, 6.66×10^{-4} mol	0.095 g, 6.05×10^{-4} mol	0.156 g, 7.56×10^{-4} mol
4	0.300 g, 7.97×10^{-4} mol	0.114 g, 7.24×10^{-4} mol	0.186 g, 90.5×10^{-4} mol
5	0.250 g, 6.43×10^{-4} mol	0.092 g, 5.85×10^{-4} mol	0.151 g, 7.30×10^{-4} mol
6	0.300 g, 7.45×10^{-4} mol	0.106 g, 6.77×10^{-4} mol	0.174 g, 8.46×10^{-4} mol
7	0.250 g, 6.00×10^{-4} mol	0.086 g, 5.46×10^{-4} mol	0.140 g, 6.82 ×10^{-4} mol

11.6.1 (3-Fluoro-4-nitrophenyl)-2-ethoxy-4-(2,4-dimethoxybenzoyl)oxybenzoate (L.2)

White powder. Yield: 0.055 g, 24%. RF: 0.09 (100% dichloromethane)

T_{CrI} 154 ˚C T_{N_FI} (77 ˚C)

ν_{max}/cm^{-1}: 3060 (Aryl-H), 2973 (-CH_3), 2932 (-CH_2), 2843 (-CH_2), 1743 (COOR), 1607 (Ar C=C), 1524 (NO_2), 1269 (C-O-C), 1215 (C-F).

δ_H/ppm (400 MHz, DMSO-d_6): 8.30 (t, *J* = 8.9 Hz, 1H, Ar-H), 8.05 (d, *J* = 8.6 Hz, 1H, Ar-H), 8.00 (d, *J* = 8.7 Hz, 1H, Ar-H), 7.71 (dd, *J* = 12.0, 2.4 Hz, 1H, Ar-H), 7.45 – 7.38 (m, 1H, Ar-H), 7.15 (d, *J* = 1.6 Hz, 1H, Ar-H), 6.98 (dd, *J* = 8.4, 2.8 Hz, 1H, Ar-H), 6.73 (d, *J* = 2.3 Hz, 1H, Ar-H), 6.69 (dd, *J* = 70.8, 1.9 Hz, 1H, Ar-H), 4.17 (q, *J* = 6.9 Hz, 2H, O$\underline{CH_2}$$CH_3$), 3.91 – 3.86 (m, 6H, 2× O$\underline{CH_3}$), 1.35 (t, *J* = 6.9 Hz, 3H, $OCH_2\underline{CH_3}$).

δ_C/ppm (100 MHz, DMSO-d_6): 165.02, 162.15, 161.86 (2× CH), 160.22, 156.24, 155.65 (d, *J* = 10.6 Hz), 153.98 (d, *J* = 262.6 Hz), 134.59 (d, *J* = 7.3 Hz), 134.16, 133.20, 127.53 (d, *J* = 2.3 Hz), 119.19 (d, *J* = 3.5 Hz), 114.51, 114.18, 112.73 (d, *J* = 23.7 Hz), 109.68, 108.12, 105.73, 98.99, 64.72, 56.02, 55.76, 14.39.

MS = $[M+Na]^+$ Calculated mass for $C_{24}H_{20}NO_9FNa$: 508.102. Found: 508.103. Diff: 0.5 ppm.

11.6.2 (3-Fluoro-4-nitrophenyl)-2-propoxy-4-(2,4-dimethoxybenzoyl)oxybenzoate (L.3)

White powder. Yield: 0.166 g, 54%. RF: 0.14 (100% dichloromethane)

T_{CrI} 125 ˚C T_{N_FI} (60 ˚C)

ν_{max}/cm^{-1}: 2969 (-CH_3), 2943 (-CH_2), 2850 (-CH_2), 1715 (COOR), 1602 (Ar C=C), 1532 (NO_2), 1287 (C-O-C), 1208 (C-F).

δ_H/ppm (400 MHz, DMSO-d_6): 8.30 (t, *J* = 8.9 Hz, 1H, Ar-H), 8.05 (d, *J* = 8.6 Hz, 1H, Ar-H), 8.00 (d, *J* = 8.7 Hz, 1H, Ar-H), 7.70 (dd, *J* = 12.0, 2.4 Hz, 1H, Ar-H), 7.40 (ddd, *J* = 9.1, 2.4, 1.2 Hz, 1H, Ar-H), 7.15 (d, *J* = 2.1 Hz, 1H, Ar-H), 6.98 (dd, *J* = 8.5, 2.1 Hz, 1H, Ar-H), 6.72 (d, *J* = 2.3 Hz, 1H, Ar-H), 6.69 (dd, *J* = 8.8, 2.3 Hz, 1H, Ar-H), 4.06 (t, *J* = 6.3 Hz, 2H, O$\underline{CH_2}$$CH_2CH_3$), 3.92 – 3.87 (m, 2× O$\underline{CH_3}$), 1.75 (h, *J* = 7.0 Hz, 2H, $OCH_2\underline{CH_2}CH_3$), 0.98 (t, *J* = 7.4 Hz, 3H, $OCH_2CH_2\underline{CH_3}$).

δ_C/ppm (100 MHz, DMSO-d_6): 165.07, 162.21, 162.00, 161.92, 160.45, 156.34, 155.71 (d, *J* = 12.1 Hz), 154.03 (d, *J* = 261.8 Hz), 134.63 (d, *J* = 7.2 Hz), 134.21, 133.34, 127.61 (d, *J* = 1.8 Hz), 119.21 (d, *J* = 3.7 Hz), 114.42, 114.18, 112.76 (d, *J* = 23.7 Hz), 109.71, 108.04, 105.79, 99.02, 70.37, 56.06, 55.79, 21.94, 10.43.

MS = $[M+Na]^+$ Calculated mass for $C_{25}H_{22}NO_9FNa$: 522.118. Found 522.120. Diff: 5 ppm.

11.6.3 (3-Fluoro-4-nitrophenyl)-2-butoxy-4-(2,4-dimethoxybenzoyl)oxybenzoate (L.4)

White powder. Yield: 0.172 g, 30%. RF: 0.16 (100% dichloromethane)

T_{CrI} 109 ˚C T_{N_FI} (42 ˚C)

ν_{max}/cm^{-1}: 2941 (-CH_2), 2875 (-CH_2), 1717 (COOR), 1601 (Ar C=C), 1527 (NO_2), 1287 (C-O-C), 1206 (C-F).

δ_H/ppm (400 MHz, DMSO-d_6): 8.31 (t, *J* = 8.9 Hz, 1H, Ar-H), 8.05 (d, *J* = 8.6 Hz, 1H, Ar-H), 8.00 (d, *J* = 8.7 Hz, 1H, Ar-H), 7.70 (dd, *J* = 12.0, 2.4 Hz, 1H, Ar-H), 7.40 (ddd, *J* = 9.0, 2.4, 1.2 Hz, 1H, Ar-H), 7.16 (d, *J* = 2.1 Hz, 1H, Ar-H), 6.98 (dd, *J* = 8.6, 2.1 Hz, 1H, Ar-H), 6.73 (d, *J* = 2.3 Hz, 1H, Ar-H), 6.69 (dd, *J* = 8.8, 2.3 Hz, 1H, Ar-H), 4.10 (t, *J* = 6.3 Hz, 2H, $O\underline{CH_2}CH_2CH_2CH_3$), 3.93 – 3.75 (m, 2× $O\underline{CH_3}$), 1.72 (p, *J* = 6.8 Hz, 2H, $OCH_2\underline{CH_2}CH_2CH_3$), 1.46 (h, *J* = 7.6 Hz, 2H, $OCH_2CH_2\underline{CH_2}CH_3$), 0.90 (t, *J* = 7.4 Hz, 3H, $OCH_2CH_2CH_2\underline{CH_3}$).

δ_C/ppm (100 MHz, DMSO-d_6): 165.03, 162.16, 161.92, 161.87, 160.43, 156.31, 155.68 (d, *J* = 11.6 Hz), 153.99 (d, *J* = 262.3 Hz), 134.17, 133.28, 127.56 (d, *J* = 1.9 Hz), 119.13 (d, *J* = 3.2 Hz), 114.37, 114.14, 112.72 (d, *J* = 23.5 Hz), 110.74, 109.69, 108.01, 105.74, 99.00, 68.53, 56.03, 55.76, 30.51, 18.59, 13.58.

MS = $[M+Na]^+$ Calculated mass for $C_{26}H_{24}NO_9FNa$: 536.133. Found: 536.135. Diff: 1.5 ppm.

11.6.4 (3-Fluoro-4-nitrophenyl)-2-pentoxy-4-(2,4-dimethoxybenzoyl)oxybenzoate (L.5)

White powder. Yield: 0.147 g, 49%. RF: 0.45 (100% dichloromethane)

T_{CrI} 118 ˚C T_{N_FI} (35 ˚C)

ν_{max}/cm^{-1}: 2943 (-CH_2), 2871 (-CH_2), 1721 (COOR), 1601 (Ar C=C), 1523 (NO_2), 1284 (C-O-C), 1207 (C-F).

δ_H/ppm (400 MHz, DMSO-d_6): 8.31 (t, *J* = 8.9 Hz, 1H, Ar-H), 8.04 (d, *J* = 8.6 Hz, 1H, Ar-H), 8.00 (d, *J* = 8.7 Hz, 1H, Ar-H), 7.69 (dd, *J* = 12.0, 2.4 Hz, 1H, Ar-H), 7.40 (ddd, *J* = 9.2, 2.4, 1.2 Hz, 1H, Ar-H), 7.16 (d, *J* = 2.1 Hz, 1H, Ar-H), 6.98 (dd, *J* = 8.6, 2.1 Hz, 1H, Ar-H), 6.73 (d, *J* = 2.3 Hz, 1H, Ar-H), 6.68 (dd, *J* = 9.1, 2.0 Hz, 1H, Ar-H), 4.09 (t, *J* = 6.3 Hz, 2H, $O\underline{CH_2}CH_2CH_2CH_2CH_3$), 3.92 – 3.84 (m, 3H, 2× $O\underline{CH_3}$), 1.73 (p, *J* = 6.4 Hz, 2H, $OCH_2\underline{CH_2}CH_2 CH_2CH_3$), 1.41 (p, *J* = 8.3 Hz, 2H, $OCH_2CH_2\underline{CH_2}CH_2CH_3$), 1.31 (h, *J* = 6.9 Hz, 2H, $OCH_2 CH_2CH_2\underline{CH_2}CH_3$), 0.83 (t, *J* = 7.2 Hz, 3H, $OCH_2CH_2CH_2CH_2\underline{CH_3}$).

δ_C/ppm (100 MHz, DMSO-d_6): 165.04, 162.17, 162.03, 161.88, 160.39, 156.31, 155.80 (d, *J* = 11.3 Hz), 154.00 (d, *J* = 262.4 Hz), 134.59 (d, *J* = 8.1 Hz), 134.17, 133.28, 127.56 (d, *J* = 2.3 Hz), 119.16 (d, *J* = 3.0 Hz), 114.42, 114.16, 112.70 (d, *J* = 23.8 Hz), 109.69, 108.02, 105.75, 99.00, 68.84, 56.03, 55.77, 28.15, 27.58, 21.75, 13.88.

MS = $[M+Na]^+$ Calculated mass for $C_{27}H_{26}NO_9FNa$: 550.149. Found: 550.148. Diff: 2 ppm.

11.6.5 (3-Fluoro-4-nitrophenyl)-2-hexoxy-4-(2,4-dimethoxybenzoyl)oxybenzoate (L.6)

White powder. Yield: 0.172 g, 48%. RF: 0.18 (100% dichloromethane)

T_{CrI} 73 ˚C T_{N_FI} (30 ˚C)

ν_{max}/cm^{-1}: 2940 (-CH_2), 2872 (-CH_2), 1743 (COOR), 1602 (Ar C=C), 1525 (NO_2), 1268 (C-O-C), 1214 (C-F).

δ_H/ppm (400 MHz, DMSO-d_6): 8.31 (t, *J* = 8.9 Hz, 1H, Ar-H), 8.03 (d, *J* = 8.6 Hz, 1H, Ar-H), 8.00 (d, *J* = 8.7 Hz, 1H, Ar-H), 7.69 (dd, *J* = 12.0, 2.4 Hz, 1H, Ar-H), 7.40 (ddd, *J* = 9.1, 2.5, 1.2 Hz, 1H, Ar-H), 7.15 (d, *J* = 2.1 Hz, 1H, Ar-H), 6.97 (dd, *J* = 8.6, 2.1 Hz, 1H, Ar-H), 6.73 (d, *J* = 2.3 Hz, 1H, Ar-H), 6.69 (dd, *J* = 8.8, 2.3 Hz, 1H, Ar-H), 4.09 (t, *J* = 6.2 Hz, 2H, $O\underline{CH_2}CH_2CH_2CH_2CH_2CH_3$), 3.94 – 3.82 (m, 2× $O\underline{CH_3}$), 1.72 (p, *J* = 6.4 Hz, 2H, $OCH_2\underline{CH_2}CH_2\,CH_2CH_2CH_3$), 1.42 (p, *J* = 7.3 Hz, 2H, $OCH_2CH_2\underline{CH_2}CH_2CH_2CH_3$), 1.33 – 1.12 (m, 4H, $OCH_2CH_2CH_2\underline{CH_2CH_2}CH_3$), 0.81 (t, *J* = 6.8 Hz, 3H, $OCH_2CH_2CH_2\,CH_2CH_2\underline{CH_3}$).

δ_C/ppm (100 MHz, DMSO-d_6): 165.02, 162.15, 162.05, 161.87, 160.35, 156.59 (d, *J* = 263.7 Hz), 156.29, 155.68 (d, *J* = 12.6 Hz), 134.58, 134.16, 133.25, 127.55 (d, *J* = 2.5 Hz), 119.14 (d, *J* = 3.7 Hz), 114.43, 114.15, 112.78 (d, *J* = 24.0 Hz), 109.68, 108.01, 105.73, 98.99, 68.84, 56.02, 55.76, 30.84, 28.43, 25.06, 22.03, 13.81.

MS = $[M+Na]^+$ Calculated mass for $C_{28}H_{28}NO_9FNa$: 564.165. Found: 564.165. Diff: 0.4 ppm.

11.6.6 (3-Fluoro-4-nitrophenyl)-2-heptoxy-4-(2,4-dimethoxybenzoyl)oxybenzoate (L.7)

White powder. Yield: 0.183 g, 60%. RF: 0.25 (100% dichloromethane)

T_{CrI} 84 ˚C T_{N_FI} (29 ˚C)

ν_{max}/cm^{-1}: 2940 (-CH_2), 2918 (-CH_2), 2857 (-CH_2), 1745 (COOR), 1603 (Ar C=C), 1526 (NO_2), 1267 (C-O-C), 1215 (C-F).

δ_H/ppm (400 MHz, DMSO-d_6): 8.32 (t, *J* = 8.9 Hz, 1H, Ar-H), 8.12 – 7.92 (m, 2H, Ar-H), 7.70 (dd, *J* = 12.0, 2.4 Hz, 1H, Ar-H), 7.41 (dt, *J* = 9.4, 1.4 Hz, 1H, Ar-H), 7.16 (d, *J* = 2.1 Hz, 1H, Ar-H), 6.98 (dd, *J* = 8.5, 2.0 Hz, 1H, Ar-H), 6.74 (d, *J* = 2.3 Hz, 1H, Ar-H), 6.71 (s, 1H, Ar-H), 4.10 (t, *J* = 6.2 Hz, 2H, $O\underline{CH_2}CH_2CH_2CH_2CH_2CH_3$), 3.93 – 3.86 (m, 6H, 2× OCH_3), 1.73 (p, *J* = 6.4 Hz, 2H, $CH_2\underline{CH_2}CH_2CH_2CH_2CH_3$), 1.43 (p, *J* = 7.2 Hz, 2H, $OCH_2CH_2\,\underline{CH_2}CH_2CH_2CH_3$), 1.34 – 1.13 (m, 6H, $OCH_2CH_2\underline{CH_2CH_2CH_2}CH_3$), 0.81 (t, *J* = 6.7 Hz, 3H¸$OCH_2CH_2CH_2CH_2CH_2\underline{CH_3}$).

δ_C/ppm (100 MHz, DMSO-d_6): 165.03, 162.17, 162.13, 161.88, 160.35, 156.30, 155.76 (d, *J* = 11.5 Hz), 153.99 (d, *J* = 263.7 Hz), 134.57 (d, *J* = 6.9 Hz), 134.17, 133.27, 127.55 (d, *J* = 1.7 Hz), 119.12 (d, *J* = 3.8 Hz), 114.45, 114.16, 112.65 (d, *J* = 23.7 Hz), 109.69, 108.02, 105.74, 99.00, 68.85, 56.03, 55.76, 31.24, 28.50, 28.34, 25.39, 21.99, 13.89.

MS = $[M+H]^+$ Calculated mass for $C_{29}H_{31}NO_9F$: 556.1983. Found: 556.1985. Diff: 0.4 ppm.

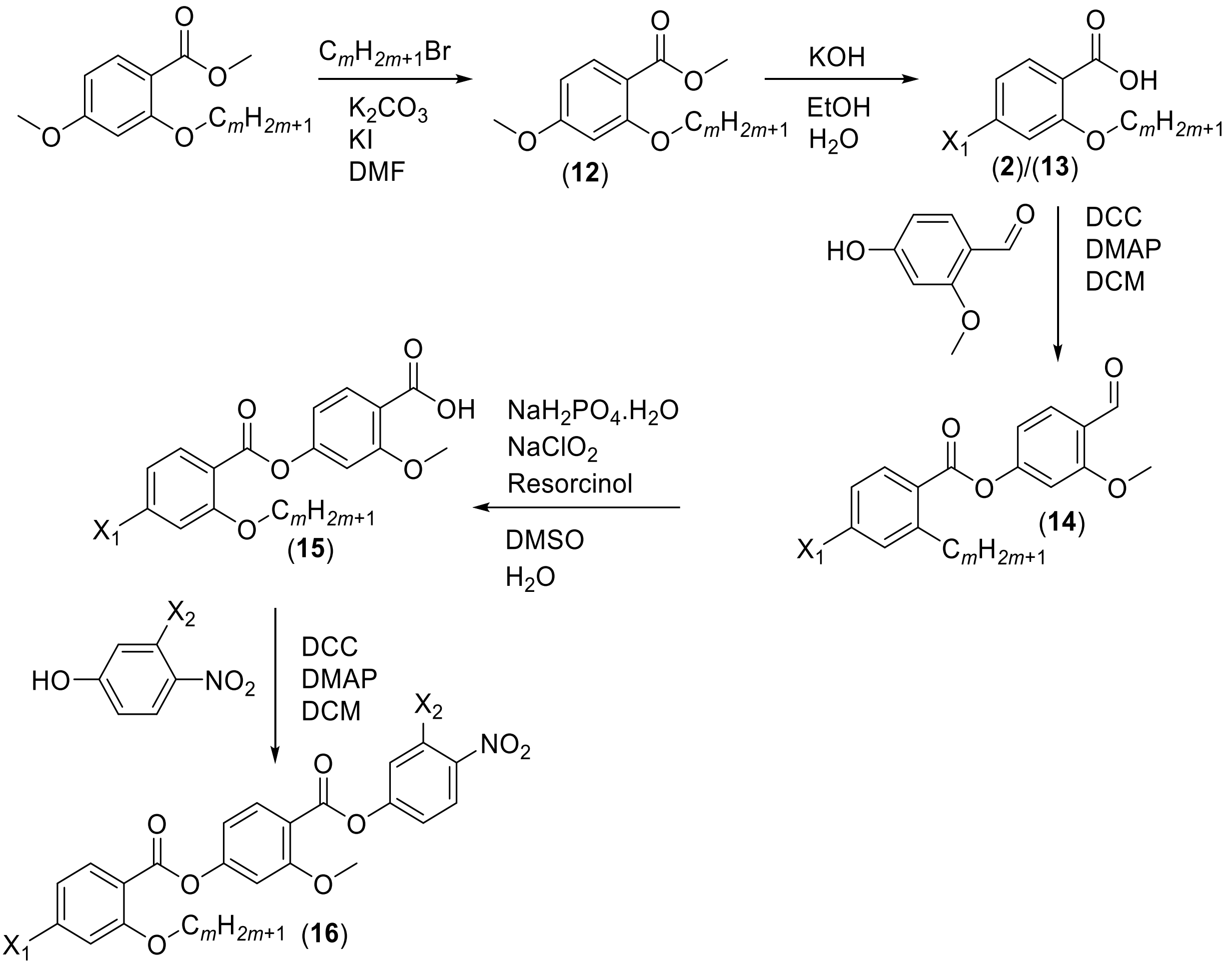

E.*m* => X_1 = Me, X_2 = H
F.*m* => X_1 = Me, X_2 = F
I.*m* => X_1 = OMe, X_2 = H
J.*m* => X_1 = OMe, X_2 = F

Figure SI3. Synthetic scheme for the E.*m*, F.*m*, I.*m*, J.*m* series. The compounds were synthesised using a five-step reaction with the final step being a Steglich esterification.

Methyl 2-alkoxy-4-methoxybenzoates (12)

To a pre-dried flask flushed with argon and fitted with a condenser, methyl 4-methoxysilicylate (1 eq, 5.00 g, 0.0274 mol), potassium iodide (except *m* = 2) (1.1 eq, 5.00 g, 0.0301 mol), and potassium carbonate (2 eq, 7.57 g, 0.0548 mol) were combined in DMF (80 mL). To the mixture, the appropriate 1-bromoalkane (1.1 eq) was added (or in the case of *m* = 2, iodoethane (1 eq, 2.20 mL, 4.27 g, 0.0274 mol) and stirred at 90°C overnight. The quantities of the 1-bromoalkanes used in each reaction are listed in **Table SI20**. The extent of the reaction was monitored by TLC using a suitable solvent system (RF values quoted in the product data). The reaction mixture was cooled to room temperature and poured into water (150 mL). The resulting suspension was extracted with ethyl acetate (2 x 250 mL). The organic fractions were combined, washed with water (3 x 100 mL) and dried over anhydrous magnesium sulfate. The magnesium sulfate was removed using vacuum filtration and the solvent evaporated under vacuum to leave a yellow oil or solid. The product was carried forwards without any further purification.

Table SI20. Quantities of 1-bromoalkanes used in the syntheses of the methyl 2-alkoxy-4-methoxybenzoates.

m	1-Bromoalkane
3	2.73 mL, 3.70 g, 0.0301 mol
4	3.23 mL, 4.12 g, 0.0301 mol
5	3.73 mL, 4.55 g, 0.0301 mol
6	4.23 mL, 4.97 g, 0.0301 mol
7	4.73 mL, 5.39 g, 0.0301 mol
8	5.20 mL, 5.81 g, 0.0301 mol
9	5.75 mL, 6.24 g, 0.0301 mol

12.1 Methyl 2-ethoxy-4-methoxybenzoate

Yellow solid. Yield: 4.36 g, 75.6 %. RF: 0.559 (40 % ethyl acetate:60 % 40:60 petroleum ether). M.P = 47 °C

ν_{max}/cm^{-1}: 2982, 2941, 2842, 1726, 1688, 1669, 1603, 1575, 1505, 1440, 1423, 1393, 1370, 1326, 1256, 1206, 1192, 1132, 1111, 1090, 1029, 982, 970, 893, 847, 822, 770, 728, 695, 652, 625, 585, 540, 469

δ_H/ppm (400 MHz, $CDCl_3$): 7.83 (1 H, d, J 8.4 Hz, Ar-H), 6.47 (2 H, m, Ar-H), 4.08 (2 H, q, J 7.0 Hz, O-$\underline{CH_2}$-CH_3), 3.84 (3 H, s, (C=O)-O-$\underline{CH_3}$), 3.82 (3 H, s, O-$\underline{CH_3}$), 1.46 (3 H, t, J 7.0 Hz, O-CH_2-$\underline{CH_3}$)

δ_C/ppm (100 MHz, $CDCl_3$): 166.21, 164.09, 160.71, 133.73, 112.65, 104.65, 100.04, 64.60, 55.43, 51.58, 14.66

12.2 Methyl 4-methoxy-2-propoxybenzoate

Yellow oil. Yield: 5.19 g, 84.5 %. RF: 0.639 (40 % ethyl acetate:60 % 40:60 petroleum ether).

ν_{max}/cm^{-1}: 2848, 2878, 2840, 1724, 1697, 1607, 1575, 1505, 1436, 1390, 1325, 1297, 1248, 1203, 1170, 1138, 1085. 1038, 994, 965, 932, 833, 768, 698, 623, 600, 462

δ_H/ppm (400 MHz, $CDCl_3$): 7.80 (1 H, d, J 8.5 Hz, Ar-H), 6.43 (2 H, m, Ar-H), 3.93 (2 H, t, J 6.5 Hz, O-$\underline{CH_2}$-CH_2-), 3.82 (3 H, s, (C=O)-O-$\underline{CH_3}$), 3.79 (3 H, s, O-$\underline{CH_3}$), 1.84 (2 H, tq, J 6.5 Hz, 7.4 Hz, O-CH_2-$\underline{CH_2}$-CH_3), 1.05 (3 H, t, J 7.4 Hz, O-CH_2-CH_2-$\underline{CH_3}$)

δ_C/ppm (100 MHz, $CDCl_3$): 166.29, 164.09, 160.83, 133.73, 112.55, 104.59, 99.80, 70.32, 55.38, 51.51, 22.51, 10.52

12.3 Methyl 2-butoxy-4-methoxybenzoate

Yellow oil. Yield: 5.19 g, 79.5 %. RF: 0.611 (40 % ethyl acetate:60 % 40:60 petroleum ether).

ν_{max}/cm^{-1}: 2955, 2873, 2840, 1725, 1697, 1669, 1607, 1575, 1505, 1436, 1389, 1325, 1298, 1248, 1203, 1169, 1138, 1086, 1037, 1010, 965, 951, 833, 768, 698, 624, 600, 462

δ_H/ppm (400 MHz, $CDCl_3$): 7.81 (1 H, d, J 9.1 Hz, Ar-H), 6.44 (2 H, m, Ar-H), 3.98 (2 H, t, J 6.5 Hz, O-$\underline{CH_2}$-CH_2-), 3.82 (3 H, s, (C=O)-O-$\underline{CH_3}$), 3.79 (3 H, s, O-$\underline{CH_3}$), 1.79 (2 H, tt, J 6.5 Hz, 7.4 Hz, O-CH_2-$\underline{CH_2}$-CH_2-), 1.50 (2 H, sext, J 7.4 Hz, O-CH_2-CH_2-$\underline{CH_2}$-CH_3), 0.96 (3 H, t, J 7.4 Hz, O-CH_2-CH_2-CH_2-$\underline{CH_3}$)

δ_C/ppm (100 MHz, $CDCl_3$): 166.28, 164.09, 160.84, 133.74, 112.56, 104.55, 99.80, 68.55, 55.38, 51.51, 31.16, 19.19, 13.81

12.4 Methyl 4-methoxy-2-pentoxybenzoate

Yellow solid. Yield: 5.40 g, 78.1 %. RF: 0.667 (40 % ethyl acetate:60 % 40:60 petroleum ether).
M.P = 46 °C

ν_{max}/cm^{-1}: 2941, 2871, 1724, 1694, 1669, 1606, 1576, 1505, 1437, 1394, 1332, 1298, 1253, 1206, 1190, 1170, 1132, 1088, 1033, 1017, 967, 952, 837, 815, 773, 932, 695, 652, 625, 591, 460

δ_H/ppm (400 MHz, $CDCl_3$): 7.84 (1 H, d, J 8.5 Hz, Ar-H), 6.46 (2 H, m, Ar-H), 3.99 (2 H, t, J 6.6 Hz, O-$\underline{CH_2}$-CH_2-), 3.84 (3 H, s, (C=O)-O-$\underline{CH_3}$), 3.83 (3 H, s, O-$\underline{CH_3}$), 1.84 (2 H, tt, J 6.6 Hz, 7.2 Hz, O-CH_2-$\underline{CH_2}$-CH_2-), 1.43 (4 H, m, O-CH_2-CH_2-$\underline{CH_2}$-$\underline{CH_2}$-CH_3), 0.93 (3 H, t, J 7.2 Hz, O-CH_2-CH_2-CH_2-CH_2-$\underline{CH_3}$)
δ_C/ppm (100 MHz, $CDCl_3$): 166.39, 164.09, 160.83, 133.79, 112.63, 104.57, 99.87, 68.93, 55.44, 51.58, 28.80, 28.13, 22.41, 14.05

12.5 Methyl 2-hexyloxy-4-methoxybenzoate

Yellow oil. Yield: 5.51 g, 75.5 %. RF: 0.611 (40 % ethyl acetate:60 % 40:60 petroleum ether).
ν_{max}/cm^{-1}: 2950, 2859, 1726, 1697, 1607, 1575, 1505, 1434, 1389, 1325, 1297, 1250, 1204, 1169, 1138, 1086, 1039, 964, 916, 835, 768, 699, 625, 600, 464
δ_H/ppm (400 MHz, $CDCl_3$): 7.84 (1 H, d, J 8.5 Hz, Ar-H), 6.47 (2 H, m, Ar-H), 4.00 (2 H, t, J 6.5 Hz, O-$\underline{CH_2}$-CH_2-), 3.85 (3 H, s, (C=O)-O-$\underline{CH_3}$), 3.83 (3 H, s, O-$\underline{CH_3}$), 1.83 (2 H, tt, J 6.5 Hz, 7.2 Hz, O-CH_2-$\underline{CH_2}$-CH_2-), 1.50 (2 H, m, O-CH_2-CH_2-$\underline{CH_2}$-CH_2-), 1.34 (4 H, m, O-CH_2-CH_2-CH_2-$\underline{CH_2}$-$\underline{CH_2}$-CH_3), 0.91 (3 H, t, J 7.0 Hz, O-CH_2-CH_2-CH_2-CH_2-CH_2-$\underline{CH_3}$)
δ_C/ppm (100 MHz, $CDCl_3$): 166.42, 164.09, 160.82, 133.80, 112.65, 104.57, 99.87, 68.95, 55.44, 51.59, 31.54, 29.08, 25.65, 22.61, 14.04

12.6 Methyl 2-heptyloxy-4-methoxybenzoate

Yellow oil. Yield: 6.44 g, 83.8 %. RF: 0.675 (40 % ethyl acetate:60 % 40:60 petroleum ether).
ν_{max}/cm^{-1}: 2929, 2857, 1726, 1697, 1670, 1607, 175, 1505, 1441, 1389, 1325, 1297, 1249, 1203, 1169, 1138, 1086, 1037, 966, 833, 768, 725, 698, 625, 599, 461
δ_H/ppm (400 MHz, $CDCl_3$): 7.83 (1 H, d, J 8.5 Hz, Ar-H), 6.46 (2 H, m, Ar-H), 3.99 (2 H, t, J 6.6 Hz, O-$\underline{CH_2}$-CH_2-), 3.84 (3 H, s, (C=O)-O-$\underline{CH_3}$), 3.83 (3 H, s, O-$\underline{CH_3}$), 1.83 (2 H, tt, J 6.6 Hz, 7.0 Hz, O-CH_2-$\underline{CH_2}$-CH_2-), 1.48 (2 H, m, O-CH_2-CH_2-$\underline{CH_2}$-CH_2-), 1.34 (6 H, m, O-CH_2-CH_2-CH_2-$\underline{CH_2}$-$\underline{CH_2}$-$\underline{CH_2}$-CH_3), 0.89 (3 H, t, J 7.0 Hz, O-CH_2-CH_2-CH_2-CH_2-CH_2-CH_2-$\underline{CH_3}$)
δ_C/ppm (100 MHz, $CDCl_3$): 166.39, 164.09, 160.82, 133.79, 112.64, 104.56, 99.87, 68.94, 55.43, 51.57, 31.79, 29.12, 29.01, 25.92, 22.62, 14.09

2-Alkoxy-4-methoxybenzoic acids (13)

To a pre-dried flask flushed with argon and fitted with a condenser, potassium hydroxide (3 eq) was added to water. **Compound 12** (1 eq) was solubilised in EtOH, added to the flask and the resultant mixture stirred at reflux overnight. The quantities of the reagents used in each reaction are listed in **Table SI21**. The extent of the reaction was monitored by TLC using an appropriate solvent system (RF values quoted in the product data). The reaction mixture was cooled to room temperature and the pH of the mixture was adjusted to 1 using 32% hydrochloric acid (25 mL). If a white solid precipitated after acidification it was collected by vacuum filtration. If no precipitate formed the reaction mixture was extracted with ethyl acetate (2 x 200 mL). The organic layers were combined, washed with water (2 x 100 mL) and dried over anhydrous magnesium sulfate. The magnesium sulfate was removed using vacuum filtration and the solvent evaporated under vacuum to leave a white or off-white solid. The product was carried forwards without any further purification (*m* = 5, 7, 8, 9 were recrystallised from hot ethanol (75 mL) to give a white solid).

Table SI21. Quantities of reagents used in the syntheses of the 2-alkoxy-4-methoxybenzoic acids.

m	(**12**)	Ethanol	Potassium Hydroxide	Water
2	5.00 g, 0.0238 mol	50 mL	4.00 g, 0.0714 mol	30 mL
3	5.00 g, 0.0223 mol	70 mL	3.75 g, 0.0669 mol	35 mL
4	5.00 g, 0.0210 mol	70 mL	3.53 g, 0.0630 mol	35 mL
5	5.20 g, 0.0206 mol	70 mL	3.47 g, 0.0618 mol	35 mL

6	3.78 g, 0.0142 mol	30 mL	2.39 g, 0.0426 mol	50 mL
7	6.00 g, 0.0214 mol	70 mL	3.60 g, 0.0642 mol	40 mL
8	6.50 g, 0.0221 mol	75 mL	3.72 g, 0.0663 mol	40 mL
9	7.00 g, 0.0227 mol	80 mL	3.82 g, 0.0681 mol	50 mL

13.1 2-Ethoxy-4-methoxybenzoic acid

White solid. Yield: 4.30 g, 92.0 %. RF: 0.306 (40 % ethyl acetate:60 % 40:60 petroleum ether). M.P = 121 °C

v_{max}/cm^{-1}: 2985, 2874, 1666, 613, 1569, 1506, 1451, 1413, 1386, 1311, 1275, 1254, 1202, 1174, 1150, 1114, 1094, 1034, 918, 890, 831, 815, 793, 765, 736, 690, 632, 616, 578, 490, 464, 410

δ_H/ppm (400 MHz, DMSO-d_6): 12.07 (1 H, s, OH), 7.68 (1 H, d, J 8.6 Hz, Ar-H), 6.59 (1 H, d, J 2.2 Hz, Ar-H), 6.56 (1 H, dd, J 8.6 Hz, 2.2 Hz, Ar-H), 4.08 (2 H, q, J 7.0 Hz, O-$\underline{CH_2}$-CH_3), 3.80 (3 H, s, O-$\underline{CH_3}$), 1.32 (3 H, t, J 7.0 Hz, O-CH_2-$\underline{CH_3}$)

δ_C/ppm (100 MHz, DMSO-d_6): 166.97, 163.96, 160.22, 133.60, 113.52, 105.72, 100.24, 64.58, 55.92, 14.97

13.2 4-Methoxy-2-propoxybenzoic acid

White solid. Yield: 3.74 g, 79.8 %. RF: 0.541 (100 % ethyl acetate). M.P = 88 °C

v_{max}/cm^{-1}: 2972, 2881, 1679, 1660, 1601, 1570, 1507, 1462, 1446, 1409, 1393, 1333, 1309, 1242, 1213, 1180, 1146, 1091, 1062, 1030, 1019, 993, 926, 834, 817, 793, 779, 698, 637, 594, 570, 490, 456, 417

δ_H/ppm (400 MHz, DMSO-d_6): 12.08 (1 H, s, OH), 7.68 (1 H, d, J 8.6 Hz, Ar-H), 6.58 (1 H, d, J 2.3 Hz, Ar-H), 6.55 (1 H, dd, J 8.6 Hz, 2.3 Hz, Ar-H), 3.98 (2 H, t, J 6.4 Hz, O-$\underline{CH_2}$-CH_2-), 3.80 (3 H, s, O-$\underline{CH_3}$), 1.72 (2 H, tq, J 6.4 Hz, 7.3 Hz, O-CH_2-$\underline{CH_2}$-CH_3), 0.99 (3 H, t, J 7.3 Hz, O-CH_2-CH_2-$\underline{CH_3}$)

δ_C/ppm (100 MHz, DMSO-d_6): 167.03, 163.97, 160.42, 133.59, 113.44, 105.68, 100.06, 70.19, 55.92, 22.46, 10.92

13.3 2-Butoxy-4-methoxybenzoic acid

White solid. Yield: 3.33 g, 70.7 %. RF: 0.568 (100 % ethyl acetate). M.P = 83 °C

v_{max}/cm^{-1}: 2942, 2872, 1681, 1657, 1600, 1570, 1506, 1446, 1410, 1393, 1331, 1305, 1243, 1212, 1178, 1146, 1091, 1064, 1029, 1011, 988, 959, 938, 835, 812, 795, 777, 739, 694, 652, 640, 594, 571, 524, 474, 458, 418

δ_H/ppm (400 MHz, DMSO-d_6): 12.07 (1 H, s, OH), 7.68 (1 H, d, J 8.6 Hz, Ar-H), 6.59 (1 H, d, J 2.3 Hz, Ar-H), 6.55 (1 H, dd, J 8.6 Hz, 2.3 Hz, Ar-H), 4.02 (2 H, t, J 6.3 Hz, O-$\underline{CH_2}$-CH_2-), 3.80 (3 H, s, O-$\underline{CH_3}$), 1.69 (2 H, tt, J 6.3 Hz, 7.4 Hz, O-CH_2-$\underline{CH_2}$-CH_2-), 1.46 (2 H, sext, J 7.4 Hz, O-CH_2-CH_2-$\underline{CH_2}$-CH_3), 0.92 (3 H, t, J 7.4 Hz, O-CH_2-CH_2-CH_2-$\underline{CH_3}$)

δ_C/ppm (100 MHz, DMSO-d_6): 167.02, 163.96, 160.41, 133.56, 113.47, 105.68, 100.07, 68.42, 55.93, 31.12, 19.12, 14.14

13.4 4-Methoxy-2-pentoxybenzoic acid

White solid. Yield: 2.21 g, 45.0 %. RF: 0.568 (100 % ethyl acetate). M.P = 75 °C

v_{max}/cm^{-1}: 2942, 2866, 2547, 1686, 1659, 1605, 1569, 1505, 1450, 1430, 1405, 1391, 1307, 1271, 1252, 1206, 1177, 1150, 1125, 1110, 1094, 1039, 1021, 940, 836, 824, 792, 771, 736, 694, 620, 584 ,499, 460, 432

δ_H/ppm (400 MHz, DMSO-d_6): 12.06 (1 H, s, OH), 7.68 (1 H, d, J 8.6 Hz, Ar-H), 6.59 (1 H, d, J 2.3 Hz, Ar-H), 6.55 (1 H, dd, J 8.6 Hz, 2.3 Hz, Ar-H), 4.01 (2 H, t, J 6.4 Hz, O-$\underline{CH_2}$-CH_2-), 3.80 (3 H, s, O-$\underline{CH_3}$), 1.71 (2 H, tt, J 6.4 Hz, 6.8 Hz, O-CH_2-$\underline{CH_2}$-CH_2-), 1.37 (4 H, m, O-CH_2-CH_2-$\underline{CH_2}$-$\underline{CH_2}$-CH_3), 0.89 (3 H, t, J 7.2 Hz, O-CH_2-CH_2-CH_2-CH_2-$\underline{CH_3}$)

δ_C/ppm (100 MHz, DMSO-d_6): 167.02, 163.96, 160.40, 133.55, 113.48, 105.69, 100.09, 68.73, 55.93, 28.71, 28.07, 22.27, 14.40

13.5 2-Hexyloxy-4-methoxybenzoic acid

Off-white solid. Yield: 3.23 g, 90.1 %. RF: 0.378 (40 % ethyl acetate:60 % 40:60 petroleum ether). M.P = 57 °C

v_{max}/cm^{-1}: 2948, 2867, 1680, 1663, 1601, 1571, 1507, 1449, 1449, 1411, 1391, 1331, 1243, 1210, 1176, 1147, 1092, 979, 948, 834, 814, 795, 777, 728, 694, 654, 642, 626, 595, 570, 523, 489, 461, 437

δ_H/ppm (400 MHz, DMSO-d_6): 11.51 (1 H, s, OH), 7.68 (1 H, d, J 8.6 Hz, Ar-H), 6.59 (1 H, d, J 2.3 Hz, Ar-H), 6.55 (1 H, dd, J 8.6 Hz, 2.3 Hz, Ar-H), 4.01 (2 H, t, J 6.4 Hz, O-$\underline{CH_2}$-CH_2-), 3.80 (3 H, s, O-$\underline{CH_3}$), 1.70 (2 H, tt, J 6.4 Hz, 6.8 Hz, O-CH_2-$\underline{CH_2}$-CH_2-), 1.44 (2 H, m, O-CH_2-CH_2-$\underline{CH_2}$-CH_2-), 1.30 (4 H, m, O-CH_2-CH_2-CH_2-$\underline{CH_2}$-$\underline{CH_2}$-CH_3), 0.87 (3 H, t, J 7.1 Hz, O-CH_2-CH_2-CH_2-CH_2-CH_2-$\underline{CH_3}$)

δ_C/ppm (100 MHz, DMSO-d_6): 167.00, 163.96, 160.42, 133.57, 113.45, 105.67, 100.06, 68.73, 55.91, 31.37, 28.98, 25.51, 22.53, 14.34

13.6 2-Heptyloxy-4-methoxybenzoic acid

White solid. Yield: 2.94 g, 51.6 %. RF: 0.410 (40 % ethyl acetate:60 % 40:60 petroleum ether). M.P = 60 °C

v_{max}/cm^{-1}: 2938, 2853, 1659, 1638, 1604, 1568, 1505, 1447, 1410, 1392, 1382, 1310, 1273, 1247, 1202, 1172, 1148, 1110, 1094, 1064, 1034, 1006, 929, 861, 828, 796, 770, 724, 693, 648, 633, 615, 584, 526, 465, 407

δ_H/ppm (400 MHz, DMSO-d_6): 12.05 (1 H, s, OH), 7.68 (1 H, d, J 8.6 Hz, Ar-H), 6.59 (1 H, d, J 2.3 Hz, Ar-H), 6.55 (1 H, dd, J 8.6 Hz, 2.3 Hz, Ar-H), 4.01 (2 H, t, J 6.4 Hz, O-$\underline{CH_2}$-CH_2-), 3.80 (3 H, s, O-$\underline{CH_3}$), 1.70 (2 H, tt, J 6.4 Hz, 6.8 Hz, O-CH_2-$\underline{CH_2}$-CH_2-), 1.44 (2 H, tt, 6.8 Hz, 7.0 Hz, O-CH_2-CH_2-$\underline{CH_2}$-CH_2-), 1.28 (6 H, m, O-CH_2-CH_2-CH_2-$\underline{CH_2}$-$\underline{CH_2}$-$\underline{CH_2}$-CH_3), 0.86 (3 H, t, J 7.0 Hz, O-CH_2-CH_2-CH_2-CH_2-CH_2-CH_2-$\underline{CH_3}$)

δ_C/ppm (100 MHz, DMSO-d_6): 166.99, 163.96, 160.42, 133.57, 113.45, 105.67, 100.07, 68.72, 55.91, 31.72, 29.03, 28.83, 25.81, 22.49, 14.42

4-Formyl-3-methoxyphenyl 2-alkyloxy-4-methylbenzoates (14.1)

To a pre-dried flask flushed with argon, **Compound 2** (1 eq), 4-hydroxy-2-methoxybenzaldehyde (0.91 eq) and *N,N′*-dicyclohexylcarbodiimide (1.2 eq) were added. The solids were solubilised with dichloromethane (100 mL) while being stirred for 2 min before 4-dimethylaminopyridine (0.091 eq) was added to the flask and the reaction was allowed to proceed overnight. The quantities of the reagents used in each reaction are listed in **Table SI22**. The extent of the reaction was monitored by TLC using an appropriate solvent system (RF values quoted in the product data). The precipitate which formed was removed by vacuum filtration and the filtrate collected. The collected solvent was evaporated under vacuum to leave a solid which was recrystallised from hot ethanol (200 mL).

Table SI22. Quantities of reagents used in the syntheses of the 4-formyl-3-methoxyphenyl 2-alkoxy-4-methylbenzoates

m	(**2**)	4-Hydroxy-2-methoxybenzaldehyde	4-Dimethylaminopyridine	*N,N′*-Dicyclohexylcarbodiimide
2	3.23 g, 0.0179 mol	2.48 g, 0.0163 mol	0.199 g, 1.63×10^{-3} mol	4.37 g, 0.0212 mol
3	3.86 g, 0.0199 mol	2.75 g, 0.0181 mol	0.221 g, 1.81×10^{-3} mol	4.84 g, 0.0235 mol
4	4.05 g, 0.0195 mol	2.69 g, 0.0177 mol	0.216 g, 1.77×10^{-3} mol	4.74 g, 0.0230 mol
5	3.51 g, 0.0158 mol	2.19 g, 0.0144 mol	0.175 g, 1.44×10^{-3} mol	3.85 g, 0.0187 mol

6	4.32 g, 0.0183 mol	2.52 g, 0.0166 mol	0.202 g, 1.66×10^{-3} mol	4.45 g, 0.0216 mol
7	4.56 g, 0.0182 mol	2.51 g, 0.0165 mol	0.202 g, 1.65×10^{-3} mol	4.43 g, 0.0215 mol

14.1.1 4-Formyl-3-methoxyphenyl 2-ethoxy-4-methylbenzoate

Yield: 2.47 g, 48 %. RF: 0.15 (100 % dichloromethane). M.P = 94 °C

v_{max}/cm^{-1}: 2975 (C-H), 2801 (CHO), 1741 (C=O), 1683 (CHO), 1601 (Ar C=C), 1572 (Ar C=C)

δ_H/ppm (400 MHz, DMSO-d_6): 10.31 (s, 1H, CHO), 7.84 (d, *J* = 7.9 Hz, 1H, Ar-H), 7.78 (d, *J* = 8.4 Hz, 1H, Ar-H), 7.15 (d, *J* = 1.9 Hz, 1H, Ar-H), 7.05 (s, 1H, Ar-H), 6.96 (dd, *J* = 8.4, 1.9 Hz, 1H, Ar-H), 6.91 (d, *J* = 7.8 Hz, 1H, Ar-H), 4.14 (q, *J* = 6.9 Hz, 2H, O$\underline{CH_2}$$CH_3$), 3.93 (s, 3H, OCH_3), 2.39 (s, 3H, Ar-CH_3), 1.35 (t, *J* = 6.9 Hz, 3H, $OCH_2$$\underline{CH_3}$)

δ_C/ppm (100 MHz, $CDCl_3$): 188.88, 163.65, 162.96, 159.92, 157.51, 146.21, 132.53, 129.96, 122.56, 121.23, 115.58, 114.60, 114.22, 106.00, 64.70, 56.00, 22.20, 14.89

14.1.2 4-Formyl-3-methoxyphenyl 4-methyl-2-propoxybenzoate

Yield: 2.18 g, 37 %. RF: 0.28 (100 % dichloromethane). M.P = 76 °C

v_{max}/cm^{-1}: 2966 (C-H), 2831 (CHO), 1740 (C=O), 1681 (CHO), 1599 (Ar C=C), 1573 (Ar C=C)

δ_H/ppm (400 MHz, DMSO-d_6): 10.31 (s, 1H, CHO), 7.83 (d, *J* = 7.9 Hz, 1H, Ar-H), 7.78 (d, *J* = 8.4 Hz, 1H, Ar-H), 7.13 (d, *J* = 2.0 Hz, 1H, Ar-H), 7.04 (s, 1H, Ar-H), 6.95 (dd, *J* = 8.4, 1.9 Hz, 1H, Ar-H), 6.90 (d, *J* = 7.9 Hz, 1H, Ar-H), 4.03 (t, *J* = 6.3 Hz, 2H, O$\underline{CH_2}$$CH_2CH_3$), 3.92 (s, 3H, OCH_3), 2.38 (s, 3H, Ar-CH_3), 1.75 (sext, 2H, *J* = 7.2 Hz, $OCH_2$$\underline{CH_2}$$CH_3$), 0.99 (t, *J* = 7.4 Hz, 3H, $OCH_2CH_2$$\underline{CH_3}$)

δ_C/ppm (100 MHz, $CDCl_3$): 188.85, 163.79, 162.97, 160.01, 157.53, 146.18, 132.60, 129.98, 122.57, 121.13, 115.54, 114.60, 114.05, 105.97, 70.49, 55.99, 22.73, 22.19, 10.74

14.1.3 4-Formyl-3-methoxyphenyl 2-butoxy-4-methylbenzoate

Yield: 1.95 g, 32 %. RF: 0.30 (100 % dichloromethane). M.P = 56 °C

v_{max}/cm^{-1}: 2959 (C-H), 2759 (CHO), 1740 (C=O), 1682 (CHO), 1599 (Ar C=C), 1571 (Ar C=C)

δ_H/ppm (400 MHz, DMSO-d_6): 10.31 (s, 1H, CHO), 7.83 (d, *J* = 7.9 Hz, 1H, Ar-H), 7.78 (d, *J* = 8.4 Hz, 1H, Ar-H), 7.13 (d, *J* = 1.9 Hz, 1H, Ar-H), 7.06 (s, 1H, Ar-H), 6.95 (dd, *J* = 8.5, 1.9 Hz, 1H, Ar-H), 6.91 (d, *J* = 7.9 Hz, 1H, Ar-H), 4.08 (t, *J* = 6.3 Hz, 2H, O$\underline{CH_2}$$(CH_2)_2CH_3$), 3.92 (s, 3H, OCH_3), 2.39 (s, 3H, Ar-CH_3), 1.71 (quin, *J* = 6.6 Hz, 2H, $OCH_2$$\underline{CH_2}$$CH_2CH_3$), 1.46 (sext, *J* = 7.3 Hz, 2H, $O(CH_2)_2$$\underline{CH_2}$$CH_3$), 0.90 (t, *J* = 7.4 Hz, 3H, $O(CH_2)_3$$\underline{CH_3}$)

δ_C/ppm (100 MHz, $CDCl_3$): 188.82, 163.74, 162.95, 159.99, 157.52, 146.16, 132.57, 129.93, 122.54, 121.09, 115.51, 114.57, 114.01, 105.94, 68.63, 55.96, 31.36, 22.17, 19.30, 13.91

14.1.4 4-Formyl-3-methoxyphenyl 4-methyl-2-pentoxybenzoate

Yield: 3.63 g, 71 %. RF: 0.33 (100 % dichloromethane). M.P = 55 °C

ν_{max}/cm^{-1}: 2958 (C-H), 2776 (CHO), 1747 (C=O), 1680 (CHO), 1598 (Ar C=C), 1571 (Ar C=C)

δ_H/ppm (400 MHz, $CDCl_3$): 10.41 (s, 1H, CHO), 7.90 (d, *J* = 7.9 Hz, 1H, Ar-H), 7.88 (d, *J* = 8.4 Hz, 1H, Ar-H), 6.90 – 6.87 (m, 2H, Ar-H), 6.85 – 6.79 (m, 2H, Ar-H), 4.06 (t, *J* = 6.5 Hz, 2H, $O\underline{CH_2}(CH_2)_3CH_3$), 3.92 (s, 3H, OCH_3), 2.41 (s, 3H, Ar-CH_3), 1.84 (quin, *J* = 6.7 Hz, 2H, $OCH_2\underline{CH_2}(CH_2)_2CH_3$), 1.52 – 1.30 (m, 4H, $OCH_2CH_2(\underline{CH_2})_2CH_3$), 0.88 (t, *J* = 7.3 Hz, 3H, $O(CH_2)_4\underline{CH_3}$)

δ_C/ppm (100 MHz, $CDCl_3$): 188.83, 163.82, 162.96, 159.97, 157.53, 146.16, 132.60, 129.95, 122.55, 121.12, 115.55, 114.57, 114.04, 105.95, 68.98, 55.97, 29.02, 28.25, 22.50, 22.18, 14.10

14.1.5 4-Formyl-3-methoxyphenyl 2-hexyloxy-4-methylbenzoate

Yield: 4.36 g, 71 %. RF: 0.35 (100 % dichloromethane). M.P = 66 °C

ν_{max}/cm^{-1}: 2953 (C-H), 2775 (CHO), 1741 (C=O), 1681 (CHO), 1597 (Ar C=C), 1571 (Ar C=C)

δ_H/ppm (400 MHz, $CDCl_3$): 10.41 (s, 1H, CHO), 7.90 (d, *J* = 7.9 Hz, 1H, Ar-H), 7.88 (d, *J* = 8.4 Hz, 1H, Ar-H), 6.90 – 6.86 (m, 2H, Ar-H), 6.85 – 6.81 (m, 2H, Ar-H), 4.06 (t, *J* = 6.5 Hz, 2H, $O\underline{CH_2}(CH_2)_4CH_3$), 3.92 (s, 3H, OCH_3), 2.41 (s, 3H, Ar-CH_3), 1.83 (quin, *J* = 6.7 Hz, 2H, $OCH_2\underline{CH_2}(CH_2)_3CH_3$), 1.49 (quin, *J* = 7.2 Hz, 2H, $O(CH_2)_2\underline{CH_2}(CH_2)_2CH_3$), 1.36 – 1.22 (m, 4H, $O(CH_2)_3(\underline{CH_2})_2CH_3$), 0.86 (t, *J* = 7.2 Hz, 3H, $O(CH_2)_5\underline{CH_3}$)

δ_C/ppm (100 MHz, $CDCl_3$): 188.82, 163.83, 162.96, 159.97, 157.53, 146.15, 132.60, 129.94, 122.56, 121.12, 115.58, 114.57, 114.05, 105.95, 69.00, 55.97, 31.62, 29.30, 25.78, 22.67, 22.18, 14.10

14.1.6 4-Formyl-3-methoxyphenyl 2-heptyloxy-4-methylbenzoate

Yield: 2.86 g, 45 %. RF: 0.38 (100 % dichloromethane). M.P = 43 °C

ν_{max}/cm^{-1}: 2956 (C-H), 2799 (CHO), 1703 (C=O), 1683 (CHO), 1600 (Ar C=C), 1585 (Ar C=C)

δ_H/ppm (400 MHz, $CDCl_3$): 10.41 (s, 1H, CHO), 7.90 (d, *J* = 7.9 Hz, 1H, Ar-H), 7.86 (d, *J* = 8.4 Hz, 1H, Ar-H), 6.90 – 6.86 (m, 2H, Ar-H), 6.86 – 6.81 (m, 2H, Ar-H), 4.06 (t, *J* = 6.5 Hz, 2H, $O\underline{CH_2}(CH_2)_5CH_3$), 3.92 (s, 3H, OCH_3), 2.41 (s, 3H, Ar-CH_3), 1.83 (quin, *J* = 6.7 Hz, 2H, $OCH_2\underline{CH_2}(CH_2)_4CH_3$), 1.48 (quin, *J* = 7.2 Hz, 2H, $O(CH_2)_2\underline{CH_2}(CH_2)_3CH_3$), 1.37 – 1.19 (m, 6H, $O(CH_2)_3(\underline{CH_2})_3CH_3$), 0.85 (t, *J* = 7.1 Hz, 3H, $O(CH_2)_6\underline{CH_3}$)

δ_C/ppm (100 MHz, $CDCl_3$): 188.81, 163.84, 162.96, 159.97, 157.54, 146.15, 132.60, 129.94, 122.55, 121.12, 115.57, 114.57, 114.05, 105.94, 69.00, 55.97, 31.86, 29.35, 29.11, 26.06, 22.68, 22.18, 14.17

4-Formyl-3-methoxyphenyl 2-alkyloxy-4-methoxybenzoates (14.2)

To a pre-dried flask flushed with argon, **Compound 13** (1 eq), 4-hydroxy-2-methoxybenzaldehyde (1.1 eq) and 4-dimethylaminopyridine (0.13 eq) were added. The solids were solubilised with dichloromethane (100 mL) and tetrahydrofuran (20 mL) while being stirred for 10 min before *N,N′*-dicyclohexylcarbodiimide (1.3 eq) was added to the flask and the reaction was allowed to proceed overnight. The quantities of the reagents used in each reaction are listed in **Table SI23**. The extent of the reaction was monitored by TLC using an appropriate solvent system (RF values quoted in the product data). The precipitate which formed was removed by vacuum filtration and the filtrate collected. The collected solvent was evaporated under vacuum to leave a solid which was recrystallised from hot ethanol (125 mL).

Table SI23. Quantities of reagents used in the syntheses of the 4-formyl-3-methoxyphenyl 4-alkyloxybenzoates

m	(**13**)	4-Hydroxy-2-methoxybenzaldehyde	4-Dimethylaminopyridine	*N,N'*-Dicyclohexylcarbodiimide
3	3.60 g, 0.0171 mol	2.86 g, 0.0188 mol	0.271 g, 2.22×10^{-3} mol	4.58 g, 0.0222 mol
4	3.60 g, 0.0161 mol	2.69 g, 0.0177 mol	0.255 g, 2.09×10^{-3} mol	4.31 g, 0.0209 mol
5	3.80 g, 0.0159 mol	2.66 g, 0.0175 mol	0.253 g, 2.07×10^{-3} mol	4.27 g, 0.0207 mol
6	3.50 g, 0.0139 mol	2.33 g, 0.0153 mol	0.221 g, 1.81×10^{-3} mol	3.73 g, 0.0181 mol
7	2.00 g, 7.51×10^{-3} mol	1.26 g, 8.26×10^{-3} mol	0.113 g, 1.87×10^{-3} mol	1.90 g, 7.51×10^{-3} mol

14.2.2 4-Formyl-3-methoxyphenyl 4-methoxy-2-propoxybenzoate

Brown solid. Yield: 3.73 g, 63.3 %. RF: 0.40 (100 % dichloromethane). M.P = 98 °C

ν_{max}/cm^{-1}: 2968 (-CH_3), 2860 (-CH_2), 1738 (COOR), 1679 (RC(=O)H), 1605 (Ar C=C), 1230 (C-O-C)

δ_H/ppm (400 MHz, $CDCl_3$): 10.41 (s, 1H, CHO), 8.03 (d, *J* = 8.8 Hz, 1H, Ar-H), 7.88 (m, 1H, Ar-H), 6.88 (m, 2H, Ar-H), 6.55 (dd, *J* = 8.8, 2.4 Hz, 1H, Ar-H), 6.51 (d, *J* = 3.4 Hz, 1H, Ar-H), 4.02 (t, *J* = 6.4 Hz, 2H, $O\underline{CH_2}CH_2CH_3$), 3.92 (s, 3H, OCH_3), 3.88 (s, 3H, OCH_3), 1.87 (m, 2H, $OCH_2\underline{CH_2}CH_3$), 1.07 (t, *J* = 7.4 Hz, 3H, $OCH_2CH_2\underline{CH_3}$)

δ_C/ppm (100 MHz, $CDCl_3$): 188.75, 165.22, 163.13, 162.84, 161.92, 157.50, 134.61, 129.85, 122.39, 114.54, 110.68, 105.91, 104.98, 99.70, 70.44, 55.87, 55.60, 22.52, 10.63

14.2.2 4-Formyl-3-methoxyphenyl 2-butoxy-4-methoxybenzoate

Off-white solid. Yield: 3.27 g, 56.7 %. RF: 0.37 (100 % dichloromethane). M.P = 105 °C

ν_{max}/cm^{-1}: 2970 (-CH_3), 2900 (-CH_2), 1745 (COOR), 1677 (RC(=O)H), 1598 (Ar C=C), 1240 (C-O-C)

δ_H/ppm (400 MHz, $CDCl_3$): 10.41 (s, 1H, CHO), 8.03 (d, *J* = 8.8 Hz, 1H, Ar-H), 7.88 (m, 1H, Ar-H), 6.88 (m, 2H, Ar-H), 6.55 (dd, *J* = 8.8, 2.3 Hz, 1H, Ar-H), 6.52 (d, *J* = 2.3 Hz, 1H, Ar-H), 4.06 (t, *J* = 6.4 Hz, 2H, $O\underline{CH_2}(CH_2)_2CH_3$), 3.92 (s, 3H, OCH_3), 3.88 (s, 3H, OCH_3), 1.83 (m, 2H, $OCH_2\underline{CH_2}CH_2CH_3$), 1.54 (m, 2H, $O(CH_2)_2\underline{CH_2}CH_3$), 0.95 (t, *J* = 7.4 Hz, 3H, $O(CH_2)_3\underline{CH_3}$)

δ_C/ppm (100 MHz, $CDCl_3$): 188.75, 165.21, 163.10, 162.84, 161.92, 157.50, 134.61, 129.84, 122.39, 114.53, 110.68, 105.90, 104.95, 99.70, 68.63, 55.87, 55.60, 31.15, 19.21, 13.81

14.2.3 4-Formyl-3-methoxyphenyl 4-methoxy-2-pentoxybenzoate

Off-white solid. Yield: 3.53 g, 59.6 %. RF: 0.40 (100 % dichloromethane). M.P = 102 °C

ν_{max}/cm^{-1}: 2958 (-CH_3), 2858 (-CH_2), 1745 (COOR), 1680 (RC(=O)H), 1599 (Ar C=C), 1236 (C-O-C)

δ_H/ppm (400 MHz, $CDCl_3$): 10.41 (s, 1H, CHO), 8.03 (d, *J* = 8.8 Hz, 1H, Ar-H), 7.89 (m, 1H, Ar-H), 6.88 (m, 2H, Ar-H), 6.55 (dd, *J* = 8.8, 2.3 Hz, 1H, Ar-H), 6.51 (d, *J* = 2.3 Hz, 1H, Ar-H), 4.05 (t, *J* = 6.5 Hz, 2H, $O\underline{CH_2}(CH_2)_3CH_3$), 3.93 (s, 3H, OCH_3), 3.88 (s, 3H, OCH_3), 1.85 (m, 2H, $OCH_2\underline{CH_2}(CH_2)_2CH_3$), 1.47 (m, 2H, $OCH_2CH_2\underline{CH_2}CH_2CH_3$), 1.35 (m, 2H, $OCH_2CH_2CH_2\underline{CH_2}CH_3$), 0.88 (t, *J* = 7.3 Hz, 3H, $O(CH_2)_4\underline{CH_3}$)

δ_C/ppm (100 MHz, $CDCl_3$): 188.75, 165.21, 163.18, 162.84, 161.89, 157.50, 134.63, 129.84, 122.39, 114.53, 110.71, 105.90, 104.96, 99.71, 68.97, 55.87, 55.60, 28.81, 28.14, 22.39, 13.99

14.2.4 4-Formyl-3-methoxyphenyl 2-hexyloxy-4-methoxybenzoate

Off-white solid. Yield: 2.83 g, 52.7 %. RF: 0.40 (100 % dichloromethane). M.P = 96 °C

ν_{max}/cm^{-1}: 2951 ($-CH_3$), 2853 ($-CH_2$), 1737 (COOR), 1676 (RC(=O)H), 1600 (Ar C=C), 1235 (C-O-C)

δ_H/ppm (400 MHz, $CDCl_3$): 10.41 (s, 1H, CHO), 8.03 (d, *J* = 8.8 Hz, 1H, Ar-H), 7.89 (m, 1H, Ar-H), 6.87 (m, 2H, Ar-H), 6.55 (dd, *J* = 8.8, 2.4 Hz, 1H, Ar-H), 6.51 (d, *J* = 2.4 Hz, 1H, Ar-H), 4.05 (t, *J* = 6.5 Hz, 2H, $O\underline{CH_2}(CH_2)_4CH_3$), 3.92 (s, 3H, OCH_3), 3.88 (s, 3H, OCH_3), 1.84 (m, 2H, $OCH_2\underline{CH_2}(CH_2)_3CH_3$), 1.49 (m, 2H, $O(CH_2)_2\underline{CH_2}(CH_2)_2CH_3$), 1.30 (m, 4H, $O(CH_2)_3(\underline{CH_2})_2CH_3$), 0.86 (t, *J* = 7.1 Hz, 3H, $O(CH_2)_5\underline{CH_3}$)

δ_C/ppm (100 MHz, $CDCl_3$): 188.75, 165.20, 163.19, 162.84, 161.89, 157.50, 134.63, 129.84, 122.39, 114.53, 110.73, 105.90, 104.96, 99.72, 68.98, 55.86, 55.60, 31.51, 29.08, 25.67, 22.56, 14.00

14.2.5 4-Formyl-3-methoxyphenyl 2-heptyloxy-4-methoxybenzoate

Orange solid. Yield: 1.84 g, 61.1 %. RF: 0.35(100 % dichloromethane). M.P = 69 °C

ν_{max}/cm^{-1}: 2958 ($-CH_3$), 2853 ($-CH_2$), 1710 (COOR), 1671 (RC(=O)H), 1600 (Ar C=C), 1247 (C-O-C)

δ_H/ppm (400 MHz, $CDCl_3$): 10.40 (s, 1H, CHO), 8.03 (d, *J* = 8.7 Hz, 1H, Ar-H), 7.88 (m, 1H, Ar-H), 6.87 (m, 2H, Ar-H), 6.54 (dd, *J* = 8.7, 2.3 Hz, 1H, Ar-H), 6.51 (d, *J* = 3.4 Hz, 1H, Ar-H), 4.04 (t, *J* = 6.5 Hz, 2H, $O\underline{CH_2}CH_2CH_3$), 3.90 (s, 3H, OCH_3), 3.87 (s, 3H, OCH_3), 1.83 (m, 2H, 2H, $OCH_2\underline{CH_2}(CH_2)_4CH_3$), 1.48 (m, 2H, $O(CH_2)_2\underline{CH_2}(CH_2)_3CH_3$), 1.24 (m, 6H, $O(CH_2)_3(\underline{CH_2})_3CH_3$), 0.85 (t, *J* = 7.1 Hz, 3H, $O(CH_2)_6\underline{CH_3}$)

δ_C/ppm (100 MHz, $CDCl_3$): 188.86, 165.22, 163.21, 162.85, 161.88, 157.52, 134.62, 129.82, 122.36, 114.53, 110.68, 105.90, 104.98, 99.71, 68.97, 55.86, 55.59, 31.74, 29.12, 29.00, 25.95, 22.57, 14.00

4-((2-Alkoxy-4-methylbenzoyl)oxy) 2-methoxybenzoic acids (15.1)

To a pre-dried flask flushed with argon, **Compound 14.1** (1 eq) and resorcinol (1.5 eq) were solubilised in DMSO (30 mL). Sodium chlorite (4 eq) and sodium hydrogen phosphate monohydrate (3.5 eq) were solubilised in water (20 mL) before being slowly poured into the reaction flask which was cooled by an ice bath. The resultant mixture was stirred at room temperature overnight. The quantities of the reagents used in each reaction are listed in **Table SI24**. The extent of the reaction was monitored by TLC using an appropriate solvent system (RF values quoted in the product data). When the reaction was completed, the mixture was diluted with water (150 mL), the pH was adjusted to pH 4 by the addition of 1 M hydrochloric acid. The precipitated solid was filtered, washed with water and then recrystallised using hot ethanol (40 mL) to give a white solid.

Table SI24. Quantities of reagents used in the syntheses of the ((2-alkoxy-4-methylbenzoyl)oxy)-2-methoxybenzoic acids

m	(**14.1**)	Sodium Chlorite	Sodium Hydrogen Phosphate Monohydrate	Resorcinol
2	2.42 g, 7.71×10^{-3} mol	2.77 g, 0.0308 mol	3.78 g, 0.0270 mol	1.28 g, 0.0116 mol
3	2.12 g, 6.46×10^{-3} mol	2.32 g, 0.0258 mol	3.16 g, 0.0226 mol	1.07 g, 9.69×10^{-3} mol
4	1.89 g, 5.53×10^{-3} mol	1.99 g, 0.0221 mol	2.72 g, 0.0194 mol	0.91 g, 8.30×10^{-3} mol
5	3.58 g, 0.0101 mol	3.64 g, 0.0404 mol	4.96 g, 0.0354 mol	1.67 g, 0.0152 mol
6	4.30 g, 0.0116 mol	4.18 g, 0.0464 mol	5.68 g, 0.0406 mol	1.91 g, 0.0174 mol
7	2.80 g, 7.29×10^{-3} mol	2.63 g, 0.0292 mol	3.57 g, 0.0255 mol	1.20 g, 0.0109 mol

15.1.1 4-((2-Ethoxy-4-methylbenzoyl)oxy) 2-methoxybenzoic acid

Yield: 1.00 g, 39 %. RF: 0.08 (20 % ethyl acetate:80 % 40:60 petroleum ether). M.P = 124 °C

ν_{max}/cm^{-1}: 2987 (C-H), 2882 (broad OH), 1740 (C=O), 1600 (Ar C=C), 1583 (Ar C=C)

δ_H/ppm (400 MHz, DMSO-d$_6$): 12.45 (s, 1H, COOH), 7.82 (d, *J* = 7.9 Hz, 1H, Ar-H), 7.74 (d, *J* = 8.4 Hz, 1H, Ar-H), 7.04 (s, 1H, Ar-H), 7.01 (d, *J* = 1.9 Hz, 1H, Ar-H), 6.90 (d, *J* = 7.9 Hz, 1H, Ar-H), 6.86 (dd, *J* = 8.4, 2.0 Hz, 1H, Ar-H), 4.13 (q, *J* = 6.9 Hz, 2H, O$\underline{CH_2}$CH$_3$), 3.82 (s, 3H, OCH$_3$), 2.38 (s, 3H, Ar-CH$_3$), 1.35 (t, *J* = 6.9 Hz, 3H, OCH$_2\underline{CH_3}$)

δ_C/ppm (100 MHz, DMSO-d$_6$): 166.65, 163.33, 159.49, 158.76, 154.47, 145.58, 131.92, 131.71, 120.93, 118.53, 115.51, 114.33, 113.62, 106.68, 64.10, 56.08, 21.52, 14.59

15.1.2 4-((4-Methyl-2-propoxybenzoyl)oxy) 2-methoxybenzoic acid

Yield: 1.06 g, 48 %. RF: 0.08 (20 % ethyl acetate:80 % 40:60 petroleum ether). M.P = 101 °C

ν_{max}/cm^{-1}: 2968 (C-H), 2880 (broad OH), 1708 (C=O), 1601 (Ar C=C), 1569 (Ar C=C)

δ_H/ppm (400 MHz, DMSO-d$_6$): 12.59 (1 H, s, OH), 7.97 (2 H, d, J 8.7 Hz, Ar-H), 7.73 (1 H, d, J 8.4 Hz, Ar-H), 7.01 (1 H, d, J 2.0 Hz, Ar-H), 6.85 (1 H, dd, J 8.4 Hz, 2.0 Hz, Ar-H), 6.72 (1 H, d, J 2.2 Hz, Ar-H), 6.68 (1 H, dd, J 8.7 Hz, 2.2 Hz, Ar-H), 4.15 (2 H, quart, 7.0 Hz, O-$\underline{CH_2}$-CH$_3$), 3.91 (3 H, s, O-$\underline{CH_3}$), 1.36 (3 H, t, 7.0 Hz, O-CH$_2$-$\underline{CH_3}$)

δ_C/ppm (100 MHz, DMSO-d$_6$): 166.62, 163.45, 159.51, 158.92, 154.51, 145.64, 131.95, 131.79, 120.87, 118.48, 115.37, 114.16, 113.61, 106.65, 69.73, 56.07, 22.12, 21.51, 10.46

15.1.3 4-((2-Butoxy-4-methylbenzoyl)oxy) 2-methoxybenzoic acid

Yield: 1.01 g, 51 %. RF: 0.08 (20 % ethyl acetate:80 % 40:60 petroleum ether). M.P = 108 °C

ν_{max}/cm^{-1}: 2961 (C-H), 2868 (broad OH), 1711 (C=O), 1602 (Ar C=C), 1567 (Ar C=C)

δ_H/ppm (400 MHz, DMSO-d_6): 12.63 (s, 1H, COOH), 7.81 (d, *J* = 7.9 Hz, 1H, Ar-H), 7.74 (d, *J* = 8.4 Hz, 1H, Ar-H), 7.05 (s, 1H, Ar-H), 7.00 (d, *J* = 2.1 Hz, 1H, Ar-H), 6.90 (d, *J* = 7.9 Hz, 1H, Ar-H), 6.85 (dd, *J* = 8.4, 2.1 Hz, 1H, Ar-H), 4.07 (t, *J* = 6.3 Hz, 2H, $O\underline{CH_2}(CH_2)_2CH_3$), 3.82 (s, 3H, OCH_3), 2.38 (s, 3H, Ar-CH_3), 1.71 (quin, *J* = 6.7 Hz, 2H, $OCH_2\underline{CH_2}CH_2CH_3$), 1.47 (sext, *J* = 7.4 Hz, 2H, $O(CH_2)_2\underline{CH_2}CH_3$), 0.90 (t, *J* = 7.4 Hz, 3H, $O(CH_2)_3\underline{CH_3}$)

δ_C/ppm (100 MHz, DMSO-d_6): 166.61, 163.44, 159.51, 158.92, 154.51, 145.63, 131.95, 131.77, 120.85, 118.47, 115.38, 114.15, 113.60, 106.62, 67.91, 56.06, 30.77, 21.51, 18.69, 13.63

15.1.4 4-((4-Methyl-2-pentoxybenzoyl)oxy) 2-methoxybenzoic acid

Yield: 1.64 g, 44 %. RF: 0.08 (20 % ethyl acetate:80 % 40:60 petroleum ether). M.P = 106 °C

v_{max}/cm^{-1}: 2943 (C-H), 2869 (broad OH), 1714 (C=O), 1605 (Ar C=C), 1568 (Ar C=C)

δ_H/ppm (400 MHz, DMSO-d_6): 12.65 (s, 1H, COOH), 7.81 (d, *J* = 7.9 Hz, 1H, Ar-H), 7.75 (d, *J* = 8.4 Hz, 1H, Ar-H), 7.03 (s, 1H, Ar-H), 7.00 (d, *J* = 2.1 Hz, 1H, Ar-H), 6.89 (d, *J* = 7.5 Hz, 1H, Ar-H), 6.85 (dd, *J* = 8.4, 2.1 Hz, 1H, Ar-H), 4.05 (t, *J* = 6.3 Hz, 2H, $O\underline{CH_2}(CH_2)_3CH_3$), 3.82 (s, 3H, OCH_3), 2.37 (s, 3H, Ar-CH_3), 1.72 (quin, *J* = 6.7 Hz, 2H, $OCH_2\underline{CH_2}(CH_2)_2CH_3$), 1.46 – 1.24 (m, 4H, $OCH_2CH_2(\underline{CH_2})_2CH_3$), 0.82 (t, *J* = 7.3 Hz, 3H, $O(CH_2)_4\underline{CH_3}$)

δ_C/ppm (100 MHz, DMSO-d_6): 166.60, 163.52, 159.51, 158.88, 154.51, 145.62, 131.94, 131.77, 120.85, 118.44, 115.40, 114.15, 113.57, 106.62, 68.21, 56.05, 28.39, 27.66, 21.83, 21.50, 13.88

15.1.5 4-((2-Hexyloxy-4-methylbenzoyl)oxy) 2-methoxybenzoic acid

Yield: 3.26 g, 73 %. RF: 0.08 (20 % ethyl acetate:80 % 40:60 petroleum ether). M.P = 133 °C

v_{max}/cm^{-1}: 2940 (C-H), 2858 (broad OH), 1728 (C=O), 1601 (Ar C=C), 1572 (Ar C=C)

δ_H/ppm (400 MHz, DMSO-d_6): 12.64 (s, 1H, COOH), 7.81 (d, *J* = 7.9 Hz, 1H, Ar-H), 7.76 (d, *J* = 8.4 Hz, 1H, Ar-H), 7.04 (s, 1H, Ar-H), 7.01 (d, *J* = 2.1 Hz, 1H, Ar-H), 6.89 (d, *J* = 7.9 Hz, 1H, Ar-H), 6.85 (dd, *J* = 8.4, 2.1 Hz, 1H, Ar-H), 4.06 (t, *J* = 6.2 Hz, 2H, $O\underline{CH_2}(CH_2)_4CH_3$), 3.82 (s, 3H, OCH_3), 2.38 (s, 3H, Ar-CH_3), 1.72 (quin, *J* = 6.7 Hz, 2H, $OCH_2\underline{CH_2}(CH_2)_3CH_3$), 1.44 (quin, *J* = 7.2 Hz, 2H, $O(CH_2)_2\underline{CH_2}(CH_2)_2CH_3$), 1.30 – 1.16 (m, 4H, $O(CH_2)_3(\underline{CH_2})_2CH_3$), 0.81 (t, *J* = 7.1 Hz, 3H, $O(CH_2)_5\underline{CH_3}$)

δ_C/ppm (100 MHz, DMSO-d_6): 166.60, 163.53, 159.50, 158.87, 154.49, 145.60, 131.92, 131.76, 120.84, 118.47, 115.41, 114.14, 113.54, 106.59, 68.21, 56.03, 30.94, 28.67, 25.15, 22.07, 21.50, 13.82

15.1.6 4-((2-Heptyloxy-4-methylbenzoyl)oxy) 2-methoxybenzoic acid

Yield: 1.35 g, 46 %. RF: 0.08 (20 % ethyl acetate:80 % 40:60 petroleum ether). M.P = 100 °C

v_{max}/cm^{-1}: 2936 (C-H), 2856 (broad OH), 1725 (C=O), 1603 (Ar C=C), 1571 (Ar C=C)

δ_H/ppm (400 MHz, DMSO-d_6): 12.63 (s, 1H, COOH), 7.80 (d, *J* = 8.0 Hz, 1H, Ar-H), 7.75 (d, *J* = 8.4 Hz, 1H, Ar-H), 7.03 (s, 1H, Ar-H), 7.00 (d, *J* = 2.1 Hz, 1H, Ar-H), 6.88 (d, *J* = 7.9 Hz, 1H, Ar-H), 6.84 (dd, *J* = 8.4, 2.1 Hz, 1H, Ar-H), 4.05 (t, *J* = 6.2 Hz, 2H, $O\underline{CH_2}(CH_2)_5CH_3$), 3.82 (s, 3H, OCH_3), 2.37 (s, 3H, Ar-CH_3), 1.71 (quin, *J* = 6.5 Hz, 2H, $OCH_2\underline{CH_2}(CH_2)_4CH_3$), 1.42 (quin, *J* = 7.2 Hz, 2H, $O(CH_2)_2\underline{CH_2}(CH_2)_3CH_3$), 1.30 – 1.11 (m, 6H, $O(CH_2)_3(\underline{CH_2})_3CH_3$), 0.80 (t, *J* = 7.2 Hz, 3H, $O(CH_2)_6\underline{CH_3}$)

δ_C/ppm (100 MHz, DMSO-d$_6$): 166.56, 163.57, 159.53, 158.86, 154.52, 145.60, 131.94, 131.77, 120.84, 118.40, 115.41, 114.13, 113.52, 106.58, 68.20, 56.03, 31.26, 28.73, 28.43, 25.45, 22.01, 21.49, 13.89

4-((2-Alkyloxy-4-methoxybenzoyl)oxy) 2-methoxybenzoic acids (15.2)

To a pre-dried flask flushed with argon, **Compound 14.2** (1 eq) and resorcinol (1.5 eq) were solubilised in DMSO (80 mL). Sodium chlorite (4 eq) and sodium hydrogen phosphate monohydrate (3.5 eq) were solubilised in water (40 mL) before being slowly poured into the reaction flask which was cooled by an ice bath. The resultant mixture was stirred at room temperature overnight. The quantities of the reagents used in each reaction are listed in **Table SI25**. The extent of the reaction was monitored by TLC using an appropriate solvent system (RF values quoted in the product data). The reaction mixture was diluted with water (250 mL) and the pH of the mixture was adjusted to 1 using 32% hydrochloric acid (25 mL). A solid precipitated after acidification which was collected by vacuum filtration and recrystallised from hot ethanol (100 mL).

Table SI25. Quantities of reagents used in the syntheses of the ((2-alkoxy-4-methoxybenzoyl)oxy) 2-methoxybenzoic acids

m	(**14.2**)	Sodium Chlorite	Sodium Hydrogen Phosphate Monohydrate	Resorcinol
3	0.940 g, 2.73×10^{-3} mol	0.986 g, 0.0109 mol	1.32 g, 9.56×10^{-3} mol	0.451 g, 4.10×10^{-3} mol
4	0.970 g, 2.71×10^{-3} mol	0.976 g, 0.0108 mol	1.31 g, 9.49×10^{-3} mol	0.448 g, 4.07×10^{-3} mol
5	1.85 g, 4.97×10^{-3} mol	1.80 g, 0.0199 mol	2.40 g, 0.0174 mol	0.821 g, 7.46×10^{-3} mol
6	1.20 g, 3.11×10^{-3} mol	1.12 g, 0.0124 mol	1.50 g, 0.0109 mol	0.514 g, 4.67×10^{-3} mol
7	1.75 g, 4.37×10^{-3} mol	1.58 g, 0.0175 mol	2.11 g, 0.0153 mol	0.722 g, 6.56×10^{-3} mol

15.2.1 4-((4-Methoxy-2-propoxybenzoyl)oxy) 2-methoxybenzoic acid

White solid. Yield: 0.495 g, 50.3 %. RF: 0.15 (100 % dichloromethane). M.P = 148 °C

v_{max}/cm^{-1}: 2980 (-CH$_3$), 2900 (-CH$_2$), 1741 (COOR), 1662 (COOH dimer), 1605 (Ar C=C), 1232 (C-O-C)

δ_H/ppm (400 MHz, DMSO-d$_6$): 12.59 (s, 1H, OH), 7.97 (d, *J* = 8.7 Hz, 2H, Ar-H), 7.73 (d, *J* = 8.4 Hz, 1H, Ar-H), 6.99 (d, *J* = 2.1 Hz, 1H, Ar-H), 6.84 (dd, *J* = 8.4, 2.1 Hz, 1H, Ar-H), 6.69 (d, *J* = 2.3 Hz, 1H, Ar-H), 6.66 (dd, *J* = 8.7 Hz, 2.3 Hz, 1H, Ar-H), 4.04 (t, *J* = 6.2 Hz, 2H, O$\underline{CH_2}$CH$_2$CH$_3$), 3.86 (s, 3H, O$\underline{CH_3}$), 3.81 (s,

3H, $O\underline{CH_3}$), 1.75 (3 H, t, 7.0 Hz, $O\text{-}CH_2\text{-}\underline{CH_3}$), 1.75 (m, 2H, $OCH_2\underline{CH_2}CH_3$), 1.00 (t, *J* = 7.4 Hz, 4H, $O(CH_2)_2\underline{CH_3}$)

δ_C/ppm (100 MHz, DMSO-d_6): 167.06, 165.25, 163.24, 161.60, 159.90, 155.05, 134.42, 132.32, 118.78, 114.13, 110.61, 107.18, 106.21, 100.03, 70.30, 56.53, 56.16, 22.48, 10.96

15.2.2 4-((2-Butoxy-4-methoxybenzoyl)oxy) 2-methoxybenzoic acid

Off-white solid. Yield: 1.00 g, 98.6 %. RF: 0.08 (100 % dichloromethane). M.P = 165 °C

v_{max}/cm^{-1}: 2955 (-CH_3), 2869 (-CH_2), 1736 (COOR), 1664 (COOH dimer), 1605 (Ar C=C), 1234 (C-O-C)

δ_H/ppm (400 MHz, DMSO-d_6): 12.62 (s, 1H, OH), 7.93 (d, *J* = 8.7 Hz, 2H, Ar-H), 7.73 (d, *J* = 8.4 Hz, 1H, Ar-H), 6.99 (d, *J* = 2.1 Hz, 1H, Ar-H), 6.84 (dd, *J* = 8.4, 2.1 Hz, 1H, Ar-H), 6.70 (d, *J* = 2.4 Hz, 1H, Ar-H), 6.66 (dd, *J* = 8.7 Hz, 2.4 Hz, 1H, Ar-H), 4.08 (t, *J* = 6.2 Hz, 2H, $O\underline{CH_2}(CH_2)_2CH_3$), 3.86 (s, 3H, $O\underline{CH_3}$), 3.81 (s, 3H, $O\underline{CH_3}$), 1.70 (m, 2H, $OCH_2\underline{CH_2}CH_2CH_3$), 1.47 (m, 2H, $O(CH_2)_2\underline{CH_2}CH_3$), 0.90 (t, *J* = 7.4 Hz, 3H, $O(CH_2)_3\underline{CH_3}$)

δ_C/ppm (100 MHz, DMSO-d_6): 166.61, 163.44, 159.51, 158.92, 154.51, 145.63, 131.95, 131.77, 120.85, 118.47, 115.38, 114.15, 113.60, 106.62, 67.91, 56.06, 30.77, 21.51, 18.69, 13.63

15.2.3 4-((4-Methoxy-2-pentoxybenzoyl)oxy) 2-methoxybenzoic acid

White solid. Yield: 1.51 g, 78.2 %. RF: 0.09 (100 % dichloromethane). M.P = 155 °C

v_{max}/cm^{-1}: 2951 (-CH_3), 2860 (-CH_2), 1719 (COOR), 1664 (COOH dimer), 1598 (Ar C=C), 1235 (C-O-C)

δ_H/ppm (400 MHz, DMSO-d_6): 12.63 (s, 1H, OH), 7.92 (d, *J* = 8.7 Hz, 2H, Ar-H), 7.74 (d, *J* = 8.4 Hz, 1H, Ar-H), 6.99 (d, *J* = 2.1 Hz, 1H, Ar-H), 6.84 (dd, *J* = 8.4, 2.1 Hz, 1H, Ar-H), 6.69 (d, *J* = 2.3 Hz, 1H, Ar-H), 6.65 (dd, *J* = 8.7 Hz, 2.3 Hz, 1H, Ar-H), 4.07 (t, *J* = 6.2 Hz, 2H, $O\underline{CH_2}(CH_2)_3CH_3$), 3.86 (s, 3H, $O\underline{CH_3}$), 3.81 (s, 3H, $O\underline{CH_3}$), 1.72 (m, 2H, $OCH_2\underline{CH_2}(CH_2)_2CH_3$), 1.42 (m, 2H, $OCH_2CH_2\underline{CH_2}CH_2CH_3$), 1.31 (m, 2H, $OCH_2CH_2CH_2\underline{CH_2}CH_3$), 0.83 (t, *J* = 7.3 Hz, 3H, $O(CH_2)_4\underline{CH_3}$)

δ_C/ppm (100 MHz, DMSO-d_6): 167.05, 165.25, 163.32, 161.57, 159.92, 155.07, 134.42, 132.33, 118.74, 114.11, 110.63, 107.15, 106.20, 100.03, 68.79, 56.51, 56.15, 28.74, 28.13, 22.27, 14.35

15.2.4 4-((2-Hexyloxy-4-methoxybenzoyl)oxy) 2-methoxybenzoic acid

White solid. Yield: 0.855 g, 68.3 %. RF: 0.15 (100 % dichloromethane). M.P = 147 °C

v_{max}/cm^{-1}: 2922 (-CH_3), 2860 (-CH_2), 1736 (COOR), 1665 (COOH dimer), 1603 (Ar C=C), 1234 (C-O-C)

δ_H/ppm (400 MHz, DMSO-d_6): 12.63 (s, 1H, OH), 7.92 (d, *J* = 8.7 Hz, 2H, Ar-H), 7.73 (d, *J* = 8.4 Hz, 1H, Ar-H), 6.98 (d, *J* = 2.1 Hz, 1H, Ar-H), 6.83 (dd, *J* = 8.4, 2.1 Hz, 1H, Ar-H), 6.69 (d, *J* = 2.4 Hz, 1H, Ar-H), 6.65 (dd, *J* = 8.7 Hz, 2.4 Hz, 1H, Ar-H), 4.07 (t, *J* = 6.2 Hz, 2H, $O\underline{CH_2}(CH_2)_4CH_3$), 3.86 (s, 3H, $O\underline{CH_3}$), 3.81 (s, 3H, $O\underline{CH_3}$), 1.71 (m, 2H, $OCH_2\underline{CH_2}(CH_2)_3CH_3$), 1.44 (m, 2H, $O(CH_2)_2\underline{CH_2}(CH_2)_2CH_3$), 1.25 (m, 4H, $O(CH_2)_3\underline{(CH_2)}_2CH_3$), 0.80 (t, *J* = 7.2 Hz, 3H, $O(CH_2)_5\underline{CH_3}$)

δ_C/ppm (100 MHz, DMSO-d_6): 167.06, 165.24, 163.35, 161.56, 159.92, 155.06, 134.41, 132.32, 118.74, 114.10, 110.65, 107.14, 106.21, 100.03, 68.81, 56.50, 56.16, 31.38, 29.02, 25.60, 22.51, 14.29

15.2.5 4-((2-Heptyloxy-4-methoxybenzoyl)oxy) 2-methoxybenzoic acid

Brown solid. Yield: 0.930 g, 51.1 %. RF: 0.21 (100 % dichloromethane). M.P = 119 °C

ν_{max}/cm^{-1}: 2924 (-CH_3), 2850 (-CH_2), 1737 (COOR), 1662 (COOH dimer), 1597 (Ar C=C), 1232 (C-O-C)

δ_H/ppm (400 MHz, DMSO-d_6): 12.60 (s, 1H, OH), 7.91 (d, *J* = 8.7 Hz, 2H, Ar-H), 7.73 (d, *J* = 8.4 Hz, 1H, Ar-H), 6.98 (d, *J* = 2.1 Hz, 1H, Ar-H), 6.83 (dd, *J* = 8.4, 2.1 Hz, 1H, Ar-H), 6.69 (d, *J* = 2.4 Hz, 1H, Ar-H), 6.65 (dd, *J* = 8.7 Hz, 2.4 Hz, 1H, Ar-H), 4.07 (t, *J* = 6.2 Hz, 2H, O$\underline{CH_2}$(CH_2)$_2$$CH_3$), 3.86 (s, 3H, O$\underline{CH_3}$), 3.81 (s, 3H, O$\underline{CH_3}$), 1.71 (m, 2H, O$CH_2$$\underline{CH_2}$($CH_2$)$_4$$CH_3$), 1.43 (m, 2H, O($CH_2$)$_2$$\underline{CH_2}$($CH_2$)$_3$$CH_3$), 1.20 (m, 6H, O($CH_2$)$_3$($\underline{CH_2}$)$_3$$CH_3$), 0.79 (t, *J* = 7.0 Hz, 3H, O(CH_2)$_6$$\underline{CH_3}$)

δ_C/ppm (100 MHz, DMSO-d_6): 167.04, 165.25, 163.41, 161.54, 159.94, 155.08, 134.42, 132.34, 118.71, 114.08, 110.66, 107.12, 106.22, 100.03, 68.80, 56.50, 56.16, 31.70, 29.08, 28.86, 25.91, 22.46, 14.37

16.1 3-Methoxy-4-((4-nitrophenoxy)carbonyl)phenyl 2-alkoxy-4-methylbenzoates (E.m)

To a pre-dried flask flushed with argon **Compound 15.1** (1 eq), 4-nitrophenol (0.91 eq) and *N,N'*-dicyclohexylcarbodiimide (1.18 eq) were added. The solids were solubilised with dichloromethane (50 mL) and stirred for 2 min before 4-dimethylaminopyridine (0.091 eq) was added to the flask. The quantities of the reagents used in each reaction are listed in **Table SI26**. The temperature of the reaction mixture was increased to room temperature and the reaction was allowed to proceed overnight. A white precipitate which formed was removed by vacuum filtration and the filtrate collected. The solvent was removed under vacuum and the crude product was purified using a silica gel column with an appropriate solvent system (RF values quoted in product data). The eluent fractions of interest were evaporated under vacuum to leave a white solid which was recrystallised from hot ethanol (50 mL).

Table SI26. Quantities of reagents used in the syntheses of the 3-methoxy-4-((4-nitrophenoxy)carbonyl)phenyl 2-alkoxy-4-methylbenzoates

m	(**15.1**)	4-Nitrophenol	4-Dimethylaminopyridine	*N,N'*-Dicyclohexylcarbodiimide
2	0.30 g, 9.1×10^{-4} mol	0.12 g, 8.3×10^{-4} mol	0.010 g, 8.3×10^{-5} mol	0.22 g, 1.08×10^{-3} mol
3	0.30 g, 8.7×10^{-4} mol	0.11 g, 7.9×10^{-4} mol	0.010 g, 7.9×10^{-5} mol	0.21 g, 1.03×10^{-3} mol
4	0.30 g, 8.4×10^{-4} mol	0.11 g, 7.6×10^{-4} mol	0.009 g, 7.6×10^{-5} mol	0.20 g, 9.9×10^{-4} mol
5	0.30 g, 8.1×10^{-4} mol	0.10 g, 7.4×10^{-4} mol	0.009 g, 7.4×10^{-5} mol	0.20 g, 9.6×10^{-4} mol
6	0.30 g, 7.8×10^{-4} mol	0.10 g, 7.1×10^{-4} mol	0.009 g, 7.6×10^{-5} mol	0.19 g, 9.2×10^{-4} mol
7	0.30 g, 7.5×10^{-4} mol	0.09 g, 6.8×10^{-4} mol	0.009 g, 7.4×10^{-5} mol	0.18 g, 8.8×10^{-4} mol

16.1.1 3-Methoxy-4-((4-nitrophenoxy)carbonyl)phenyl 2-ethoxy-4-methylbenzoate (E.2)

Yield: 0.13 g, 33 %. RF: 0.28 (100 % dichloromethane).

T_{CrI} 148 °C T_{N_FI} (82 °C)

ν_{max}/cm^{-1}: 2977 (C-H), 1715 (C=O), 1592 (Ar C=C), 1572 (Ar C=C), 1522 (NO), 1351 (NO)

δ_H/ppm (400 MHz, $CDCl_3$): 8.35 (d, *J* = 8.7 Hz, 2H, Ar-H), 8.09 (d, *J* = 8.6 Hz, 1H, Ar-H), 7.86 (d, *J* = 7.7 Hz, 1H, Ar-H), 7.59 (d, *J* = 8.7 Hz, 2H, Ar-H), 7.18 (s, 1H, Ar-H), 7.06 (s, 1H, Ar-H), 7.02 (d, *J* = 8.6 Hz, 1H, Ar-H), 6.92 (d, *J* = 7.7 Hz, 1H, Ar-H), 4.15 (q, *J* = 6.9 Hz, 2H, $O\underline{CH_2}CH_3$), 3.90 (s, 3H, OCH_3), 2.39 (s, 3H, Ar-CH_3), 1.36 (t, *J* = 6.9 Hz, 3H, $OCH_2\underline{CH_3}$)

δ_C/ppm (100 MHz, $CDCl_3$): 163.61, 162.56, 161.80, 159.95, 156.88, 155.97, 146.29, 145.36, 133.79, 132.55, 125.28 (2C), 122.88 (2C), 121.25, 115.51, 114.84, 114.23, 114.09, 106.66, 64.70, 56.42, 22.22, 14.91

MS = $[M+H]^+$: Calculated for $C_{24}H_{22}NO_8$: 452.1353. Found: 452.1345; Difference: 1.8 ppm

16.1.2 3-Methoxy-4-((4-nitrophenoxy)carbonyl)phenyl 4-methyl-2-propoxybenzoate (E.3)

Yield: 0.13 g, 35 %. RF: 0.33 (100 % dichloromethane).

T_{CrI} 92 °C T_{N_FI} (62 °C)

ν_{max}/cm^{-1}: 2972 (C-H), 1750 (C=O), 1602 (Ar C=C), 1584 (Ar C=C), 1522 (NO), 1350 (NO)

δ_H/ppm (400 MHz, $CDCl_3$): 8.31 (d, *J* = 9.2 Hz, 2H, Ar-H), 8.12 (d, *J* = 9.1 Hz, 1H, Ar-H), 7.93 (d, *J* = 7.8 Hz, 1H, Ar-H), 7.41 (d, *J* = 9.2 Hz, 2H, Ar-H), 6.97 – 6.92 (m, 2H, Ar-H), 6.88 – 6.83 (m, 2H, Ar-H), 4.05 (t, *J* = 6.4 Hz, 2H, $O\underline{CH_2}CH_2CH_3$), 3.95 (s, 3H, OCH_3), 2.43 (s, 3H, Ar-CH_3), 1.87 (sext, 2H, *J* = 6.9 Hz, $OCH_2\underline{CH_2}CH_3$), 1.08 (t, *J* = 7.4 Hz, 3H, $OCH_2CH_2\underline{CH_3}$)

δ_C/ppm (100 MHz, $CDCl_3$): 163.78, 162.57, 161.81, 160.03, 156.90, 155.97, 146.27, 145.36, 133.81, 132.63, 125.28 (2C), 122.88 (2C), 121.16, 115.47, 114.84, 114.10, 114.07, 106.64, 70.49, 56.41, 22.74, 22.21, 10.75

MS = $[M+H]^+$: Calculated for $C_{25}H_{24}NO_8$: 466.1525. Found: 466.1502; Difference: 4.9 ppm

16.1.3 3-Methoxy-4-((4-nitrophenoxy)carbonyl)phenyl 2-butoxy-4-methylbenzoate (E.4)

Yield: 0.14 g, 37 %. RF: 0.40 (100 % dichloromethane).

T_{CrI} 106 °C T_{N_FI} (41 °C)

ν_{max}/cm^{-1}: 2957 (C-H), 1720 (C=O), 1603 (Ar C=C), 1584 (Ar C=C), 1518 (NO), 1347 (NO)

δ_H/ppm (400 MHz, $CDCl_3$): 8.31 (d, *J* = 8.3 Hz, 2H, Ar-H), 8.12 (d, *J* = 8.8 Hz, 1H, Ar-H), 7.93 (d, *J* = 7.9 Hz, 1H, Ar-H), 7.41 (d, *J* = 8.4 Hz, 2H, Ar-H), 6.98 – 6.92 (m, 2H, Ar-H), 6.88 – 6.82 (m, 2H, Ar-H), 4.09 (t, *J* = 6.4 Hz, 2H, $O\underline{CH_2}(CH_2)_2CH_3$), 3.95 (s, 3H, OCH_3), 2.43 (s, 3H, Ar-CH_3), 1.84 (quin, *J* = 6.8 Hz, 2H, $OCH_2\underline{CH_2}CH_2CH_3$), 1.54 (sext, *J* = 7.3 Hz, 2H, $O(CH_2)_2\underline{CH_2}CH_3$), 0.96 (t, *J* = 7.3 Hz, 3H, $O(CH_2)_3\underline{CH_3}$)

δ_C/ppm (100 MHz, $CDCl_3$): 163.76, 162.56, 161.81, 160.04, 156.90, 155.97, 146.27, 145.36, 133.81, 132.63, 125.28 (2C), 122.88 (2C), 121.14, 115.47, 114.84, 114.09, 114.05, 106.63, 68.67, 56.40, 31.39, 22.21, 19.33, 13.95

MS = $[M+Na]^+$: Calculated for $C_{26}H_{26}NO_8$: 480.1664. Found: 480.1658; Difference: 1.2 ppm

16.1.4 3-Methoxy-4-((4-nitrophenoxy)carbonyl)phenyl 4-methyl-2-pentoxybenzoate (E.5)

Yield: 0.10 g, 28 %. RF: 0.45 (100 % dichloromethane).

T_{CrI} 78 °C T_{N_FI} (31 °C)

ν_{max}/cm^{-1}: 2936 (C-H), 1744 (C=O), 1601 (Ar C=C), 1581 (Ar C=C), 1522 (NO), 1351 (NO)

δ_H/ppm (400 MHz, $CDCl_3$): 8.31 (d, *J* = 9.0 Hz, 2H, Ar-H), 8.13 (d, *J* = 9.2 Hz, 1H, Ar-H), 7.93 (d, *J* = 7.8 Hz, 1H, Ar-H), 7.41 (d, *J* = 9.0 Hz, 2H, Ar-H), 6.98 – 6.92 (m, 2H, Ar-H), 6.88 – 6.81 (m, 2H, Ar-H), 4.08 (t, *J* = 6.5 Hz, 2H, $O\underline{CH_2}(CH_2)_3CH_3$), 3.95 (s, 3H, OCH_3), 2.43 (s, 3H, Ar-CH), 1.86 (quin, *J* = 6.7 Hz, 2H, $OCH_2\underline{CH_2}(CH_2)_2CH_3$), 1.53 – 1.31 (m, 4H, $OCH_2CH_2(\underline{CH_2})_2CH_3$), 0.90 (t, *J* = 7.2 Hz, 3H, $O(CH_2)_4\underline{CH_3}$)

δ_C/ppm (100 MHz, $CDCl_3$): 163.81, 162.57, 161.82, 160.02, 156.91, 155.97, 146.27, 145.37, 133.81, 132.65, 125.29 (2C), 122.89 (2C), 121.16, 115.49, 114.84, 114.10, 114.07, 106.64, 69.01, 56.41, 29.04, 28.27, 22.52, 22.22, 14.14

MS = $[M+H]^+$: Calculated for $C_{27}H_{28}NO_8$: 494.1811. Found: 494.1815; Difference: -0.8 ppm

16.1.5 3-Methoxy-4-((4-nitrophenoxy)carbonyl)phenyl 2-hexyloxy-4-methylbenzoate (E.6)

Yield: 0.17 g, 47 %. RF: 0.40 (100 % dichloromethane).

T_{CrI} 107 °C T_{N_FI} (17 °C)

ν_{max}/cm^{-1}: 2928 (C-H), 1719 (C=O), 1592 (Ar C=C), 1581 (Ar C=C), 1524 (NO), 1349 (NO)

δ_H/ppm (400 MHz, $CDCl_3$): 8.32 (d, *J* = 9.0 Hz, 2H, Ar-H), 8.12 (d, *J* = 9.2 Hz, 1H, Ar-H), 7.93 (d, *J* = 7.8 Hz, 1H, Ar-H), 7.42 (d, *J* = 9.1 Hz, 2H, Ar-H), 6.97 – 6.92 (m, 2H, Ar-H), 6.90 – 6.81 (m, 2H, Ar-H), 4.08 (t, *J* = 6.5 Hz, 2H, $O\underline{CH_2}(CH_2)_4CH_3$), 3.95 (s, 3H, OCH_3), 2.43 (s, 3H, Ar-CH_3), 1.84 (quin, *J* = 6.8 Hz, 2H, $OCH_2\underline{CH_2}(CH_2)_3CH_3$), 1.49 (quin, *J* = 7.2 Hz, 2H, $O(CH_2)_2\underline{CH_2}(CH_2)_2CH_3$), 1.36 – 1.22 (m, 4H, $O(CH_2)_3(\underline{CH_2})_2CH_3$), 0.87 (t, *J* = 7.2 Hz, 3H, $O(CH_2)_5\underline{CH_3}$)

δ_C/ppm (100 MHz, $CDCl_3$): 163.82, 162.57, 161.82, 160.03, 156.92, 155.98, 146.26, 145.38, 133.80, 132.65, 125.29 (2C), 122.89 (2C), 121.16, 115.50, 114.84, 114.10, 114.08, 106.64, 69.02, 56.41, 31.64, 29.32, 25.79, 22.70, 22.22, 14.13

MS = $[M+H]^+$: Calculated for $C_{28}H_{30}NO_8$: 508.1995. Found: 508.1971; Difference: 4.7 ppm

16.1.6 3-Methoxy-4-((4-nitrophenoxy)carbonyl)phenyl 2-heptyloxy-4-methylbenzoate (E.7)

Yield: 0.19 g, 56 %. RF: 0.45 (100 % dichloromethane).

T_{CrI} 110 °C T_{N_FI} (14 °C)

v_{max}/cm^{-1}: 2920 (C-H), 1736 (C=O), 1592 (Ar C=C), 1572 (Ar C=C), 1524 (NO), 1346 (NO)

δ_H/ppm (400 MHz, $CDCl_3$): 8.31 (d, *J* = 9.1 Hz, 2H, Ar-H), 8.12 (d, *J* = 9.1 Hz, 1H, Ar-H), 7.92 (d, *J* = 7.9 Hz, 1H, Ar-H), 7.41 (d, *J* = 9.1 Hz, 2H, Ar-H), 6.97 – 6.92 (m, 2H, Ar-H), 6.87 – 6.81 (m, 2H, Ar-H), 4.08 (t, *J* = 6.5 Hz, 2H, $O\underline{CH_2}(CH_2)_5CH_3$), 3.95 (s, 3H, OCH_3), 2.43 (s, 3H, Ar-CH_3), 1.85 (quin, *J* = 6.7 Hz, 2H, $OCH_2\underline{CH_2}(CH_2)_4CH_3$), 1.48 (quin, *J* = 7.2 Hz, 2H, $O(CH_2)_2\underline{CH_2}(CH_2)_3CH_3$), 1.39 – 1.18 (m, 6H, $O(CH_2)_3\underline{(CH_2)_3}CH_3$), 0.86 (t, *J* = 7.2 Hz, 3H, $O(CH_2)_6\underline{CH_3}$)

δ_C/ppm (100 MHz, $CDCl_3$): 163.83, 162.57, 161.82, 160.02, 156.92, 155.98, 146.26, 145.38, 133.80, 132.64, 125.29, 122.88, 121.16, 115.51, 114.84, 114.09, 114.08, 106.63, 69.02, 56.41, 31.89, 29.37, 29.14, 26.08, 22.71, 22.22, 14.20

MS = $[M+H]^+$: Calculated for $C_{29}H_{32}NO_8$: 522.2116. Found: 522.2128; Difference: -2.3 ppm

16.2 3-Methoxy-4-((3-fluoro-4-nitrophenoxy)carbonyl)phenyl 2-alkoxy-4-methylbenzoates (F.m)

To a pre-dried flask flushed with argon **Compound 15.1** (1 eq), 3-fluoro-4-nitrophenol (0.91 eq) and *N,N'*-dicyclohexylcarbodiimide (1.18 eq) were added. The solids were solubilised with dichloromethane (50 mL) and stirred for 2 min before 4-dimethylaminopyridine (0.091 eq) was added to the flask. The quantities of the reagents used in each reaction are listed in **Table SI27**. The temperature of the reaction mixture was increased to room temperature and the reaction was allowed to proceed overnight. A white precipitate which formed was removed by vacuum filtration and the filtrate collected. The solvent was removed under vacuum and the crude product was purified using a silica gel column with an appropriate solvent system (RF values quoted in product data). The eluent fractions of interest were evaporated under vacuum to leave a white solid which was recrystallised from hot ethanol (50 mL).

Table SI27. Quantities of reagents used in the syntheses of the 3-methoxy-4-((3-fluoro-4-nitrophenoxy)carbonyl)phenyl 2-alkoxy-4-methylbenzoates

m	(**15.1**)	3-Fluoro-4-nitrophenol	4-Dimethylaminopyridine	*N,N'*-Dicyclohexylcarbodiimide
2	0.30 g, 9.1×10^{-4} mol	0.13 g, 8.3×10^{-4} mol	0.010 g, 8.3×10^{-5} mol	0.22 g, 1.08×10^{-3} mol
3	0.30 g, 8.7×10^{-4} mol	0.12 g, 7.9×10^{-4} mol	0.010 g, 7.9×10^{-5} mol	0.21 g, 1.03×10^{-3} mol
4	0.30 g, 8.4×10^{-4} mol	0.12 g, 7.6×10^{-4} mol	0.009 g, 7.6×10^{-5} mol	0.20 g, 9.9×10^{-4} mol
5	0.30 g, 8.1×10^{-4} mol	0.12 g, 7.4×10^{-4} mol	0.009 g, 7.4×10^{-5} mol	0.20 g, 9.6×10^{-4} mol
6	0.30 g, 7.8×10^{-4} mol	0.11 g, 7.1×10^{-4} mol	0.009 g, 7.6×10^{-5} mol	0.19 g, 9.2×10^{-4} mol
7	0.30 g, 7.5×10^{-4} mol	0.11 g, 6.8×10^{-4} mol	0.009 g, 7.4×10^{-5} mol	0.18 g, 8.8×10^{-4} mol

16.2.1 3-Methoxy-4-((3-fluoro-4-nitrophenoxy)carbonyl)phenyl 2-ethoxy-4-methylbenzoate (E.2)

Yield: 0.15 g, 39 %. RF: 0.30 (100 % dichloromethane).

T_{CrI} 123 °C T_{N_FI} (87 °C)

ν_{max}/cm^{-1}: 2983 (C-H), 1721 (C=O), 1600 (Ar C=C), 1584 (Ar C=C), 1525 (NO), 1347 (NO)

δ_H/ppm (400 MHz, $CDCl_3$): 8.17 (app t, *J* = 8.7 Hz, 1H, Ar-H), 8.09 (d, *J* = 8.4 Hz, 1H, Ar-H), 7.93 (d, *J* = 7.8 Hz, 1H, Ar-H), 7.28 (dd, *J* = 11.6, 2.3 Hz, 1H, Ar-H), 7.21 (ddd, *J* = 9.1, 2.3, 1.3 Hz, 1H, Ar-H), 6.98 – 6.94 (m, 2H, Ar-H), 6.89 – 6.82 (m, 2H, Ar-H), 4.16 (q, *J* = 7.0 Hz, 2H, $O\underline{CH_2}CH_3$), 3.95 (s, 3H, OCH_3), 2.42 (s, 3H, Ar-CH_3), 1.49 (t, *J* = 7.0 Hz, 3H, $OCH_2\underline{CH_3}$)

δ_C/ppm (100 MHz, $CDCl_3$): 163.56, 162.02, 161.93, 159.97, 157.65, 157.11, 156.16, 156.06, 155.00, 146.35, 134.77, 134.70, 133.84, 132.56, 127.22, 127.20, 121.26, 118.36, 118.32, 115.45, 114.33, 114.23, 114.16, 112.68, 112.44, 106.69, 64.70, 56.44, 22.22, 14.90

MS = $[M+H]^+$: Calculated for $C_{24}H_{21}NO_8F$: 470.1261. Found: 470.1251; Difference: 2.1 ppm.

16.2.2 3-Methoxy-4-((3-fluoro-4-nitrophenoxy)carbonyl)phenyl 4-methyl-2-propoxybenzoate (E.3)

Yield: 0.13 g, 35 %. RF: 0.35 (100 % dichloromethane).

T_{CrI} 111 °C T_{N_FI} (68 °C)

ν_{max}/cm^{-1}: 2972 (C-H), 1748 (C=O), 1603 (Ar C=C), 1585 (Ar C=C), 1524 (NO), 1350 (NO)

δ_H/ppm (400 MHz, $CDCl_3$): 8.18 (app t, *J* = 8.6 Hz, 1H, Ar-H), 8.10 (d, *J* = 9.1 Hz, 1H, Ar-H), 7.93 (d, *J* = 7.8 Hz, 1H, Ar-H), 7.32 – 7.18 (m, 2H, Ar-H), 6.99 – 6.92 (m, 2H, Ar-H), 6.89 – 6.81 (m, 2H, Ar-H), 4.05 (t, *J* = 6.4 Hz, 2H, $O\underline{CH_2}CH_2CH_3$), 3.95 (s, 3H, OCH_3), 2.43 (s, 3H, Ar-CH_3), 1.88 (sext, 2H, *J* = 6.9 Hz, $OCH_2\underline{CH_2}CH_3$), 1.08 (t, *J* = 7.4 Hz, 3H, $OCH_2CH_2\underline{CH_3}$)

δ_C/ppm (100 MHz, $CDCl_3$): 163.72, 162.02, 161.95, 160.06, 157.65, 157.14, 156.16, 156.06, 155.00, 146.33, 134.77, 134.70, 133.86, 132.64, 127.22, 127.20, 121.17, 118.35, 118.31, 115.40, 114.34, 114.16, 114.07, 112.68, 112.44, 106.67, 70.49, 56.42, 22.74, 22.22, 10.75

MS = $[M+H]^+$: Calculated for $C_{25}H_{23}NO_8F$: 466.1525. 484.1417. Found: 484.1408; Difference: 1.9 ppm

16.2.3 3-Methoxy-4-((3-fluoro-4-nitrophenoxy)carbonyl)phenyl 2-butoxy-4-methylbenzoate (E.4)

Yield: 0.13 g, 34 %. RF: 0.48 (100 % dichloromethane).

T_{CrI} 96 °C T_{N_FI} (51 °C)

ν_{max}/cm^{-1}: 2970 (C-H), 1739 (C=O), 1601 (Ar C=C), 1583 (Ar C=C), 1526 (NO), 1352 (NO)

δ_H/ppm (400 MHz, $CDCl_3$): 8.18 (app t, *J* = 8.6 Hz, 1H, Ar-H), 8.10 (d, *J* = 9.2 Hz, 1H, Ar-H), 7.93 (d, *J* = 8.0 Hz, 1H, Ar-H), 7.31 – 7.18 (m, 2H, Ar-H), 6.97 – 6.93 (m, 2H, Ar-H), 6.88 – 6.83 (m, 2H, Ar-H), 4.09 (t, *J* = 6.4 Hz, 2H, $O\underline{CH_2}(CH_2)_2CH_3$), 3.96 (s, 3H, OCH_3), 2.43 (s, 3H, Ar-CH_3), 1.84 (quin, *J* = 6.7 Hz, 2H, $OCH_2\underline{CH_2}CH_2CH_3$), 1.54 (sext, *J* = 7.3 Hz, 2H, $O(CH_2)_2\underline{CH_2}CH_3$), 0.96 (t, *J* = 7.4 Hz, 3H, $O(CH_2)_3\underline{CH_3}$)

δ_C/ppm (100 MHz, $CDCl_3$): 163.71, 162.04, 161.95, 160.07, 157.66, 157.15, 156.18, 156.07, 155.02, 146.33, 134.79, 134.72, 133.86, 132.64, 127.27, 127.21, 121.17, 118.36, 118.32, 115.43, 114.36, 114.17, 114.07, 112.68, 112.45, 106.68, 68.69, 56.43, 31.41, 22.23, 19.34, 13.95

MS = $[M+H]^+$: Calculated for $C_{26}H_{25}NO_8F$: 498.1553. Found: 498.1564; Difference: -2.2 ppm

16.2.4 3-Methoxy-4-((3-fluoro-4-nitrophenoxy)carbonyl)phenyl 4-methyl-2-pentoxybenzoate (E.5)

Yield: 0.09 g, 23 %. RF: 0.50 (100 % dichloromethane).

T_{CrI} 87 °C T_{N_FI} (39 °C)

v_{max}/cm^{-1}: 2963 (C-H), 1734 (C=O), 1602 (Ar C=C), 1583 (Ar C=C), 1527 (NO), 1356 (NO)

δ_H/ppm (400 MHz, $CDCl_3$): 8.18 (app t, *J* = 8.7 Hz, 1H, Ar-H), 8.10 (d, *J* = 9.2 Hz, 1H, Ar-H), 7.92 (d, *J* = 7.8 Hz, 1H, Ar-H), 7.31 – 7.18 (m, 2H, Ar-H), 6.98 – 6.91 (m, 2H, Ar-H), 6.89 – 6.81 (m, 2H, Ar-H), 4.08 (t, *J* = 6.5 Hz, 2H, $O\underline{CH_2}(CH_2)_3CH_3$), 3.96 (s, 3H, OCH_3), 2.43 (s, 3H, Ar-CH_3), 1.86 (quin, *J* = 6.7 Hz, 2H, $OCH_2\underline{CH_2}(CH_2)_2CH_3$), 1.53 – 1.30 (m, 4H, $OCH_2CH_2(\underline{CH_2})_2CH_3$), 0.89 (t, *J* = 7.2 Hz, 3H, $O(CH_2)_4\underline{CH_3}$)

δ_C/ppm (100 MHz, $CDCl_3$): 163.76, 162.03, 161.95, 160.05, 157.66, 157.15, 156.17, 156.06, 155.02, 146.33, 134.78, 134.71, 133.86, 132.66, 127.23, 127.21, 121.17, 118.36, 118.32, 115.42, 114.34, 114.17, 114.07, 112.69, 112.45, 106.68, 69.01, 56.43, 29.04, 28.27, 22.52, 22.23, 14.14

MS = $[M+H]^+$: Calculated for $C_{27}H_{27}NO_8F$: 512.1722. Found: 512.1721; Difference: 0.2 ppm

16.2.5 3-Methoxy-4-((3-fluoro-4-nitrophenoxy)carbonyl)phenyl 2-hexyloxy-4-methylbenzoate (E.6)

Yield: 0.08 g, 22 %. RF: 0.48 (100 % dichloromethane).

T_{CrI} 81 °C T_{N_FI} (29 °C)

v_{max}/cm^{-1}: 2930 (C-H), 1743 (C=O), 1601 (Ar C=C), 1578 (Ar C=C), 1521 (NO), 1343 (NO)

δ_H/ppm (400 MHz, $CDCl_3$): 8.18 (app t, *J* = 8.7 Hz, 1H, Ar-H), 8.10 (d, *J* = 8.8 Hz, 1H, Ar-H), 7.92 (d, *J* = 7.8 Hz, 1H, Ar-H), 7.30 – 7.25 (m, 1H, Ar-H), 7.21 (ddd, *J* = 9.1, 2.2, 1.2 Hz, 1H, Ar-H), 6.98 – 6.92 (m, 2H, Ar-H), 6.90 – 6.81 (m, 2H, Ar-H), 4.08 (t, *J* = 6.5 Hz, 2H, $O\underline{CH_2}(CH_2)_4CH_3$), 3.95 (s, 3H, OCH_3), 2.43 (s, 3H, Ar-CH_3), 1.85 (quin, *J* = 6.7 Hz, 2H, $OCH_2\underline{CH_2}(CH_2)_3CH_3$), 1.50 (quin, *J* = 7.2 Hz, 2H, $O(CH_2)_2\underline{CH_2}(CH_2)_2CH_3$), 1.38 – 1.24 (m, 4H, $O(CH_2)_3(\underline{CH_2})_2CH_3$), 0.87 (t, *J* = 7.2 Hz, 3H, $O(CH_2)_5\underline{CH_3}$)

δ_C/ppm (100 MHz, $CDCl_3$): 163.76, 162.03, 161.95, 160.04, 157.66, 157.15, 156.17, 156.06, 155.01, 146.32, 134.77, 134.70, 133.85, 132.65, 127.23, 127.21, 121.16, 118.36, 118.32, 115.42, 114.33, 114.16, 114.07, 112.68, 112.44, 106.67, 69.02, 56.42, 31.63, 29.31, 25.79, 22.70, 22.22, 14.13

MS = $[M+H]^+$: Calculated for $C_{28}H_{29}NO_8F$: 526.1888. Found: 526.1877; Difference: 2.1 ppm

16.2.6 3-Methoxy-4-((3-fluoro-4-nitrophenoxy)carbonyl)phenyl 2-heptyloxy-4-methylbenzoate (E.7)

Yield: 0.07 g, 19 %. RF: 0.50 (100 % dichloromethane).

T_{CrI} 85 °C T_{N_FI} (27 °C)

v_{max}/cm^{-1}: 2920 (C-H), 1744 (C=O), 1602 (Ar C=C), 1568 (Ar C=C), 1524 (NO), 1350 (NO)

δ_H/ppm (400 MHz, $CDCl_3$): 8.31 (appt t, *J* = 8.7 Hz, 1H, Ar-H), 8.10 (d, *J* = 8.9 Hz, 1H, Ar-H), 7.92 (d, *J* = 7.8 Hz, 1H, Ar-H), 7.31 – 7.26 (m, 1H, Ar-H), 7.21 (ddd, *J* = 9.1, 2.3, 1.3 Hz, 1H, Ar-H), 6.97 – 6.92 (m,

2H, Ar-H), 6.87 – 6.82 (m, 2H, Ar-H), 4.08 (t, *J* = 6.5 Hz, 2H, O$\underline{CH_2}$$(CH_2)_5CH_3$), 3.96 (s, 3H, OCH_3), 2.43 (s, 3H, Ar-CH_3), 1.85 (quin, *J* = 6.7 Hz, 2H, $OCH_2\underline{CH_2}(CH_2)_4CH_3$), 1.49 (quin, *J* = 7.0 Hz, 2H, $O(CH_2)_2\underline{CH_2}(CH_2)_3CH_3$), 1.39 – 1.20 (m, 6H, $O(CH_2)_3(\underline{CH_2})_3CH_3$), 0.86 (t, *J* = 7.2 Hz, 3H, $O(CH_2)_6\underline{CH_3}$)

δ_C/ppm (100 MHz, $CDCl_3$): 163.83, 162.57, 161.82, 160.02, 156.92, 155.98, 146.26, 145.38, 133.80, 132.64, 125.29, 122.88, 121.16, 115.51, 114.84, 114.09, 114.08, 106.63, 69.02, 56.41, 31.89, 29.37, 29.14, 26.08, 22.71, 22.22, 14.20

MS = $[M+H]^+$: Calculated for $C_{29}H_{31}NO_8F$: 540.2039. Found: 540.2034; Difference: 0.9 ppm

16.3 3-Methoxy-4-((4-nitrophenoxy)carbonyl)phenyl 2-alkyloxy-4-methoxybenzoates (I.m)

To a pre-dried flask flushed with argon, **Compound 15.2** (1 eq) and *N,N'*-dicyclohexylcarbodiimide (1.5 eq) were added to the flask. The solids were solubilised with dichloromethane (30 mL) and stirred for 10 min before 4-nitrophenol (1.2 eq) was added then another 15 min before 4-dimethylaminopyridine (0.15 eq) was added. The quantities of the reagents used in each reaction are listed in **Table SI28**. The reaction was allowed to proceed overnight. The white precipitate which formed was removed by vacuum filtration and the filtrate collected. The solvent was removed under vacuum and the crude product was purified using a silica gel column with an appropriate solvent system (RF values quoted in product data). The eluent fractions of interest were evaporated under vacuum to leave a white solid which was recrystallised from hot ethanol (50 mL).

Table SI28. Quantities of reagents used in the syntheses of the 3-methoxy-4-((4-nitrophenoxy)carbonyl)phenyl 2-alkoxy-4-methoxybenzoates

m	(**15.2**)	4-Nitrophenol	4-Dimethylaminopyridine	*N,N'*-Dicyclohexylcarbodiimide
3	0.150 g, 4.16×10^{-4} mol	0.070 g, 5.00×10^{-4} mol	0.008 g, 6.24×10^{-5} mol	0.129 g, 6.24×10^{-4} mol
4	0.150 g, 4.01×10^{-4} mol	0.067 g, 4.81×10^{-4} mol	0.007 g, 6.02×10^{-5} mol	0.124 g, 6.02×10^{-4} mol
5	0.300 g, 7.72×10^{-4} mol	0.129 g, 9.27×10^{-4} mol	0.014 g, 1.16×10^{-4} mol	0.239 g, 1.16×10^{-3} mol
6	0.250 g, 6.21×10^{-4} mol	0.104 g, 7.45×10^{-4} mol	0.011 g, 9.32×10^{-5} mol	0.192 g, 9.32×10^{-4} mol
7	0.300 g, 7.20×10^{-4} mol	0.120 g, 8.64×10^{-4} mol	0.013 g, 1.08×10^{-4} mol	0.223 g, 1.08×10^{-3} mol

16.3.1 3-Methoxy-4-((4-nitrophenoxy)carbonyl)phenyl 4-methyoxy-2-propoxybenzoate (I.3)

Yield: 0.042 g, 21.0 %. RF: 0.22 (100 % dichloromethane).

T_{CrI} 134 °C T_{N_FI} (62 °C)

ν_{max}/cm^{-1}: 2977 ($-CH_3$), 2900 ($-CH_2$), 1747 (COOR), 1605 (Ar C=C), 1517 (NO_2), 1232 (C-O-C)

δ_H/ppm (400 MHz, $CDCl_3$): 8.31 (d, *J* = 9.2 Hz, 2H, Ar-H), 8.12 (m, 1H, Ar-H), 8.06 (d, *J* = 8.8 Hz, 1H, Ar-H), 7.41 (d, *J* = 9.2 Hz, 2H, Ar-H), 6.94 (m, 2H, Ar-H), 6.57 (dd, *J* = 8.8, 2.3 Hz, 1H, Ar-H), 6.52 (d, *J* = 2.3 Hz, 1H, Ar-H), 4.03 (t, *J* = 6.4 Hz, 2H, $O\underline{CH_2}CH_2CH_3$), 3.95 (s, 3H, OCH_3), 3.89 (s, 3H, OCH_3), 1.89 (m, 2H, $OCH_2\underline{CH_2}CH_3$), 1.08 (t, *J* = 7.4 Hz, 3H, $OCH_2CH_2\underline{CH_3}$)

δ_C/ppm (100 MHz, $CDCl_3$): 165.27, 163.12, 162.46, 161.95, 161.69, 156.87, 155.86, 145.25, 134.64, 133.68, 125.17, 122.76, 114.63, 114.04, 110.61, 106.58, 105.02, 99.71, 70.44, 56.29, 55.61, 22.53, 10.65

MS = $[M+H]^+$: Calculated for $C_{25}H_{24}NO_9$: 482.1451. Found: 482.1474; Difference: 4.8 ppm

16.3.2 3-Methoxy-4-((4-nitrophenoxy)carbonyl)phenyl 2-butoxy-4-methyoxybenzoate (I.4)

Yield: 0.034 g, 17.1 %. RF: 0.20 (100 % dichloromethane).

T_{CrI} 133 °C T_{N_FI} (42 °C)

ν_{max}/cm^{-1}: 2958 ($-CH_3$), 1748 (COOR), 1702 (COOR), 1613 (Ar C=C), 1516 (NO_2), 1270 (C-O-C)

δ_H/ppm (400 MHz, $CDCl_3$): 8.31 (d, *J* = 9.2 Hz, 2H, Ar-H), 8.11 (m, 1H, Ar-H), 8.05 (d, *J* = 8.7 Hz, 1H, Ar-H), 7.41 (d, *J* = 9.1 Hz, 2H, Ar-H), 6.94 (m, 2H, Ar-H), 6.57 (dd, *J* = 8.7, 2.3 Hz, 1H, Ar-H), 6.52 (d, *J* = 2.3 Hz, 1H, Ar-H), 4.06 (t, *J* = 6.4 Hz, 2H, $O\underline{CH_2}(CH_2)_2CH_3$), 3.95 (s, 3H, OCH_3), 3.89 (s, 3H, OCH_3), 1.83 (m, 2H, $OCH_2\underline{CH_2}CH_2CH_3$), 1.54 (m, 2H, $O(CH_2)_2\underline{CH_2}CH_3$), 0.98 (t, *J* = 7.4 Hz, 3H, $O(CH_2)_3\underline{CH_3}$)

δ_C/ppm (100 MHz, $CDCl_3$): 165.27, 163.14, 162.45, 161.90, 161.70, 156.89, 155.86, 145.23, 134.66, 133.67, 125.18, 122.77, 114.63, 114.04, 110.62, 106.58, 105.01, 99.71, 68.67, 56.29, 55.61, 31.10, 19.32, 13.90

MS = $[M+H]^+$: Calculated for $C_{26}H_{26}NO_9$: 496.1608. Found: 496.1619; Difference: 2.2 ppm

16.3.3 3-Methoxy-4-((4-nitrophenoxy)carbonyl)phenyl 4-methoxy-2-pentoxybenzoate (I.5)

Yield: 0.077 g, 19.6 %. RF: 0.29 (100 % dichloromethane).

T_{CrI} 137 °C T_{N_FI} (34 °C)

ν_{max}/cm^{-1}: 2955 ($-CH_3$), 2930 ($-CH_2$), 2871 ($-CH_2$), 1748 (COOR), 1702 (COOR), 1612 (Ar C=C), 1515 (NO_2), 1265 (C-O-C)

δ_H/ppm (400 MHz, $CDCl_3$): 8.31 (d, *J* = 9.1 Hz, 2H, Ar-H), 8.12 (m, 1H, Ar-H), 8.05 (d, *J* = 8.8 Hz, 1H, Ar-H), 7.41 (d, *J* = 9.1 Hz, 2H, Ar-H), 6.94 (m, 2H, Ar-H), 6.57 (dd, *J* = 8.8, 2.4 Hz, 1H, Ar-H), 6.52 (d, *J* = 2.4 Hz, 1H, Ar-H), 4.06 (t, *J* = 6.5 Hz, 2H, $O\underline{CH_2}(CH_2)_3CH_3$), 3.95 (s, 3H, OCH_3), 3.89 (s, 3H, OCH_3), 1.86 (m, 2H, $OCH_2\underline{CH_2}(CH_2)_2CH_3$), 1.49 (m, 2H, $OCH_2CH_2\underline{CH_2}CH_2CH_3$), 1.37 (m, 2H, $OCH_2CH_2CH_2\underline{CH_2}CH_3$), 0.89 (t, *J* = 7.3 Hz, 3H, $O(CH_2)_4\underline{CH_3}$)

δ_C/ppm (100 MHz, $CDCl_3$): 165.27, 163.16, 162.46, 161.93, 161.70, 156.88, 155.86, 145.25, 134.66, 133.67, 125.17, 122.77, 114.62, 114.04, 110.62, 106.58, 105.00, 99.72, 68.97, 56.29, 55.61, 28.81, 28.14, 22.40, 14.02

MS = $[M+H]^+$: Calculated for $C_{27}H_{28}NO_9$: 510.1764. Found: 510.1770; Difference: 1.2 ppm

16.3.4 3-Methoxy-4-((4-nitrophenoxy)carbonyl)phenyl 2-hexyloxy-4-methoxybenzoate (I.6)

Yield: 0.125 g, 38.3 %. RF: 0.29 (100 % dichloromethane).

T_{CrI} 121 °C T_{N_FI} (23 °C)

ν_{max}/cm^{-1}: 2920 ($-CH_2$), 1749 (COOR), 1702 (COOR), 1610 (Ar C=C), 1520 (NO_2), 1265 (C-O-C)

δ_H/ppm (400 MHz, $CDCl_3$): 8.31 (d, *J* = 9.2 Hz, 2H, Ar-H), 8.12 (m, 1H, Ar-H), 8.05 (d, *J* = 8.8 Hz, 1H, Ar-H), 7.42 (d, *J* = 9.1 Hz, 2H, Ar-H), 6.94 (m, 2H, Ar-H), 6.56 (dd, *J* = 8.8, 2.3 Hz, 1H, Ar-H), 6.52 (d, *J* = 2.3 Hz, 1H, Ar-H), 4.06 (t, *J* = 6.5 Hz, 2H, $O\underline{CH_2}(CH_2)_4CH_3$), 3.95 (s, 3H, OCH_3), 3.89 (s, 3H, OCH_3), 1.86 (m, 2H, $OCH_2\underline{CH_2}(CH_2)_3CH_3$), 1.50 (m, 2H, $O(CH_2)_2\underline{CH_2}(CH_2)_2CH_3$), 1.31 (m, 4H, $O(CH_2)_3(\underline{CH_2})_2CH_3$), 0.87 (t, *J* = 7.2 Hz, 3H, $O(CH_2)_5\underline{CH_3}$)

δ_C/ppm (100 MHz, $CDCl_3$): 165.26, 163.16, 162.46, 161.93, 161.69, 156.88, 155.86, 145.25, 134.65, 133.66, 125.17, 122.77, 114.61, 114.03, 110.63, 106.58, 105.00, 99.73, 68.99, 56.29, 55.61, 31.51, 29.09, 25.67, 22.58, 14.02

MS = $[M+H]^+$: Calculated for $C_{28}H_{30}NO_9$: 524.1921. Found: 524.1936; Difference: 2.9 ppm

16.3.5 3-Methoxy-4-((4-nitrophenoxy)carbonyl)phenyl 2-heptyloxy-4-methoxybenzoate (I.7)

Yield: 0.155 g, 40.0 %. RF: 0.21 (100 % dichloromethane).

T_{CrI} 97 °C T_{N_FI} (24 °C)

ν_{max}/cm^{-1}: 2953 ($-CH_3$), 2912 ($-CH_2$), 1747 (COOR), 1717 (COOR), 1606 (Ar C=C), 1523 (NO_2), 1262 (C-O-C)

δ_H/ppm (400 MHz, $CDCl_3$): 8.31 (d, *J* = 9.2 Hz, 2H, Ar-H), 8.12 (m, 1H, Ar-H), 8.05 (d, *J* = 8.7 Hz, 1H, Ar-H), 7.41 (d, *J* = 9.2 Hz, 2H, Ar-H), 6.94 (m, 2H, Ar-H), 6.56 (dd, *J* = 8.8, 2.3 Hz, 1H, Ar-H), 6.52 (d, *J* = 2.3 Hz, 1H, Ar-H), 4.06 (t, *J* = 6.5 Hz, 2H, $O\underline{CH_2}(CH_2)_5CH_3$), 3.95 (s, 3H, OCH_3), 3.89 (s, 3H, OCH_3), 1.86 (m, 2H, $OCH_2\underline{CH_2}(CH_2)_4CH_3$), 1.49 (m, 2H, $O(CH_2)_2\underline{CH_2}(CH_2)_3CH_3$), 1.29 (m, 6H, $O(CH_2)_3(\underline{CH_2})_3CH_3$), 0.86 (t, *J* = 7.0 Hz, 3H, $O(CH_2)_6\underline{CH_3}$)

δ_C/ppm (100 MHz, $CDCl_3$): 165.26, 163.18, 162.46, 161.92, 161.70, 156.88, 155.86, 145.25, 134.65, 133.66, 125.17, 122.76, 114.61, 114.03, 110.64, 106.57, 105.00, 99.73, 68.98, 56.29, 55.61, 31.76, 29.13, 29.01, 25.95, 22.59, 14.08

MS = $[M+H]^+$: Calculated for $C_{29}H_{32}NO_9$: 538.2077. Found: 538.2081; Difference: 0.7 ppm

16.4 3-Methoxy-4-((3-fluoro-4-nitrophenoxy)carbonyl)phenyl 2-alkyloxy-4-methoxybenzoates (J.m)

To a pre-dried flask flushed with argon, **Compound 15.2** (1 eq) and *N,N'*-dicyclohexylcarbodiimide (1.5 eq) were added to the flask. The solids were solubilised with dichloromethane (30 mL) and stirred for

10 min before 3-fluoro-4-nitrophenol (1.2 eq) was added then another 15 min before 4-dimethylaminopyridine (0.15 eq) was added. The quantities of the reagents used in each reaction are listed in **Table SI29**. The reaction was allowed to proceed overnight. The white precipitate which formed was removed by vacuum filtration and the filtrate collected. The solvent was removed under vacuum and the crude product was purified using a silica gel column with an appropriate solvent system (RF values quoted in product data). The eluent fractions of interest were evaporated under vacuum to leave a white solid which was recrystallised from hot ethanol (50 mL).

Table SI29. Quantities of reagents used in the syntheses of the 3-methoxy-4-((4-nitrophenoxy)carbonyl)phenyl 2-alkoxy-4-methoxybenzoates

m	(**15.2**)	3-Fluoro-4-nitrophenol	4-Dimethylaminopyridine	*N,N'*-Dicyclohexylcarbodiimide
3	0.150 g, 4.16×10^{-4} mol	0.079 g, 5.00×10^{-4} mol	0.008 g, 6.24×10^{-5} mol	0.129 g, 6.24×10^{-4} mol
4	0.150 g, 4.01×10^{-4} mol	0.076 g, 4.81×10^{-4} mol	0.007 g, 6.02×10^{-5} mol	0.124 g, 6.02×10^{-4} mol
5	0.300 g, 7.72×10^{-4} mol	0.146 g, 9.27×10^{-4} mol	0.014 g, 1.16×10^{-4} mol	0.239 g, 1.16×10^{-3} mol
6	0.250 g, 6.21×10^{-4} mol	0.117 g, 7.45×10^{-4} mol	0.011 g, 9.32×10^{-5} mol	0.192 g, 9.32×10^{-4} mol
7	0.300 g, 7.20×10^{-4} mol	0.136 g, 8.64×10^{-4} mol	0.013 g, 1.08×10^{-4} mol	0.223 g, 1.08×10^{-3} mol

16.4.1 3-Methoxy-4-((3-fluoro-4-nitrophenoxy)carbonyl)phenyl 4-methoxy-2-propoxybenzoate (J.3)

Yield: 0.040 g, 19.3 %. RF: 0.24 (100 % dichloromethane).

T_{CrI} 136 °C T_{N_FI} (66 °C)

ν_{max}/cm^{-1}: 2953 (-CH_3), 1745 (COOR), 1606 (Ar C=C), 1523 (NO_2), 1269 (C-O-C), 1210 (C-F)

δ_H/ppm (400 MHz, $CDCl_3$): 8.17 (appt t, *J* = 9.0 Hz, 1H, Ar-H), 8.10 (d, *J* = 8.6 Hz, 1H, Ar-H), 8.06 (d, *J* = 8.8 Hz, 1H, Ar-H), 7.29 (m, 1H, Ar-H), 7.21 (ddd, *J* = 9.0, 2.4, 1.3 Hz, 1H, Ar-H), 6.94 (m, 2H, Ar-H), 6.57 (dd, *J* = 8.8, 2.4 Hz, 1H, Ar-H), 6.53 (d, *J* = 2.4 Hz, 1H, Ar-H), 4.03 (t, *J* = 6.4 Hz, 2H, O$\underline{CH_2}$$CH_2CH_3$), 3.95 (s, 3H, OCH_3), 3.89 (s, 3H, OCH_3), 1.89 (m, 2H, $OCH_2$$\underline{CH_2}$$CH_3$), 1.08 (t, *J* = 7.4 Hz, 3H, $OCH_2CH_2$$\underline{CH_3}$)

δ_F/ppm (376 MHz, $CDCl_3$): -113.42

δ_C/ppm (100 MHz, $CDCl_3$): 165.30, 163.07, 161.97, 161.92, 161.82, 157.54, 157.10, 156.05, 155.94, 154.89, 134.65, 134.58, 133.72, 127.10, 127.08, 118.23, 118.19, 114.12, 114.10, 112.56, 112.33, 110.54, 106.61, 105.03, 99.71, 70.44, 56.30, 55.62, 22.53, 10.64

MS = $[M+H]^+$: Calculated for $C_{25}H_{23}NO_9F$: 500.1357. Found: 500.1361; Difference: 0.8 ppm

16.4.2 3-Methoxy-4-((3-fluoro-4-nitrophenoxy)carbonyl)phenyl 2-butoxy-4-methoxybenzoate (J.4)

Yield: 0.025 g, 12.1 %. RF: 0.21 (100 % dichloromethane).

T_{CrI} 96 °C T_{N_FI} (48 °C)

ν_{max}/cm^{-1}: 2955 ($-CH_3$), 2871 ($-CH_2$), 1751 (COOR), 1701 (COOR), 1603 (Ar C=C), 1524 (NO_2), 1260 (C-O-C), 1209 (C-F)

δ_H/ppm (400 MHz, $CDCl_3$): 8.17 (appt t, *J* = 9.0 Hz, 1H, Ar-H), 8.09 (d, *J* = 8.6 Hz, 1H, Ar-H), 8.05 (d, *J* = 8.7 Hz, 1H, Ar-H), 7.28 (m, 1H, Ar-H), 7.21 (ddd, *J* = 9.0, 2.4, 1.3 Hz, 1H, Ar-H), 6.94 (m, 2H, Ar-H), 6.56 (dd, *J* = 8.7, 2.4 Hz, 1H, Ar-H), 6.53 (d, *J* = 2.4 Hz, 1H, Ar-H), 4.07 (t, *J* = 6.4 Hz, 2H, $O\underline{CH_2}(CH_2)_2CH_3$), 3.95 (s, 3H, OCH_3), 3.89 (s, 3H, OCH_3), 1.84 (m, 2H, $OCH_2\underline{CH_2}CH_2CH_3$), 1.54 (m, 2H, $O(CH_2)_2\underline{CH_2}CH_3$), 0.96 (t, *J* = 7.4 Hz, 3H, $O(CH_2)_3\underline{CH_3}$)

δ_F/ppm (376 MHz, $CDCl_3$): -113.43

δ_C/ppm (100 MHz, $CDCl_3$): 165.30, 163.06, 161.97, 161.92, 161.82, 157.50, 157.10, 156.05, 155.94, 154.89, 134.65, 134.58, 133.71, 127.10, 127.08, 118.23, 118.19, 114.11, 114.10, 112.56, 112.33, 110.53, 106.60, 105.00, 99.71, 68.64, 56.30, 55.61, 31.15, 19.22, 13.82

MS = $[M+H]^+$: Calculated for $C_{26}H_{25}NO_9F$: 514.1513. Found: 514.1519; Difference: 1.2 ppm

16.4.3 3-Methoxy-4-((3-fluoro-4-nitrophenoxy)carbonyl)phenyl 4-methoxy 2-pentoxybenzoate (J.5)

Yield: 0.020 g, 4.9 %. RF: 0.21 (100 % dichloromethane).

T_{CrI} 112 °C T_{N_FI} (36 °C)

ν_{max}/cm^{-1}: 2953 ($-CH_3$), 2930 ($-CH_2$), 2869 ($-CH_2$), 1751 (COOR), 1702 (COOR), 1602 (Ar C=C), 1527 (NO_2), 1263 (C-O-C), 1209 (C-F)

δ_H/ppm (400 MHz, $CDCl_3$): 8.18 (appt t, *J* = 9.1 Hz, 1H, Ar-H), 8.10 (d, *J* = 8.7 Hz, 1H, Ar-H), 8.05 (d, *J* = 8.8 Hz, 1H, Ar-H), 7.28 (m, 1H, Ar-H), 7.21 (ddd, *J* = 9.1, 2.4, 1.3 Hz, 1H, Ar-H), 6.93 (m, 2H, Ar-H), 6.57 (dd, *J* = 8.7, 2.3 Hz, 1H, Ar-H), 6.52 (d, *J* = 2.3 Hz, 1H, Ar-H), 4.06 (t, *J* = 6.5 Hz, 2H, $O\underline{CH_2}(CH_2)_3CH_3$), 3.95 (s, 3H, OCH_3), 3.89 (s, 3H, OCH_3), 1.86 (m, 2H, $OCH_2\underline{CH_2}(CH_2)_2CH_3$), 1.48 (m, 2H, $OCH_2CH_2\underline{CH_2}CH_2CH_3$), 1.48 (m, 2H, $O(CH_2)_3\underline{CH_2}CH_3$), 0.89 (t, *J* = 7.3 Hz, 3H, $O(CH_2)_4\underline{CH_3}$)

δ_F/ppm (376 MHz, $CDCl_3$): -113.39

δ_C/ppm (100 MHz, $CDCl_3$): 165.30, 163.10, 161.95, 161.91, 161.83, 157.54, 157.11, 156.05, 155.94, 154.89, 134.67, 134.59, 133.71, 127.10, 127.08, 118.23, 118.19, 114.11, 114.10 112.57, 112.33, 110.55, 106.61, 105.01, 99.72, 68.97, 56.30, 55.62, 28.81, 28.14, 22.39, 14.01

MS = $[M+H]^+$: Calculated for $C_{27}H_{27}NO_9F$: 528.1670. Found: 528.1684; Difference: 2.7 ppm

16.4.4 3-Methoxy-4-((3-fluoro-4-nitrophenoxy)carbonyl)phenyl 2-hexyloxy-4-methoxybenzoate (J.6)

Yield: 0.066 g, 19.6 %. RF: 0.31 (100 % dichloromethane).

T_{CrI} 99 °C T_{N_FI} (31 °C)

ν_{max}/cm^{-1}: 2941 (-CH_2), 1753 (COOR), 1708 (COOR), 1603 (Ar C=C), 1527 (NO_2), 1263 (C-O-C), 1209 (C-F)

δ_H/ppm (400 MHz, $CDCl_3$): 8.18 (appt t, *J* = 9.0 Hz, 1H, Ar-H), 8.09 (d, *J* = 8.5 Hz, 1H, Ar-H), 8.05 (d, *J* = 8.7 Hz, 1H, Ar-H), 7.28 (m, 1H, Ar-H), 7.21 (ddd, *J* = 9.0, 2.4, 1.3 Hz, 1H, Ar-H), 6.94 (m, 2H, Ar-H), 6.56 (dd, *J* = 8.7, 2.3 Hz, 1H, Ar-H), 6.52 (d, *J* = 2.3 Hz, 1H, Ar-H), 4.06 (t, *J* = 6.5 Hz, 2H, $O\underline{CH_2}(CH_2)_4CH_3$), 3.95 (s, 3H, OCH_3), 3.89 (s, 3H, OCH_3), 1.86 (m, 2H, $OCH_2\underline{CH_2}(CH_2)_3CH_3$), 1.50 (m, 2H, $O(CH_2)_2\underline{CH_2}(CH_2)_2CH_3$), 1.31 (m, 4H, $O(CH_2)_3(\underline{CH_2})_2CH_3$), 0.87 (t, *J* = 7.1 Hz, 3H, $O(CH_2)_5\underline{CH_3}$)

δ_F/ppm (376 MHz, $CDCl_3$): -113.42

δ_C/ppm (100 MHz, $CDCl_3$): 165.30, 163.10, 161.95, 161.92, 161.82, 157.54, 157.11, 156.05, 155.94, 154.89, 134.66, 134.58, 133.71, 127.10, 127.08, 118.23, 118.19, 114.11, 114.10, 112.57, 112.33, 110.56, 106.61, 105.01, 99.72, 68.98, 56.30, 55.62, 31.51, 29.08, 25.67, 22.58, 14.01

MS = $[M+H]^+$: Calculated for $C_{28}H_{29}NO_9F$: 542.1826. Found: 542.1832; Difference: 1.1 ppm

16.4.5 3-Methoxy-4-((3-fluoro-4-nitrophenoxy)carbonyl)phenyl 2-heptyloxy-4-methoxybenzoate (J.7)

Yield: 0.188 g, 47.0 %. RF: 0.18 (100 % dichloromethane).

T_{CrI} 82 °C T_{N_FI} (29 °C)

ν_{max}/cm^{-1}: 2953 (-CH_3), 2912 (-CH_2), 1747 (COOR), 1717 (COOR), 1606 (Ar C=C), 1523 (NO_2), 1262 (C-O-C), 1203 (C-F)

δ_H/ppm (400 MHz, $CDCl_3$): 8.17 (appt t, *J* = 9.0 Hz, 1H, Ar-H), 8.09 (d, *J* = 8.6 Hz, 1H, Ar-H), 8.05 (d, *J* = 8.8 Hz, 1H, Ar-H), 7.29 (m, 1H, Ar-H), 7.21 (ddd, *J* = 9.0, 2.4, 1.3 Hz, 1H, Ar-H), 6.94 (m, 2H, Ar-H), 6.56 (dd, *J* = 8.8, 2.4 Hz, 1H, Ar-H), 6.52 (d, *J* = 2.4 Hz, 1H, Ar-H), 4.06 (t, *J* = 6.5 Hz, 2H, $O\underline{CH_2}(CH_2)_5CH_3$), 3.95 (s, 3H, OCH_3), 3.89 (s, 3H, OCH_3), 1.86 (m, 2H, $OCH_2\underline{CH_2}(CH_2)_4CH_3$), 1.49 (m, 2H, $O(CH_2)_2\underline{CH_2}(CH_2)_3CH_3$), 1.27 (m, 6H, $O(CH_2)_3(\underline{CH_2})_3CH_3$), 0.86 (t, *J* = 7.0 Hz, 3H, $O(CH_2)_6\underline{CH_3}$))

δ_F/ppm (376 MHz, $CDCl_3$): -113.42

δ_C/ppm (100 MHz, $CDCl_3$): 165.30, 163.12, 161.94, 161.91, 161.82, 157.54, 157.11, 156.05, 155.94, 154.89, 134.66, 134.58, 133.71, 127.10, 127.08, 118.23, 118.19, 114.10, 114.09, 112.56, 112.33, 110.56, 106.60, 105.01, 99.73, 68.98, 56.30, 55.62, 31.75, 29.13, 29.01, 25.95, 22.59, 14.08

MS = $[M+H]^+$: Calculated for $C_{29}H_{31}NO_8F$: 556.1983. Found: 556.1998; Difference: 2.7 ppm

Supplementary Figures

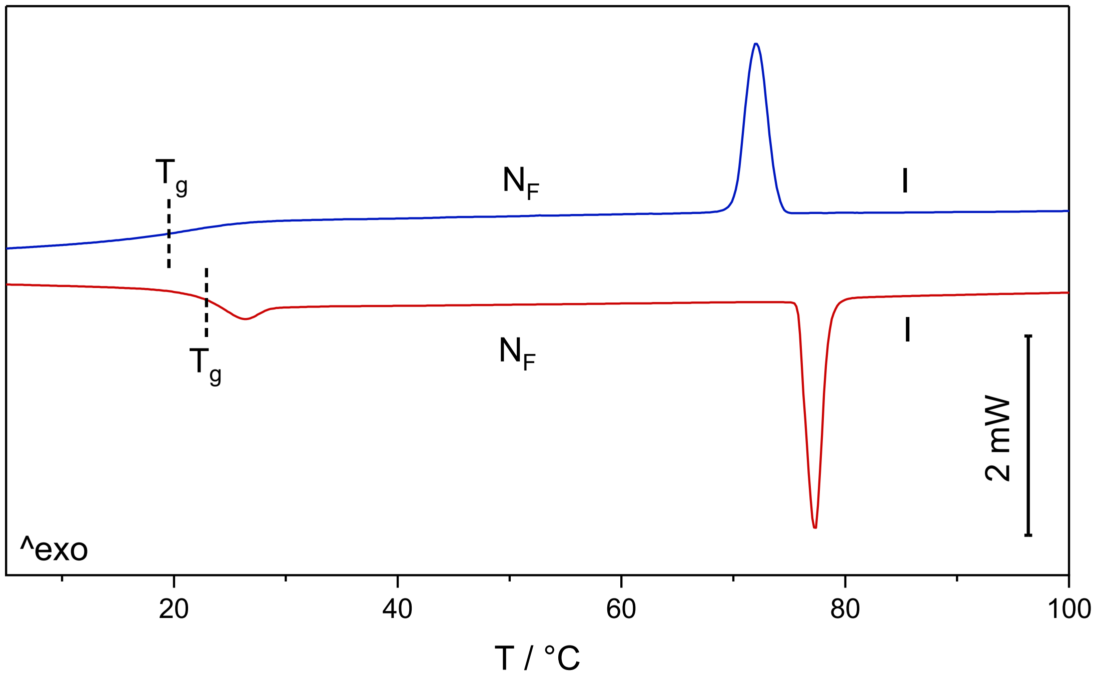

Figure SI4. DSC traces obtained for K.2. Cooling from isotropic phase (top, blue) and reheat (bottom, red). Melting point on initial heat = 154 °C.

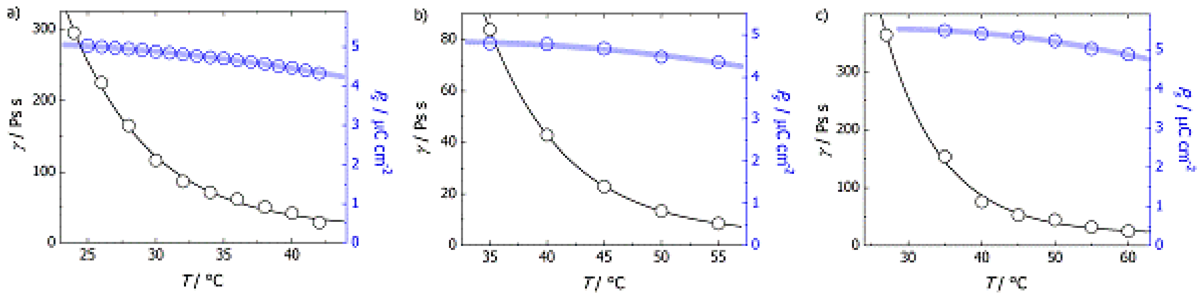

Figure SI5. Rotation viscosity and spontaneous electric polarisation for a range of compounds with a seven carbon long lateral chain. Rotational viscosity (black circles) and spontaneous electric polarization (blue circles) measured vs. temperature in the N_F phase of compounds (a) B.7, (b) C.7 and (c) D.7. Lines are a guide for the eye of the trends.

Supplementary Tables

Table SI30. Transition temperatures and associated entropy changes for the A.*m* series.

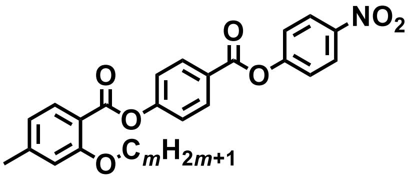

A.*m*

m	$T_{Cr\text{-}}$/ °C	T_{N_FN}/ °C<br>*T_{N_FI}/ °C	T_{NI}/ °C	$T_{N_F\text{-}Cr}$/ °C	$\Delta S_{Cr\text{-}}/R$	$\Delta S_{N_FN}/R$<br>*$\Delta S_{N_FI}/R$	$\Delta S_{NI}/R$
[b]1	158	[a]128	[a]156	[a]65	11.6	[a]0.26	[a]0.15
2	133	[a]102	[a]105	[a]50	9.1	[a]0.48	[a]0.08
3	132	[a]*79	-	-	9.2	[a]*0.78	-
4	136	[a]*58	-	-	13.2	[a]*0.88	-
5	120	[a]*42	-	-	12.4	[a]*0.78	-
6	97	[a]*29	-	-	8.5	[a]*0.52	-
7	60	[a]*26	-	-	9.3	[a]*0.56	-
8	81	[a]*26	-	-	9.4	[a]*0.49	-

[a]Values extracted from DSC cooling traces. [b]Reported previously.[2]

Table SI31. Transition temperatures and associated entropy changes for the B.*m* series.

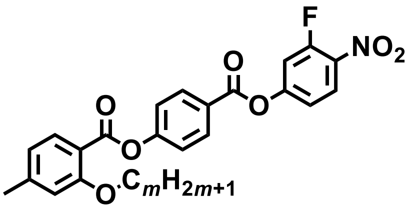

B.*m*

m	$T_{Cr\text{-}}$/ °C	T_{N_FI}/ °C	$T_{N_F\text{-}Cr}$/ °C	$\Delta S_{Cr\text{-}}/R$	$\Delta S_{N_FI}/R$
[c]1	159	[a]136	[a]90	13.1	[a]1.21
2	143	[a]108	[a]107	8.6	[b]-
3	110	[a]87	[a]76	11.3	[a]1.35
4	101	[a]69	-	11.7	[a]1.19
5	117	[a]52	-	13.6	[a]1.07
6	78	[a]43	-	10.1	[a]1.03
7	63	[a]40	-	11.3	[a]1.05
8	68	[a]37	-	11.3	[a]1.06

[a]Values extracted from DSC cooling traces. [b]Crystallisation precluded measurement. [c]Reported previously.[2]

Table SI32. Transition temperatures and associated entropy changes for the C.*m* series.

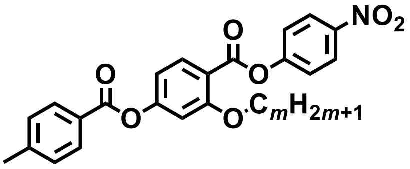

C.*m*

m	T_{Cr-}/ °C	T_{N_FN}/ °C<br>*T_{N_FI}/ °C	T_{NI}/ °C	T_{N_F-Cr}/ °C	$\Delta S_{Cr-}/R$	$\Delta S_{N_FN}/R$<br>*$\Delta S_{N_FI}/R$	$\Delta S_{NI}/R$
[b]1	164	[a]153	[a]171	[a]126	11.7	[a]0.22	[a]0.31
2	146	[a]*129	-	[a]118	13.8	[a]*1.25	-
3	161	[a]*106	-	[a]99	12.3	[a]*1.30	-
4	134	[a]*88	-	[a]74	11.6	[a]*1.24	-
5	110	[a]*73	-	-	9.7	[a]*1.12	-
6	90	[a]*65	-	-	10.6	[a]*1.23	-
7	82	[a]*60	-	-	14.3	[a]*1.16	-

[a]Values extracted from DSC cooling traces. [b]Reported previously.[2]

Table SI33. Transition temperatures and associated entropy changes for the D.*m* series.

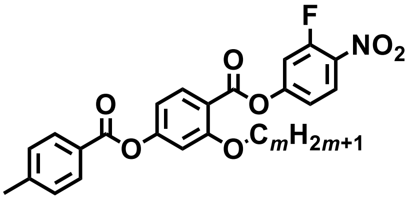

D.*m*

m	T_{Cr-}/ °C	T_{N_FI}/ °C	T_{N_F-Cr}/ °C	$\Delta S_{Cr-}/R$	$\Delta S_{N_FI}/R$
[b]1	171	[a]154	[a]138	13.1	[a]1.33
2	145	[a]128	[a]117	8.6	[a]1.71
3	136	[a]109	[a]79	11.3	[a]1.63
4	133	[a]93	-	11.7	[a]1.51
5	134	[a]81	[a]25	13.6	[a]1.46
6	89	[a]73	-	10.1	[a]1.40
7	66	[a]70	-	11.3	[a]1.37

[a]Values extracted from DSC cooling traces. [b]Reported previously.[2,3]

Table SI34. Transition temperatures and associated entropy changes for the E.*m* series.

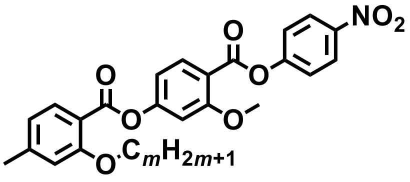

E.*m*

m	T_{Cr-}/ °C	T_{N_FI}/ °C	$T_{N_F\text{-}g}$/ °C	ΔS_{Cr-}/R	ΔS_{N_FI}/R
[b]1	147	[a]106	-	13.3	[a]1.54
2	148	[a]82	-	13.5	[a]1.49
3	92	[a]62	-	12.5	[a]1.36
4	106	[a]41	-	13.7	[a]1.24
5	78	[a]31	[a]5	12.3	[a]1.23
6	107	[a]17	-	14.3	[a]1.08
7	110	[a]14	-	14.4	[a]0.99

[a]Values extracted from DSC cooling traces. [b]Reported previously.[2]

Table SI35. Transition temperatures and associated entropy changes for the F.*m* series.

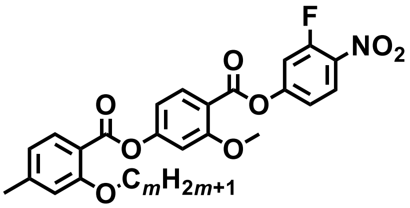

F.*m*

m	T_{Cr-}/ °C	T_{N_FI}/ °C	$T_{N_F\text{-}Cr}$/ °C	ΔS_{Cr-}/R	ΔS_{N_FI}/R
[b]1	171	[a]106	-	11.9	[a]1.48
2	123	[a]87	-	13.5	[a]1.60
3	111	[a]68	-	15.0	[a]1.53
4	96	[a]51	-	12.4	[a]1.31
5	87	[a]39	-	13.7	[a]1.18
6	81	[a]29	-	10.6	[a]1.13
7	85	[a]27	-	11.6	[a]1.09

[a]Values extracted from DSC cooling traces. [b]Reported previously.[2]

Table SI36. Transition temperatures and associated entropy changes for the G.*m* series.

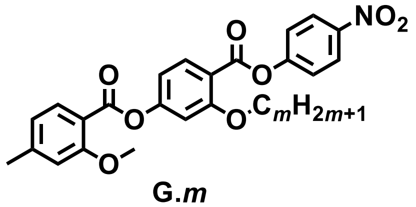

G.*m*

m	T_{Cr-}/ °C	T_{N_FI}/ °C	T_{N_F-g}/ °C	$\Delta S_{Cr-}/R$	$\Delta S_{N_FI}/R$
[b]1	147	[a]106	-	13.3	[a]1.54
2	122	[a]77	[a]21	10.0	[a]1.60
3	110	[a]62	[a]15	6.6	[a]1.51
4	124	[a]42	[a]10	13.5	[a]1.32
5	124	[a]28	[a]5	16.2	[a]1.26
6	123	[a]26	-	12.8	[a]1.11
7	101	[a]20	-	14.1	[a]1.16

[a]Values extracted from DSC cooling traces. [b]Reported previously.[2]

Table SI37. Transition temperatures and associated entropy changes for the H.*m* series.

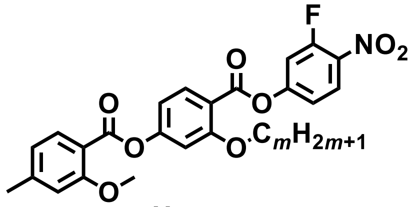

H.*m*

m	T_{Cr-}/ °C	T_{N_FI}/ °C	T_{N_F-g}/ °C	$\Delta S_{Cr-}/R$	$\Delta S_{N_FI}/R$
[b]1	171	[a]106	-	11.9	[a]1.48
2	144	[a]88	[a]24	14.9	[a]1.63
3	134	[a]73	[a]15	15.1	[a]1.81
4	100	[a]56	[a]6	9.6	[a]1.18
5	101	[a]43	[a]4	13.8	[a]1.19
6	86	[a]40	-	12.1	[a]1.20
7	93	[a]37	-	12.0	[a]1.01

[a]Values extracted from DSC cooling traces. [b]Reported previously.[2]

Table SI38. Transition temperatures and associated entropy changes for the I.*m* series.

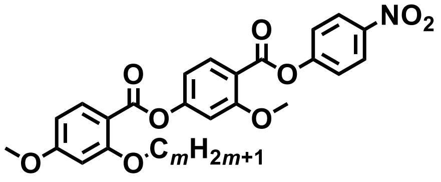

I.*m*

m	T_{Cr-}/ °C	$T_{N_F I}$/ °C	T_{N_F-Cr}/ °C<br>*T_{I-Cr}/ °C<br>‡T_{N_F-g}/ °C	ΔS_{Cr-}/R	$\Delta S_{N_F I}$/R
[d]1	167	[a]104	[a]32	15.1	[a]1.34
2	145	[a]81	-	13.5	[a]1.13
3	134	[a]62	-	13.3	[a]1.18
4	133	[a]42	-	16.4	[a]1.12
5	137	[b]34	[a]*92	17.1	-
6	121	[a]23	[a]*60	18.1	[c]-
7	97	[a]24	[a]‡2	16.5	[a]0.99

[a]Values extracted from DSC cooling traces. [b]Measured using polarized optical microscopy.

[c]Crystallisation precluded measurement. [d]Reported previously.[4]

Table SI39. Transition temperatures and associated entropy changes for the J.*m* series.

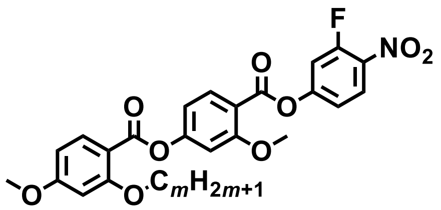

J.*m*

m	T_{Cr-}/ °C	$T_{N_F I}$/ °C	T_{N_F-Cr}/ °C<br>‡T_{N_F-g}/ °C	ΔS_{Cr-}/R	$\Delta S_{N_F I}$/R
[b]1	204	[a]99	-	15.8	[a]1.23
2	174	[a]82	[a]78	15.7	[a]0.86
3	136	[a]66	-	14.1	[a]1.20
4	96	[a]48	-	13.3	[a]1.11
5	112	[a]36	-	18.5	[a]0.91
6	99	[a]31	-	16.0	[a]0.89
7	82	[a]29	[a]‡0	10.1	[a]0.82

[a]Values extracted from DSC cooling traces. [b]Reported previously.[4]

Table SI40. Transition temperatures and associated entropy changes for the K.*m* series.

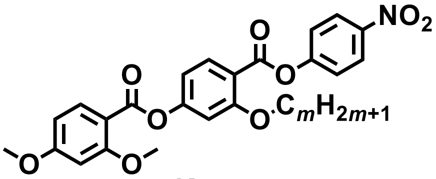

K.*m*

m	T_{Cr-}/ °C	T_{N_FI}/ °C	$T_{N_F\text{-}g}$/ °C	ΔS_{Cr-}/R	ΔS_{N_FI}/R
[b]1	167	[a]104	[a]32	15.1	[a]1.34
2	154	[a]72	[a]22	15.7	[a]1.27
3	133	[a]51	[a]20	11.0	[a]1.22
4	139	[a]28	[a]16	12.8	[a]1.09
5	141	[a]16	-	17.4	[a]0.79
6	114	[a]10	-	12.8	[a]0.52
7	80	[a]14	-	15.8	[a]1.02

[a]Values extracted from DSC cooling traces. [b]Reported previously.[4]

Table SI41. Transition temperatures and associated entropy changes for the L.*m* series.

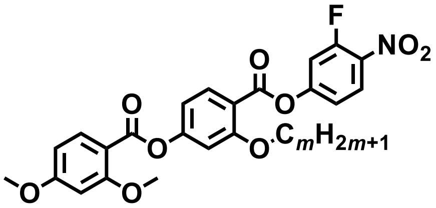

L.*m*

m	T_{Cr-}/ °C	T_{N_FI}/ °C	$T_{N_F\text{-}g}$/ °C	ΔS_{Cr-}/R	ΔS_{N_FI}/R
[b]1	204	[a]99	-	15.8	[a]1.23
2	154	[a]77	-	14.9	[a]1.32
3	125	[a]60	-	13.4	[a]1.32
4	109	[a]42	[a]10	11.7	[a]1.10
5	118	[a]35	-	8.6	[a]1.13
6	73	[a]30	[a]6	12.0	[a]0.94
7	84	[a]29	-	15.7	[a]0.98

[a]Values extracted from DSC cooling traces. [b]Reported previously.[4]

Supplementary References

1 N. Tufaha, E. Cruickshank, D. Pociecha, E. Gorecka, J. M. D. Storey and C. T. Imrie, *Chem. – A Eur. J.*, 2023, **29**, e202300073.

2 E. Cruickshank, R. Walker, M. M. Majewska, E. Gorecka, D. Pociecha, J. M. D. Storey and C. T. Imrie, *ACS Omega*, 2025, **10**, 23609–23619.

3 E. Cruickshank, P. Rybak, M. M. Majewska, S. Ramsay, C. Wang, C. Zhu, R. Walker, J. M. D. Storey, C. T. Imrie, E. Gorecka and D. Pociecha, *ACS Omega*, 2023, **8**, 36562–36568.

4 E. Cruickshank, N. Tufaha, R. Walker, S. Brown, E. Gorecka, D. Pociecha, J. M. D. Storey and C. T. Imrie, *Liq. Cryst.*, 2024, **51**, 401–415.
