# Supplementary material for: Investigating room temperature ferroelectric nematogens and their structure-property relationships
Source: Nat Commun. 2026 Feb 20;17:2965. doi: 10.1038/s41467-026-69484-z (PMC13035879; doi:10.1038/s41467-026-69484-z)
Supplement: Supplementary file 2 — Description of Additional Supplementary Files [file 41467_2026_69484_MOESM2_ESM.pdf]

Description of Additional Supplementary Files

File Name: Supplementary Data 1

Description: Optimised atomic coordinates for the molecular modelling reported in Figure 7. Calculation results tabulated for a) A.1, b) C.1, c) E.1 and d) F.1.
